# Supplementary material for: Intermolecular diastereoselective annulation of azaarenes into fused N-heterocycles by Ru(II) reductive catalysis
Source: Nat Commun. 2022 May 2;13:2393. doi: 10.1038/s41467-022-29985-z (PMC9061824; doi:10.1038/s41467-022-29985-z)
Supplement: Supplementary file 1 — Supplementary Information [file 41467_2022_29985_MOESM1_ESM.pdf]

Supplementary Information

Intermolecular Diastereoselective Annulation of Azaarenes into Fused N-heterocycles by Ru(II) Reductive Catalysis

He Zhao,[1,4] Yang Wu,[1,4] Chenggang Ci,[2] Zhenda Tan,[1] Jian Yang,[1] Huanfeng Jiang,[1] Pierre. H. Dixneuf,[3] and Min Zhang[1✉]

[1] Key Lab of Functional Molecular Engineering of Guangdong Province, School of Chemistry and Chemical Engineering, South China University of Technology, Guangzhou 510641, China.

[2] Key Laboratory of Computational Catalytic Chemistry of Guizhou Province, Department of Chemistry and Chemical Engineering, Qiannan Normal University for Nationalities, Duyun 558000, China

[3] University of Rennes, ISCR, UMR CNRS 6226, 35000 Rennes, France.

[4] These authors contributed equally: He Zhao, Yang Wu.

✉Correspondence: minzhang@scut.edu.cn (M.Z.).

Table of contents

General information S2

Supplementary methods S3-S9

- Optimization of reaction conditions
- Substrates Preparation
- Preparation of intermediates
- Typical procedure for the synthesis of product **P1**
- Synthetic utility

Supplementary discussion S9-18

- Crystal data of **P1**
- NOESY spectra of compounds **P1**, **P8** and **P51**
- Control experiments
- Deuterium experiments
- Detection of the ester products by GC-MS
- Computational Details

Analytical data of the obtained compounds S19-S47

NMR spectra of the obtained compounds S48-S134

Supplementary references S135

General information

All the obtained products were characterized by melting points (m.p.), ^{1}H-NMR, ^{13}C-NMR. Melting points (m.p.) were performed using a Büchi AG M-565. ^{1}H-NMR, ^{13}C-NMR and NOESY spectra were obtained on Bruker-400 or Bruker-500 and referenced to 7.26 ppm for chloroform solvent or 2.54 ppm for dimethyl sulfoxide solvent with TMS as internal standard (0 ppm). Chemical shifts were reported in parts per million (ppm, δ) downfield from tetramethylsilane. Proton coupling patterns are described as singlet (s), doublet (d), triplet (t), multiplet (m). Analytical HPLC was run with an Agilent 1260 Infinity, equipped with a Daicel CHIRALPAK IA (4.6 mm × 250 mm) and a Agilent LC1260 II DAD-G7115A detector. The high resolution mass spectrums were recorded on a Bruker maXis impact mass spectrometer. X-Ray diffraction data of one these crystals were collected on a Bruker SMART diffractometer. And the structures were solved by direct methods SHELXS-2014.

All the reagents were purchased from *Bide Pharmatech Ltd.* and *Energy Chemical*. All solvents were purchased from *Greagent* (Shanghai Titansci incorporated company) and used without further purification. All reactions were heated by metal sand bath (WATTCAS, LAB-500, https://www.wattcas.com). Column chromatography was performed on silica gel (200-300 mesh). Reactions were monitored by using thin layer chromatography (TLC) (Qingdao Jiyida silica gel reagent factory GF254).

Noteworthy, the employed N-heteroarenes are named **A'**, the N-heteroarenium salts are named **A**, the anilines are named **B**, and the obtained products are named **P**.

Supplementary Methods

Optimization of reaction conditions

Supplementary Table 1. Optimization of reaction conditions[a]

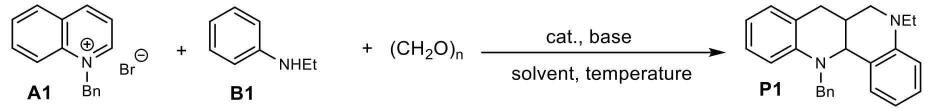

Entry	Catalyst	Base	Solvent	T (°C)	Yields of P1[b]
1	$Ru_3(CO)_{12}$/Xantphos	$Mg(OMe)_2$	methanol	65	0
2	$RuHCl(CO)(PPh_3)_3$	$Mg(OMe)_2$	methanol	65	0
3	$Co_2(CO)_8$	$Mg(OMe)_2$	methanol	65	0
4	$FeCl_3$	$Mg(OMe)_2$	methanol	65	0
5	$MnBr(CO)_5$/Xantphos	$Mg(OMe)_2$	methanol	65	0
6	$NiCl_2(PPh)_2$	$Mg(OMe)_2$	methanol	65	0
7	[Ru(*p*-cymene)Cl_2]$_2$	*t*-BuONa	methanol	65	0
8	[Ru(*p*-cymene)Cl_2]$_2$	NaOH	methanol	65	0
9	[Ru(*p*-cymene)Cl_2]$_2$	$CsCO_3$	methanol	65	0
10	[Ru(*p*-cymene)Cl_2]$_2$	Et_3N	methanol	65	0
11	[Ru(*p*-cymene)Cl_2]$_2$	$MgCl_2$	methanol	65	0
12	[Ru(*p*-cymene)Cl_2]$_2$	NaOTf	methanol	65	0

[a] Unless otherwise stated, the reaction in MeOH (1 mL) was performed with **A1** (0.2 mmol), **B1** (0.2 mmol), cat. (1 mol %), base (0.75 eq), $(CH_2O)_n$ (10 eq) at 65 °C for 18 h under N_2 protection.

[b] NMR yield by using anisole as the internal standard.

Substrate preparation

Synthesis of N-heteroarenium Bromide Salts

Method 1: Synthesis of compound **A1**: quinoline (3 mmol), benzyl bromide (6 mmol) and acetone (5 mL) were introduced in a flask (50 mL). The resulting mixture was then stirred at room temperature for 24 h. Then, the solvent was removed. The reaction mixture was washed with small amount of diethyl ether and finally dried under vacuum to get the salt **A1**.

Method 2: Synthesis of compound **A16**: quinoline (3 mmol), (2-bromoethyl)benzene (3 mmol) and toluene (3 mL) were introduced in a flask (50 mL). The resulting mixture was stirred at 110 °C for 24 h. Then, the solvent was removed. The reaction mixture was washed with small amount of diethyl ether and finally dried under vacuum to get the salt **A16**.

(1) 1-(2-(benzyloxy)-2-oxoethyl)quinolin-1-ium bromide (**A19**)

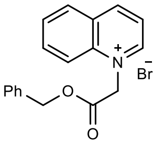

Method 1; Yellow solid; ^{1}H NMR (400 MHz, DMSO-d_6) δ 9.68 (d, J = 4.0Hz, 1H), 9.47 (d, J = 8.4 Hz, 1H), 8.56 (d, J = 8.0 Hz, 1H), 8.49 (d, J = 9.0 Hz, 1H), 8.37 – 8.23 (m, 2H), 8.08 (t, J = 7.6 Hz, 1H), 7.46 – 7.30 (m, 5H), 6.33 (s, 2H), 5.26 (s, 2H).^{13}C NMR (101 MHz, DMSO-d_6) δ 166.6, 151.7, 149.6, 138.7,

136.6, 135.4, 131.2, 130.6, 129.8, 129.0, 128.9, 128.6, 122.6, 119.4, 68.1, 57.9. HRMS (ESI): Calcd. for $C_{18}H_{16}BrNO_2$ $[M–Br]^+$: 278.1181; found: 278.1173.

(2) ethene,1,1'-(propane-1,3-diyl)bis(quinolin-1-ium) bromide (**A21**)

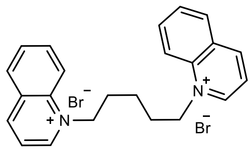

Method 1; Black solid; ^{1}H NMR (500 MHz, DMSO-*d*$_6$) δ 9.77 (d, *J* = 5.2 Hz, 1H), 9.37 (d, *J* = 8.4 Hz, 1H), 8.69 (d, *J* = 9.0 Hz, 1H), 8.58 – 8.52 (m, 1H), 8.33 – 8.22 (m, 3H), 8.08 (t, *J* = 7.6 Hz, 1H), 5.16 (t, *J* = 7.6 Hz, 3H), 2.19 – 2.03 (m, 3H), 1.59-1.63 (m, 1H). ^{13}C NMR (101 MHz, DMSO-*d*$_6$) δ 150.2, 147.8, 137.8, 136.2, 131.2, 130.3, 130.1, 122.7, 122.6, 119.6, 57.4, 29.3, 23.1. HRMS (ESI): Calcd. for $C_{23}H_{25}Br_2N_2$ $[M–Br+H]^+$: 329.2018; found: 329.2012.

Preparation of intermediates

(1) Synthesis of 1-benzyl-1,2-dihydroquinoline (int-1)

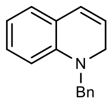

To a suspension of 1-benzylquinolin-1-ium bromide **A1** (3.0 mmol) in 25 mL of THF was slowly added lithium aluminium hydride (3.0 mmol) at room temperature. The reaction was stirred at room temperature for 20 min, then slowly quenched with 0.1 mL of water and stirred for an additional 10 min. To the reaction was added 0.2 mL 10 % sodium hydroxide solution and stirred at room temperature for an additional 10 min. The reaction was added 5% Na_2CO_3 solution and the resulting mixture was extracting with ethyl acetate, dried with anhydrous sodium sulfate, and then concentrated by removing the solvent under vacuum to give the unstable crude 1-benzyl-1,2-dihydroquinoline as a yellow oil. ^{1}H NMR (400 MHz, Chloroform-*d*) δ 7.51 – 7.38 (m, 5H), 7.12 (d, *J* = 8.0 Hz, 1H), 7.02 (d, *J* = 5.8 Hz, 1H), 6.74 (t, *J* = 7.4 Hz, 1H), 6.61 (d, *J* = 8.2 Hz, 1H), 6.50 (d, *J* = 2.2 Hz, 1H), 5.78 (dt, *J* = 9.9, 3.8 Hz, 1H), 4.50 (s, 2H), 4.33 (dd, *J* = 3.8, 2.0 Hz, 2H). ^{13}C NMR (101 MHz, $CDCl_3$) δ 145.5, 137.6, 129.3, 128.8, 127.4, 127.2, 127.1, 126.7, 122.1, 121.9, 117.0, 110.2, 53.8, 50.6. HRMS (ESI): Calcd. for $C_{16}H_{16}N$ $[M+H]^+$: 222.1277; found: 222.1276.

(2) Synthesis of 2-benzyl-1,2-dihydroisoquinoline (int-6)

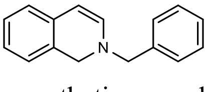

The synthetic procedure of **int-6** is similar to that of **int-1** described above. Yellow oil; ^{1}H NMR (500 MHz, $CDCl_3$) δ 7.30 – 7.24 (m, 4H), 7.22 (td, *J* = 6.6, 6.1, 2.6 Hz, 1H), 7.03 (t, *J* = 7.7 Hz, 1H), 6.89 (td, *J* = 7.4, 1.2 Hz, 1H), 6.82 (d, *J* = 7.6 Hz, 1H), 6.76 (d, *J* = 7.4 Hz, 1H), 6.17 (d, *J* = 7.4 Hz, 1H), 5.26 (d, *J* = 7.4 Hz, 1H), 4.13 (s, 2H), 4.03 (s, 2H).^{13}C NMR (126 MHz, $CDCl_3$) δ 139.0, 137.4, 134.0, 128.8, 128.3, 127.7, 127.7, 125.7, 124.9, 122.9, 98.3, 59.4, 51.1.

Typical procedure for the synthesis of product P1

Under N_2 atmosphere, [Ru(*p*-cymene)Cl_2]$_2$ (1 mol %), 1-benzylquinolin-1-ium bromide **A1** (0.2 mmol), N-ethylaniline **B1** (0.2 mmol), $Mg(OMe)_2$ (0.75 eq, 12.9 mg), $(CH_2O)_n$ (10.0 eq, 60

mg) and methanol (1 mL) were introduced in a Schlenk tube, successively. Then the Schlenk tube was closed and the resulting mixture was stirred at 55 °C for 18 h. After cooling down to room temperature, the mixture was extracted with ethyl acetate, washed with 5% Na_2CO_3 solution, dried with anhydrous sodium sulfate, and then concentrated by removing the solvent under vacuum. Finally, the residue was purified by preparative TLC on silica to give 12-benzyl-5-ethyl-5,6,6a,7,12,12a-hexahydrodibenzo[*b*,*h*][1,6]naphthyridine **P1**.

Synthetic Utility

Synthesis of Compound P81

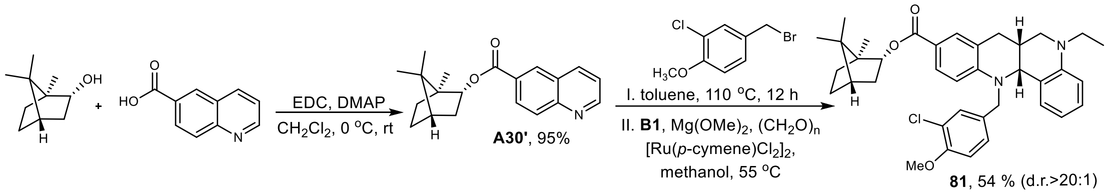

81, 54 % (d.r.>20:1)

To a suspension of quinoline-6-carboxylic acid (1 mmol) in CH_2Cl_2 was added DMAP (4-(dimethylamino)-pyridine, 2 mmol) in one portion at room temperature. The reaction mixture was stirred for 10 min at room temperature. N-(3-(Dimethylamino)propyl)-N'-ethylcarbodiimide hydrochloride (EDC, 1 mmol) was added at 0 °C. Then the resultant suspension was stirred for 10 min at 0 °C. A solution of (-)-Bomeol (0.8 mmol) in CH_2Cl_2 was added at 0 °C. The cooling bath was removed, and the reaction mixture was stirred at room temperature for 16 h. The solution was diluted by the addition of saturated aqueous NH_4Cl solution and CH_2Cl_2 at room temperature. The organic phase was dried with anhydrous sodium sulfate, and then concentrated by removing the solvent under vacuum. Finally, the residue was purified by preparative TLC on silica to give a white solid **A30'** (234.8 mg, 95% yield).[1]

Under N_2 atmosphere, **A30'** (0.2 mmol), 4-(bromomethyl)-2-chloro-1-methoxybenzene (0.2 mmol), and toluene (1.0 mL) were introduced in a Schlenk tube, successively. The Schlenk tube was closed and the resulting mixture was stirred at 110 °C for 12 h. Then the solvent was removed and to the reaction were added [Ru(*p*-cymene)Cl_2]$_2$ (1 mol%), N-ethylaniline **B1** (0.2 mmol), $Mg(OMe)_2$ (75mol %), paraformaldehyde (10 eq) and methanol (1 mL) The reaction was performed at 55 °C for 18 h. After cooling down to room temperature, the resulting mixture was extracting with CH_2Cl_2, washed with 5% Na_2CO_3 solution, dried with anhydrous sodium sulfate, and then concentrated by removing the solvent under vacuum. Finally, the residue was purified by preparative TLC on silica to give **P81** (54% yield).

Synthesis of Compound P82

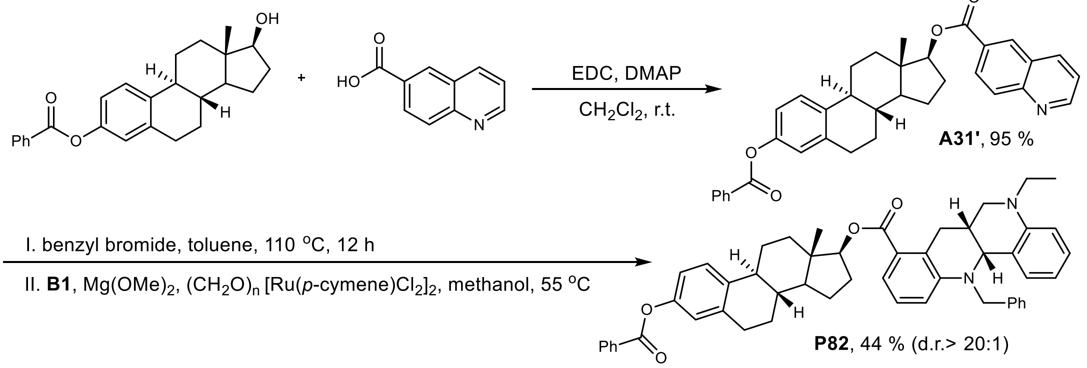

P82, 44 % (d.r.> 20:1)

To a suspension of quinoline-6-carboxylic acid (1 mmol) in CH_2Cl_2 was added DMAP (4-(dimethylamino)-pyridine, 2 mmol) in one portion at room temperature. The reaction mixture was stirred for 10 min at room temperature. N-(3-(Dimethylamino)propyl)-N'-ethylcarbodiimide hydrochloride (EDC, 1 mmol) was added at 0 °C. Then the resultant suspension was stirred for 10 min at 0 °C. A solution of 1-Adamantanol (0.8 mmol) in CH_2Cl_2 was added at 0 °C. The cooling bath was removed, and the reaction mixture was stirred at room temperature for 16 h. The solution was diluted by the addition of saturated aqueous NH_4Cl solution and CH_2Cl_2 at room temperature. The organic phase was dried with anhydrous sodium sulfate, and then concentrated by removing the solvent under vacuum. Finally, the residue was purified by preparative TLC on silica to give a white solid **A31'** (235.6 mg, 91% yield).[1]

Under N_2 atmosphere, **A31'** (0.2 mmol), benzyl bromide (0.2 mmol), and toluene (1.0 mL) were introduced in a Schlenk tube, successively. The Schlenk tube was closed and the resulting mixture was stirred at 110 °C for 12 h. Then the solvent was removed and to the reaction were added [Ru(*p*-cymene)Cl_2]$_2$ (1 mol%), N-ethylaniline **A50** (0.2 mmol), Mg(OMe)$_2$ (75 mol%), Paraformaldehyde (10 equiv) and methanol (1 mL). The reaction was performed at 55 °C for 18 h. After cooling down to room temperature, the resulting mixture was extracting with CH_2Cl_2, washed with 5% Na_2CO_3 solution, dried with anhydrous sodium sulfate, and then concentrated by removing the solvent under vacuum. Finally, the residue was purified by preparative TLC on silica to give **P82** (44% yield).

Synthesis of Compound P83

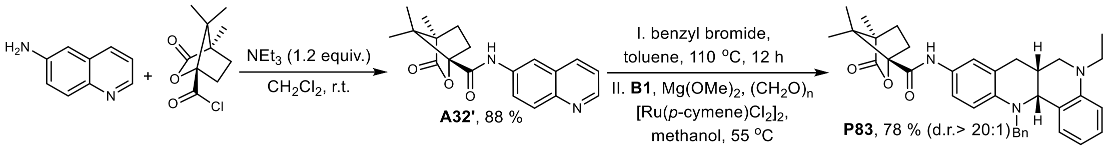

To a solution of 6-aminoquinoline (10 mmol, 1.44 g) in anhydrous CH_2Cl_2 was added triethylamine (12 mmol, 1.21 g) and (-)-(1*S*,4*R*)-Camphanoyl chloride (11 mmol, 2.38 g) at 0 °C. The reaction mixture was stirred at room temperature until the reaction was completed (monitored by TLC). Water (50 mL) was added to the mixture and extracted with CH_2Cl_2 (30 mL). The organic phase was dried over anhydrous sodium sulfate, and concentrated under reduced pressure. The residue was purified by flash chromatography on silica gel to afford **A32'** (88% yield).[2]

Under N_2 atmosphere, **A32'** (0.2 mmol), benzyl bromide (0.2 mmol), and toluene (1.0 mL) were introduced in a Schlenk tube, successively. The Schlenk tube was closed and the resulting mixture was stirred at 110 °C for 12 h. Then the solvent was removed and to the reaction were added [Ru(*p*-cymene)Cl_2]$_2$ (1 mol%), N-ethylaniline (0.2 mmol), Mg(OMe)$_2$ (75 mol%), paraformaldehyde (10 equiv) and toluene (1 mL) The reaction was performed at 55 °C for 18 h. After cooling down to room temperature, the resulting mixture was extracting with CH_2Cl_2, washed with 5% Na_2CO_3 solution, dried with anhydrous sodium sulfate, and then concentrated by removing the solvent under vacuum. Finally, the residue was purified by preparative TLC on silica to give **P83** (78% yield).

Debenzylation of compounds P29 and P51

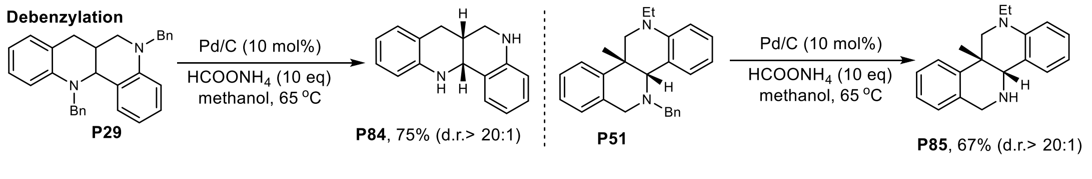

Under N_2 atmosphere, 10 mol % Pd/C, **P29** (0.2 mmol, 83.2 g), $HCOONH_4$ (2 mmol, 136 mg), and methanol (0.5 mL) were introduced in a Schlenk tube, successively. Then the Schlenk tube was closed and the resulting mixture was stirred at 65 °C for 12 h. After cooling down to room temperature, the resulting mixture was extracting with ethyl acetate, washed with H_2O, dried with anhydrous sodium sulfate, and then concentrated by removing the solvent under vacuum. Finally, the residue was purified by preparative TLC on silica to give **P84** (74% yield). Similarly, compound **P51** underwent N-debenzylation under the same reaction conditions to afforded **P85** in 67% yield.

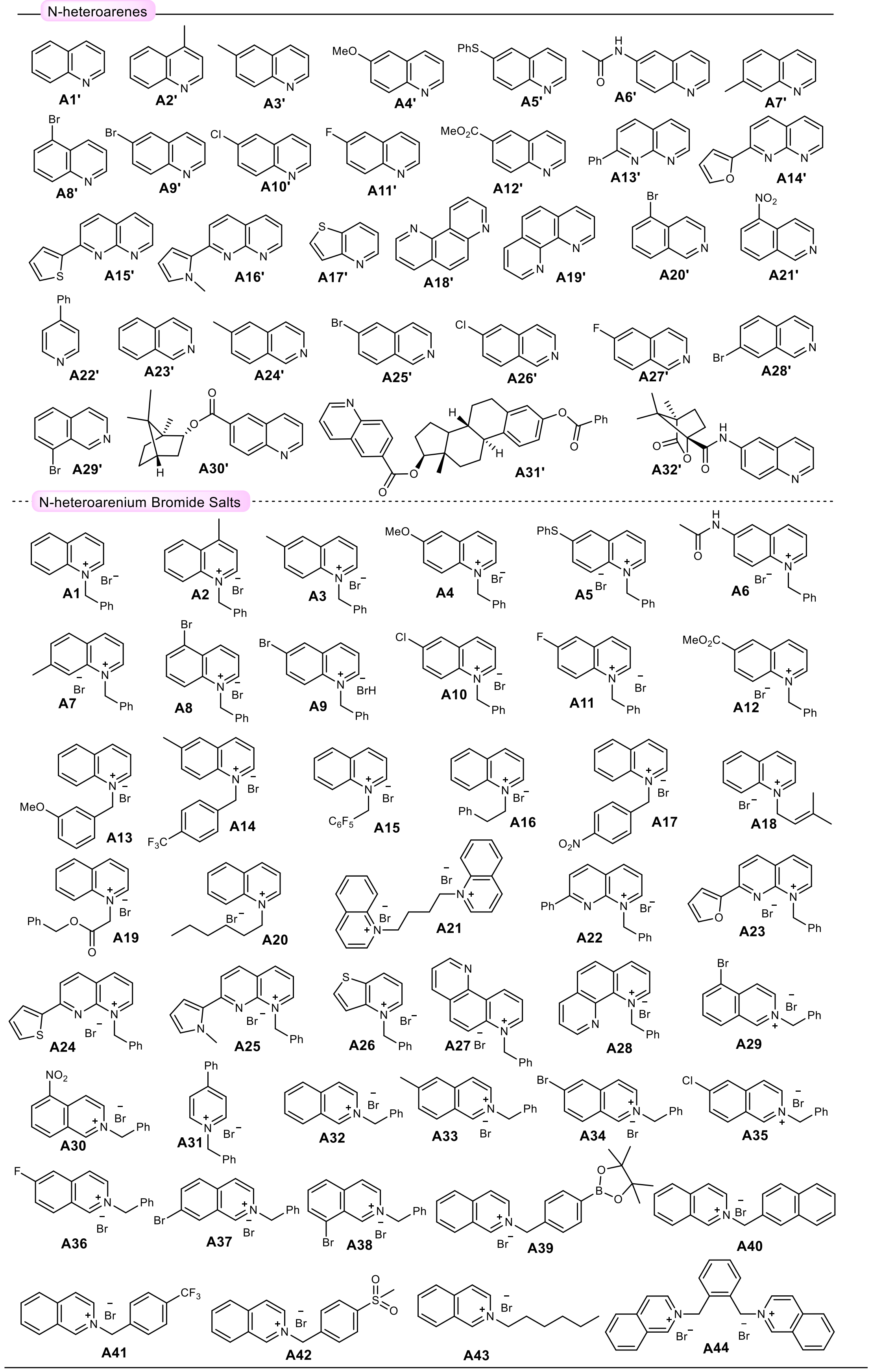

Supplementary Fig. 1. Substrates employed for the reaction.

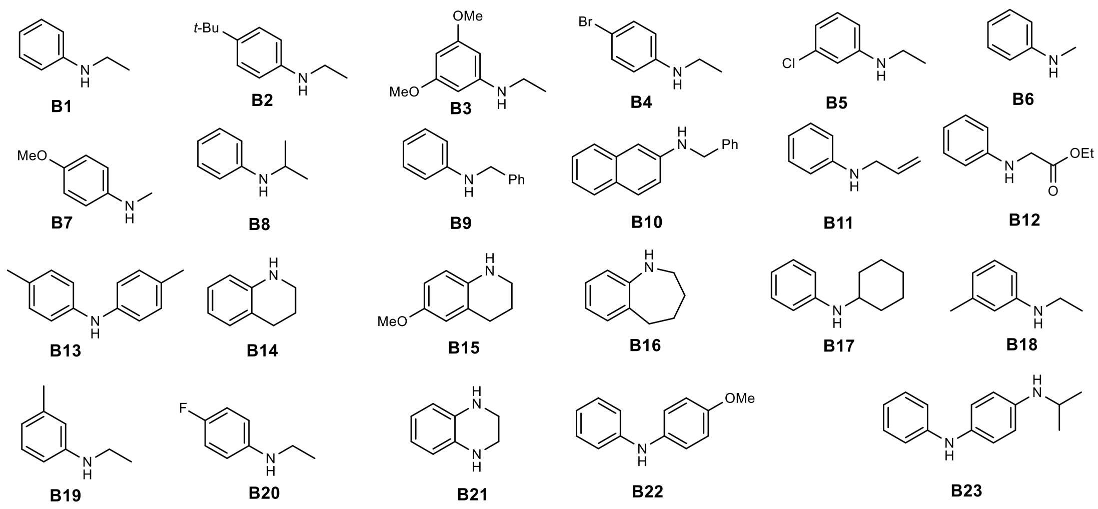

Supplementary Fig. 2. Substrate N-substituted aniline B employed for the reaction.

Supplementary Discussion

(1) Single crystal X-ray diffraction data of P1

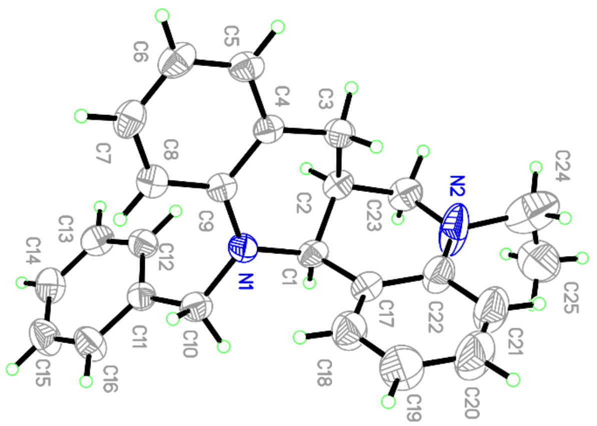

Supplementary Fig. 3 Molecular structure of P1 (CCDC 2051637) is displayed with thermal ellipsoids set at 30% probability.

Yellow block-like single crystals of **P1** were grown by layering a THF solution with n-hexane at ambient temperature. X-Ray diffraction data of one these crystals were collected on a Bruker APEX-II CCD. The measurements were performed with Mo-Kα radiation (λ = 0.71073 Å). Data were collected at 293 (2) K, using the phi and omega scans to a maximum θ value of 29.275°. The data were refined by full-matrix least-squares techniques on F^2 with SHELXL-2014. And the structures were solved by direct methods SHELXL-2014. All the non-hydrogen atoms were refined anisotropically. The hydrogen atoms were included at geometrically idealized positions. An ORTEP representation of the structure is shown below.

Supplementary Table 2. Crystal data and structure refinement for P1.

Identification code	**P1**	
Empirical formula	$C_{25}H_{26}N_2$	
Formula weight	354.48	
Temperature	293(2) K	
Wavelength	0.71073 Å	
Crystal system	Orthorhombic	
Space group	$P2_12_12_1$	
Unit cell dimensions	a = 12.8508(19) Å	α= 90°.
	b = 14.337(3) Å	β= 90°.
	c = 21.517(5) Å	γ = 90°.
Volume	3964.4(13) $Å^3$	
Z	8	
Density (calculated)	1.188 Mg/m^3	
Absorption coefficient	0.069 mm^{-1}	
F(000)	1520	
Crystal size	0.220 x 0.200 x 0.180 mm^3	
Theta range for data collection	3.415 to 29.275°.	
Index ranges	-17<=h<=11, -17<=k<=19, -27<=l<=27	
Reflections collected	19084	
Independent reflections	9204 [R(int) = 0.0465]	
Completeness to theta = 25.242°	99.6 %	
Absorption correction	Semi-empirical from equivalents	
Refinement method	Full-matrix least-squares on F^2	
Data / restraints / parameters	9204 / 233 / 543	
Goodness-of-fit on F^2	0.965	
Final R indices [I>2sigma(I)]	R1 = 0.0640, wR2 = 0.1025	
R indices (all data)	R1 = 0.1826, wR2 = 0.1425	
Absolute structure parameter	-1.6(10)	
Extinction coefficient	n/a	
Largest diff. peak and hole	0.154 and -0.177 e.$Å^{-3}$	

(2) NOESY spectra of partial obtained compounds

NOESY spectrum of P1

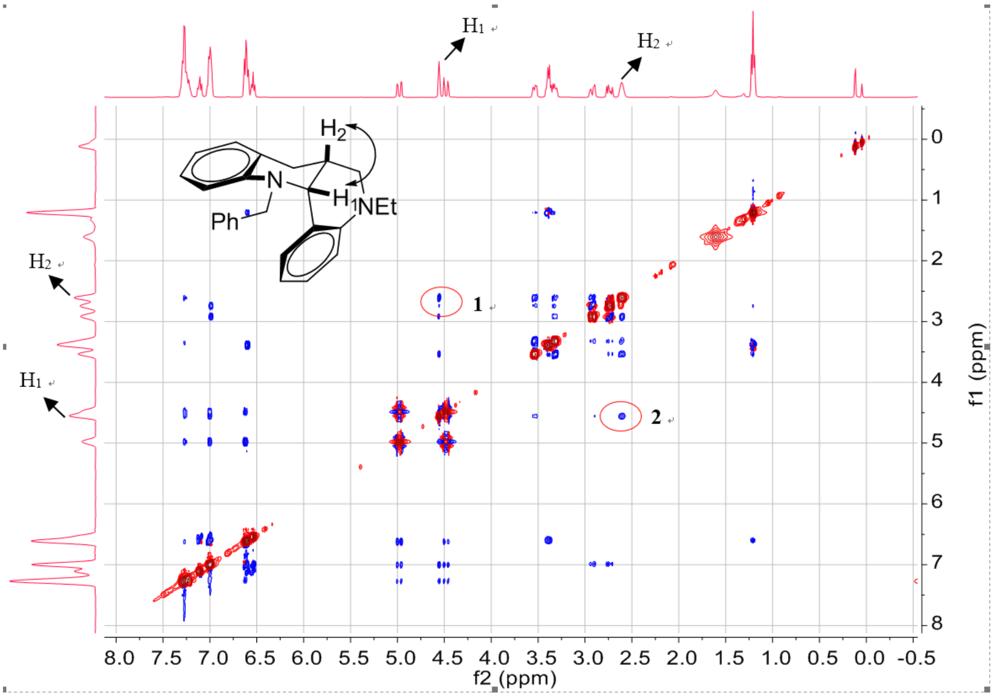

Supplementary Fig. 4. NOESY spectrum of compound P1.

NOESY spectrum of P8

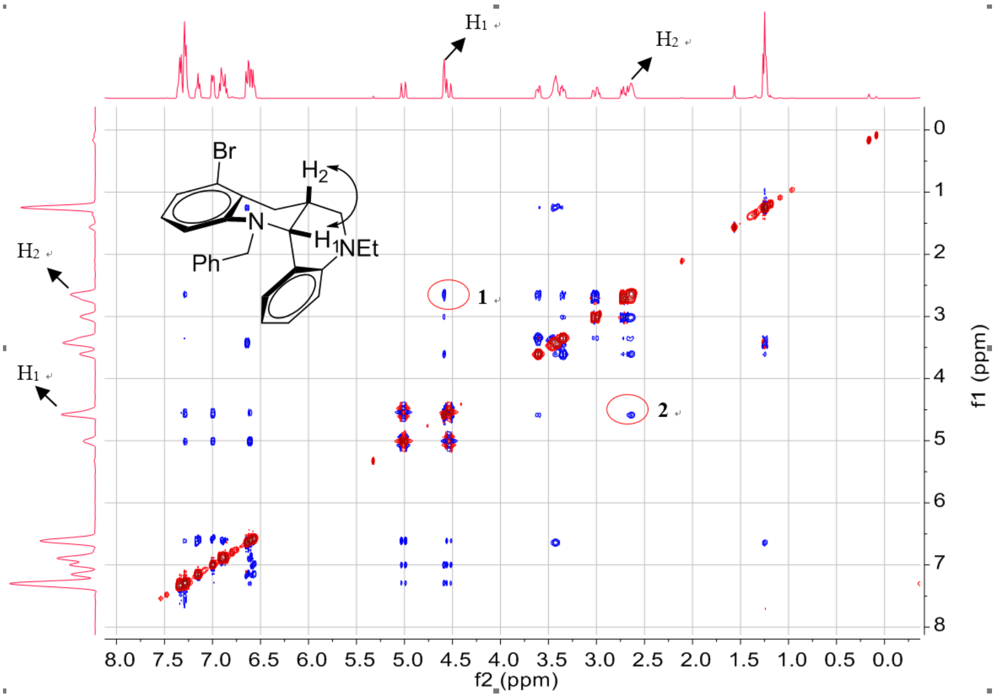

Supplementary Fig. 5 NOESY spectrum of compound P8.

NOESY spectrum of P51

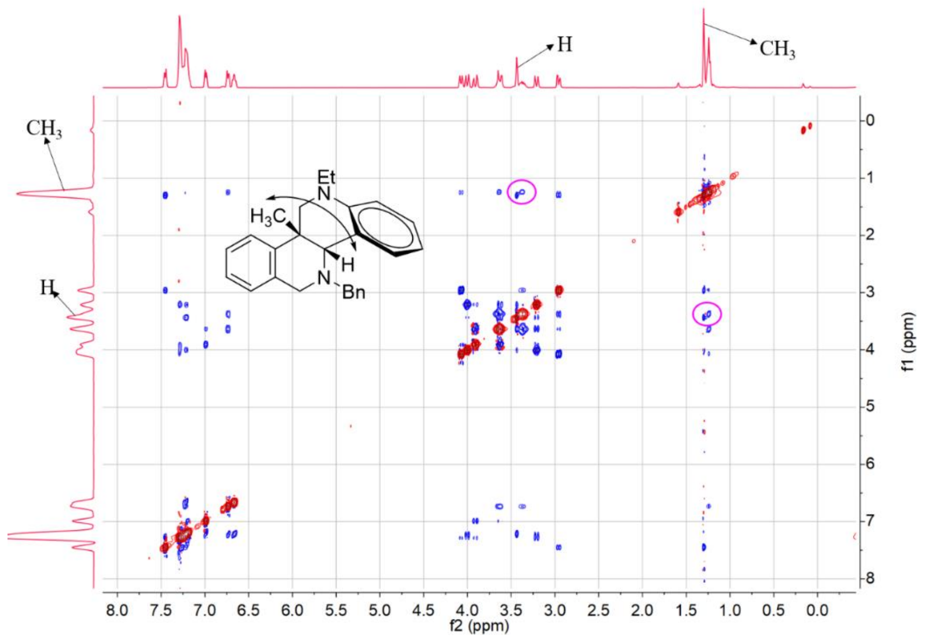

Supplementary Fig. 6 NOESY spectrum of compound P51.

(3) Control experiments

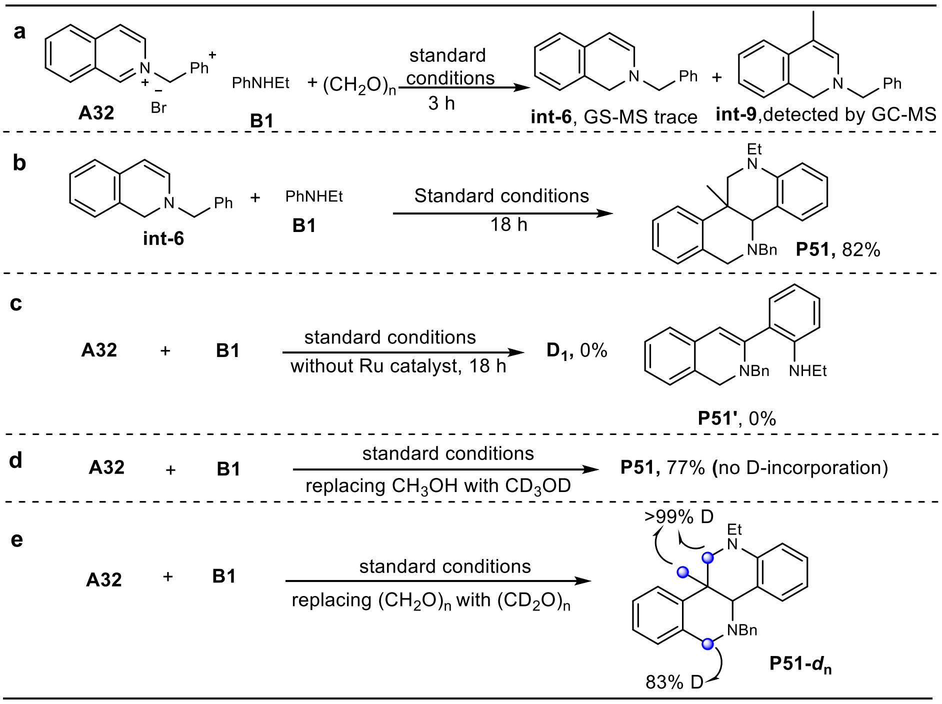

Supplementary Fig. 7 Supplementary control experiments.

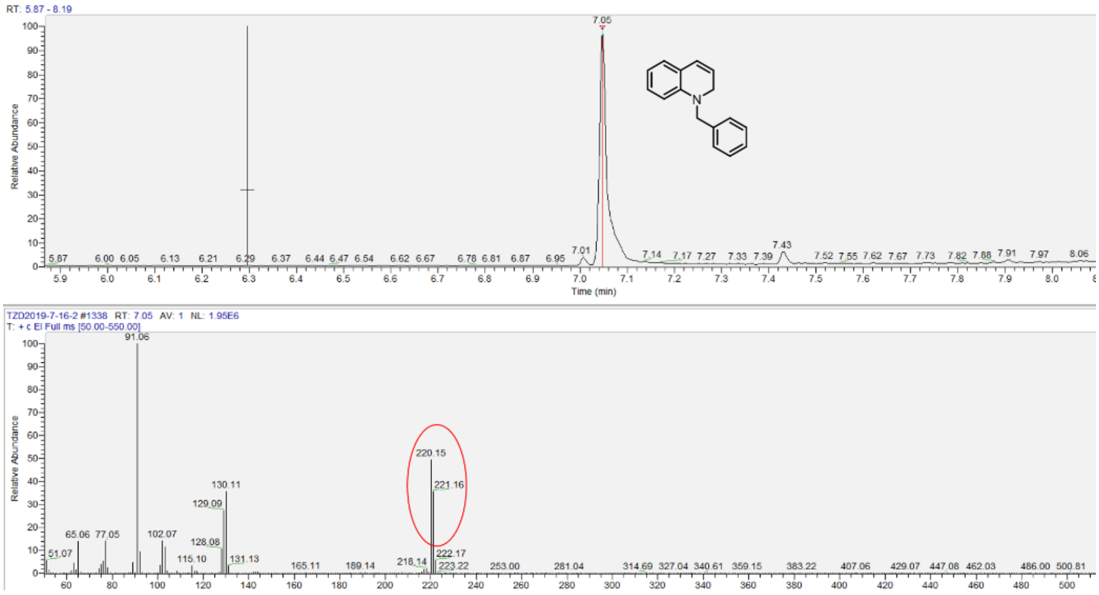

Supplementary Fig. 8 GC-MS spectrum of int-1.

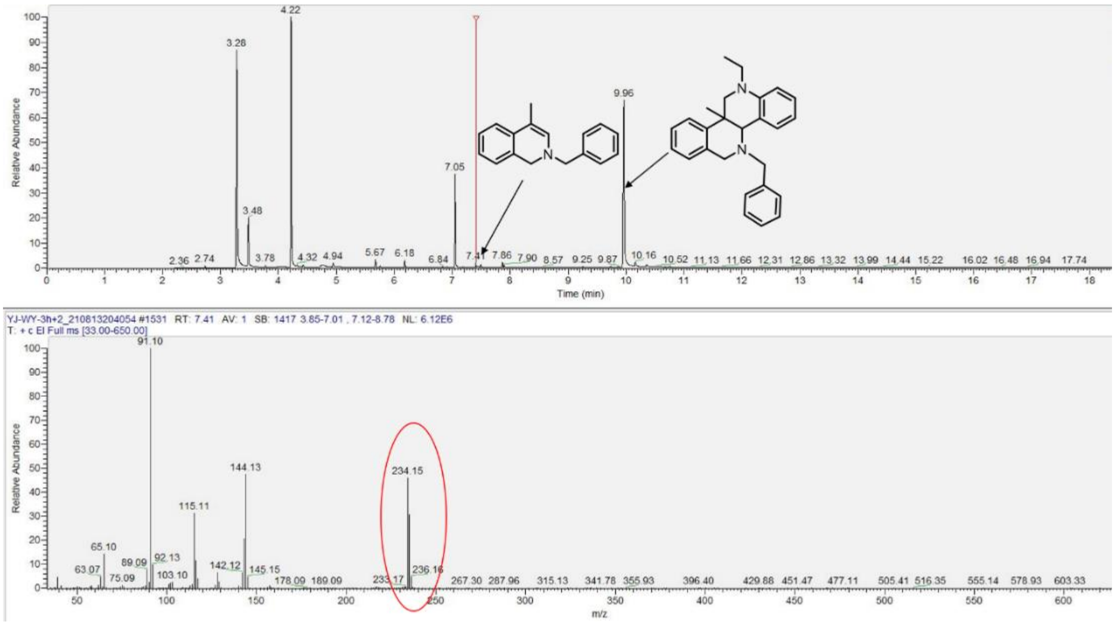

Supplementary Fig. 9 GC-MS spectrum of int-9.

(4) Deuterium Experiments

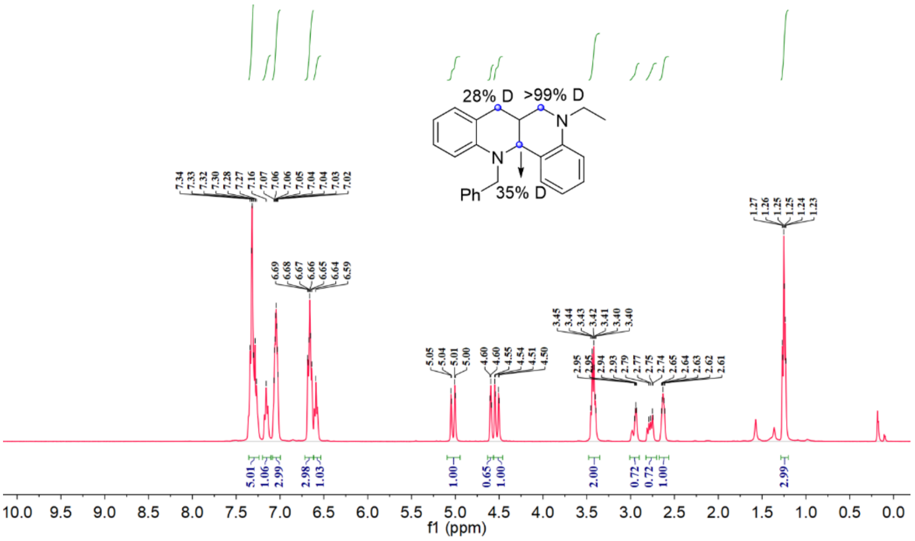

Supplementary Fig. 10 ^{1}H-NMR spectrum of P1-d_n.

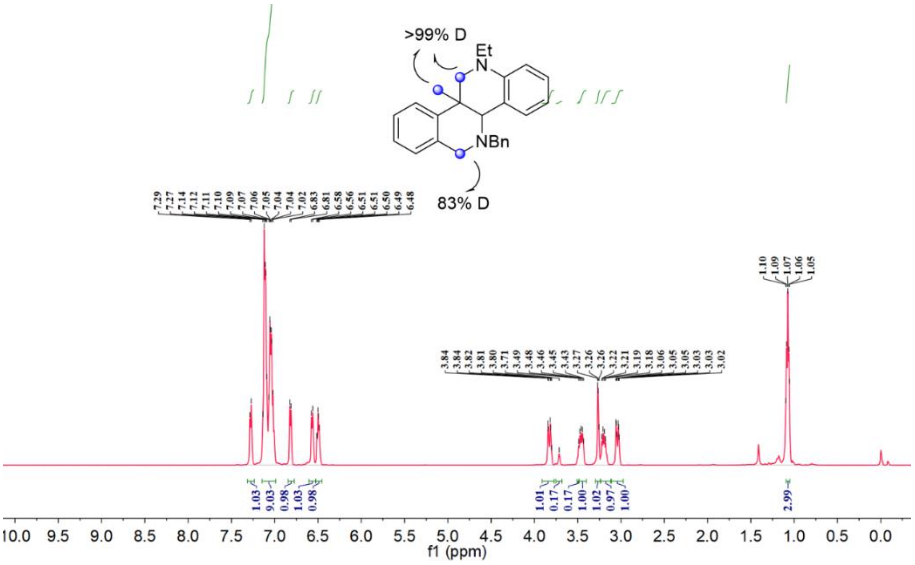

Supplementary Fig. 11 ^{1}H-NMR spectrum of P51-d_n.

(5) Detection of the ester products by GC-MS

By replacing MeOH with *t*-BuOH as the solvent, the GC-MS analysis of the model reaction shows the formation of *t*-butyl formate, which is formed via the addition of alcohol to formaldehyde followed by a base-mediated anion exchange with the ruthenium catalyst and Ru-catalyzed *β*-hydride elimination. Meanwhile, such a process generates the reductive species [HRu^{II}X], which is involved in the reductive annulation reaction.

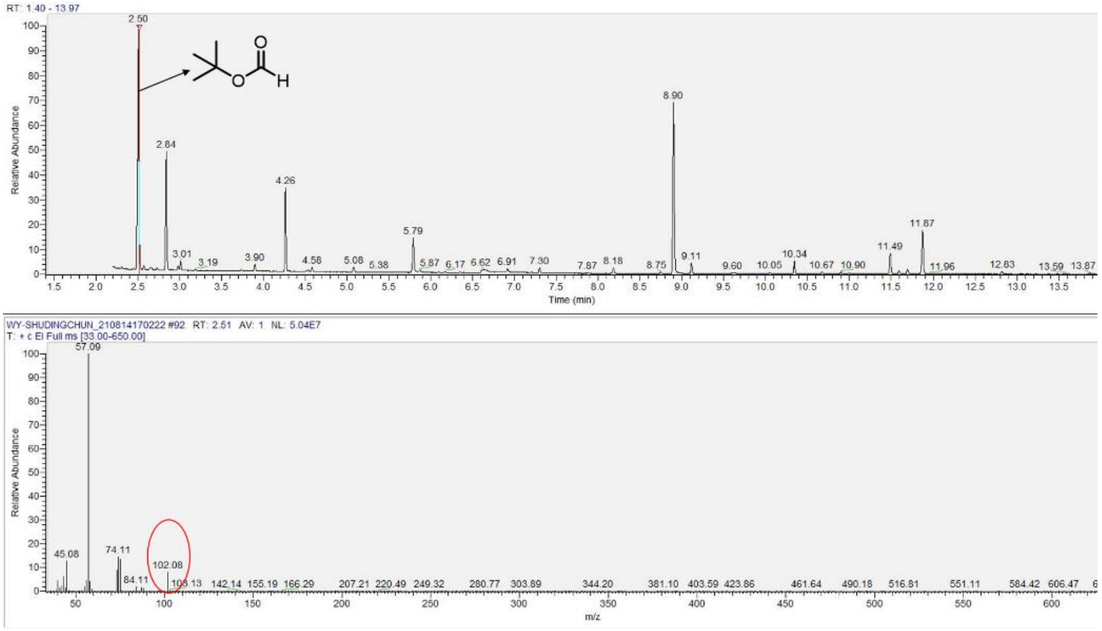

Supplementary Fig. 12 GC-MS analysis of the model reaction by replacing MeOH with *t*-BuOH as the solvent.

(6) Computational Details

All calculations were carried out using the Gaussian 16 C.01 program package.[3] The geometry optimizations were performed using hybrid B3LYP exchange correlation[4-6]. The LanL2DZ basis set pseudopotential[7] was used for Ru atom. The 6-311G(d, p) basis set[8-10] was used for remaining atoms. Vibrational frequency calculations were performed to characterize the nature of each stationary point and to make the zero-point energy (ZPE) corrections. The synchronous transit-guided quasi-Newton (QSTN) method, implemented with the Gaussian keyword QST3[11] was performed to located the transition states. The minimum energy path (MEP) was constructed by intrinsic reaction coordinate (IRC) theory for each reaction channel[12]. A tight convergence (10^{-12} au) criterion was employed, and the solvent CH_3OH was considered using the IEF-PCM solvent model[13].

Potential energy surface for the formation of [Ru^{II}HCl] employing different bases:

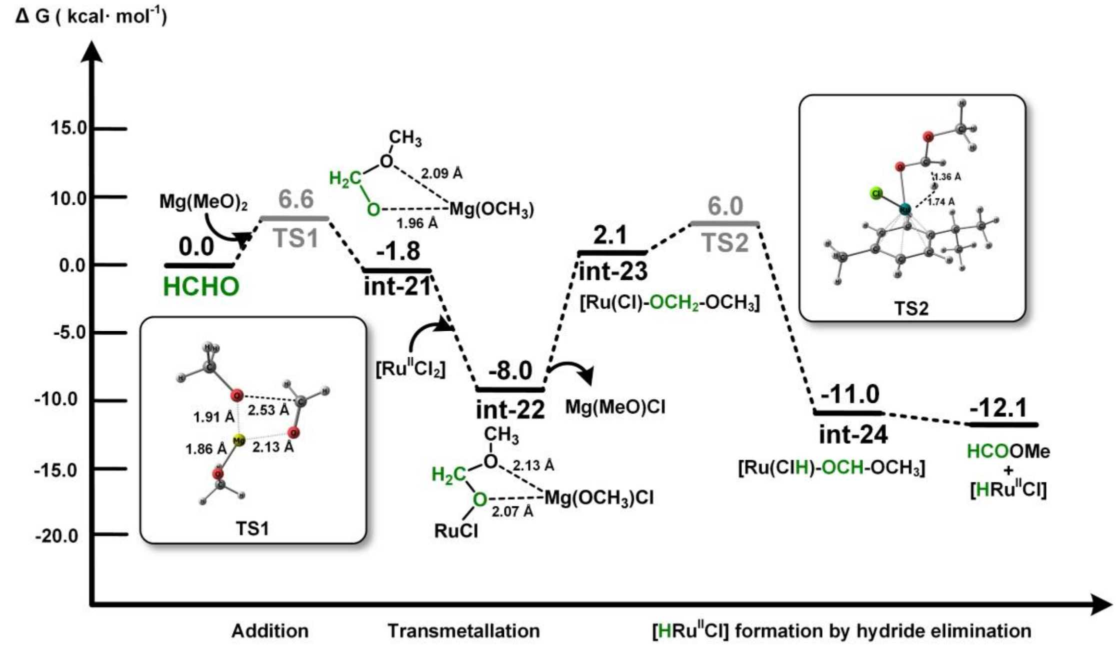

Supplementary Fig. 13 Potential energy surface for the formation of [Ru^{II}HCl] employing $Mg(OMe)_2$ as the base (free energies in kcal·mol^{-1}). The process from int-22 to int-23 is referred to the transmetallation step.

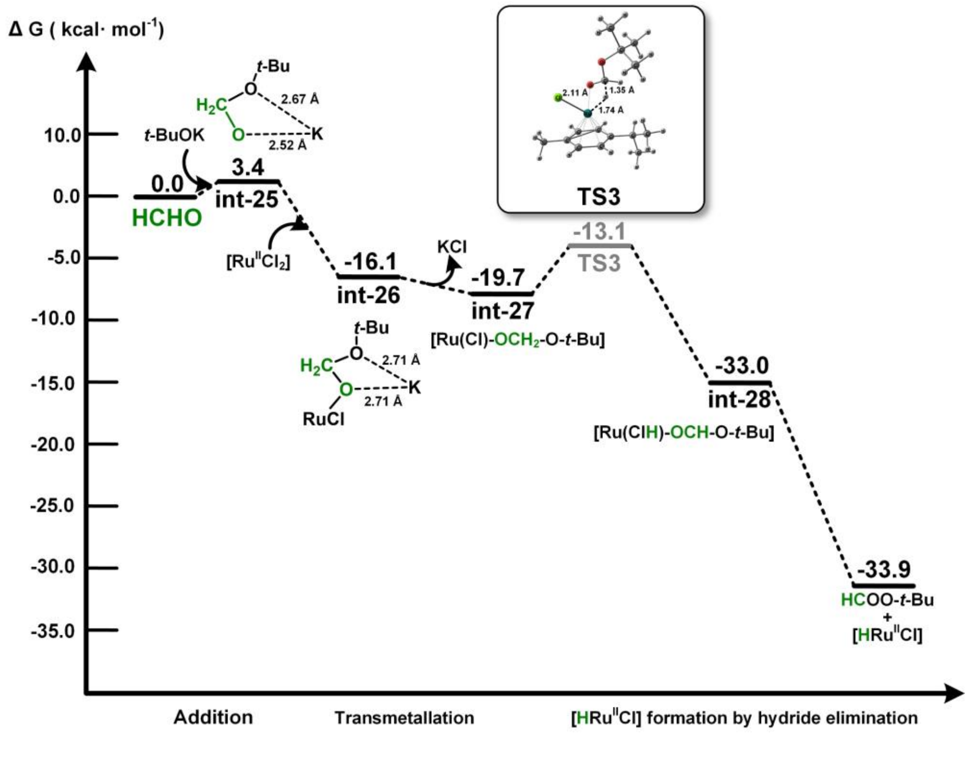

Supplementary Fig. 14 Potential energy surface for the formation of [Ru^{II}HCl] employing *t*-BuOK as the base (free energies in kcal·mol^{-1}). The process from int-26 to int-27 is referred to the transmetallation step.

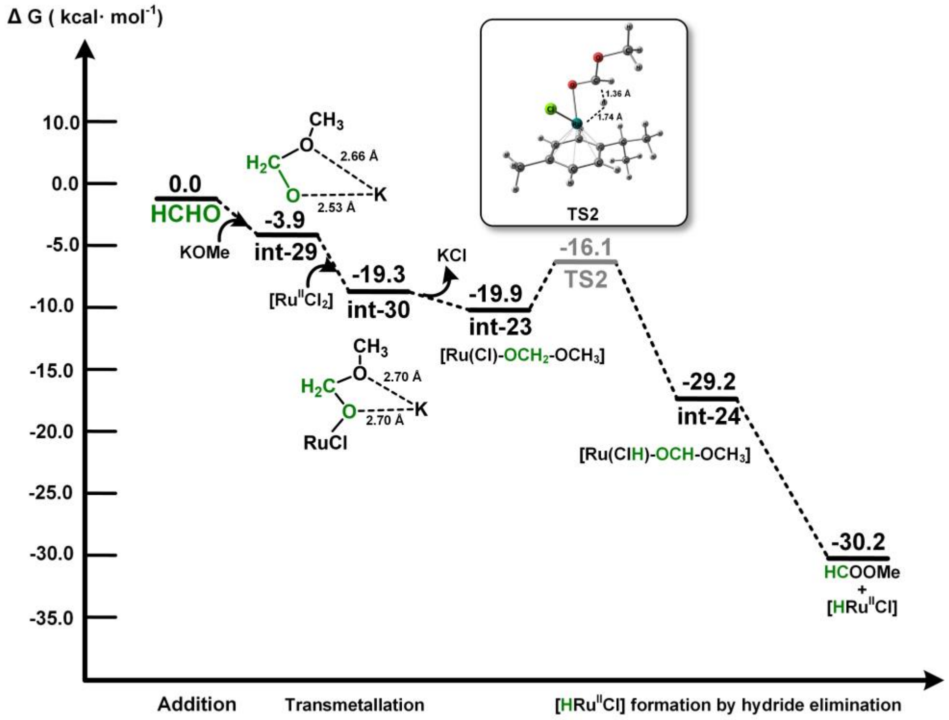

Supplementary Fig. 15 Potential energy surface for the formation of [Ru^{II}HCl] employing KOMe as the base (free energies in kcal·mol^{-1}). The process from int-29 to int-30 is referred to the transmetallation step.

To understand the origin of generating different products **P1** and **P51**, the formation of *β*-methylated product **P1'** from **int-2** has also been investigated by means of the same DFT calculations. Similar to the processes for the formation product **P51** (see Fig. 8b in the manuscript), the calculated highest barrier for **P1'** generation is 21.4 kcal·mol^{-1} (Supplementary Fig.16), which is corresponding to transition state **TS19** (**int-16 → int-17**), higher than any barrier of the favorable pathway toward the generation of product **P1** (Fig. 8a, **int-2 → int-3a → int-4 → P1**).

As for the formation of product **P51'** (Supplementary Fig. 17), the reaction pathway is similar with the generation of compound **P1** (see Fig. 8a in the manuscript). Herein, we also calculated the most favorable pathway: the addition of **int-6** to **B1'**, C1-C5 bond formation, and H^{+}-release and **P51'** formation. As shown in Fig. S14, despite the absence of a methyl unit in **int-19** (*vs.* **int-10a**), the associated barrier is still significant ($\Delta G^{\neq}$ = 18.3 kcal·mol^{-1}, **TS22**), higher than any transition states calculated in Fig. 8b.

Based on the above-described calculation results, it supports that the generation of product **P1'** and **P51'** is disfavored under the same reaction conditions. All in one, the enamine intermediates arising from hydride transfer to the azaarenium salts play a critical role in determining the product structures.

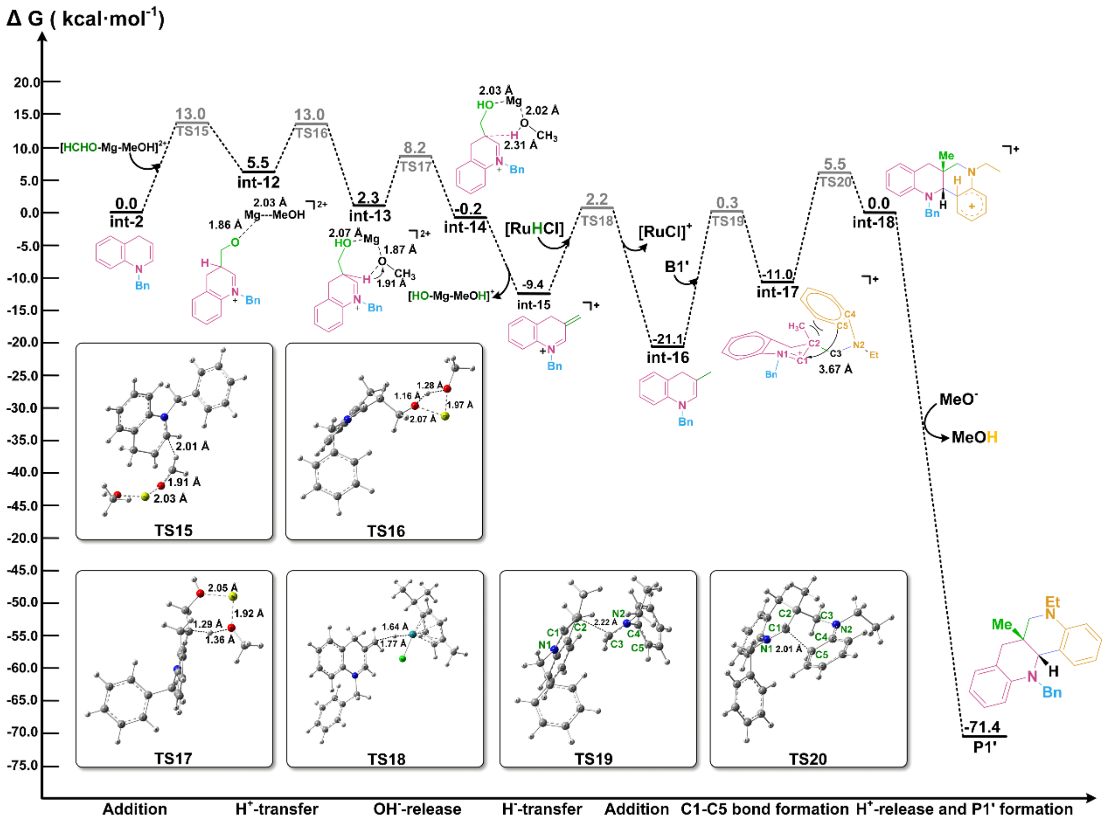

Supplementary Fig. 16 Potential energy surface for the process of int-2 to P1' (free energies in kcal·mol^{-1}).

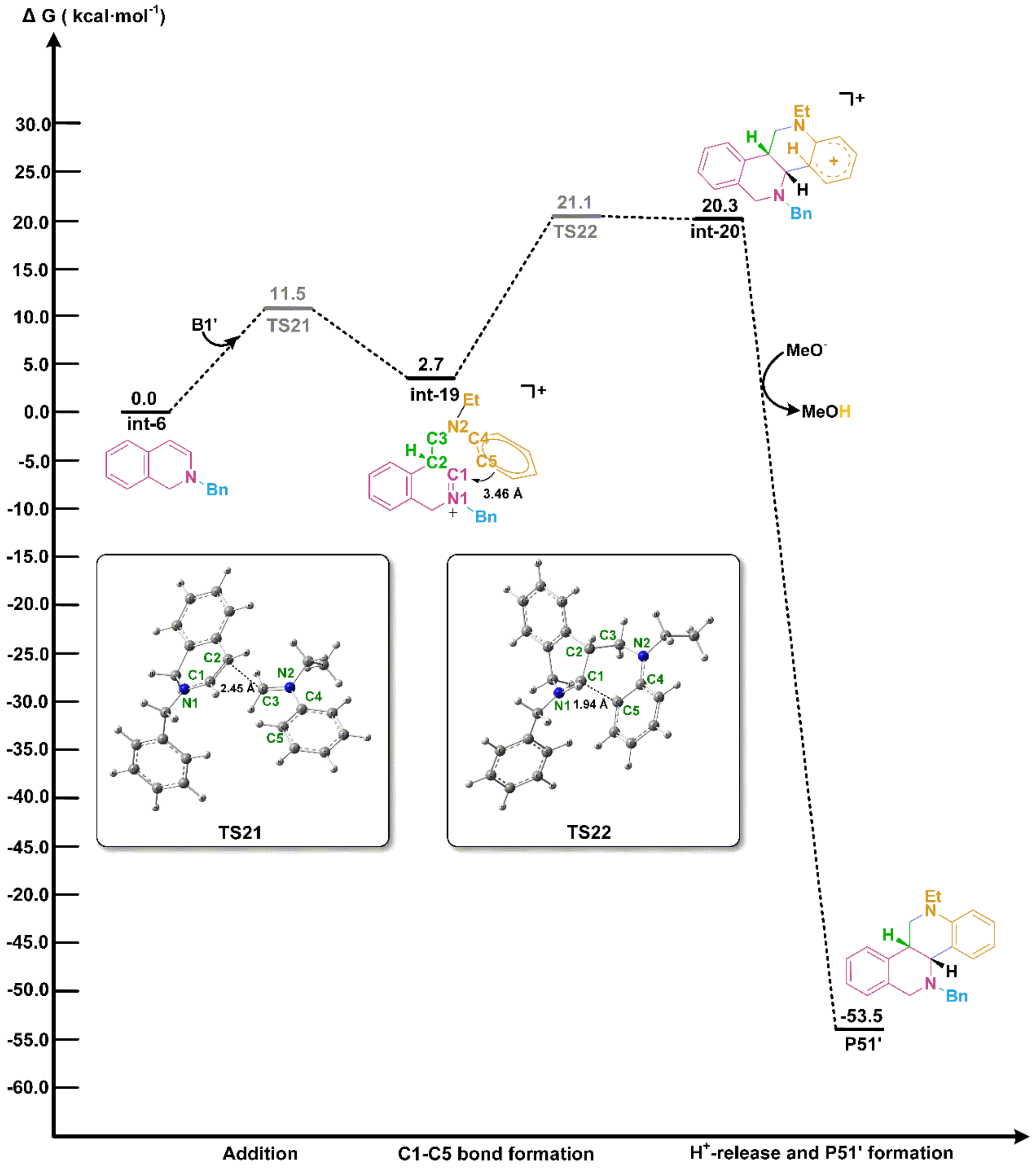

Supplementary Fig. 17 Potential energy surface for the process of int-6 to P51' (free energies in kcal·mol-1).

Analytic data of the obtained compounds

(1) (6a-*cis*)-12-benzyl-5-ethyl-5,6,6a,7,12,12a-hexahydrodibenzo[*b,h*][1,6]naphthyridine (**P1**)

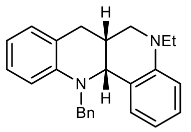

Brown solid; m.p. : 110-112 ºC, (64.4 mg, 91% yield); ^{1}H NMR (500 MHz, $CDCl_3$) δ 7.28 – 7.13 (m, 5H), 7.05 (t, *J* = 6.9 Hz, 1H), 6.99 – 6.88 (m, 3H), 6.60 – 6.53 (m, 3H), 6.49 (t, *J* = 7.3 Hz, 1H), 4.92 (d, *J* = 17.2 Hz, 1H), 4.50 (d, *J* = 3.6 Hz, 1H), 4.42 (d, *J* = 17.2 Hz, 1H), 3.47 (dd, *J* = 11.6, 5.0 Hz, 1H), 3.33 (q, *J* = 7.2 Hz, 2H), 3.26 (dd, *J* = 11.6, 6.2 Hz, 1H), 2.86 (dd, *J* = 16.4, 5.4 Hz, 1H), 2.68 (dd, *J* = 16.3, 8.4 Hz, 1H), 2.61 – 2.49 (m, 1H), 1.15 (t, *J* = 5.0 Hz, 3H). ^{13}C NMR (126 MHz, $CDCl_3$) δ 143.8, 143.7, 139.5, 129.6, 128.8, 128.6, 128.5, 127.2, 126.6, 126.6, 121.9, 120.8, 116.4, 115.2, 112.3, 110.2, 58.5, 53.8, 50.5, 44.7, 30.0, 29.7, 11.1. HRMS (ESI): Calcd. for $C_{25}H_{27}N_2$ $[M+H]^+$: 355.2169; found: 355.2172.

(2) (6a-*cis*)-12-benzyl-5-ethyl-7-methyl-5,6,6a,7,12,12a-hexahydrodibenzo[*b,h*][1,6]naphthyridine (**P2**)

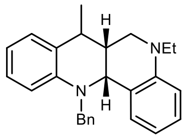

Yellow solid; m.p. : 146-148 ºC, (52.3 mg, 71% yield); ^{1}H NMR (500 MHz, $CDCl_3$) δ 7.21 – 7.16 (m, 2H), 7.15 – 7.12 (m, 1H), 7.11 – 7.06 (m, 4H), 7.01 – 6.93 (m, 1H), 6.91 (dd, *J* = 7.4, 1.8 Hz, 1H), 6.64 (td, *J* = 7.3, 1.2 Hz, 1H), 6.56 – 6.51 (m, 2H), 6.44 (td, *J* = 7.3, 1.2 Hz, 1H), 4.75 (d, *J* = 17.4 Hz, 1H), 4.50 (d, *J* = 2.8 Hz, 1H), 4.26 (d, *J* = 17.4 Hz, 1H), 3.42 – 3.26 (m, 3H), 3.15 (dd, *J* = 11.3, 5.2 Hz, 1H), 2.79 (dd, *J* = 7.2, 3.0 Hz, 1H), 2.24 – 2.16 (m, 1H), 1.41 (d, *J* = 7.2 Hz, 3H), 1.14 (t, *J* = 5.0 Hz, 3H).^{13}C NMR (126 MHz, $CDCl_3$) δ 144.4, 143.3, 139.9, 131.2, 129.5, 129.4, 128.3, 127.3, 126.4, 126.2, 125.8, 120.8, 116.5, 114.4, 113.0, 110.3, 54.0, 51.1, 48.9, 44.6, 36.7, 33.5, 24.5, 11.2.HRMS (ESI): Calcd. for $C_{26}H_{29}N_2$ $[M+H]^+$: 369.2325; found: 369.2321.

(3) (6a-*cis*)-12-benzyl-5-ethyl-9-methyl-5,6,6a,7,12,12a-hexahydrodibenzo[*b,h*][1,6]naphthyridine (**P3**)

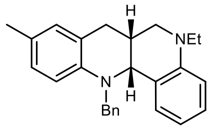

Yellow solid; m.p. : 113-115 ºC, (68.4 mg, 93% yield); ^{1}H NMR (500 MHz, $CDCl_3$) δ 7.19 – 7.11 (m, 4H), 7.09 – 7.03 (m, 1H), 6.97 (t, *J* = 7.8 Hz, 1H), 6.87 (d, *J* = 7.4 Hz, 1H), 6.69 (s, 1H), 6.68 (s, 1H), 6.46 (d, *J* = 8.2 Hz, 1H), 6.42 – 6.36 (m, 2H), 4.79 (d, *J* = 17.0 Hz, 1H), 4.38 (d, *J* = 3.4 Hz, 1H), 4.31 (d, *J* = 17.0 Hz, 1H), 3.37 (dd, *J* = 11.6, 5.0 Hz, 1H), 3.21-3.24 (m, 2H), 3.19 (dd, *J* = 11.4, 6.4 Hz, 1H), 2.77 (dd, *J* = 16.4, 5.4 Hz, 1H), 2.57 (dd, *J* = 16.4, 8.2 Hz, 1H), 2.49 – 2.40 (m, 1H), 2.08 (s, 3H), 1.06 (t, *J* = 5.0 Hz, 3H). ^{13}C NMR (126 MHz, $CDCl_3$) δ 142.7, 140.3, 138.7, 129.2, 127.8, 127.5, 127.3, 126.6, 125.5, 125.4, 124.3, 120.9, 119.7, 114.0, 111.4, 109.1, 57.3, 52.8, 49.4, 43.6, 28.9, 28.5, 19.2, 10.0. HRMS (ESI): Calcd. for $C_{26}H_{29}N_2$ $[M+H]^+$: 369.2325; found: 369.2320.

(4) (6a-*cis*)-12-benzyl-5-ethyl-9-methoxy-5,6,6a,7,12,12a-hexahydrodibenzo[*b,h*][1,6] naphthyridine (**P4**)

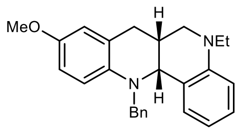

Brown solid; m.p. : 129-131 ºC, (49.1 mg, 64% yield); ^{1}H NMR (500 MHz, $CDCl_3$) δ 7.24 (d, *J* = 5.4 Hz, 4H), 7.17 (q, *J* = 5.6, 4.6 Hz, 1H), 7.06 (t, 1H), 6.99 (d, *J* = 7.4 Hz, 1H), 6.59 – 6.51 (m, 4H), 6.48 (t, *J* = 7.4 Hz, 1H), 4.82 (d, *J* = 17.0 Hz, 1H), 4.42 (d, *J* = 3.4 Hz, 1H), 4.38 (d, *J* = 16.8 Hz, 1H), 3.67 (s, 3H), 3.46 (dd, *J* = 11.4, 5.0 Hz, 1H), 3.34 (q, *J* = 7.0, 3.6 Hz, 2H), 3.29 (dd, *J* = 11.6, 6.2 Hz, 1H), 2.87 (dd, *J* = 16.6, 5.6 Hz, 1H), 2.67 (dd, *J* = 16.6, 8.2 Hz, 1H), 2.57 – 2.49 (m, 1H), 1.15 (t, *J* = 5.0 Hz, 3H). ^{13}C NMR (126 MHz, $CDCl_3$) δ 151.2, 143.8, 140.0, 138.1, 128.9, 128.5, 128.4, 126.7, 126.6, 122.5, 121.9, 115.3, 115.1, 114.1, 112.6, 110.1, 58.2, 55.6, 54.7, 50.5, 44.7, 29.7, 29.6, 11.1. HRMS (ESI): Calcd. for $C_{26}H_{29}N_2O$ $[M+H]^+$: 385.2274; found: 385.2269.

(5) (6a-*cis*)-12-benzyl-5-ethyl-9-(phenylthio)-5,6,6a,7,12,12a-hexahydrodibenzo[*b,h*][1,6] naphthyridine (**P5**)

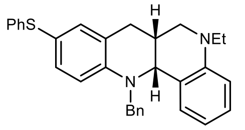

Brown solid; m.p. : 130-132 ºC, (62.8 mg, 68% yield); ^{1}H NMR (500 MHz, $CDCl_3$) δ 7.22 – 7.15 (m, 2H), 7.14 – 7.05 (m, 5H), 7.05 – 6.93 (m, 6H), 6.87 (d, *J* = 7.6 Hz, 1H), 6.51 – 6.41 (m, 3H), 4.87 (d, *J* = 17.2 Hz, 1H), 4.46 (d, *J* = 3.4 Hz, 1H), 4.38 (d, *J* = 17.2 Hz, 1H), 3.42 (dd, *J* = 11.8, 5.0 Hz, 1H), 3.25 (q, *J* = 7.2 Hz, 2H), 3.14 (dd, *J* = 11.6, 5.8 Hz, 1H), 2.72 (dd, *J* = 16.4, 5.2 Hz, 1H), 2.63 – 2.53 (m, 1H), 2.52 – 2.33 (m, 1H), 1.07 (t, *J* = 5.0 Hz, 3H). ^{13}C NMR (126 MHz, $CDCl_3$) δ 143.0, 142.5, 138.9, 137.7, 134.9, 132.9, 127.7, 127.5, 127.5, 126.1, 125.8, 125.4, 124.0, 120.9, 120.3, 116.3, 114.1, 111.8, 109.2, 57.6, 52.8, 49.3, 43.6, 28.7, 28.4, 10.0. HRMS (ESI): Calcd. for $C_{31}H_{31}N_2S$ $[M+H]^+$: 463.2202; found: 463.2193.

(6) N-((6a-*cis*)-12-benzyl-5-ethyl-5,6,6a,7,12,12a-hexahydrodibenzo[b,h][1,6]naphthyridin-9-yl)-acetamide (**P6**)

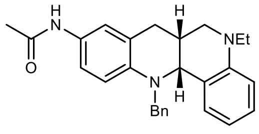

Orange solid; m.p. : 121-123 ºC, (74.8 mg, 91% yield); ^{1}H NMR (500 MHz, $CDCl_3$) δ 7.29 – 7.15 (m, 6H), 7.05 (t, *J* = 7.8 Hz, 1H), 6.92 (t, *J* = 8.6 Hz, 2H), 6.54 (d, *J* = 8.2 Hz, 1H), 6.50 – 6.44 (m, 2H), 4.87 (d, *J* = 17.0 Hz, 1H), 4.46 (d, *J* = 3.4 Hz, 1H), 4.39 (d, *J* = 17.0 Hz, 1H), 3.44 (dd, *J* = 11.6, 5.0 Hz, 1H), 3.32 (q, *J* = 7.2 Hz, 2H), 3.23 (dd, *J* = 11.6, 6.2 Hz, 1H), 2.81 (dd, *J* = 16.6, 5.6 Hz, 1H), 2.64 (dd, *J* = 16.6, 8.4 Hz, 1H), 2.53 – 2.47 (m, 1H), 2.02 (s, 3H), 1.14 (t, *J* = 5.0 Hz, 3H). ^{13}C NMR (126 MHz, $CDCl_3$) δ 168.3, 143.7, 140.9, 139.4, 128.8, 128.6, 128.5, 127.2, 126.7, 126.6, 122.8, 121.7, 121.3, 120.3, 115.1, 112.5, 110.2, 58.4, 54.0, 50.4, 44.6, 29.8, 29.6, 24.2, 11.0. HRMS (ESI): Calcd. for $C_{27}H_{30}N_3O$ $[M+H]^+$: 412.2383; found: 412.2375.

(7) (6a-*cis*)-12-benzyl-5-ethyl-10-methyl-5,6,6a,7,12,12a-hexahydrodibenzo[*b,h*][1,6]naphthyrid-ine (**P7**)

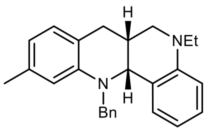

Yellow solid; m.p. : 111-113 ºC, (59.6 mg, 81% yield); ^{1}H NMR (500 MHz, $CDCl_3$) δ 7.22 – 7.06 (m, 5H), 6.97 (t, *J* = 7.8 Hz, 1H), 6.86 (d, *J* = 7.6 Hz, 1H), 6.75 (d, *J* = 7.4 Hz, 1H), 6.45 (d, *J* = 8.4 Hz, 1H), 6.40 (t, *J* = 7.5 Hz, 1H), 6.37 – 6.31 (m, 2H), 4.84 (d, *J* = 17.2 Hz, 1H), 4.38 (d, *J* = 3.2 Hz, 1H), 4.33 (d, *J* = 17.0 Hz, 1H), 3.37 (dd, *J* = 11.6, 5.0 Hz, 1H), 3.24 (q, *J* = 7.4 Hz, 2H), 3.17 (dd, *J* = 11.6, 6.0 Hz, 1H), 2.74 (dd, *J* = 16.4, 5.2 Hz, 1H), 2.55 (dd, *J* = 16.4, 8.4 Hz, 1H), 2.47 – 2.37 (m, 1H), 2.08 (s, 3H), 1.06 (t, *J* = 5.0 Hz, 3H). ^{13}C NMR (126 MHz, $CDCl_3$) δ 142.7, 142.5, 138.5, 135.6, 128.4, 127.7, 127.4, 127.3, 125.5, 125.5, 120.8, 116.8, 116.2, 114.0, 111.8, 109.0, 57.2, 52.6, 49.4, 43.5, 29.0, 28.3, 20.6, 10.0. HRMS (ESI): Calcd. for $C_{26}H_{29}N_2$ $[M+H]^+$: 369.2325; found: 369.2315.

(8) (6a-*cis*)-12-benzyl-8-bromo-5-ethyl-5,6,6a,7,12,12a-hexahydrodibenzo[*b,h*][1,6] naphthyridi-ne (**P8**)

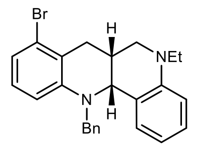

Brown solid; m.p. : 125-127 ºC, (34.64 mg, 40% yield); ^{1}H NMR (400 MHz, $CDCl_3$) δ 7.40 – 7.26 (m, 5H), 7.20 – 7.09 (m, 1H), 7.01 (d, *J* = 7.4 Hz, 1H), 6.95 – 6.85 (m, 2H), 6.69 – 6.55 (m, 3H), 5.03 (d, *J* = 17.2 Hz, 1H), 4.60 (d, *J* = 3.4 Hz, 1H), 4.55 (d, *J* = 17.2 Hz, 1H), 3.62 (dd, *J* = 11.6, 4.8 Hz, 1H), 3.44 (q, *J* = 7.2 Hz, 2H), 3.36 (dd, *J* = 11.8, 5.4 Hz, 1H), 3.03 (dd, *J* = 16.8, 5.4 Hz, 1H), 2.72 (dd, *J* = 16.8, 9.0 Hz, 1H), 2.68 – 2.58 (m, 1H), 1.26 (t, *J* = 5.0 Hz, 3H). ^{13}C NMR (126 MHz, $CDCl_3$) δ 144.0, 142.6, 137.8, 127.7, 127.5, 127.3, 126.8, 125.8, 125.4, 125.0, 120.2, 119.4, 114.2, 110.3, 109.3, 57.3, 53.5, 49.5, 43.6, 29.3, 28.8, 10.0. HRMS (ESI): Calcd. for $C_{25}H_{26}BrN_2$ $[M+H]^+$: 433.1274; found: 433.1268.

(9) (6a-*cis*)-12-benzyl-9-bromo-5-ethyl-5,6,6a,7,12,12a-hexahydrodibenzo[*b,h*][1,6]naphthyridine (**P9**)

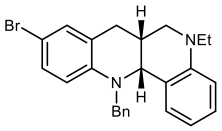

Brown solid; m.p. : 139-141 ºC, (38.1 mg, 44% yield); ^{1}H NMR (500 MHz, $CDCl_3$) δ 7.28 – 7.22 (m, 2H), 7.21 – 7.16 (m, 3H), 7.10 – 7.05 (m, 1H), 7.04 – 6.98 (m, 2H), 6.92 (dd, *J* = 7.5, 1.6 Hz, 1H), 6.56 (d, *J* = 8.2 Hz, 1H), 6.50 (t, *J* = 7.4 Hz, 1H), 6.42 (d, *J* = 8.6 Hz, 1H), 4.89 (d, *J* = 17.2 Hz, 1H), 4.50 (d, *J* = 3.6 Hz, 1H), 4.42 (d, *J* = 17.2 Hz, 1H), 3.50 (dd, *J* = 11.2, 5.0 Hz, 1H), 3.33 (q, *J* = 7.0 Hz, 2H), 3.23 (dd, *J* = 11.6, 5.8 Hz, 1H), 2.81 (dd, *J* = 16.6, 5.4 Hz, 1H), 2.66 (dd, *J* = 16.6, 8.8 Hz, 1H), 2.57 – 2.50 (m, 1H), 1.16 (t, *J* = 5.0, 3H). ^{13}C NMR (126 MHz, $CDCl_3$) δ 143.6, 142.6, 138.8, 131.9, 129.8, 128.7, 128.6, 128.5, 126.8, 126.4, 123.1, 121.3, 115.2, 113.8, 110.3,

108.1, 58.5, 54.0, 50.3, 44.6, 29.7, 29.4, 11.0. HRMS (ESI): Calcd. for $C_{25}H_{26}BrN_2$ $[M+H]^+$: 433.1274; found: 433.1269.

(10) (6a-*cis*)-12-benzyl-9-chloro-5-ethyl-5,6,6a,7,12,12a-hexahydrodibenzo[*b,h*][1,6]naphthyridine (**P10**)

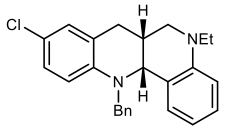

Yellow solid; m.p. : 138-140 ºC, (31.0 mg, 40% yield); ^{1}H NMR (500 MHz, $CDCl_3$) δ 7.22 – 7.16 (m, 2H), 7.14 – 7.09 (m, 3H), 7.00 – 6.89 (m, 1H), 6.87 – 6.79 (m, 3H), 6.51 – 6.47 (m, 1H), 6.46 – 6.35 (m, 2H), 4.83 (d, *J* = 17.2 Hz, 1H), 4.43 (d, *J* = 3.6 Hz, 1H), 4.36 (d, *J* = 17.2 Hz, 1H), 3.43 (dd, *J* = 11.6, 5.0 Hz, 1H), 3.27 (q, *J* = 7.0 Hz, 2H), 3.16 (dd, *J* = 11.6, 5.8 Hz, 1H), 2.74 (dd, *J* = 16.6, 5.4 Hz, 1H), 2.59 (dd, *J* = 16.6, 8.8 Hz, 1H), 2.51 – 2.38 (m, 1H), 1.08 (t, *J* = 5.0 Hz, 3H). ^{13}C NMR (126 MHz, $CDCl_3$) δ 143.6, 142.1, 138.9, 129.1, 128.7, 128.6, 126.9, 126.8, 126.5, 122.6, 121.4, 120.9, 115.2, 113.4, 110.3, 58.5, 54.1, 50.4, 44.6, 29.7, 29.4, 11.0. HRMS (ESI): Calcd. for $C_{25}H_{26}ClN_2$ $[M+H]^+$: 389.1779; found: 389.1772.

(11) (6a-*cis*)-12-benzyl-5-ethyl-9-fluoro-5,6,6a,7,12,12a-hexahydrodibenzo[*b,h*][1,6]naphthyridine (**P11**)

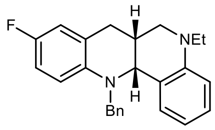

Brown solid; m.p. : 81-83 ºC, (46.2 mg, 62% yield); ^{1}H NMR (500 MHz, $CDCl_3$) δ 7.32 – 7.16 (m, 5H), 7.07 (t, *J* = 8.0 Hz, 1H), 6.96 (d, *J* = 7.6 Hz, 1H), 6.66 (d, *J* = 8.6 Hz, 2H), 6.55 (d, *J* = 8.4 Hz, 1H), 6.52 – 6.41 (m, 2H), 4.87 (d, *J* = 17.0 Hz, 1H), 4.47 (d, *J* = 3.2 Hz, 1H), 4.42 (d, *J* = 17.0 Hz, 1H), 3.50 (dd, *J* = 11.8, 4.8 Hz, 1H), 3.35 (q, *J* = 7.2 Hz, 2H), 3.26 (dd, *J* = 11.6, 5.8 Hz, 1H), 2.83 (dd, *J* = 16.8, 5.4 Hz, 1H), 2.67 (dd, *J* = 16.8, 8.6 Hz, 1H), 2.59 – 2.49 (m, 1H), 1.11 (t, *J* = 5.0 Hz, 3H). ^{13}C NMR (126 MHz, $CDCl_3$) δ 156.0, 154.2, 143.7, 140.0, 139.4, 128.7, 128.5, 127.7 (d, *J* = 237.0 Hz), 126.6, 122.5 (d, *J* = 6.4 Hz), 121.6, 115.8 (d, *J* = 22.68 Hz), 115.2, 113.4 (d, *J* = 21.42 Hz), 113.4 (d, *J* = 7.56 Hz), 110.2, 58.4, 54.7, 50.5, 44.7, 29.6, 29.5, 11.1. ^{19}F NMR (471 MHz, $CDCl_3$) δ -129.45(s). HRMS (ESI): Calcd. for $C_{25}H_{26}FN_2$ $[M+H]^+$: 373.2075; found: 373.2068.

(12) methyl((6a-*cis*)-12-benzyl-5-ethyl-5,6,6a,7,12,12a-hexahydrodibenzo[*b,h*][1,6]naphthyridine)-9-carboxylate (**P12**)

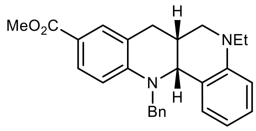

Yellow solid; m.p. : 151-153 ºC, (49.4 mg, 60% yield); ^{1}H NMR (500 MHz, $CDCl_3$) δ 7.66 (s, 1H), 7.64 (s, 1H), 7.30 – 7.14 (m, 5H), 7.08 (t, *J* = 7.8 Hz, 1H), 6.91 (d, *J* = 7.6 Hz, 1H), 6.56 (d, *J* = 9.4 Hz, 2H), 6.53 – 6.50 (m, 1H), 5.02 (d, *J* = 17.2 Hz, 1H), 4.58 (d, *J* = 4.6 Hz, 1H), 4.51 (d, *J* = 17.2 Hz, 1H), 3.80 (s, 3H), 3.52 (dd, *J* = 12.0, 5.0 Hz, 1H), 3.34 – 3.30 (m, 2H), 3.22 (dd, *J* = 11.8, 5.8 Hz, 1H), 2.87 (dd, *J* = 16.4, 5.2 Hz, 1H), 2.72 (dd, *J* = 16.4, 9.0 Hz, 1H), 2.60 – 2.51 (m, 1H), 1.16

(t, J = 5.0 Hz, 3H). ^{13}C NMR (126 MHz, $CDCl_3$) δ 167.4, 147.5, 143.6, 138.1, 131.3, 129.5, 128.9, 128.7, 128.4, 127.0, 126.4, 121.0, 120.1, 117.4, 115.3, 111.0, 110.4, 58.8, 53.7, 51.5, 50.2, 44.6, 29.7, 29.5, 11.0. HRMS (ESI): Calcd. for $C_{27}H_{29}N_2O_2$ $[M+H]^+$: 413.2224; found: 413.2216.

(13) (6a-*cis*)-5-ethyl-12-(3-methoxybenzyl)-5,6,6a,7,12,12a-hexahydrodibenzo[*b,h*][1,6]naphthyridine (**P13**)

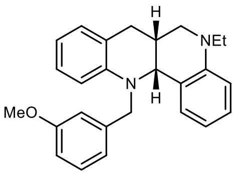

Black solid; m.p. : 79-81 ºC, (72.3 mg, 94% yield); ^1H NMR (500 MHz, $CDCl_3$) δ 7.19 – 7.12 (m, 1H), 7.06 (t, J = 7.8 Hz, 1H), 6.98 – 6.88 (m, 3H), 6.81 (d, J = 7.6 Hz, 1H), 6.76 (s, 1H), 6.71 (d, J = 8.2 Hz, 1H), 6.59 – 6.52 (m, 3H), 6.49 (t, J = 7.3 Hz, 1H), 4.88 (d, J = 17.2 Hz, 1H), 4.49 (d, J = 3.4 Hz, 1H), 4.40 (d, J = 17.2 Hz, 1H), 3.71 (s, 3H), 3.47 (dd, J = 11.6, 5.0 Hz, 1H), 3.32 (q, J = 7.6 Hz, 2H), 3.25 (dd, J = 11.6, 6.2 Hz, 1H), 2.85 (dd, J = 16.4, 5.4 Hz, 1H), 2.67 (dd, J = 16.4, 8.4 Hz, 1H), 2.58 – 2.49 (m, 1H), 1.15 (t, J = 5.0 Hz, 3H). ^{13}C NMR (126 MHz, $CDCl_3$) δ 159.9, 143.8, 143.7, 141.4, 129.6, 129.5, 128.8, 128.6, 127.2, 121.9, 120.9, 118.9, 116.44, 115.2, 112.4, 112.4, 111.8, 110.2, 58.5, 55.2, 53.9, 50.5, 44.7, 30.0, 29.7, 11.1. HRMS (ESI): Calcd. for $C_{26}H_{29}N_2O$ $[M+H]^+$: 385.2274; found: 385.2266.

(14) (6a-*cis*)-5-ethyl-9-methyl-12-(4-(trifluoromethyl)benzyl)-5,6,6a,7,12,12a-hexahydrodibenzo [*b,h*][1,6]naphthyridine (**P14**)

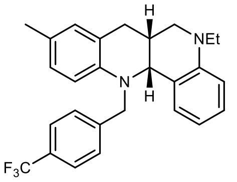

White solid; m.p. : 112-114 ºC, (83.7 mg, 96% yield); ^1H NMR (500 MHz, $CDCl_3$) δ 7.38 (d, J = 8.0 Hz, 2H), 7.19 (d, J = 8.0 Hz, 2H), 6.97 (m, 1H), 6.84 (d, J = 7.4 Hz, 1H), 6.76 – 6.66 (m, 2H), 6.46 (d, J = 8.4 Hz, 1H), 6.39 (t, J = 7.4 Hz, 1H), 6.29 (d, J = 8.2 Hz, 1H), 4.77 (d, J = 17.4 Hz, 1H), 4.35 (d, J = 3.0 Hz, 1H), 4.30 (d, J = 17.4 Hz, 1H), 3.36 – 3.19 (m, 4H), 2.88 (dd, J = 16.6, 5.6 Hz, 1H), 2.58 (dd, J = 16.6, 6.8 Hz, 1H), 2.49 – 2.43 (m, 1H), 2.09 (s, 3H), 1.07 (t, J = 5.0 Hz, 3H). ^{13}C NMR (126 MHz, $CDCl_3$) δ 144.5, 143.9, 141.3, 130.5, 129.4, 129.0, 128.8 (q, J = 32.76 Hz), 127.9, 126.8, 126.0, 125.4 (q, J = 3.78 Hz), 124.4 (q, J = 272.16 Hz), 121.5, 120.9, 115.0, 112.5, 110.3, 58.6, 53.1, 50.0, 44.6, 30.2, 29.8, 20.3, 11.1. ^{19}F NMR (471 MHz, $CDCl_3$) δ -62.19. HRMS (ESI): Calcd. for $C_{27}H_{28}F_3N_2$ $[M+H]^+$: 437.2199; found: 437.2194.

(15) (6a-*cis*)-5-ethyl-12-((perfluorophenyl)methyl)-5,6,6a,7,12,12a-hexahydrodibenzo[*b,h*][1,6] naphthyridine (**P15**)

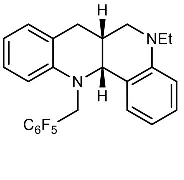

Black solid; m.p. : 56-58 ºC, (55.9 mg, 63% yield); ^{1}H NMR (500 MHz, $CDCl_3$) δ 7.04 (q, *J* = 7.5 Hz, 2H), 6.91 (t, *J* = 7.9 Hz, 2H), 6.79 (d, *J* = 8.3 Hz, 1H), 6.71 – 6.60 (m, 1H), 6.54 – 6.45 (m, 2H), 5.05 (d, *J* = 16.0 Hz, 1H), 4.59 (d, *J* = 3.8 Hz, 1H), 4.51 (d, *J* = 16.0 Hz, 1H), 3.65 (dd, *J* = 11.8, 4.4 Hz, 1H), 3.39 – 3.26 (m, 2H), 3.18 (dd, *J* = 11.8, 4.0 Hz, 1H), 2.74 – 2.60 (m, 2H), 2.53 – 2.44 (m, 1H), 1.16 (t, *J* = 5.0 Hz, 3H). ^{13}C NMR (126 MHz, $CDCl_3$) δ 146.5-144.5 (m), 143.5, 142.1, 141.4-136.4 (m), 129.9, 128.4, 127.6, 127.0, 122.5, 121.4, 117.7, 115.3, 112.9-112.7 (m), 112.4, 109.9, 59.2, 51.1, 45.0, 44.7, 28.5, 28.3, 10.9. ^{19}F NMR (471 MHz, $CDCl_3$) δ -142.4 (dd, *J* = 23.6, 8.0 Hz), -155.5 (t, *J* = 20.9 Hz), -162.1 (td, *J* = 22.2, 8.4 Hz). HRMS (ESI): Calcd. for $C_{25}H_{22}F_5N_2$ $[M+H]^+$: 445.1698; found: 445.1688.

(16) (6a-*cis*)-5-ethyl-12-phenethyl-5,6,6a,7,12,12a-hexahydrodibenzo[*b*,*h*][1,6]naphthyridine (**P16**)

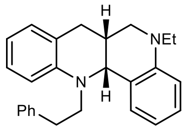

Black oil; (62.6 mg, 85% yield); ^{1}H NMR (500 MHz, $CDCl_3$) δ 7.22 – 7.15 (m, 2H), 7.12 – 7.07 (m, 1H), 7.06 – 6.97 (m, 4H), 6.90 (d, *J* = 7.6 Hz, 1H), 6.84 (d, *J* = 7.4 Hz, 1H), 6.71 (d, *J* = 8.4 Hz, 1H), 6.54 – 6.41 (m, 3H), 4.16 (d, *J* = 3.6 Hz, 1H), 3.92 – 3.80 (m, 1H), 3.34 (dd, *J* = 15.6, 8.0 Hz, 1H), 3.28 (dd, *J* = 12.3, 5.6 Hz, 1H), 3.22 (q, *J* = 7.2 Hz, 2H), 3.06 (dd, *J* = 11.6, 5.8 Hz, 1H), 2.85 – 2.76 (m, 1H), 2.71 – 2.61 (m, 2H), 2.52 (dd, *J* = 16.3, 8.8 Hz, 1H), 2.23 – 2.16 (m, 1H), 1.04 (t, *J* = 5.0 Hz, 3H). ^{13}C NMR (126 MHz, $CDCl_3$) δ 143.6, 142.9, 139.8, 129.8, 129.0, 128.7, 128.6, 128.5, 127.3, 126.2, 122.1, 121.0, 115.8, 115.2, 111.1, 110.1, 58.4, 52.3, 50.4, 44.7, 33.7, 29.6, 29.4, 11.0. HRMS (ESI): Calcd. for $C_{26}H_{29}N_2$ $[M+H]^+$: 369.2320; found: 369.2320.

(17) (6a-*cis*)-5-ethyl-12-(4-nitrophenethyl)-5,6,6a,7,12,12a-hexahydrodibenzo[*b*,*h*][1,6]naphthyridine (**P17**)

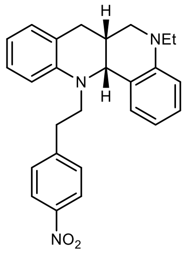

Brown solid; m.p. : 105-107 ºC, (67.7 mg, 82% yield); ^{1}H NMR (500 MHz, $CDCl_3$) δ 8.10 (d, *J* = 8.2 Hz, 2H), 7.25 – 7.17 (m, 2H), 7.12 (t, *J* = 8.1 Hz, 2H), 7.01 – 6.91 (m, 2H), 6.72 (d, *J* = 8.4 Hz, 1H), 6.62 (t, *J* = 7.6 Hz, 1H), 6.59 – 6.52 (m, 2H), 4.21 (d, *J* = 3.4 Hz, 1H), 3.90 (t, *J* = 12.4 Hz, 1H), 3.41 (dt, *J* = 15.8, 8.4 Hz, 1H), 3.35 – 3.26 (m, 3H), 3.17 (dd, *J* = 12.0, 7.0 Hz, 1H), 3.02 – 2.92 (m, 1H), 2.84 (dd, *J* = 16.6, 5.2 Hz, 1H), 2.78 – 2.69 (m, 1H), 2.61 (dd, *J* = 16.5, 7.8 Hz, 1H), 2.33 – 2.26 (m, 1H), 1.12 (t, *J* = 5.0 Hz, 3H). ^{13}C NMR (126 MHz, $CDCl_3$) δ 147.7, 146.6, 143.8, 142.7, 130.0, 129.8, 129.0, 129.0, 127.4, 123.7, 121.5, 121.0, 116.4, 115.0, 111.1, 110.3, 58.5, 51.4, 49.9, 44.6, 33.9, 29.7, 29.6, 11.0. HRMS (ESI): Calcd. for $C_{26}H_{28}N_3O_2$ $[M+H]^+$: 414.2176; found: 414.2167.

(18) (6a-*cis*)-5-ethyl-12-(3-methylbut-2-en-1-yl)-5,6,6a,7,12,12a-hexahydrodibenzo[*b*,*h*][1,6]naphthyridine (**P18**)

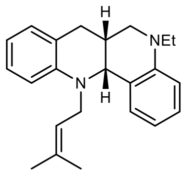

Black oil; (47.1 mg, 71% yield); ^{1}H NMR (500 MHz, $CDCl_3$) δ 7.04 – 6.90 (m, 3H), 6.83 (d, *J* = 7.4 Hz, 1H), 6.58 (d, *J* = 8.4 Hz, 1H), 6.54 – 6.40 (m, 3H), 5.20 – 5.10 (m, 1H), 4.32 (d, *J* = 3.6 Hz, 1H), 4.15 (dd, *J* = 16.8, 5.6 Hz, 1H), 3.74 (dd, *J* = 16.7, 6.4 Hz, 1H), 3.40 (dd, *J* = 11.6, 4.8 Hz, 1H), 3.25 (q, *J* = 7.2 Hz, 2H), 3.13 (dd, *J* = 11.5, 5.9 Hz, 1H), 2.70 (dd, *J* = 16.5, 5.5 Hz, 1H), 2.55 (dd, *J* = 16.5, 9.0 Hz, 1H), 2.34 – 2.29 (m, 1H), 1.61 (s, 3H), 1.56 (s, 3H), 1.07 (t, *J* = 7.0 Hz, 3H). ^{13}C NMR (126 MHz, $CDCl_3$) δ 142.6, 142.5, 132.9, 128.5, 128.0, 127.3, 126.0, 121.3, 120.7, 120.3, 114.9, 114.1, 111.1, 109.0, 56.4, 49.4, 47.1, 43.6, 28.6, 28.5, 24.7, 17.0, 9.9. HRMS (ESI): Calcd. for $C_{23}H_{29}N_2$ $[M+H]^+$: 333.2325; found: 333.2317.

(19) Benzyl 2-((6a-*cis*)-5-ethyl-5,6a,7,12a-tetrahydrodibenzo[b,h][1,6]naphthyridin-12(6H)-yl)a-cetate (**P19**)

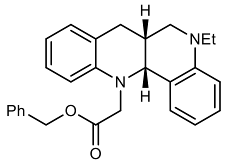

Yellow solid; m.p. : 67-69 ºC, (18.2 mg, 22% yield); ^{1}H NMR (500 MHz, $CDCl_3$) δ 7.34 – 7.29 (m, 3H), 7.28 – 7.22 (m, 2H), 7.12 (t, *J* = 7.8 Hz, 1H), 7.03 (t, *J* = 8.0 Hz, 1H), 6.99 (d, *J* = 7.4 Hz, 1H), 6.93 (d, *J* = 7.4 Hz, 1H), 6.90 – 6.72 (m, 1H), 6.59 (d, *J* = 8.4 Hz, 1H), 6.50 – 6.45 (m, 2H), 5.13 (s, 2H), 4.52 (d, *J* = 3.8 Hz, 1H), 4.20 (d, *J* = 18.4 Hz, 1H), 3.88 (d, *J* = 18.4 Hz, 1H), 3.33 – 3.25 (m, 3H), 3.21 (dd, *J* = 11.4, 5.2 Hz, 1H), 3.08 (dd, *J* = 16.6, 5.8 Hz, 1H), 2.65 (dd, *J* = 16.6, 4.8 Hz, 1H), 2.54 – 2.46 (m, 1H), 1.10 (t, *J* = 7.1 Hz, 3H). ^{13}C NMR (126 MHz, $CDCl_3$) δ 171.6, 144.0, 143.3, 135.9, 130.3, 129.8, 129.3, 128.6, 128.2, 128.2, 127.3, 121.1, 120.7, 117.4, 114.8, 111.5, 110.6, 66.5, 57.6, 49.6, 49.0, 44.7, 30.0, 29.8, 11.0. HRMS (ESI): Calcd. for $C_{27}H_{29}N_2O_2$ $[M+H]^+$: 413.2224; found: 413.2214.

(20) (6a-*cis*)-5-ethyl-12-hexyl-5,6,6a,7,12,12a-hexahydrodibenzo[b,h][1,6]naphthyridine (**P20**)

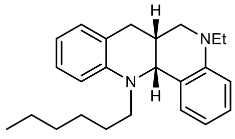

Black oil; (40.4 mg, 58% yield); ^{1}H NMR (500 MHz, $CDCl_3$) δ 7.04 – 6.95 (m, 2H), 6.90 (d, *J* = 7.6 Hz, 1H), 6.81 (d, *J* = 7.4 Hz, 1H), 6.61 (d, *J* = 8.4 Hz, 1H), 6.50 – 6.38 (m, 3H), 4.31 (d, *J* = 4.0 Hz, 1H), 3.69 – 3.54 (m, 1H), 3.48 (dd, *J* = 11.6, 4.6 Hz, 1H), 3.26 (p, *J* = 7.1 Hz, 2H), 3.13 – 3.04 (m, 2H), 2.63 (dd, *J* = 16.4, 5.2 Hz, 1H), 2.54 (dd, *J* = 16.4, 9.4 Hz, 1H), 2.36 – 2.27 (m, 1H), 1.57 – 1.45 (m, 2H), 1.24 – 1.15 (m, 6H), 1.08 (t, *J* = 7.1 Hz, 3H), 0.81 (t, *J* = 5.8 Hz, 3H). ^{13}C NMR (126 MHz, $CDCl_3$) δ 143.6, 143.3, 129.6, 128.4, 128.3, 127.1, 122.5, 121.0, 115.5, 115.3, 111.3, 110.1, 58.3, 51.0, 50.9, 44.7, 31.7, 29.5, 29.4, 27.1, 26.8, 22.7, 14.1, 11.0. HRMS (ESI): Calcd. for $C_{24}H_{33}N_2$ $[M+H]^+$: 349.2638; found: 349.2634.

(21) 1-((6a-*cis*)-5-ethyl-5,6a,7,12a-tetrahydrodibenzo[b,h][1,6]naphthyridin-12(6H)-yl)-5-((6a-*cis*)-5-ethyl-5,6a,7,12a-tetrahydrodibenzo[b,h][1,6]naphthyridin-12(6H)-yl)pentane (**P21**)

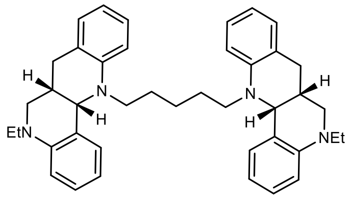

Brown solid, m.p. : 67-69 ºC (84.6 mg, 71% yield); ^{1}H NMR (500 MHz, $CDCl_3$) δ 7.05 (q, *J* = 7.2 Hz, 4H), 6.95 (d, *J* = 7.6 Hz, 2H), 6.88 (d, *J* = 7.4 Hz, 2H), 6.65 (d, *J* = 8.4 Hz, 2H), 6.58 – 6.45 (m, 6H), 4.34 (d, *J* = 3.4 Hz, 2H), 3.75 – 3.63 (m, 2H), 3.52 (td, *J* = 11.7, 4.6 Hz, 2H), 3.39 – 3.25 (m, 4H), 3.14 (dq, *J* = 14.6, 8.0, 7.2 Hz, 4H), 2.74 – 2.66 (m, 2H), 2.60 (dd, *J* = 16.4, 9.6 Hz, 2H), 2.43 – 2.30 (m, 2H), 1.66 – 1.53 (m, 4H), 1.35 – 1.23 (m, 2H), 1.20 – 1.08 (m, 6H). ^{13}C NMR (126 MHz, $CDCl_3$) δ 143.6, 143.2, 129.7, 128.3, 128.3, 127.1, 122.4, 121.0, 115.6, 115.2, 111.3, 110.1, 58.3, 50.8, 44.7, 29.5, 29.4, 29.4, 27.2, 24.7, 24.7, 11.0. HRMS (ESI): Calcd. for $C_{41}H_{49}N_4$ $[M+H]^+$: 597.3952; found: 597.3941.

(22) (6a-*cis*)-12-benzyl-2-(tert-butyl)-5-ethyl-5,6,6a,7,12,12ahexahydrodibenzo[*b*,*h*][1,6]naphthyridine (**P22**)

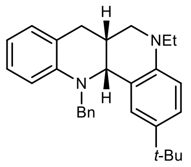

White solid; m.p. : 102-104 ºC, (59.9 mg, 73% yield); ^{1}H NMR (400 MHz, $CDCl_3$) δ 7.31 – 7.24 (m, 2H), 7.21 (d, *J* = 7.2 Hz, 3H), 7.12 (dd, *J* = 8.6, 2.4 Hz, 1H), 7.04 – 6.98 (m, 2H), 6.94 (d, *J* = 2.4 Hz, 1H), 6.67 – 6.58 (m, 2H), 6.54 (d, *J* = 8.6 Hz, 1H), 4.85 (d, *J* = 17.4 Hz, 1H), 4.56 (d, *J* = 3.2 Hz, 1H), 4.40 (d, *J* = 17.4 Hz, 1H), 3.42 – 3.27 (m, 4H), 3.07 (dd, *J* = 16.6, 5.6 Hz, 1H), 2.76 (dd, *J* = 16.6, 6.4 Hz, 1H), 2.63 – 2.53 (m, 1H), 1.21 – 1.14 (m, 12H). ^{13}C NMR (101 MHz, $CDCl_3$) δ 144.0, 141.6, 139.5, 137.3, 129.5, 128.3, 127.2, 126.7, 126.6, 126.4, 125.5, 120.9, 120.7, 116.3, 112.5, 109.8, 58.2, 52.8, 49.8, 44.7, 33.5, 31.5, 30.0, 11.2, 1.1. HRMS (ESI): Calcd. for $C_{29}H_{35}N_2$ $[M+H]^+$: 411.2795; found: 411.2787.

(23) (6a-*cis*)-12-benzyl-5-ethyl-1,3-dimethoxy-5,6,6a,7,12,12a-hexahydrodibenzo[*b*,*h*][1,6]naphthyridine (**P23**)

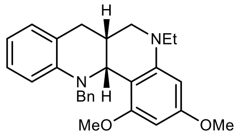

Brown solid; m.p. : 87-89 ºC, (65.4 mg, 79% yield); ^{1}H NMR (400 MHz, $CDCl_3$) δ 7.37 – 7.22 (m, 5H), 7.09 – 7.03 (m, 2H), 6.72 – 6.64 (m, 2H), 6.56 (s, 1H), 6.29 (s, 1H), 4.92 (d, *J* = 17.2 Hz, 1H), 4.55 (d, *J* = 3.2 Hz, 1H), 4.47 (d, *J* = 17.4 Hz, 1H), 3.91 (s, 3H), 3.67 (s, 3H), 3.48 – 3.29 (m, 4H), 3.08 (dd, *J* = 16.6, 5.8 Hz, 1H), 2.81 (dd, *J* = 16.6, 6.8 Hz, 1H), 2.66 – 2.55 (m, 1H), 1.24 (t, *J* = 7.0 Hz, 3H). ^{13}C NMR (101 MHz, $CDCl_3$) δ 149.9, 143.9, 139.6, 139.5, 138.9, 129.6, 128.5, 127.3, 126.7, 126.6, 120.8, 116.5, 114.9, 113.5, 112.6, 96.5, 57.6, 56.9, 56.0, 53.1, 50.0, 45.3, 30.3, 29.9, 11.3. HRMS (ESI): Calcd. for $C_{27}H_{31}N_2O_2$ $[M+H]^+$: 415.2380; found: 415.2373.

(24) (6a-*cis*)-12-benzyl-2-bromo-5-ethyl-5,6,6a,7,12,12a-hexahydrodibenzo[*b*,*h*][1,6]naphthyridine (**P24**)

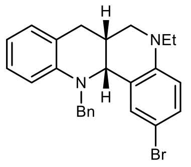

White solid; m.p. : 134-136 ºC, (27.6 mg, 32% yield); ^{1}H NMR (400 MHz, $CDCl_3$) δ 7.36 – 7.23 (m, 5H), 7.17 (dd, *J* = 8.7, 2.4 Hz, 1H), 7.11 – 6.96 (m, 3H), 6.73 – 6.63 (m, 2H), 6.45 (d, *J* = 8.8 Hz, 1H), 5.03 (d, *J* = 17.0 Hz, 1H), 4.60 – 4.41 (m, 2H), 3.56 (dd, *J* = 11.7, 5.0 Hz, 1H), 3.36 (q, *J* = 7.2 Hz, 2H), 3.29 (dd, *J* = 11.6, 5.4 Hz, 1H), 2.87 (dd, *J* = 16.4, 5.4 Hz, 1H), 2.73 (dd, *J* = 16.3, 9.2 Hz, 1H), 2.60 – 2.51 (m, 1H), 1.20 (t, *J* = 7.0 Hz, 3H). ^{13}C NMR (101 MHz, $CDCl_3$) δ 143.3, 142.6, 139.1, 131.1, 130.9, 129.6, 128.6, 127.3, 126.8, 126.6, 124.1, 120.7, 116.7, 112.5, 111.8, 106.8, 58.2, 54.3, 50.7, 44.7, 29.5, 29.4, 10.9. HRMS (ESI): Calcd. for $C_{25}H_{26}BrN_2$ $[M+H]^+$: 433.1274; found: 433.1267.

(25) (6a-*cis*)-12-benzyl-3-chloro-5-ethyl-5,6,6a,7,12,12a-hexahydrodibenzo[*b*,*h*][1,6]naphthyridine (**P25**)

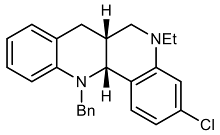

Black solid; m.p. : 90-92 ºC, (34.9 mg, 45% yield); ^{1}H NMR (400 MHz, $CDCl_3$) δ 7.36 – 7.21 (m, 5H), 7.06 – 6.97 (m, 2H), 6.90 (d, *J* = 7.9 Hz, 1H), 6.68 – 6.61 (m, 2H), 6.55 (d, *J* = 1.9 Hz, 1H), 6.50 (dd, *J* = 8.0, 1.8 Hz, 1H), 4.95 (d, *J* = 17.0 Hz, 1H), 4.50 (d, *J* = 3.4 Hz, 1H), 4.46 (d, *J* = 17.2 Hz, 1H), 3.53 (dd, *J* = 11.7, 4.9 Hz, 1H), 3.42 – 3.28 (m, 3H), 2.93 (dd, *J* = 16.4, 5.4 Hz, 1H), 2.72 (dd, *J* = 16.4, 8.6 Hz, 1H), 2.62 – 2.48 (m, 1H), 1.22 (t, *J* = 7.0 Hz, 3H). ^{13}C NMR (101 MHz, $CDCl_3$) δ 144.7, 143.4, 139.2, 134.3, 129.7, 129.6, 128.5, 127.3, 126.7, 126.5, 120.7, 120.2, 116.7, 114.8, 112.6, 109.8, 57.9, 53.9, 50.3, 44.7, 29.5, 29.3, 10.9. HRMS (ESI): Calcd. for $C_{25}H_{26}ClN_2$ $[M+H]^+$: 389.1779; found: 389.1766.

(26) (6a-*cis*)-12-benzyl-5-methyl-5,6,6a,7,12,12a-hexahydrodibenzo[*b*,*h*][1,6]naphthyridine (**P26**)

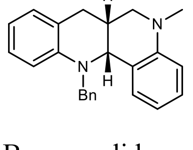

Brown solid; m.p. : 121-123 ºC, (48.3 mg, 71% yield); ^{1}H NMR (500 MHz, $CDCl_3$) δ 7.29 – 7.14 (m, 5H), 7.12 – 7.05 (m, 1H), 7.00 – 6.90 (m, 3H), 6.61 – 6.50 (m, 4H), 4.93 (dd, *J* = 17.1, 4.2 Hz, 1H), 4.51 (d, *J* = 7.4 Hz, 1H), 4.45 (dd, *J* = 17.1, 4.2 Hz, 1H), 3.50 (dd, *J* = 8.2, 5.6 Hz, 1H), 3.15 (dd, *J* = 11.1, 4.4 Hz, 1H), 2.88 (s, 3H), 2.84 – 2.73 (m, 1H), 2.68 – 2.55 (m, 2H). ^{13}C NMR (126 MHz, $CDCl_3$) δ 145.2, 143.7, 139.4, 129.6, 128.5, 128.0, 127.3, 126.7, 126.6, 122.9, 120.8, 116.3, 116.0, 112.1, 110.6, 58.4, 53.9, 53.7, 38.2, 30.6, 30.2. HRMS (ESI): Calcd. for $C_{24}H_{25}N_2$ $[M+H]^+$: 341.2012; found: 341.2010.

(27) (6a-*cis*)-12-benzyl-2-methoxy-5-methyl-5,6,6a,7,12,12a-hexahydrodibenzo[*b*,*h*][1,6]naphthyridine (**P27**)

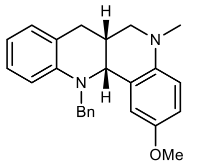

White solid; m.p. : 91-93 ºC, (59.9 mg, 81% yield); ^{1}H NMR (400 MHz, $CDCl_3$) δ 7.39 – 7.32 (m, 4H), 7.30 – 7.25 (m, 1H), 7.06 (t, *J* = 7.8 Hz, 1H), 7.00 (d, *J* = 7.4 Hz, 1H), 6.78 (dd, *J* = 8.6, 2.9 Hz, 1H), 6.73 – 6.57 (m, 4H), 5.07 (d, *J* = 17.2 Hz, 1H), 4.59 (s, 1H), 4.55 (s, 1H), 3.73 (s, 3H), 3.59 (dd, *J* = 10.8, 5.4 Hz, 1H), 3.05 (dd, *J* = 11.2, 3.8 Hz, 1H), 2.92 (s, 3H), 2.80 (d, *J* = 14.4 Hz, 1H), 2.75 – 2.61 (m, 2H). ^{13}C NMR (101 MHz, $CDCl_3$) δ 151.44, 143.67, 139.93, 139.16, 129.43, 128.61, 127.29, 126.80, 126.62, 125.75, 121.00, 116.31, 114.47, 112.68, 111.93, 111.72, 58.55, 55.74, 54.67, 54.43, 38.71, 31.02, 30.45. HRMS (ESI): Calcd. for $C_{25}H_{27}N_2O$ $[M+H]^+$: 371.2118; found: 371.2111.

(28) (6a-*cis*)-12-benzyl-5-isopropyl-5,6,6a,7,12,12a-hexahydrodibenzo[*b*,*h*][1,6]naphthyridine (**P28**)

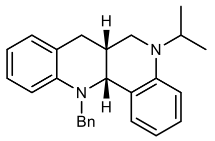

Yellow solid; m.p. : 116-118 ºC, (63.3 mg, 86% yield); ^{1}H NMR (400 MHz, $CDCl_3$) δ 7.40 – 7.24 (m, 5H), 7.18 (t, *J* = 7.8 Hz, 1H), 7.09 – 7.00 (m, 3H), 6.75 (d, *J* = 8.4 Hz, 1H), 6.71 – 6.64 (m, 2H), 6.59 (t, *J* = 7.3 Hz, 1H), 5.03 (d, *J* = 17.2 Hz, 1H), 4.58 (d, *J* = 3.2 Hz, 1H), 4.53 (d, *J* = 17.2 Hz, 1H), 4.21 (p, *J* = 6.8 Hz, 1H), 3.47 (dd, *J* = 11.5, 5.4 Hz, 1H), 3.30 (dd, *J* = 11.5, 6.2 Hz, 1H), 2.95 (dd, *J* = 16.2, 5.4 Hz, 1H), 2.74 (dd, *J* = 16.2, 8.2 Hz, 1H), 2.68 – 2.57 (m, 1H), 1.33 (d, *J* = 6.8 Hz, 3H), 1.29 (d, *J* = 6.8 Hz, 3H). ^{13}C NMR (101 MHz, $CDCl_3$) δ 144.3, 143.8, 139.6, 129.7, 128.9, 128.7, 128.5, 127.2, 126.7, 126.6, 122.4, 120.8, 116.4, 115.0, 112.3, 110.5, 58.6, 53.7, 46.9, 42.8, 29.9, 29.8, 19.2, 18.7. HRMS (ESI): Calcd. for $C_{26}H_{29}N_2$ $[M+H]^+$: 369.2325; found: 369.2316.

(29) (6a-*cis*)-5,12-dibenzyl-5,6,6a,7,12,12a-hexahydrodibenzo[*b*,*h*][1,6]naphthyridine (**P29**)

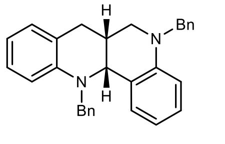

Brown solid; m.p. : 115-117 ºC, (63.2 mg, 76% yield); ^{1}H NMR (400 MHz, $CDCl_3$) δ 7.45 – 7.24 (m, 10H), 7.12 – 6.98 (m, 4H), 6.74 – 6.52 (m, 4H), 5.03 (dd, *J* = 17.2, 2.2 Hz, 1H), 4.67 (d, *J* = 5.4 Hz, 1H), 4.63 – 4.48 (m, 3H), 3.62 (dd, *J* = 11.4, 5.0 Hz, 1H), 3.44 (dd, *J* = 11.5, 6.2 Hz, 1H), 2.98 (dd, *J* = 16.5, 5.4 Hz, 1H), 2.85 (dd, *J* = 16.3, 8.4 Hz, 1H), 2.70 (dd, *J* = 8.8, 2.8 Hz, 1H). ^{13}C NMR (101 MHz, $CDCl_3$) δ 144.6, 143.7, 139.5, 138.6, 129.7, 128.8, 128.7, 128.6, 127.3, 127.1, 126.7, 126.7, 126.6, 122.0, 120.8, 116.5, 116.0, 112.4, 111.1, 58.5, 54.5, 53.8, 51.6, 30.5, 29.8. HRMS (ESI): Calcd. for $C_{30}H_{29}N_2$ $[M+H]^+$: 417.2325; found: 417.2317.

(30) (8a-*cis*)-7,14-dibenzyl-7,8,8a,9,14,14a-hexahydrobenzo[*b*]naphtho[2,1-*h*][1,6]naphthyridine (**P30**)

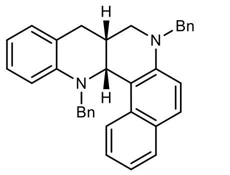

Brown solid; m.p. : 111-113 ºC, (40.0 mg, 43% yield); ^{1}H NMR (500 MHz, $CDCl_3$) δ 7.81 (d, *J* = 8.8 Hz, 1H), 7.61 (d, *J* = 8.0 Hz, 1H), 7.52 (d, *J* = 9.2 Hz, 1H), 7.36 – 7.21 (m, 6H), 7.15 – 7.02 (m, 5H), 6.99 (t, *J* = 7.9 Hz, 1H), 6.92 (d, *J* = 7.4 Hz, 2H), 6.87 (d, *J* = 9.2 Hz, 1H), 6.65 (t, *J* = 7.3 Hz, 1H), 6.54 (d, *J* = 8.4 Hz, 1H), 5.34 (s, 1H), 4.74 (d, *J* = 17.4 Hz, 1H), 4.42 (d, *J* = 17.4 Hz, 2H), 4.21 (d, *J* = 17.0 Hz, 1H), 3.69 (t, *J* = 12.2 Hz, 1H), 3.46 (dd, *J* = 16.9, 6.4 Hz, 1H), 3.30 (dd, *J* = 11.2, 5.5 Hz, 1H), 2.74 (d, *J* = 17.0 Hz, 1H), 2.69 – 2.60 (m, 1H). ^{13}C NMR (126 MHz, $CDCl_3$) δ 144.7, 143.2, 141.1, 138.4, 134.1, 130.0, 129.7, 128.8, 128.6, 128.0, 127.3, 127.1, 126.8, 126.5, 126.4, 126.2, 126.0, 121.0, 120.9, 120.2, 116.7, 114.9, 113.4, 110.7, 54.6, 52.0, 50.3, 48.6, 31.2, 30.9. HRMS (ESI): Calcd. for $C_{34}H_{31}N_2$ $[M+H]^+$: 467.2482; found: 467.2480.

(31) (6a-*cis*)-5-allyl-12-benzyl-5,6,6a,7,12,12a-hexahydrodibenzo[*b*,*h*][1,6]naphthyridine (**P31**)

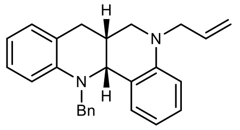

Brown solid; m.p. : 85-87 ºC, (63.0 mg, 86% yield); ^{1}H NMR (400 MHz, $CDCl_3$) δ 7.36 – 7.20 (m, 5H), 7.11 (t, *J* = 7.8 Hz, 1H), 7.06 – 6.97 (m, 3H), 6.65 (d, *J* = 7.8 Hz, 2H), 6.62 – 6.55 (m, 2H), 5.96 – 5.83 (m, 1H), 5.26 (dd, *J* = 11.4, 1.8 Hz, 1H), 5.23 – 5.21 (m, 1H), 4.99 (d, *J* = 17.2 Hz, 1H), 4.60 (d, *J* = 3.4 Hz, 1H), 4.51 (d, *J* = 17.2 Hz, 1H), 3.94 (d, *J* = 4.9 Hz, 2H), 3.55 (dd, *J* = 11.4, 5.2 Hz, 1H), 3.36 (dd, *J* = 11.4, 6.0 Hz, 1H), 2.95 (dd, *J* = 16.2, 5.4 Hz, 1H), 2.77 (dd, *J* = 16.3, 8.4 Hz, 1H), 2.69 – 2.60 (m, 1H). ^{13}C NMR (101 MHz, $CDCl_3$) δ 144.1, 143.7, 139.4, 133.2, 129.6, 128.6, 128.5, 127.2, 126.6, 126.6, 121.9, 120.7, 116.4, 116.2, 115.6, 112.3, 110.8, 58.4, 53.7, 53.0, 51.0, 30.3, 29.8. HRMS (ESI): Calcd. for $C_{26}H_{27}N_2$ $[M+H]^+$: 367.2169; found: 367.2162.

(32) ethyl 2-((6a-*cis*)-12-benzyl-6a,7,12,12a-tetrahydrodibenzo[*b*,*h*][1,6]naphthyridin-5(6H)-yl)-acetate (**P32**)

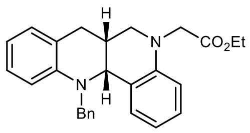

Brown solid; m.p. : 113-115 ºC, (41.2 mg, 50% yield); ^{1}H NMR (400 MHz, $CDCl_3$) δ 7.38 – 7.21 (m, 5H), 7.10 (t, *J* = 7.8 Hz, 1H), 7.05 – 6.95 (m, 3H), 6.70 – 6.56 (m, 3H), 6.41 (d, *J* = 8.2 Hz, 1H), 5.01 (d, *J* = 17.2 Hz, 1H), 4.61 (d, *J* = 3.4 Hz, 1H), 4.53 (d, *J* = 17.2 Hz, 1H), 4.25 (q, *J* = 7.2 Hz, 2H), 4.03 (d, *J* = 4.0 Hz, 2H), 3.70 (dd, *J* = 11.0, 5.2 Hz, 1H), 3.43 (dd, *J* = 11.0, 5.4 Hz, 1H), 3.03 – 2.78 (m, 2H), 2.72 – 2.63 (m, 1H), 1.32 (t, *J* = 7.2 Hz, 3H). ^{13}C NMR (101 MHz, $CDCl_3$) δ 170.8, 143.6, 143.5, 139.3, 129.6, 128.6, 128.5, 128.4, 127.2, 126.7, 126.6, 122.4, 120.9, 116.7, 116.4, 112.2, 110.1, 61.0, 58.3, 54.2, 52.4, 52.2, 30.2, 29.5, 14.3. HRMS (ESI): Calcd. for $C_{27}H_{29}N_2O_2$ $[M+H]^+$: 413.2224; found: 413.2218.

(33) (6a-*cis*)-12-benzyl-2-methyl-5-(*p*-tolyl)-5,6,6a,7,12,12a-hexahydrodibenzo[*b*,*h*][1,6]naphtha-yridine (**P33**)

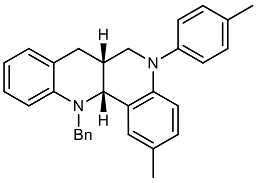

Yellow solid; m.p. : 149-151 ºC, (41.3 mg, 48% yield); ^{1}H NMR (500 MHz, $CDCl_3$) δ 7.21 – 7.14 (m, 4H), 7.14 – 7.09 (m, 1H), 7.07 – 7.00 (m, 4H), 6.93 – 6.85 (m, 2H), 6.77 (s, 1H), 6.70 (dd, *J* = 8.3, 2.0 Hz, 1H), 6.59 (d, *J* = 8.4 Hz, 1H), 6.51 (d, *J* = 7.8 Hz, 2H), 4.94 (d, *J* = 17.2 Hz, 1H), 4.49 (d, *J* = 3.0 Hz, 1H), 4.42 (d, *J* = 17.2 Hz, 1H), 3.77 (dd, *J* = 11.2, 5.2 Hz, 1H), 3.44 (dd, *J* = 11.4, 5.2 Hz, 1H), 2.86 – 2.78 (m, 1H), 2.71 – 2.61 (m, 2H), 2.25 (s, 3H), 2.10 (s, 3H). ^{13}C NMR (126 MHz, $CDCl_3$) δ 144.9, 144.0, 141.1, 139.4, 132.8, 130.0, 129.5, 129.0, 128.6, 128.5, 127.4, 127.3, 126.7, 126.5, 125.3, 123.4, 120.8, 116.4, 116.1, 112.2, 58.5, 54.0, 52.3, 32.0, 30.2, 20.9, 20.7. HRMS (ESI): Calcd. for$C_{31}H_{31}N_2$ $[M+H]^+$: 431.2482; found: 431.2482.

(34) (8a-*cis*)-14-benzyl-5,6,8a,9,14,14a-hexahydro-4H,8H-benzo[*b*]quinolino[1,8-*gh*][1,6]naphth-ayridine (**P34**)

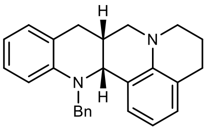

Black solid; m.p. : 99-101 ºC, (51.2 mg, 70% yield); ^{1}H NMR (400 MHz, $CDCl_3$) δ 7.40 – 7.24 (m, 5H), 7.13 – 7.02 (m, 2H), 6.91 (t, *J* = 7.2 Hz, 2H), 6.68 (t, *J* = 7.4 Hz, 2H), 6.52 (t, *J* = 7.4 Hz, 1H), 5.02 (d, *J* = 17.2 Hz, 1H), 4.60 (d, *J* = 3.4 Hz, 1H), 4.53 (d, *J* = 17.2 Hz, 1H), 3.51 (dd, *J* = 11.6, 5.4 Hz, 1H), 3.39 – 3.20 (m, 3H), 3.00 (dd, *J* = 16.2, 5.2 Hz, 1H), 2.89 – 2.74 (m, 3H), 2.72 – 2.62 (m, 1H), 2.08 – 1.95 (m, 2H). ^{13}C NMR (101 MHz, $CDCl_3$) δ 143.8, 141.0, 139.7, 129.6, 128.5, 128.2, 127.3, 126.7, 126.6, 126.6, 121.4, 120.9, 120.8, 116.3, 114.8, 112.2, 58.4, 53.7, 52.2, 49.8, 30.1, 30.0, 27.8, 21.9. HRMS (ESI): Calcd. for $C_{26}H_{27}N_2$ $[M+H]^+$: 367.2169; found: 367.2169.

(35) (8a-*cis*)-14-benzyl-2-methoxy-5,6,8a,9,14,14a-hexahydro-4H,8H-benzo[*b*]quinolino[1,8-*gh*] [1,6]naphthyridine (**P35**)

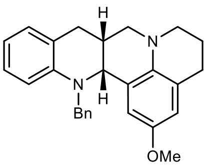

Brown solid; m.p. : 97-99 ºC, (55.6 mg, 70% yield); ^{1}H NMR (500 MHz, $CDCl_3$) δ 7.29 – 7.22 (m, 4H), 7.20 – 7.16 (m, 1H), 6.99 – 6.91 (m, 2H), 6.61 – 6.54 (m, 2H), 6.44 (d, *J* = 11.2 Hz, 2H), 4.95 (d, *J* = 17.0 Hz, 1H), 4.50 – 4.41 (m, 2H), 3.62 (s, 3H), 3.41 (dd, *J* = 11.4, 5.6 Hz, 1H), 3.19 (dt, *J* = 11.3, 5.8 Hz, 1H), 3.10 – 3.01 (m, 2H), 2.86 – 2.66 (m, 4H), 2.62 – 2.54 (m, 1H), 1.99 – 1.88 (m, 2H).^{13}C NMR (126 MHz, $CDCl_3$) δ 150.3, 143.6, 139.4, 135.5, 129.4, 128.5, 127.1, 126.7, 126.6, 123.1, 122.8, 121.1, 116.3, 113.5, 112.4, 112.2, 58.5, 55.7, 54.3, 53.1, 49.9, 30.2, 30.1, 27.9, 22.1. HRMS (ESI): Calcd. for $C_{27}H_{29}N_2O$ $[M+H]^+$: 397.2274; found: 397.2275.

(36) (9a-*cis*)-15-benzyl-4,5,6,7,9a,10,15,15a-octahydro-9H-azepino[3,2,1-*ij*]quinolino[3,2-*c*]quinoline (**P36**)

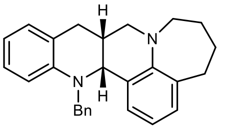

Brown solid; m.p. : 66-68 ºC, (35.7 mg, 47% yield); ^{1}H NMR (400 MHz, $CDCl_3$) δ 7.38 – 7.20 (m, 5H), 7.09 – 7.02 (m, 2H), 6.98 (d, *J* = 7.4 Hz, 1H), 6.90 (d, *J* = 7.6 Hz, 1H), 6.69 – 6.61 (m, 3H), 5.00 (d, *J* = 17.2 Hz, 1H), 4.60 – 4.44 (m, 2H), 3.49 – 3.40 (m, 2H), 3.36 (dd, *J* = 11.1, 6.6 Hz, 1H), 3.28 – 3.20 (m, 1H), 3.04 – 2.95 (m, 1H), 2.92 – 2.79 (m, 2H), 2.76 – 2.63 (m, 2H), 1.97 – 1.75 (m, 4H). ^{13}C NMR (101 MHz, $CDCl_3$) δ 145.9, 144.2, 139.7, 130.9, 130.7, 129.6, 128.5, 127.2, 126.8, 126.6, 126.5, 126.4, 120.8, 117.9, 116.4, 112.3, 58.8, 54.6, 54.1, 53.1, 34.0, 32.5, 30.2, 27.8, 25.6. HRMS (ESI): Calcd. for $C_{27}H_{29}N_2$ $[M+H]^+$: 381.2325; found: 381.2317.

(37) (6a-*cis*)-12-benzyl-5-ethyl-10-phenyl-5,6,6a,7,12,12a-hexahydrobenzo[h]pyrido[2,3-*b*][1,6] naphthyridine (**P37**)

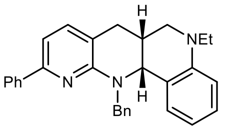

Yellow solid; m.p. : 156-158 ºC, (53.4 mg, 62% yield); ^{1}H NMR (500 MHz, $CDCl_3$) δ 7.87 – 7.84 (m, 2H), 7.26 (t, *J* = 7.9 Hz, 2H), 7.23 – 7.14 (m, 5H), 7.13 – 7.05 (m, 2H), 7.03 – 6.96 (m, 2H), 6.88 (d, *J* = 7.5 Hz, 1H), 6.48 – 6.42 (m, 2H), 6.05 (d, *J* = 15.4 Hz, 1H), 4.51 (d, *J* = 3.8 Hz, 1H), 4.30 (d, *J* = 15.6 Hz, 1H), 3.41 (dd, *J* = 11.7, 5.0 Hz, 1H), 3.23 – 3.12 (m, 2H), 3.02 (dd, *J* = 11.7, 4.6 Hz, 1H), 2.67 – 2.47 (m, 2H), 2.39 – 2.24 (m, 1H), 1.03 (t, *J* = 7.2 Hz, 3H). ^{13}C NMR (126 MHz, $CDCl_3$) δ 154.0, 153.2, 143.5, 140.4, 140.1, 137.2, 128.6, 128.4, 128.3, 128.1, 127.6, 126.6, 126.5, 121.6, 115.5, 114.4, 110.3, 108.6, 56.6, 50.8, 50.6, 44.7, 29.6, 28.9, 11.0. HRMS (ESI): Calcd. for $C_{30}H_{30}N_3$ $[M+H]^+$: 432.2434; found: 432.2428.

(38) (8a-*cis*)-14-benzyl-12-phenyl-5,6,8a,9,14,14a-hexahydro-4H,8H-pyrido[2,3-*b*]quinolino[1,8-*gh*][1,6]naphthyridine (**P38**)

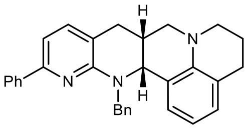

Red solid; m.p. : 131-133 ºC, (44.3 mg, 50% yield); ^{1}H NMR (500 MHz, $CDCl_3$) δ 7.93 (d, *J* = 7.8 Hz, 2H), 7.34 (t, *J* = 7.5 Hz, 2H), 7.30 – 7.13 (m, 7H), 6.96 (d, *J* = 7.6 Hz, 1H), 6.89 (d, *J* = 7.6 Hz, 1H), 6.81 (d, *J* = 7.4 Hz, 1H), 6.45 (t, *J* = 7.4 Hz, 1H), 6.08 – 5.96 (m, 1H), 4.57 (d, *J* = 3.8 Hz, 1H), 4.43 (d, *J* = 15.6 Hz, 1H), 3.39 (dd, *J* = 11.6, 5.2 Hz, 1H), 3.18 (td, *J* = 11.3, 6.7, 4.7 Hz, 1H), 3.04 (td, *J* = 16.6, 11.5, 4.8 Hz, 2H), 2.75 – 2.68 (m, 2H), 2.69 – 2.62 (m, 2H), 2.44 – 2.37 (m, 1H), 1.93 – 1.80 (m, 2H).^{13}C NMR (126 MHz, $CDCl_3$) δ 154.1, 153.2, 140.8, 140.5, 140.1, 137.1, 128.4, 128.3, 128.2, 128.1, 127.6, 126.5, 126.1, 121.4, 120.5, 115.1, 114.4, 108.5, 56.5, 52.3, 50.5, 49.8, 29.6, 29.3, 27.7, 21.8. HRMS (ESI): Calcd. for $C_{31}H_{30}N_3$ $[M+H]^+$: 444.2434; found: 444.2433.

(39) (6a-*cis*)-12-benzyl-5-ethyl-10-(furan-2-yl)-5,6,6a,7,12,12a-hexahydrobenzo[*h*]pyrido[2,3-*b*][1,6]naphthyridine (**P39**)

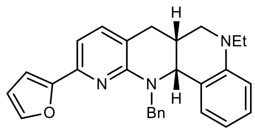

Black solid; m.p. : 136-138 ºC, (48.8 mg, 58% yield); ^{1}H NMR (500 MHz, $CDCl_3$) δ 7.40 (s, 1H), 7.31 – 7.16 (m, 5H), 7.14 – 7.01 (m, 3H), 6.91 (dd, *J* = 7.5, 1.6 Hz, 1H), 6.86 – 6.80 (m, 1H), 6.58 – 6.51 (m, 2H), 6.42 (s, 1H), 6.03 (d, *J* = 15.4 Hz, 1H), 4.58 (d, *J* = 3.6 Hz, 1H), 4.33 (d, *J* = 15.4 Hz, 1H), 3.49 (dd, *J* = 11.8, 4.8 Hz, 1H), 3.37 – 3.20 (m, *J* = 7.2 Hz, 2H), 3.10 (dd, *J* = 12.0, 4.6 Hz, 1H), 2.71 – 2.57 (m, 2H), 2.41 – 2.29 (m, 1H), 1.19 – 1.08 (m, 3H). ^{13}C NMR (126 MHz, $CDCl_3$) δ 155.1, 153.9, 145.7, 143.5, 142.2, 140.3, 136.9, 128.5, 128.3, 128.1, 127.7, 126.6, 121.5, 115.4, 114.6, 111.7, 110.2, 107.1, 107.0, 56.5, 50.8, 50.5, 44.6, 29.5, 29.0, 11.0. HRMS (ESI): Calcd. for $C_{28}H_{28}N_3O$ $[M+H]^+$: 422.2227; found: 422.2218.

(40) (6a-*cis*)-12-benzyl-5-ethyl-10-(thiophen-2-yl)-5,6,6a,7,12,12a-hexahydrobenzo[*h*]pyrido[2,3-*b*][1,6]naphthyridine (**P40**)

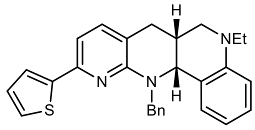

Black solid; m.p. : 81-83 ºC, (63.8 mg, 73% yield); ^{1}H NMR (500 MHz, $CDCl_3$) δ 7.44 (dd, *J* = 3.7, 1.2 Hz, 1H), 7.32 (d, *J* = 7.6 Hz, 2H), 7.29 – 7.14 (m, 4H), 7.13 – 7.02 (m, 3H), 7.01 (dd, *J* = 5.0, 3.6 Hz, 1H), 6.86 (d, *J* = 7.6 Hz, 1H), 6.59 – 6.49 (m, 2H), 6.02 (s, 1H), 4.60 (d, *J* = 3.8 Hz, 1H), 4.35 (d, *J* = 15.4 Hz, 1H), 3.51 (dd, *J* = 11.7, 4.8 Hz, 1H), 3.35 – 3.18 (m, *J* = 7.4 Hz, 2H), 3.10 (dd, *J* = 11.7, 4.4 Hz, 1H), 2.64 (dd, *J* = 8.2, 3.6 Hz, 2H), 2.44 – 2.29 (m, 1H), 1.11 (t, *J* = 7.0 Hz, 3H). ^{13}C NMR (126 MHz, $CDCl_3$) δ 153.6, 148.6, 146.7, 143.5, 140.2, 137.0, 128.5, 128.3, 127.8, 127.63, 126.6, 126.1, 123.1, 121.5, 115.5, 114.3, 110.2, 107.0, 56.6, 50.9, 50.6, 44.6, 29.5, 28.8, 11.0. HRMS (ESI): Calcd. for $C_{28}H_{28}N_3S$ $[M+H]^+$: 438.1998; found: 438.1988.

(41) (9a-*cis*)-15-benzyl-13-(thiophen-2-yl)-4,5,6,7,9a,10,15,15a-octahydro-9H-azepino[3',2',1':8,1]quinolino[4,3-*b*][1,8]naphthyridine (**P41**)

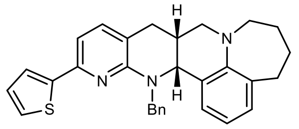

Brown solid; m.p. : 92-94 ºC, (61.2 mg, 66% yield); ^{1}H NMR (500 MHz, $CDCl_3$) δ 7.35 (d, *J* = 3.4 Hz, 1H), 7.21 (d, *J* = 7.2 Hz, 2H), 7.16 – 7.11 (m, 3H), 7.09 (d, *J* = 2.8 Hz, 1H), 6.99 (dd, *J* = 7.5, 2.0 Hz, 1H), 6.94 – 6.88 (m, 1H), 6.85 – 6.73 (m, 3H), 6.56 – 6.50 (m, 1H), 5.80 (s, 1H), 4.42 (d, *J* = 3.6 Hz, 1H), 4.38 – 4.28 (m, 1H), 3.29 – 3.13 (m, 2H), 3.08 – 3.01 (m, 1H), 2.97 – 2.85 (m, 1H), 2.78 – 2.55 (m, 3H), 2.50 – 2.42 (m, 1H), 2.38 – 2.32 (m, 1H), 1.78 – 1.55 (m, 4H). ^{13}C NMR (126 MHz, $CDCl_3$) δ 154.1, 148.6, 146.7, 145.6, 140.4, 137.0, 130.9, 130.6, 128.2, 127.9, 127.6, 126.5, 126.2, 126.1, 125.9, 123.1, 118.1, 114.3, 107.0, 57.1, 54.3, 54.0, 50.3, 33.9, 32.2, 29.3, 27.7, 25.6. HRMS (ESI): Calcd. for $C_{30}H_{30}N_3S$ $[M+H]^+$: 464.2155; found: 464.2154.

(42) (6a-*cis*)-12-benzyl-2-methoxy-5-methyl-10-(1-methyl-1H-pyrrol-2-yl)-5,6,6a,7,12,12a-hexahydrobenzo[*h*]pyrido[2,3-*b*][1,6]naphthyridine (**P42**)

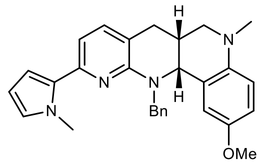

Brown solid; m.p. : 92-95 ºC, (46.8 mg, 52% yield); ^{1}H NMR (500 MHz, $CDCl_3$) δ 7.29 – 7.21 (m, 4H), 7.21 – 7.16 (m, 1H), 7.09 (d, *J* = 7.6 Hz, 1H), 6.79 (d, *J* = 7.6 Hz, 1H), 6.72 (dd, *J* = 8.7, 3.0 Hz, 1H), 6.68 (d, *J* = 3.0 Hz, 1H), 6.57 (d, *J* = 2.2 Hz, 1H), 6.53 (d, *J* = 8.8 Hz, 1H), 6.47 (dt, *J* = 3.1, 1.4 Hz, 1H), 6.11 – 6.07 (m, 1H), 6.01 (d, *J* = 16.0 Hz, 1H), 4.54 (d, *J* = 3.4 Hz, 1H), 4.32 (d, *J* = 16.2 Hz, 1H), 3.67 (s, 3H), 3.66 (s, 3H), 3.49 (dd, *J* = 11.2, 5.4 Hz, 1H), 2.86 (dd, *J* = 11.2, 3.0 Hz, 1H), 2.81 (s, 3H), 2.63 – 2.52 (m, 3H).^{13}C NMR (126 MHz, $CDCl_3$) δ 153.4, 151.7, 149.2, 139.8, 139.7, 136.8, 133.1, 128.3, 126.9, 126.5, 125.5, 125.3, 113.8, 112.8, 112.1, 111.9, 110.0, 109.5, 107.1, 56.7, 55.8, 54.5, 51.0, 38.7, 36.9, 31.0, 30.0. HRMS (ESI): Calcd. for $C_{29}H_{31}N_4O$ $[M+H]^+$: 451.2492; found: 451.2490.

(43) (6a-*cis*)-11-benzyl-5-ethyl-5,6,6a,7,11,11a-hexahydrobenzo[*h*]thieno[3,2-*b*][1,6]naphthyridine (**P43**)

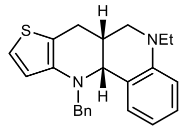

Brown solid; m.p. : 127-129 ºC, (36.1 mg, 50% yield); ^{1}H NMR (500 MHz, $CDCl_3$) δ 7.22 – 7.08 (m, 5H), 7.01 – 6.95 (m, 2H), 6.87 (d, *J* = 5.4 Hz, 1H), 6.52 (d, *J* = 5.4 Hz, 1H), 6.48 (d, *J* = 8.2 Hz, 1H), 6.41 (t, *J* = 7.3 Hz, 1H), 4.53 (d, *J* = 16.2 Hz, 1H), 4.19 (d, *J* = 3.2 Hz, 1H), 4.15 (d, *J* = 16.2 Hz, 1H), 3.35 – 3.18 (m, 4H), 2.90 – 2.84 (m, 1H), 2.52 (dd, *J* = 16.4, 6.2 Hz, 1H), 2.43 – 2.34 (m, 1H), 1.08 (t, *J* = 7.2 Hz, 3H).^{13}C NMR (126 MHz, $CDCl_3$) δ 144.0, 142.7, 140.3, 129.2, 128.7, 128.4, 127.24, 126.7, 121.4, 121.0, 119.2, 114.7, 112.4, 110.0, 57.2, 49.9, 46.8, 44.7, 25.0, 11.0, 1.0. HRMS (ESI): Calcd. for $C_{23}H_{25}N_2S$ $[M+H]^+$: 361.1733; found: 361.1729.

(44) (6a-*cis*)-11-benzyl-5-cyclohexyl-5,6,6a,7,11,11a-hexahydrobenzo[h]thieno[3,2-*b*][1,6]naphthyridine (**P44**)

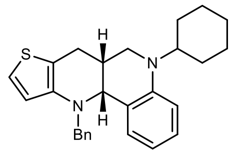

Brwon solid; m.p. : 87-89 ºC, (51.3 mg, 62% yield); ^{1}H NMR (400 MHz, $CDCl_3$) δ 7.31 – 7.24 (m, 4H), 7.22 (d, *J* = 5.3, 3.8 Hz, 1H), 7.14 – 7.05 (m, 2H), 6.97 (d, *J* = 5.4 Hz, 1H), 6.64 (d, *J* = 8.4 Hz, 1H), 6.60 (d, *J* = 5.4 Hz, 1H), 6.51 (t, *J* = 7.3 Hz, 1H), 4.64 (d, *J* = 16.2 Hz, 1H), 4.30 – 4.19 (m, 2H), 3.64 (td, *J* = 12.8, 12.0, 6.6 Hz, 1H), 3.41 – 3.20 (m, 2H), 2.97 (dd, *J* = 16.5, 6.0 Hz, 1H), 2.59 (dd, *J* = 16.3, 5.6 Hz, 1H), 2.51 – 2.41 (m, 1H), 1.98 – 1.79 (m, 4H), 1.74 (d, *J* = 13.2 Hz, 1H), 1.62 – 1.49 (m, 2H), 1.47 – 1.36 (m, 3H).^{13}C NMR (101 MHz, $CDCl_3$) δ 144.3, 142.9, 140.5, 129.1, 128.8, 128.3, 127.2, 126.6, 121.3, 121.3, 119.2, 114.3, 113.2, 110.2, 57.4, 56.1, 43.6, 30.1, 29.5,

29.3, 26.3, 26.2, 26.1, 26.0, 25.2. HRMS (ESI): Calcd. for $C_{27}H_{31}N_2S$ [M+H]$^+$: 415.2202; found: 415.2200

(45) (7a-*cis*)-7-benzyl-12-ethyl-7,7a,12,13,13a,14-hexahydroquinolino[4,3-*j*][1,7]phenanthroline (**P45**)

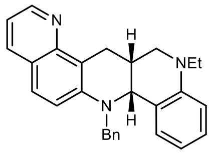

Brown solid; m.p. : 125-127 ºC, (59.9 mg, 74% yield); ^{1}H NMR (500 MHz, $CDCl_3$) δ 8.74 (dd, *J* = 4.3, 1.8 Hz, 1H), 7.85 (dd, *J* = 8.0, 2.0 Hz, 1H), 7.42 (d, *J* = 9.2 Hz, 1H), 7.28 (s, 2H), 7.27 (s, 2H), 7.24 – 7.18 (m, 1H), 7.10 (d, *J* = 9.2 Hz, 1H), 7.06 – 7.01 (m, 2H), 6.98 (d, *J* = 7.6 Hz, 1H), 6.54 (d, *J* = 8.8 Hz, 1H), 6.46 (t, *J* = 7.4 Hz, 1H), 5.13 (d, *J* = 17.2 Hz, 1H), 4.64 – 4.53 (m, 2H), 3.59 (dd, *J* = 11.6, 4.8 Hz, 1H), 3.52 (dd, *J* = 17.4, 5.8 Hz, 1H), 3.41 – 3.25 (m, 3H), 3.09 (dd, *J* = 17.4, 9.4 Hz, 1H), 2.70 – 2.58 (m, 1H), 1.17 (t, *J* = 7.0 Hz, 3H). ^{13}C NMR (126 MHz, $CDCl_3$) δ 149.7, 147.8, 143.9, 143.9, 139.1, 135.8, 128.7, 128.6, 128.0, 127.0, 126.6, 126.6, 121.4, 121.1, 117.0, 116.3, 115.0, 114.2, 110.2, 58.6, 54.7, 50.9, 44.8, 29.1, 24.5, 11.1. HRMS (ESI): Calcd. for $C_{28}H_{28}N_3$ [M+H]$^+$: 406.2278; found: 406.2268.

(46) (7a-*cis*)-7-benzyl-12-ethyl-10-methyl-7,7a,12,13,13a,14-hexahydroquinolino[4,3-*j*][1,7]phenanthroline (**P46**)

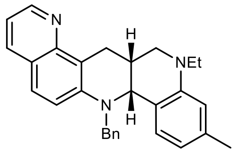

Yellow solid; m.p. : 111-113 ºC, (55.3 mg, 66% yield); ^{1}H NMR (400 MHz, $CDCl_3$) δ 8.79 (dd, *J* = 4.3, 1.8 Hz, 1H), 7.91 (dd, *J* = 8.0, 1.8 Hz, 1H), 7.47 (d, *J* = 9.2 Hz, 1H), 7.32 (d, *J* = 4.4 Hz, 4H), 7.28 – 7.22 (m, 1H), 7.14 (d, *J* = 9.2 Hz, 1H), 7.10 (dd, *J* = 8.1, 4.4 Hz, 1H), 6.90 (d, *J* = 7.6 Hz, 1H), 6.41 (s, 1H), 6.33 (d, *J* = 7.6 Hz, 1H), 5.16 (d, *J* = 17.2 Hz, 1H), 4.70 – 4.57 (m, 2H), 3.62 (dd, *J* = 11.6, 4.8 Hz, 1H), 3.55 (dd, *J* = 17.5, 6.0 Hz, 1H), 3.45 – 3.34 (m, 3H), 3.14 (dd, *J* = 17.5, 9.0 Hz, 1H), 2.73 – 2.62 (m, 1H), 2.25 (s, 3H), 1.22 (t, *J* = 7.0 Hz, 3H).^{13}C NMR (126 MHz, $CDCl_3$) δ 149.6, 147.8, 143.8, 143.8, 139.2, 138.3, 135.8, 128.6, 128.0, 126.9, 126.6, 126.5, 121.3, 118.3, 116.9, 116.3, 115.8, 114.2, 110.9, 58.4, 54.6, 50.9, 44.8, 29.1, 24.4, 21.7, 11.2. HRMS (ESI): Calcd. for $C_{29}H_{30}N_3$ [M+H]$^+$: 420.2434; found: 420.2430.

(47) (7a-*cis*)-14-benzyl-9-ethyl-7,7a,8,9,13b,14-hexahydroquinolino[4,3-*b*][1,10]phenanthroline (**P47**)

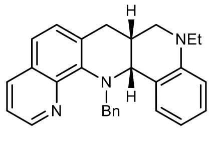

Brown solid; m.p. : 146-148 ºC, (68.8 mg, 85% yield); ^{1}H NMR (500 MHz, $CDCl_3$) δ 8.89 (d, *J* = 5.8 Hz, 1H), 7.96 (d, *J* = 9.8 Hz, 1H), 7.80 (d, *J* = 7.6 Hz, 2H), 7.57 (d, *J* = 7.6 Hz, 1H), 7.32 (m, 2H), 7.30 – 7.16 (m, 3H), 7.02 (d, *J* = 8.4 Hz, 1H), 6.89 (t, *J* = 7.18 Hz, 1H), 6.40 (d, *J* = 8.2 Hz, 1H), 6.34 (t, *J* = 7.4 Hz, 1H), 5.26 (d, *J* = 14.4 Hz, 1H), 4.46 (d, *J* = 14.4 Hz, 1H), 4.33 (d, *J* = 4.2

Hz, 1H), 3.64 (dd, J = 11.8, 3.8 Hz, 1H), 3.34 (dd, J = 14.8, 7.2 Hz, 1H), 3.24 (dd, J = 14.8, 7.4 Hz, 1H), 3.19 (d, J = 11.6 Hz, 1H), 2.81 – 2.66 (m, 2H), 2.48 – 2.37 (m, 1H), 1.11 (t, J = 7.0 Hz, 3H). ^{13}C NMR (126 MHz, $CDCl_3$) δ 147.9, 143.8, 143.7, 141.5, 140.9, 136.1, 129.1, 128.9, 128.3, 128.2, 127.8, 127.7, 127.4, 127.1, 121.7, 120.3, 120.2, 115.6, 109.2, 60.0, 55.5, 51.9, 44.9, 28.3, 23.5, 11.0. HRMS (ESI): Calcd. for $C_{28}H_{28}N_3$ $[M+H]^+$: 406.2278; found: 406.2267.

(48) (*cis*)-5-benzyl-10-bromo-12-ethyl-4b,5,6,10b,11,12-hexahydrodibenzo[*c*,*h*][1,6]naphthyridine (**P48**)

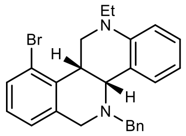

Brown solid; m.p. : 93-95 ºC, (51.8 mg, 60% yield); ^{1}H NMR (400 MHz, $CDCl_3$) δ 7.45 (d, J = 7.8 Hz, 1H), 7.36 – 7.20 (m, 6H), 7.14 (dd, J = 7.4, 1.8 Hz, 1H), 7.05 (t, J = 7.6 Hz, 1H), 6.95 (d, J = 7.6 Hz, 1H), 6.79 (d, J = 8.2 Hz, 1H), 6.65 (t, J = 7.2 Hz, 1H), 4.27 – 4.11 (m, 1H), 3.95 (ddd, J = 13.2, 8.6, 2.8 Hz, 2H), 3.69 – 3.55 (m, 2H), 3.52 – 3.35 (m, 4H), 3.15 (dd, J = 13.2, 1.8 Hz, 1H), 1.28 (t, J = 7.0 Hz, 3H). ^{13}C NMR (101 MHz, $CDCl_3$) δ 144.6, 140.4, 138.1, 135.9, 133.4, 130.5, 129.6, 128.3, 128.2, 127.8, 126.7, 125.8, 124.6, 119.1, 114.3, 111.1, 61.4, 57.6, 55.5, 49.0, 45.0, 38.8, 11.2. HRMS (ESI): Calcd. for $C_{25}H_{26}BrN_2$ $[M+H]^+$: 433.1274; found: 433.1271.

(49) (*cis*)-5-benzyl-12-ethyl-10-nitro-4b,5,6,10b,11,12-hexahydrodibenzo[*c*,*h*][1,6]naphthyridine (**P49**)

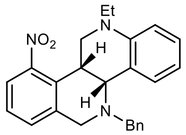

Yellow solid; m.p. : 94-96 ºC, (39.1 mg, 49% yield); ^{1}H NMR (400 MHz, $CDCl_3$) δ 7.81 (d, J = 8.0 Hz, 1H), 7.31 – 7.16 (m, 8H), 7.10 (dd, J = 7.4, 1.6 Hz, 1H), 6.74 (d, J = 8.2 Hz, 1H), 6.60 (td, J = 7.2, 1.0 Hz, 1H), 4.13 (d, J = 13.2 Hz, 1H), 4.07 – 3.99 (m, 2H), 3.86 – 3.76 (m, 1H), 3.65 (t, J = 1.6 Hz, 1H), 3.60-3.51 (m, 2H), 3.43-3.34 (m, 2H), 3.11 (d, J = 16 Hz, 1H), 1.23 (t, J = 8 Hz, 3H).^{13}C NMR (101 MHz, $CDCl_3$) δ 149.0, 144.4, 140.0, 139.0, 133.0, 131.7, 131.6, 129.6, 128.4, 128.2, 126.9, 126.8, 122.9, 118.6, 114.3, 111.1, 60.8, 57.7, 55.4, 50.4, 45.0, 35.1, 11.1. HRMS (ESI): Calcd. for $C_{25}H_{26}N_3O_2$ $[M+H]^+$: 400.2020; found: 400.2018.

(50) (*cis*)-1-benzyl-6-ethyl-4-phenyl-1,4,4a,5,6,10*b*-hexahydrobenzo[*h*][1,6]naphthyridine (**P50**)

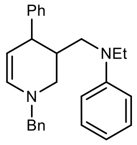

Yellowish oil (25.0 mg, 49% yield); ^{1}H NMR (500 MHz, $CDCl_3$) δ 7.42 – 7.40 (m, 2H), 7.37 – 7.32 (m, 6H), 7.29 – 7.25 (m, 2H), 7.14 – 7.10 (m, 2H), 6.65 (d, J = 8.0 Hz, 2H), 6.58 (t, J = 7.2 Hz, 1H), 5.88 (dd, J = 4.7, 2.3 Hz, 1H), 3.67 (d, J = 12.7 Hz, 1H), 3.53 – 3.48 (m, 2H), 3.38 (dd, J = 17.2, 4.6 Hz, 1H), 3.23 (dq, J = 14.0, 6.9 Hz, 1H), 3.11 (dd, J = 14.2, 3.5 Hz, 1H), 3.05 (d, J = 11.4 Hz, 2H), 2.95 (dq, J = 14.2, 6.9 Hz, 1H), 2.86 – 2.82 (m, 1H), 2.39 (dd, J = 11.5, 3.8 Hz, 1H), 0.96 (t, J = 7.0 Hz, 3H). ^{13}C NMR (126 MHz, $CDCl_3$) δ 148.1, 140.9, 138.6, 138.1, 129.4, 129.1, 128.5, 128.3,

127.3, 127.2, 126.1, 124.8, 115.3, 112.1, 62.9, 53.7, 52.3, 51.6, 44.9, 37.4, 11.5. HRMS (ESI): Calcd. for $C_{27}H_{31}N_2$ $[M+H]^+$: 383.2482; found: 383.2476.

(51) (*cis*)-5-benzyl-12-ethyl-10b-methyl-4b,5,6,10b,11,12-hexahydrodibenzo[*c*,*h*][1,6]naphthyridine (**P51**)

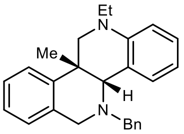

Brown solid; m.p. : 140-142 ºC, (61.1mg, 83% yield); ^{1}H NMR (400 MHz, $CDCl_3$) δ 7.44 (d, *J* = 7.8 Hz, 1H), 7.32 – 7.24 (m, 5H), 7.23 – 7.13 (m, 4H), 6.98 (d, *J* = 7.6 Hz, 1H), 6.72 (d, *J* = 8.4 Hz, 1H), 6.65 (t, *J* = 7.2 Hz, 1H), 4.06 (d, *J* = 11.2 Hz, 1H), 3.98 (d, *J* = 13.4 Hz, 1H), 3.89 (d, *J* = 15.4 Hz, 1H), 3.67 – 3.57 (m, 2H), 3.42 (s, 1H), 3.35 (dt, *J* = 14.4, 7.2 Hz, 1H), 3.19 (d, *J* = 13.4 Hz, 1H), 2.94 (dd, *J* = 11.0, 1.4 Hz, 1H), 1.28 (s, 3H), 1.23 (t, *J* = 12.0, 4.0 Hz, 3H). ^{13}C NMR (101 MHz, $CDCl_3$) δ 143.6, 141.4, 140.8, 135.0, 133.9, 129.1, 128.3, 128.1, 126.5, 126.5, 126.4, 126.3, 126.1, 118.8, 114.3, 110.5, 68.0, 58.0, 57.1, 55.6, 44.8, 36.6, 22.7, 11.0. HRMS (ESI): Calcd. for $C_{26}H_{29}N_2$ $[M+H]^+$: 369.2325; found: 369.2323.

(52) (*cis*)-5-benzyl-12-ethyl-9,10b-dimethyl-4b,5,6,10b,11,12-hexahydrodibenzo[*c*,*h*][1,6]naphthyridine (**P52**)

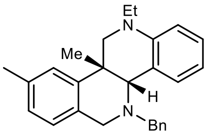

Brown solid; m.p. : 115-117 ºC, (53.4 mg, 70% yield); ^{1}H NMR (400 MHz, $CDCl_3$) δ 7.29 – 7.12 (m, 8H), 6.99 (d, *J* = 7.6 Hz, 1H), 6.86 (d, *J* = 7.6 Hz, 1H), 6.70 (d, *J* = 8.2 Hz, 1H), 6.63 (t, *J* = 7.2 Hz, 1H), 4.03 (d, *J* = 11.2 Hz, 1H), 3.95 (d, *J* = 13.4 Hz, 1H), 3.84 (d, *J* = 15.2 Hz, 1H), 3.66 – 3.51 (m, 2H), 3.43 – 3.28 (m, 2H), 3.17 (d, *J* = 13.2 Hz, 1H), 2.93 (d, *J* = 11.4 Hz, 1H), 2.38 (s, 3H), 1.26 (s, 3H), 1.21 (t, *J* = 7.0 Hz, 3H).^{13}C NMR (101 MHz, $CDCl_3$) δ 143.6, 141.2, 140.9, 135.6, 133.9, 131.9, 129.0, 128.3, 128.0, 127.0, 126.9, 126.5, 126.4, 118.9, 114.3, 110.4, 68.0, 57.9, 57.1, 55.4, 44.8, 36.5, 22.7, 21.4, 11.0. HRMS (ESI): Calcd. for $C_{27}H_{31}N_2$ $[M+H]^+$: 383.2482; found: 383.2478.

(53) (*cis*)-5-benzyl-9-bromo-12-ethyl-10b-methyl-4b,5,6,10b,11,12-hexahydrodibenzo[*c*,*h*][1,6] naphthyridine (**P53**)

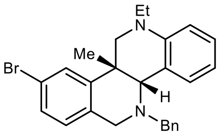

Brown solid; m.p. : 94-96 ºC, 58.9 mg, 66% yield); ^{1}H NMR (500 MHz, $CDCl_3$) δ 7.41 (t, *J* = 2.4 Hz, 1H), 7.15 – 7.09 (m, 5H), 7.08 – 6.98 (m, 3H), 6.69 (d, *J* = 8.2 Hz, 1H), 6.57 (dd, *J* = 8.2, 2.4 Hz, 1H), 6.51 (td, *J* = 7.2, 2.6 Hz, 1H), 3.88 – 3.81 (m, 2H), 3.69 (dd, *J* = 15.4, 2.8 Hz, 1H), 3.50 – 3.42 (m, 1H), 3.38 (dd, *J* = 15.4, 2.6 Hz, 1H), 3.25 – 3.18 (m, 2H), 3.06 (dd, *J* = 13.4, 2.6 Hz, 1H), 2.78 (dt, *J* = 11.2, 1.8 Hz, 1H), 1.12 (d, *J* = 2.4 Hz, 3H), 1.08 (td, *J* = 7.0, 2.2 Hz, 3H). ^{13}C NMR (126 MHz, $CDCl_3$) δ 143.8, 143.6, 140.5, 134.0, 133.8, 129.5, 129.2, 129.2, 128.3, 128.2, 128.2, 126.7, 119.9, 118.4, 114.5, 110.6, 67.6, 57.9, 56.9, 55.0, 44.8, 36.9, 22.8, 11.1. HRMS (ESI): Calcd. for $C_{26}H_{28}BrN_2$ $[M+H]^+$: 447.1430; found: 447.1429.

(54) (*cis*)-5-benzyl-9-chloro-12-ethyl-10b-methyl-4b,5,6,10b,11,12-hexahydrodibenzo[*c*,*h*][1,6] naphthyridine (**P54**)

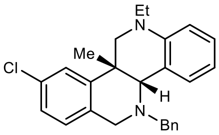

Yellow solid; m.p. : 96-98 ºC, (59.5 mg, 74% yield); ^{1}H NMR (400 MHz, $CDCl_3$) δ 7.42 (t, *J* = 2.2 Hz, 1H), 7.28 (t, *J* = 3.0 Hz, 4H), 7.25 – 7.18 (m, 3H), 7.15 (dt, *J* = 8.2, 2.0 Hz, 1H), 6.90 (d, *J* = 8.2 Hz, 1H), 6.76 – 6.71 (m, 1H), 6.67 (td, *J* = 7.5, 2.2 Hz, 1H), 4.08 – 3.94 (m, 2H), 3.87 (dd, *J* = 15.4, 2.2 Hz, 1H), 3.67 – 3.52 (m, 2H), 3.44 – 3.33 (m, 2H), 3.22 (dd, *J* = 13.4, 2.2 Hz, 1H), 2.94 (dt, *J* = 11.4, 1.8 Hz, 1H), 1.28 (d, *J* = 2.0 Hz, 3H), 1.24 (td, *J* = 7.2, 2.0 Hz, 3H). ^{13}C NMR (101 MHz, $CDCl_3$) δ 143.6, 143.4, 140.6, 133.8, 133.5, 131.9, 129.3, 128.3, 128.2, 127.9, 126.7, 126.6, 126.3, 118.5, 114.6, 110.6, 67.6, 58.0, 56.9, 55.0, 44.9, 36.9 22.8, 11.1. HRMS (ESI): Calcd. for $C_{26}H_{28}ClN_2$ $[M+H]^+$: 403.1936; found: 403.1935.

(55) (*cis*)-5-benzyl-12-ethyl-9-fluoro-10b-methyl-4b,5,6,10b,11,12-hexahydrodibenzo[*c*,*h*][1,6] naphthyridine (**P55**)

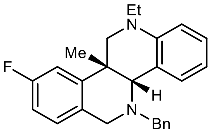

Brown solid; m.p. : 81-83 ºC, (50.2 mg, 65% yield); ^{1}H NMR (400 MHz, $CDCl_3$) δ 7.30 – 7.18 (m, 7H), 7.13 (dt, *J* = 10.6, 2.2 Hz, 1H), 6.96 – 6.83 (m, 2H), 6.73 (d, *J* = 8.2 Hz, 1H), 6.66 (t, *J* = 7.2 Hz, 1H), 4.07 – 3.94 (m, 2H), 3.86 (d, *J* = 15.2 Hz, 1H), 3.69 – 3.51 (m, 2H), 3.43 – 3.30 (m, 2H), 3.21 (dd, *J* = 13.2, 1.6 Hz, 1H), 2.94 (t, *J* = 10.6 Hz, 1H), 1.26 (dd, *J* = 5.8, 1.6 Hz, 3H), 1.23 (dd, *J* = 7.2, 1.4 Hz, 3H).^{13}C NMR (101 MHz, $CDCl_3$) δ 162.7, 160.3, 143.6, 143.5, 140.6, 133.8, 130.6, 130.5, 129.2, 128.3, 128.1, 127.9, 127.8, 126.6, 118.5, 114.5, 113.4, 113.2, 113.1, 112.9, 110.6, 67.6, 57.9, 56.9, 54.9, 44.9, 36.9, 22.8, 11.0. ^{19}F NMR (376 MHz, $CDCl_3$) δ -116.3. HRMS (ESI): Calcd. for $C_{26}H_{28}FN_2$ $[M+H]^+$: 387.2231; found: 387.2229.

(56) (*cis*)-5-benzyl-8-bromo-12-ethyl-10b-methyl-4b,5,6,10b,11,12-hexahydrodibenzo[*c*,*h*][1,6] naphthyridine (**P56**)

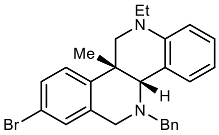

Black solid; m.p. : 74-76 ºC, (53.5 mg, 60% yield); ^{1}H NMR (500 MHz, $CDCl_3$) δ 7.29 (dd, *J* = 8.4, 2.2 Hz, 1H), 7.24 – 7.17 (m, 5H), 7.17 – 7.09 (m, 3H), 7.05 (d, *J* = 2.0 Hz, 1H), 6.65 (d, *J* = 8.2 Hz, 1H), 6.59 (tt, *J* = 7.2, 1.4 Hz, 1H), 3.97 – 3.85 (m, 2H), 3.81 – 3.67 (m, 1H), 3.63 – 3.45 (m, 2H), 3.33 (d, *J* = 1.6 Hz, 1H), 3.32 – 3.22 (m, 1H), 3.13 (dd, *J* = 13.2, 1.6 Hz, 1H), 2.84 (dd, *J* = 11.0, 1.6 Hz, 1H), 1.18 (d, *J* = 1.6 Hz, 3H), 1.15 (td, *J* = 7.0, 1.6 Hz, 3H). ^{13}C NMR (126 MHz, $CDCl_3$) δ 143.6, 140.5, 140.4, 137.4, 133.8, 129.4, 129.3, 129.2, 128.3, 128.3, 128.2, 126.7, 119.9, 118.5, 114.6, 110.6, 67.7, 57.9, 56.9, 54.9, 44.9, 36.5, 22.8, 11.0. HRMS (ESI): Calcd. for $C_{26}H_{28}BrN_2$ $[M+H]^+$: 447.1430; found: 447.1425.

(57) (*cis*)-5-benzyl-7-bromo-12-ethyl-10b-methyl-4b,5,6,10b,11,12-hexahydrodibenzo[*c*,*h*][1,6] naphthyridine (**P57**)

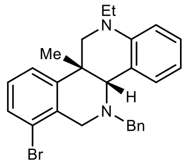

Yellow solid; m.p. : 117-119 ºC, (74.0 mg, 83% yield); ^{1}H NMR (400 MHz, $CDCl_3$) δ 7.48 – 7.39 (m, 2H), 7.28 (d, *J* = 3.6 Hz, 4H), 7.23 (dd, *J* = 10.0, 5.6 Hz, 3H), 7.14 (t, *J* = 7.8 Hz, 1H), 6.77 – 6.63 (m, 2H), 4.05 (dd, *J* = 18.2, 11.8 Hz, 3H), 3.62 (dq, *J* = 14.2, 7.0 Hz, 1H), 3.53 – 3.41 (m, 2H), 3.37 (dd, *J* = 14.6, 7.2 Hz, 1H), 3.31 (d, *J* = 13.4 Hz, 1H), 2.97 (dd, *J* = 11.2, 1.4 Hz, 1H), 1.28 (s, 3H), 1.24 (t, *J* = 7.0 Hz, 3H). ^{13}C NMR (101 MHz, $CDCl_3$) δ 144.2, 143.6, 140.4, 134.6, 133.7, 130.3, 129.2, 128.4, 128.1, 127.5, 126.6, 125.7, 122.7, 118.6, 114.6, 110.5, 67.2, 58.0, 56.9, 56.4, 44.9, 37.1, 23.1, 11.1. HRMS (ESI): Calcd. for $C_{26}H_{28}BrN_2$ $[M+H]^+$: 447.1430; found: 447.1429.

(58) (*cis*)-12-ethyl-10b-methyl-5-(4-(4,4,5,5-tetramethyl-1,3,2-dioxaborolan-2-yl)benzyl)-4b,5,6,10b,11,12-hexahydrodibenzo[*c*,*h*][1,6]naphthyridine (**P58**)

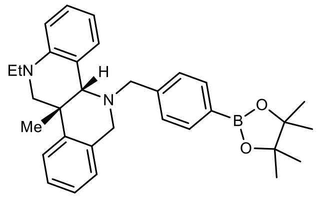

Yellow solid; m.p. : 76-78 ºC, (63.2 mg, 64% yield); ^{1}H NMR (400 MHz, $CDCl_3$) δ 7.41 (dd, *J* = 7.8, 1.2 Hz, 1H), 7.29 – 7.14 (m, 9H), 6.95 (d, *J* = 7.4 Hz, 1H), 6.69 (d, *J* = 8.2 Hz, 1H), 6.62 (td, *J* = 7.2, 1.1 Hz, 1H), 4.03 (d, *J* = 11.0 Hz, 1H), 3.95 (d, *J* = 13.2 Hz, 1H), 3.86 (d, *J* = 15.4 Hz, 1H), 3.68 – 3.53 (m, 2H), 3.41 – 3.27 (m, 2H), 3.16 (d, *J* = 13.2 Hz, 1H), 2.96 – 2.82 (m, 1H), 1.25 (s, 3H), 1.20 (t, *J* = 7.0 Hz, 3H). ^{13}C NMR (126 MHz, $CDCl_3$) δ 144.3, 143.6, 141.4, 134.9, 134.6, 133.9, 129.1, 127.8, 126.5, 126.3, 126.3, 126.1, 118.8, 114.3, 110.5, 83.7, 67.9, 58.1, 57.1, 55.7, 44.8, 36.6, 24.9, 22.6, 11.0. HRMS (ESI): Calcd. for $C_{32}H_{40}BN_2O_2$ $[M+H]^+$: 495.3177; found: 495.3183.

(59) (*cis*)-12-ethyl-10b-methyl-5-(naphthalen-2-ylmethyl)-4b,5,6,10b,11,12-hexahydrodibenzo[*c*,*h*][1,6]naphthyridine (**P59**)

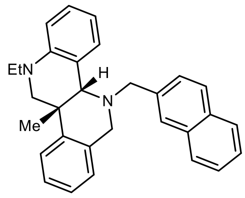

Brown solid; m.p. : 129-131 ºC, (72.7 mg, 87% yield); ^{1}H NMR (500 MHz, $CDCl_3$) δ 7.63 (dq, *J* = 8.8, 5.2, 4.2 Hz, 2H), 7.57 – 7.49 (m, 2H), 7.32 – 7.19 (m, 4H), 7.06 (tdd, *J* = 10.9, 5.2, 2.7 Hz, 3H), 6.98 (td, *J* = 7.6, 3.3 Hz, 1H), 6.74 (dd, *J* = 7.6, 3.2 Hz, 1H), 6.60 – 6.43 (m, 2H), 3.95 (tt, *J* = 9.0, 4.2 Hz, 2H), 3.73 (dt, *J* = 15.5, 4.4 Hz, 1H), 3.47 (ddd, *J* = 11.0, 7.6, 3.5 Hz, 2H), 3.31 – 3.24 (m, 1H), 3.20 – 3.06 (m, 2H), 2.86 – 2.65 (m, 1H), 1.17 – 1.11 (m, 3H), 1.08 (td, *J* = 7.1, 3.5 Hz, 3H). ^{13}C NMR (126 MHz, $CDCl_3$) δ 143.7, 141.5, 138.4, 135.0, 134.0, 133.4, 132.7, 129.3, 127.8, 127.7, 127.7, 127.0, 126.8, 126.5, 126.4, 126.4, 126.2, 125.9, 125.4, 118.9, 114.5, 110.6, 68.1, 58.3, 57.2, 55.8, 44.9, 36.7, 22.8, 11.1. HRMS (ESI): Calcd. for $C_{30}H_{31}N_2$ $[M+H]^+$: 419.2482; found: 419.2477.

(60) (*cis*)-12-ethyl-10b-methyl-5-(4-(trifluoromethyl)benzyl)-4b,5,6,10b,11,12-hexahydrodibenzo[*c*,*h*][1,6]naphthyridine (**P60**)

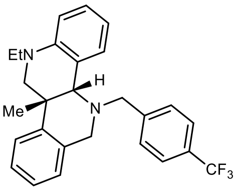

Brown solid; m.p. : 91-93 ºC, (74.1 mg, 85% yield); ^{1}H NMR (400 MHz, $CDCl_3$) δ 7.49 (dd, *J* = 18.8, 7.8 Hz, 3H), 7.38 (d, *J* = 7.8 Hz, 2H), 7.32 – 7.25 (m, 1H), 7.23 – 7.11 (m, 3H), 7.01 (d, *J* = 7.6 Hz, 1H), 6.68 (td, *J* = 9.4, 9.0, 6.8 Hz, 2H), 4.13 – 3.95 (m, 2H), 3.88 (dd, *J* = 15.2, 2.2 Hz, 1H), 3.71 (d, *J* = 15.2 Hz, 1H), 3.67 – 3.53 (m, 1H), 3.45 (s, 1H), 3.40 – 3.26 (m, 2H), 2.98 (dt, *J* = 11.2, 1.8 Hz, 1H), 1.30 (d, *J* = 2.0 Hz, 3H), 1.23 (td, *J* = 7.0, 2.0 Hz, 3H). ^{13}C NMR (101 MHz, $CDCl_3$) δ 145.1, 143.6, 141.3, 134.6, 133.8, 129.3, 128.5, 126.5, 126.4, 126.3, 125.0, 124.9, 118.4, 114.4, 110.4, 68.0, 57.5, 57.1, 56.1, 44.8, 36.6, 22.7, 11.1. ^{19}F NMR (376 MHz, $CDCl_3$) δ -62.20, -62.21, -62.23. HRMS (ESI): Calcd. for $C_{27}H_{28}F_3N_2$ $[M+H]^+$: 437.2199; found: 437.2198.

(61) (*cis*)-12-ethyl-10b-methyl-5-(4-(methylsulfonyl)benzyl)-4b,5,6,10b,11,12-hexahydrodibenzo [*c*,*h*][1,6]naphthyridine (**P61**)

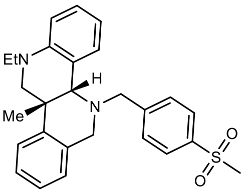

Brown solid; m.p. : 114-116 ºC, (63.3 mg, 71% yield); ^{1}H NMR (400 MHz, $CDCl_3$) δ 7.78 (d, *J* = 8.0 Hz, 2H), 7.43 (t, *J* = 8.7 Hz, 3H), 7.33 – 7.23 (m, 1H), 7.17 (dt, *J* = 14.2, 7.0 Hz, 3H), 7.00 (d, *J* = 7.6 Hz, 1H), 6.62 (t, *J* = 7.6 Hz, 2H), 4.02 (dd, *J* = 12.6, 6.3 Hz, 2H), 3.86 (d, *J* = 15.0 Hz, 1H), 3.75 (d, *J* = 15.2 Hz, 1H), 3.56 (dq, *J* = 14.2, 7.0 Hz, 1H), 3.46 – 3.27 (m, 3H), 3.03 (s, 3H), 2.96 (d, *J* = 11.2 Hz, 1H), 1.26 (s, 3H), 1.19 (t, *J* = 7.0 Hz, 3H). ^{13}C NMR (101 MHz, $CDCl_3$) δ 147.7, 143.6, 141.2, 138.4, 134.4, 133.7, 129.4, 129.0, 127.0, 126.6, 126.5, 126.4, 126.3, 118.3, 114.4, 110.2, 68.0, 57.5, 57.0, 56.4, 44.8, 44.6, 36.5, 22.6, 11.1. HRMS (ESI): Calcd. for $C_{27}H_{31}N_2O_2S$ $[M+H]^+$: 447.2101; found: 447.2099.

(62) (*cis*)-12-ethyl-5-hexyl-10b-methyl-4b,5,6,10b,11,12-hexahydrodibenzo[*c*,*h*][1,6]naphthyridine (**P62**)

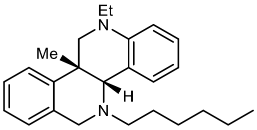

Yellow solid; m.p. : 77-79 ºC, (47.8 mg, 66% yield); ^{1}H NMR (500 MHz, $CDCl_3$) δ 7.27 (dt, *J* = 7.5, 2.1 Hz, 1H), 7.12 – 7.07 (m, 3H), 6.99 – 6.95 (m, 2H), 6.56 – 6.54 (m, 1H), 6.50 – 6.46 (m, 1H), 3.97 – 3.94 (m, 1H), 3.74 (dd, *J* = 11.1, 3.0 Hz, 1H), 3.50 (dd, *J* = 15.3, 2.7 Hz, 1H), 3.43 – 3.35 (m, 1H), 3.23 – 3.15 (m, 1H), 2.75 – 2.72 (m, 1H), 2.56 – 2.50 (m, 1H), 2.11 – 2.04 (m, 1H), 1.22 – 1.17 (m, 1H), 1.11 – 0.92 (m, 12H), 0.73 – 0.69 (m, 3H). ^{13}C NMR (126 MHz, $CDCl_3$) δ 143.7, 141.5, 135.3, 133.8, 128.8, 126.4, 126.3, 126.2, 126.1, 119.1, 114.1, 110.1, 68.1, 56.9, 55.6, 53.0, 44.8, 36.4, 31.6, 27.8, 26.7, 22.7, 22.7, 14.1, 11.0. HRMS (ESI): Calcd. for $C_{25}H_{35}N_2$ $[M+H]^+$: 363.5589; found: 363.5451.

(63) 1-(((*cis*)-12-ethyl-10b-methyl-6,10b,11,12-tetrahydrodibenzo[*c*,*h*][1,6]naphthyridin-5(4b*H*)-yl)methyl)-2-(((*cis*)-12-ethyl-10b-methyl-6,10b,11,12-tetrahydrodibenzo[*c*,*h*][1,6]naphthyridin-5 (4b*H*)-yl)methyl)benzene (**P63**)

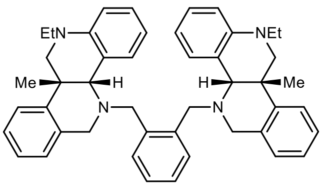

Yellow solid; m.p. : 133-135 ºC, (55.3 mg, 42% yield); ^{1}H NMR (500 MHz, $CDCl_3$) δ 7.27 (dd, *J* = 7.5, 3.6 Hz, 2H), 7.10 (t, *J* = 8.3 Hz, 2H), 7.06 – 6.89 (m, 10H), 6.74 (ddd, *J* = 17.1, 7.6, 2.6 Hz, 2H), 6.54 (dt, *J* = 7.6, 3.5 Hz, 2H), 6.47 (tt, *J* = 7.1, 3.3 Hz, 2H), 3.89 – 3.59 (m, 6H), 3.47 – 3.32 (m, 4H), 3.26 – 3.09 (m, 4H), 2.97 (td, *J* = 12.6, 3.4 Hz, 2H), 2.75 (ddd, *J* = 21.8, 11.2, 2.9 Hz, 2H), 1.11 (t, *J* = 3.7 Hz, 6H), 1.04 (td, *J* = 7.1, 2.7 Hz, 6H). ^{13}C NMR (126 MHz, $CDCl_3$) δ 143.7, 141.4, 141.4, 140.5, 140.3, 135.1, 135.1, 133.9, 133.8, 129.1, 128.6, 128.1, 127.9, 127.8, 126.5, 126.5, 126.4, 126.3, 126.2, 126.2, 126.1, 118.9, 114.3, 110.4, 110.4, 68.0, 68.0, 58.0, 58.0, 57.1, 55.7, 55.7, 44.8, 44.8, 36.6, 22.7, 22.7, 11.1. HRMS (ESI): Calcd. for $C_{46}H_{51}N_4$ $[M+H]^+$: 658.3887; found: 658.3896.

(64) (*cis*)-5-benzyl-3-methoxy-10b,12-dimethyl-4b,5,6,10b,11,12-hexahydrodibenzo[*c*,*h*][1,6]naphthyridine (**P64**)

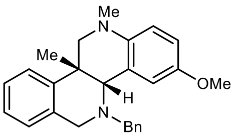

Brown solid; m.p. : 128-130 ºC, (49.9 mg, 65% yield); ^{1}H NMR (500 MHz, $CDCl_3$) δ 7.30 (d, *J* = 7.8 Hz, 1H), 7.16 – 7.07 (m, 6H), 7.03 (d, *J* = 7.5 Hz, 1H), 6.84 (d, *J* = 7.6 Hz, 1H), 6.78 (t, *J* = 2.3 Hz, 1H), 6.72 (dt, *J* = 9.0, 2.1 Hz, 1H), 6.53 (d, *J* = 8.8 Hz, 1H), 3.82 (d, *J* = 13.3 Hz, 1H), 3.72 (d, *J* = 12.3 Hz, 5H), 3.53 (d, *J* = 15.4 Hz, 1H), 3.37 (s, 1H), 3.20 (d, *J* = 13.3 Hz, 1H), 2.87 (s, 4H), 1.23 – 1.16 (m, 3H). ^{13}C NMR (126 MHz, $CDCl_3$) δ 150.3, 141.3, 140.7, 140.4, 134.8, 128.3, 128.1, 126.6, 126.5, 126.4, 126.1, 120.6, 118.8, 114.8, 112.0, 67.7, 60.0, 57.6, 56.0, 54.9, 39.3, 37.2, 23.6. HRMS (ESI): Calcd. for $C_{26}H_{29}N_2O$ $[M+H]^+$: 385.2274; found: 385.2271.

(65) (*cis*)-5-benzyl-12-ethyl-2,10b-dimethyl-4b,5,6,10b,11,12-hexahydrodibenzo[*c*,*h*][1,6]naphthyridine (**P65**)

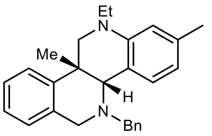

Brown solid; m.p. : 110-112 ºC, (61.1 mg, 80% yield); ^{1}H NMR (500 MHz, $CDCl_3$) δ 7.28 (dd, *J* = 7.9, 3.2 Hz, 1H), 7.11 (qq, *J* = 6.0, 3.5, 3.0 Hz, 5H), 7.06 – 6.97 (m, 2H), 6.92 (dd, *J* = 7.6, 3.8 Hz, 1H), 6.81 (dd, *J* = 7.7, 2.8 Hz, 1H), 6.44 – 6.29 (m, 2H), 3.87 (td, *J* = 13.8, 12.6, 4.0 Hz, 2H), 3.73 (dd, *J* = 15.4, 3.8 Hz, 1H), 3.46 (ddt, *J* = 16.0, 14.1, 5.2 Hz, 2H), 3.28 – 3.13 (m, 2H), 3.02 (dd, *J* = 13.4, 3.7 Hz, 1H), 2.77 (dt, *J* = 11.2, 2.4 Hz, 1H), 2.20 (d, *J* = 3.7 Hz, 3H), 1.13 (d, *J* = 3.8 Hz, 3H), 1.07 (dt, *J* = 7.0, 3.9 Hz, 3H). ^{13}C NMR (126 MHz, $CDCl_3$) δ 143.5, 141.6, 140.9, 138.8, 135.1, 133.8, 128.4, 128.1, 126.5, 126.5, 126.4, 126.3, 126.1, 116.1, 115.5, 111.1, 67.7, 58.0, 57.1, 55.6, 44.8, 36.7, 22.8, 21.9, 11.1. HRMS (ESI): Calcd. for $C_{27}H_{31}N_2$ $[M+H]^+$: 383.2482; found: 383.2481.

(66) (*cis*)-5-benzyl-3-(tert-butyl)-12-ethyl-10b-methyl-4b,5,6,10b,11,12-hexahydrodibenzo[*c*,*h*][1,6]naphthyridine (**P66**)

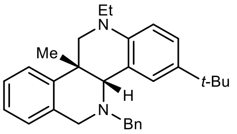

Brown solid; m.p. : 131-133 ºC, (67.8 mg, 80% yield); ^{1}H NMR (400 MHz, $CDCl_3$) δ 7.45 (d, *J* = 7.8 Hz, 1H), 7.34 – 7.15 (m, 9H), 6.99 (d, *J* = 7.4 Hz, 1H), 6.73 – 6.59 (m, 1H), 4.03 – 3.86 (m, 3H), 3.72 – 3.57 (m, 2H), 3.46 (s, 1H), 3.33 (dq, *J* = 14.4, 7.0 Hz, 1H), 3.25 (dd, *J* = 13.2, 1.6 Hz, 1H), 2.94 (d, *J* = 11.0 Hz, 1H), 1.38 (d, *J* = 1.8 Hz, 9H), 1.31 (d, *J* = 1.6 Hz, 3H), 1.22 (td, *J* = 7.0, 1.4 Hz, 3H). ^{13}C NMR (101 MHz, $CDCl_3$) δ 141.7, 141.4, 141.0, 137.1, 135.0, 130.7, 128.4, 128.1, 126.5, 126.5, 126.4, 126.3, 126.1, 125.7, 118.5, 110.1, 68.1, 57.6, 57.1, 55.4, 44.9, 36.8, 33.6, 31.7, 23.2, 11.1. HRMS (ESI): Calcd. for $C_{30}H_{37}N_2$ $[M+H]^+$: 425.2951; found: 425.2950.

(67) (*cis*)-5-benzyl-3-bromo-12-ethyl-10b-methyl-4b,5,6,10b,11,12-hexahydrodibenzo[*c*,*h*][1,6]naphthyridine (**P67**)

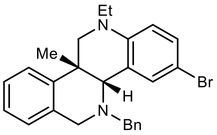

Brown solid; m.p. : 95-97 ºC, (49.1 mg, 55% yield); ^{1}H NMR (400 MHz, $CDCl_3$) δ 7.29 (dd, *J* = 7.8, 1.2 Hz, 1H), 7.16 – 6.98 (m, 9H), 6.83 (d, *J* = 7.4 Hz, 1H), 6.58 (d, *J* = 8.2 Hz, 1H), 6.53 – 6.46 (m, 1H), 3.91 (d, *J* = 11.2 Hz, 1H), 3.84 (d, *J* = 13.4 Hz, 1H), 3.75 (d, *J* = 15.4 Hz, 1H), 3.48 (dq, *J* = 14.2, 6.7 Hz, 2H), 3.28 (d, *J* = 1.4 Hz, 1H), 3.20 (dt, *J* = 14.2, 7.0 Hz, 1H), 3.05 (d, *J* = 13.2 Hz, 1H), 2.80 (dd, *J* = 11.2, 1.3 Hz, 1H), 1.14 (s, 3H), 1.08 (t, *J* = 7.0 Hz, 3H). ^{13}C NMR (101 MHz, $CDCl_3$) δ 143.7, 141.4, 140.8, 135.0, 133.9, 129.1, 128.3, 128.1, 126.5, 126.5, 126.4, 126.3, 126.1, 118.8, 114.3, 110.5, 68.0, 58.0, 57.1, 55.6, 44.8, 36.6, 22.7, 11.0. HRMS (ESI): Calcd. for $C_{26}H_{28}BrN_2$ $[M+H]^+$: 447.1430; found: 447.1429.

(68) (*cis*)-5-benzyl-3-chloro-12-ethyl-10b-methyl-4b,5,6,10b,11,12-hexahydrodibenzo[*c*,*h*][1,6]naphthyridine (**P68**)

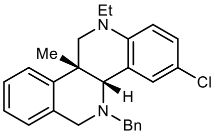

Brown solid; m.p. : 85-87 ºC, (50.6 mg, 63% yield); ^{1}H NMR (500 MHz, $CDCl_3$) δ 7.28 (d, *J* = 7.6 Hz, 1H), 7.19 – 7.04 (m, 6H), 7.05 – 6.98 (m, 1H), 6.83 (td, *J* = 8.1, 7.1, 2.7 Hz, 2H), 6.77 (ddt, *J* = 11.6, 5.4, 2.8 Hz, 1H), 6.46 (dd, *J* = 9.1, 4.4 Hz, 1H), 3.89 – 3.80 (m, 2H), 3.74 (dd, *J* = 15.5, 3.6 Hz, 1H), 3.54 – 3.36 (m, 2H), 3.25 (d, *J* = 3.5 Hz, 1H), 3.20 – 3.05 (m, 2H), 2.83 – 2.64 (m, 1H), 1.15 – 1.10 (m, 3H), 1.05 (ddt, *J* = 7.1, 4.7, 2.3 Hz, 3H). ^{13}C NMR (126 MHz, $CDCl_3$) δ 141.1, 140.4, 140.3, 134.8, 128.3, 128.2, 126.7, 126.5, 126.4, 126.3, 126.2, 120.0, 120.0, 119.5, 119.3, 115.7, 115.5, 111.2, 111.2, 67.8, 57.9, 57.0, 55.3, 45.2, 36.7, 23.0, 10.8. HRMS (ESI): Calcd. for $C_{26}H_{28}ClN_2$ $[M+H]^+$: 403.1936; found: 403.1935.

(69) (*cis*)-5-benzyl-12-ethyl-3-fluoro-10b-methyl-4b,5,6,10b,11,12-hexahydrodibenzo[*c*,*h*][1,6]naphthyridine (**P69**)

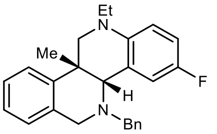

Brown solid; m.p. : 83-85 ºC, (48.6 mg, 63% yield); ^{1}H NMR (500 MHz, $CDCl_3$) δ 7.27 (dd, *J* = 7.9, 3.3 Hz, 1H), 7.08 (dt, *J* = 44.1, 4.0 Hz, 8H), 6.98 (ddd, *J* = 9.0, 4.3, 2.4 Hz, 1H), 6.83 (dd, *J* = 7.6, 2.6 Hz, 1H), 6.46 (dd, *J* = 8.9, 3.2 Hz, 1H), 3.88 (dd, *J* = 11.2, 3.7 Hz, 1H), 3.82 (dd, *J* = 13.3, 3.8 Hz, 1H), 3.75 (dd, *J* = 15.5, 3.7 Hz, 1H), 3.53 – 3.35 (m, 2H), 3.23 – 3.13 (m, 2H), 3.09 (dd, *J* = 13.2, 3.7 Hz, 1H), 2.78 (dq, *J* = 8.9, 3.1, 2.4 Hz, 1H), 1.11 (d, *J* = 3.6 Hz, 3H), 1.06 (td, *J* = 7.0, 3.3 Hz, 3H). ^{13}C NMR (126 MHz, $CDCl_3$) δ 142.3, 140.9, 140.4, 134.8, 132.9, 128.8, 128.4, 128.1, 126.6, 126.5, 126.4, 126.3, 120.4, 118.7, 111.6, 67.7, 58.0, 57.0, 55.7, 45.0, 36.5, 22.7, 10.9. ^{19}F NMR (471 MHz, $CDCl_3$) δ -131.5. HRMS (ESI): Calcd. for $C_{26}H_{28}FN_2$ $[M+H]^+$: 387.2231; found: 387.2230.

(70) (*cis*)-5-benzyl-12-isopropyl-10b-methyl-4b,5,6,10b,11,12-hexahydrodibenzo[*c*,*h*][1,6]naphthyridine (**P70**)

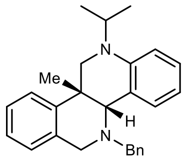

Brown solid; m.p. : 111-113 ºC, (58.8 mg, 77% yield); ^{1}H NMR (400 MHz, $CDCl_3$) δ 7.48 (dd, *J* = 7.8, 1.2 Hz, 1H), 7.35 – 7.13 (m, 9H), 7.01 (d, *J* = 7.4 Hz, 1H), 6.83 (d, *J* = 8.2 Hz, 1H), 6.67 (t, *J* = 7.4 Hz, 1H), 4.32 (p, *J* = 6.6 Hz, 1H), 4.04 (d, *J* = 13.4 Hz, 1H), 3.93 (d, *J* = 15.2 Hz, 1H), 3.80 (d, *J* = 11.2 Hz, 1H), 3.64 (d, *J* = 15.4 Hz, 1H), 3.44 (d, *J* = 1.4 Hz, 1H), 3.23 (d, *J* = 13.2 Hz, 1H), 3.04 (dd, *J* = 11.2, 1.4 Hz, 1H), 1.34 (d, *J* = 6.6 Hz, 3H), 1.30 – 1.25 (m, 6H). ^{13}C NMR (101 MHz, $CDCl_3$) δ 144.3, 141.8, 140.8, 135.1, 134.0, 129.2, 128.4, 128.1, 126.5, 126.5, 126.5, 126.3, 126.2, 119.2, 114.2, 110.5, 68.1, 58.0, 55.6, 49.1, 46.5, 36.2, 22.6, 19.6, 19.0. HRMS (ESI): Calcd. for $C_{27}H_{31}N_2$ $[M+H]^+$: 383.2482; found: 383.2480.

(71) (*cis*)-5-benzyl-12-cyclohexyl-10b-methyl-4b,5,6,10b,11,12-hexahydrodibenzo[*c*,*h*][1,6]naphthyridinee (**P71**)

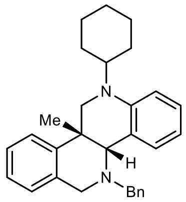

Brown solid; m.p. : 126-128 ºC, (65.0 mg, 77% yield); ^{1}H NMR (500 MHz, $CDCl_3$) δ 7.29 (dd, *J* = 8.0, 2.8 Hz, 1H), 7.16 – 6.98 (m, 9H), 6.82 (dd, *J* = 7.7, 2.5 Hz, 1H), 6.63 (dd, *J* = 8.5, 3.1 Hz, 1H), 6.47 (dt, *J* = 8.0, 4.5 Hz, 1H), 3.91 – 3.82 (m, 1H), 3.78 – 3.69 (m, 1H), 3.67 – 3.57 (m, 2H), 3.45 (dd, *J* = 15.4, 3.1 Hz, 1H), 3.25 (d, *J* = 3.1 Hz, 1H), 3.10 – 3.00 (m, 1H), 2.91 (dt, *J* = 11.2, 2.3 Hz, 1H), 1.84 – 1.70 (m, 4H), 1.64 – 1.58 (m, 1H), 1.50 – 1.27 (m, 4H), 1.08 (d, *J* = 3.0 Hz, 3H), 1.04 (d, *J* = 3.5 Hz, 1H). ^{13}C NMR (126 MHz, $CDCl_3$) δ 144.2, 141.8, 140.9, 135.1, 134.0, 129.1, 128.4, 128.1, 126.5, 126.5, 126.4, 126.3, 126.1, 119.1, 114.0, 110.4, 68.1, 58.1, 55.8, 55.6, 50.6, 36.3, 30.0, 29.7, 26.4, 26.3, 26.1, 22.6. HRMS (ESI): Calcd. for $C_{30}H_{35}N_2$ $[M+H]^+$: 423.2795; found: 423.2794.

(72) (*cis*)-12-allyl-5-benzyl-10b-methyl-4b,5,6,10b,11,12-hexahydrodibenzo[*c*,*h*][1,6]naphthyridine (**P72**)

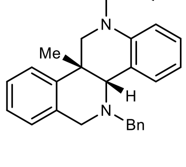

Yellow solid; m.p. : 89-91 ºC, (60.8 mg, 80% yield); ^{1}H NMR (400 MHz, $CDCl_3$) δ 7.42 (d, *J* = 7.8 Hz, 1H), 7.30 – 7.13 (m, 9H), 6.97 (d, *J* = 7.6 Hz, 1H), 6.66 (dt, *J* = 7.4, 3.2 Hz, 2H), 5.89 (ddt, *J* = 17.2, 9.8, 4.8 Hz, 1H), 5.35 – 5.09 (m, 2H), 4.14 – 3.82 (m, 5H), 3.61 (d, *J* = 15.4 Hz, 1H), 3.44 (s, 1H), 3.21 (d, *J* = 13.4 Hz, 1H), 2.98 (dd, *J* = 11.2, 1.6 Hz, 1H), 1.30 (s, 3H). ^{13}C NMR (101 MHz, $CDCl_3$) δ 144.1, 141.3, 140.7, 134.9, 133.7, 133.2, 129.1, 128.3, 128.1, 126.5, 126.4, 126.4, 126.3, 126.1, 118.7, 116.0, 114.8, 111.0, 68.0, 58.0, 57.8, 55.7, 53.3, 36.9, 36.6, 22.9. HRMS (ESI): Calcd. for $C_{27}H_{29}N_2$ $[M+H]^+$: 381.2325; found: 381.2323.

(73) (*cis*)-6,13-dibenzyl-14a-methyl-5,6,6a,13,14,14a-hexahydrobenzo[*c*]naphtho[1,2-*h*][1,6]naphthyridine (**P73**)

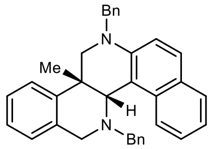

Brown solid; m.p. : 123-125 ºC, (73.9 mg, 77% yield); ^{1}H NMR (500 MHz, $CDCl_3$) δ 7.94 (dd, *J* = 8.6, 5.7 Hz, 1H), 7.56 (dd, *J* = 8.2, 4.6 Hz, 1H), 7.48 (dd, *J* = 9.1, 4.3 Hz, 1H), 7.41 – 7.34 (m, 1H), 7.26 (dd, *J* = 8.1, 4.0 Hz, 1H), 7.23 – 6.93 (m, 14H), 6.87 (dd, *J* = 7.8, 3.8 Hz, 1H), 4.74 (dd, *J* = 17.4, 4.1 Hz, 1H), 4.52 (dd, *J* = 17.3, 3.9 Hz, 1H), 4.26 (d, *J* = 5.1 Hz, 1H), 4.09 (dd, *J* = 11.4, 5.2 Hz, 1H), 3.92 – 3.78 (m, 2H), 3.63 (dd, *J* = 13.8, 5.7 Hz, 1H), 3.31 (dd, *J* = 13.7, 5.4 Hz, 1H), 3.07 – 2.96 (m, 1H), 1.19 (d, *J* = 4.9 Hz, 3H).^{13}C NMR (126 MHz, $CDCl_3$) δ 142.3, 141.8, 141.2, 138.8, 135.7, 135.1, 129.7, 128.8, 128.7, 128.2, 128.1, 127.1, 127.0, 126.7, 126.7, 126.6, 126.5, 126.4, 126.3, 122.3, 121.2, 114.8, 108.9, 61.0, 58.0, 56.3, 55.2, 55.2, 36.6, 23.2. HRMS (ESI): Calcd. for $C_{35}H_{33}N_2$ $[M+H]^+$: 481.2638; found: 481.2637.

(74) ethyl2-((*cis*)-5-benzyl-10b-methyl-5,6,10b,11-tetrahydrodibenzo[*c*,*h*][1,6]naphthyridin-12(4b*H*)-yl)acetate (**P74**)

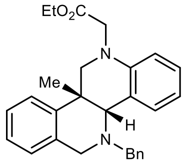

Yellow solid; m.p. : 131-133 ºC, (34.9 mg, 41% yield); ^{1}H NMR (500 MHz, $CDCl_3$) δ 7.37 (dd, *J* = 7.9, 1.3 Hz, 1H), 7.28 – 7.10 (m, 9H), 6.92 (d, *J* = 7.6 Hz, 1H), 6.67 (td, *J* = 7.3, 1.1 Hz, 1H), 6.47 (d, *J* = 8.2 Hz, 1H), 4.23 – 4.07 (m, 4H), 3.97 (d, *J* = 17.9 Hz, 1H), 3.90 (d, *J* = 13.3 Hz, 1H), 3.83 (d, *J* = 15.4 Hz, 1H), 3.58 (d, *J* = 15.4 Hz, 1H), 3.45 (s, 1H), 3.20 (d, *J* = 13.3 Hz, 1H), 3.01 (dd, *J* = 10.8, 1.5 Hz, 1H), 1.27 (s, 3H), 1.21 (td, *J* = 7.1, 1.4 Hz, 3H).^{13}C NMR (126 MHz, $CDCl_3$) δ 170.9, 143.6, 141.0, 140.7, 134.9, 133.7, 129.2, 128.4, 128.0, 126.5, 126.3, 126.3, 126.2, 119.2, 115.8, 110.0, 67.7, 60.9, 58.9, 57.8, 55.4, 52.8, 36.9, 23.0, 14.3. HRMS (ESI): Calcd. for $C_{28}H_{31}N_2O_2$ $[M+H]^+$: 427.2380; found: 427.2376.

(75) (*cis*)-5-benzyl-10b-methyl-12-phenyl-4b,5,6,10b,11,12-hexahydrodibenzo[*c*,*h*][1,6]naphthyridine (**P75**)

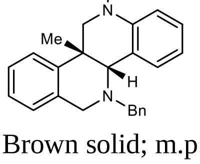

Brown solid; m.p. : 107-109 ºC, (41.6 mg, 50% yield); ^{1}H NMR (500 MHz, $CDCl_3$) δ 7.35 (dt, *J* = 15.5, 7.8 Hz, 3H), 7.29 – 7.21 (m, 7H), 7.20 – 7.13 (m, 3H), 7.10 (td, *J* = 7.3, 1.2 Hz, 1H), 7.00

(ddd, J = 8.7, 7.2, 1.5 Hz, 1H), 6.92 (d, J = 7.5 Hz, 1H), 6.71 (t, J = 7.4 Hz, 1H), 6.67 (d, J = 8.3 Hz, 1H), 4.21 (d, J = 11.4 Hz, 1H), 4.03 (d, J = 13.3 Hz, 1H), 3.86 (d, J = 15.4 Hz, 1H), 3.64 (d, J = 15.4 Hz, 1H), 3.56 (s, 1H), 3.40 (dd, J = 11.4, 1.4 Hz, 1H), 3.32 (d, J = 13.3 Hz, 1H), 1.36 (s, 3H). ^{13}C NMR (126 MHz, $CDCl_3$) δ 147.8, 143.8, 140.8, 140.7, 133.5, 129.6, 128.4, 128.2, 126.6, 126.5, 126.4, 126.4, 126.3, 126.2, 124.8, 120.2, 116.8, 114.5, 67.5, 59.7, 57.8, 55.0, 37.6, 23.2. HRMS (ESI): Calcd. for $C_{30}H_{29}N_2$ $[M+H]^+$: 417.2325; found: 417.2324.

(76) (*cis*)-5-benzyl-12-(4-methoxyphenyl)-10b-methyl-4b,5,6,10b,11,12-hexahydrodibenzo[*c*,*h*][1,6]naphthyridine (**P76**)

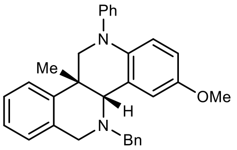

Brown solid; m.p. : 93-95 ºC, (44.6 mg, 50% yield); ^{1}H NMR (500 MHz, $CDCl_3$) δ 7.25 (d, J = 7.8 Hz, 1H), 7.21 (d, J = 7.6 Hz, 2H), 7.18 – 7.13 (m, 3H), 7.12 – 7.04 (m, 4H), 7.01 (dd, J = 8.0, 6.8 Hz, 1H), 6.92 – 6.82 (m, 4H), 6.61 – 6.54 (m, 1H), 6.42 – 6.38 (m, 1H), 4.21 – 4.06 (m, 1H), 3.96 (d, J = 13.4 Hz, 1H), 3.78 (d, J = 15.5 Hz, 1H), 3.74 (s, 3H), 3.55 (d, J = 15.4 Hz, 1H), 3.45 (s, 1H), 3.27 – 3.14 (m, 2H), 1.29 (d, J = 1.1 Hz, 3H). ^{13}C NMR (126 MHz, $CDCl_3$) δ 157.3, 144.6, 140.9, 140.8, 140.5, 134.9, 133.6, 128.5, 128.5, 128.3, 128.2, 126.6, 126.5, 126.4, 126.3, 126.1, 119.2, 116.0, 115.0, 113.7, 67.6, 60.1, 57.9, 55.5, 55.3, 37.3, 23.0. HRMS (ESI): Calcd. for $C_{31}H_{31}N_2O$ $[M+H]^+$: 447.2431; found: 447.2430.

(77) (*cis,cis*)-16,19-dibenzyl-4b,10a-dimethyl-4b,5,7,8,10,10a,15,16,16a,18b,19,20-dodecahydrodiisoquinolino[4,3-*c*:3',4'-*i*]pyrazino[1,2,3,4-*lmn*][1,10]phenanthroline (**P77**)

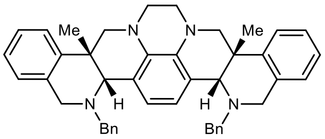

Brown solid; m.p. : 116-118 ºC, (51.5 mg, 41% yield); ^{1}H NMR (500 MHz, $CDCl_3$) δ 7.28 (dd, J = 7.9, 2.9 Hz, 2H), 7.14 (ddt, J = 28.0, 22.9, 6.9 Hz, 12H), 7.05 – 6.98 (m, 2H), 6.83 (d, J = 7.7 Hz, 2H), 6.48 (d, J = 3.7 Hz, 2H), 3.90 (dd, J = 13.6, 3.5 Hz, 2H), 3.80 – 3.63 (m, 4H), 3.57 – 3.38 (m, 4H), 3.27 (d, J = 3.4 Hz, 2H), 3.24 – 3.06 (m, 4H), 2.74 (d, J = 10.8 Hz, 2H), 1.17 (d, J = 3.5 Hz, 6H). ^{13}C NMR (126 MHz, $CDCl_3$) δ 141.73, 141.19, 135.12, 130.42, 128.39, 128.07, 126.53, 126.47, 126.35, 125.98, 121.17, 116.61, 67.17, 58.59, 57.38, 55.40, 47.32, 36.72, 23.55. HRMS (ESI): Calcd. for $C_{44}H_{45}N_4$ $[M+H]^+$: 629.3639; found: 629.3639.

(78) (*cis*)-6-benzyl-14a-methyl-6,6a,11,12,14,14a-hexahydro-5*H*,10*H*-benzo[*c*]quinolino[1,8-*gh*][1,6]naphthyridine (**P78**)

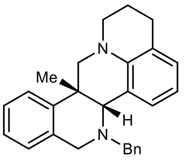

Brown solid; m.p. : 121-123 ºC, (64.6 mg, 85% yield); ^{1}H NMR (400 MHz, $CDCl_3$) δ 7.44 (dd, J = 7.8, 1.2 Hz, 1H), 7.28 (dd, J = 8.0, 2.8 Hz, 5H), 7.22 – 7.13 (m, 2H), 7.06 (dd, J = 7.6, 1.4 Hz, 1H), 6.98 (d, J = 7.6 Hz, 1H), 6.91 (dd, J = 7.2, 1.6 Hz, 1H), 6.56 (t, J = 7.4 Hz, 1H), 3.99 (d, J = 13.4 Hz, 1H), 3.95 – 3.82 (m, 2H), 3.66 (d, J = 15.4 Hz, 1H), 3.46 (d, J = 1.4 Hz, 1H), 3.39 – 3.17 (m, 3H), 2.91 (dd, J = 11.2, 1.6 Hz, 1H), 2.86 – 2.70 (m, 2H), 2.14 – 2.05 (m, 1H), 2.01 – 1.88 (m, 1H),

1.31 (s, 3H). ^{13}C NMR (101 MHz, $CDCl_3$) δ 141.6, 141.4, 141.0, 135.1, 131.4, 128.4, 128.3, 128.0, 126.5, 126.4, 126.3, 126.0, 121.2, 117.6, 114.1, 67.8, 58.6, 57.7, 55.5, 49.7, 36.6, 28.0, 23.2, 21.8. HRMS (ESI): Calcd. for $C_{27}H_{29}N_2$ $[M+H]^+$: 381.2325; found: 381.2324.

(79) (*cis*)-6-benzyl-15a-methyl-6,6a,10,11,12,13,15,15a-octahydro-5*H*-azepino[3,2,1-*ij*]isoquinolino[4,3-*c*]quinoline (**P79**)

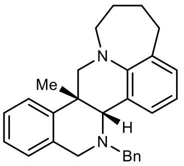

Black solid; m.p. : 140-142 ºC, (48.9 mg, 62% yield); ^1H NMR (400 MHz, $CDCl_3$) δ 7.41 (d, *J* = 8.0 Hz, 1H), 7.29 – 7.20 (m, 6H), 7.15 (td, *J* = 7.4, 1.2 Hz, 1H), 7.09 (dd, *J* = 7.4, 1.6 Hz, 1H), 7.02 (dd, *J* = 7.4, 1.6 Hz, 1H), 6.96 (d, *J* = 7.6 Hz, 1H), 6.72 (t, *J* = 7.4 Hz, 1H), 4.04 (d, *J* = 13.2 Hz, 1H), 3.91 – 3.76 (m, 2H), 3.60 (d, *J* = 15.6 Hz, 1H), 3.47 (d, *J* = 1.6 Hz, 1H), 3.34 – 3.11 (m, 4H), 2.84 –2.70 (m, 2H), 1.87 – 1.73 (m, 3H), 1.65 – 1.59 (m, 1H), 1.21 (s, 3H). ^{13}C NMR (101 MHz, $CDCl_3$) δ 147.0, 141.5, 140.8, 134.8, 131.7, 131.2, 130.8, 128.4, 128.1, 126.6, 126.5, 126.4, 126.3, 126.0, 123.3, 117.7, 68.3, 62.2, 58.0, 56.6, 55.3, 38.4, 35.2, 29.3, 25.6, 23.1. HRMS (ESI): Calcd. for $C_{28}H_{31}N_2$ $[M+H]^+$: 395.2482; found: 395.2480.

(80) (*cis,cis*)-5,5'-dibenzyl-12-isopropyl-10b,10'b-dimethyl-4b,4'b,5,5',6,10b,10'b,11,11',12-decahydro-6'*H*-3,12'-bidibenzo[*c*,*h*][1,6]naphthyridine (**P80**)

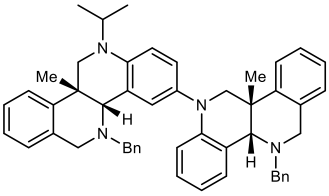

Brown solid; m.p. : 118-120 ºC, (87.8 mg, 62% yield); ^1H NMR (500 MHz, $CDCl_3$) δ 7.40 (dd, *J* = 8.0, 2.4 Hz, 1H), 7.37 – 7.30 (m, 3H), 7.28 – 7.13 (m, 12H), 7.10 – 7.01 (m, 3H), 6.93 (p, *J* = 8.0, 7.4 Hz, 3H), 6.76 (dd, *J* = 7.9, 3.9 Hz, 1H), 6.62 (t, *J* = 7.5 Hz, 1H), 6.48 (td, *J* = 7.6, 2.9 Hz, 1H), 4.26 – 4.17 (m, 2H), 4.12 – 3.94 (m, 2H), 3.90 – 3.77 (m, 2H), 3.74 – 3.61 (m, 2H), 3.59 – 3.49 (m, 2H), 3.39 – 3.25 (m, 3H), 3.20 (ddd, *J* = 12.9, 9.6, 2.8 Hz, 1H), 2.98 (d, *J* = 11.3 Hz, 1H), 1.39 (d, *J* = 2.8 Hz, 3H), 1.28 (dd, *J* = 6.4, 2.9 Hz, 3H), 1.21 (q, *J* = 2.9 Hz, 6H). ^{13}C NMR (126 MHz, $CDCl_3$) δ 145.3, 142.3, 141.7, 141.1, 141.0, 140.9, 140.8, 140.7, 135.0, 134.4, 134.3, 133.5, 132.8, 132.7, 128.4, 128.4, 128.4, 128.3, 128.2, 128.1, 126.6, 126.6, 126.5, 126.4, 126.3, 126.3, 126.3, 126.2, 126.1, 120.4, 118.8, 118.6, 115.4, 115.3, 113.7, 113.5, 111.4, 111.4, 67.9, 67.8, 67.7, 60.4, 60.1, 57.9, 57.7, 57.5, 55.4, 55.4, 55.3, 49.0, 46.7, 37.3, 37.2, 36.2, 23.2, 23.2, 22.8, 19.7, 18.8. HRMS (ESI): Calcd. for $C_{51}H_{53}N_4$ $[M+H]^+$: 721.4265; found: 721.4264.

(81) (1S,2R,4S)-1,7,7-trimethylbicyclo[2.2.1]heptan-2-yl-(6a-*cis*)-12-(3-chloro-4-methoxybenzyl)-5-ethyl-5,6,6a,7,12,12a-hexahydrodibenzo[*b*,*h*][1,6]naphthyridine-9-carboxylate (**P81**)

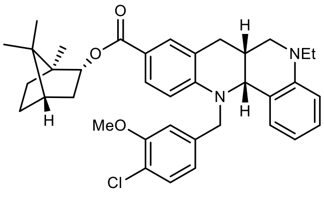

Brown solid; m.p. : 101-103 ºC, (64.6 mg, 54% yield); ^{1}H NMR (500 MHz, $CDCl_3$) δ 7.70 (dd, *J* = 8.6, 2.2 Hz, 1H), 7.66 (d, *J* = 2.0 Hz, 1H), 7.14 (d, *J* = 2.2 Hz, 1H), 7.10 – 7.04 (m, 1H), 7.02 (d, *J* = 8.2 Hz, 1H), 6.90 (d, *J* = 7.8 Hz, 1H), 6.80 (d, *J* = 8.4 Hz, 1H), 6.58 – 6.48 (m, 3H), 5.03 (d, *J* = 15.2 Hz, 1H), 4.90 (d, *J* = 17.0 Hz, 1H), 4.55 (d, *J* = 3.4 Hz, 1H), 4.39 (d, *J* = 17.0 Hz, 1H), 3.84 (s, 3H), 3.54 – 3.48 (m, 1H), 3.40 – 3.28 (m, *J* = 7.3 Hz, 2H), 3.23 (dd, *J* = 11.7, 6.4 Hz, 1H), 2.94 (dd, *J* = 16.2, 4.8 Hz, 1H), 2.74 (dd, *J* = 16.4, 8.2 Hz, 1H), 2.60 – 2.52 (m, 1H), 2.45 – 2.35 (m, 1H), 2.13 – 2.05 (m, 1H), 1.80 – 1.73 (m, 1H), 1.68 (t, *J* = 4.6 Hz, 1H), 1.39 – 1.22 (m, 2H), 1.17 (t, *J* = 7.0 Hz, 3H), 1.09 – 1.01 (m, 1H), 0.93 (s, 3H), 0.88 (s, 3H), 0.86 (s, 3H). ^{13}C NMR (126 MHz, $CDCl_3$) δ 167.1, 153.9, 147.2, 143.7, 131.6, 131.2, 129.5, 129.1, 128.1, 125.6, 122.7, 120.8, 120.1, 118.5, 115.2, 112.2, 111.1, 110.4, 79.5, 58.9, 56.2, 52.7, 49.9, 49.0, 47.8, 45.1, 44.6, 37.0, 29.9, 29.7, 29.6, 28.1, 27.5, 19.8, 19.0, 13.6, 11.0. HRMS (ESI): Calcd. for $C_{37}H_{44}ClN_2O_3$ $[M+H]^+$: 599.3035; found: 599.3028.

(82) (8R,9S,13S,17S)-3-(benzoyloxy)-13-methyl-7,8,9,11,12,13,14,15,16,17-decahydro-6H-cyclopenta[a]phenanthren-17-yl (6a-cis)-12-benzyl-5-ethyl-5,6,6a,7,12,12a-hexahydrodibenzo[*b*,*h*][1,6]naphthyridine-8-carboxylate (**P82**)

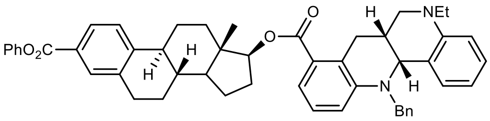

Yellow solid; m.p. : 105-107 ºC, (66.6 mg, 44% yield); ^{1}H NMR (400 MHz, $CDCl_3$) δ 8.25 (d, *J* = 7.8 Hz, 2H), 7.77 – 7.65 (m, 3H), 7.55 (t, *J* = 8.0 Hz, 2H), 7.39 – 7.32 (m, 5H), 7.27 – 7.24 (m, 1H), 7.15 (t, *J* = 8.0 Hz, 1H), 7.04 – 6.98 (m, 3H), 6.69 – 6.54 (m, 3H), 5.10 (d, *J* = 16.0 Hz, 1H), 4.99 – 4.90 (m, 1H), 4.66 – 4.54 (m, 2H), 3.62 – 3.55 (m, 1H), 3.40 (q, *J* = 8.0 Hz, 2H), 3.32 – 3.24 (m, 1H), 3.04 – 2.87 (m, 3H), 2.85 – 2.74 (m, 1H), 2.68 – 2.59 (m, 1H), 2.40 – 2.23 (m, 3H), 1.99 (t, *J* = 11.4 Hz, 2H), 1.88 – 1.79 (m, 1H), 1.75 – 1.33 (m, 7H), 1.23 (t, *J* = 7.2 Hz, 3H), 0.99 (s, 3H). ^{13}C NMR (101 MHz, $CDCl_3$) δ 166.9, 165.5, 148.7, 147.4, 143.5, 138.3, 138.2, 138.1, 133.5, 131.3, 130.2, 129.8, 129.5, 128.9, 128.7, 128.6, 128.3, 127.0, 126.6, 126.4, 121.7, 121.1, 120.1, 118.7, 118.0, 115.4, 111.1, 110.4, 82.3, 59.0, 53.9, 50.3, 49.9, 44.7, 44.1, 43.3, 38.3, 37.0, 29.8, 29.6, 29.5, 27.9, 27.1, 26.2, 23.5, 12.5, 11.1. HRMS (ESI): Calcd. for $C_{51}H_{53}N_2O_4$ $[M+H]^+$: 757.4000; found: 757.3983.

(83) (1S,4R)-N-((6a-cis)-12-benzyl-5-ethyl-5,6,6a,7,12,12a-hexahydrodibenzo[*b*,*h*][1,6]naphthyridin-9-yl)-4,7,7-trimethyl-3-oxo-2-oxabicyclo[2.2.1]heptane-1-carboxamide (**P83**)

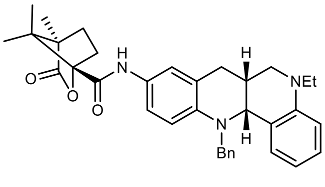

Yellow solid; m.p. : 88-90 ºC, (85.7 mg, 78% yield); ^{1}H NMR (500 MHz, $CDCl_3$) δ 7.89 (s, 1H), 7.30 – 7.14 (m, 6H), 7.09 – 7.01 (m, 2H), 6.93 (d, *J* = 9.6 Hz, 1H), 6.57 – 6.44 (m, 3H), 4.91 (d, *J* = 17.2 Hz, 1H), 4.49 (d, *J* = 3.4 Hz, 1H), 4.42 (d, *J* = 17.2 Hz, 1H), 3.56 – 3.41 (m, 1H), 3.37 – 3.30 (m, 2H), 3.25 (dd, *J* = 11.6, 6.0 Hz, 1H), 2.92 – 2.81 (m, 1H), 2.72 – 2.64 (m, 1H), 2.61 – 2.48 (m, 2H), 2.01 – 1.86 (m, 2H), 1.74 – 1.61 (m, 1H), 1.17 – 1.11 (m, 9H), 0.93 (s, 3H) ^{13}C NMR (126 MHz, $CDCl_3$) δ 178.2, 164.6, 143.7, 141.2, 139.2, 128.7, 128.5, 126.7, 126.6, 126.2, 122.1, 121.6,

121.4, 119.7, 115.2, 112.5, 110.3, 92.6, 58.5, 55.5, 54.2, 54.0, 50.4, 44.6, 30.5, 29.8, 29.6, 29.2, 16.8, 16.6, 11.1, 9.8. HRMS (ESI): Calcd. for $C_{35}H_{40}N_3O_3$ $[M+H]^+$: 550.3064; found: 550.3058.

(84) (6a-*cis*)-5,6,6a,7,12,12a-hexahydrodibenzo[*b*,*h*][1,6]naphthyridine (**P84**)

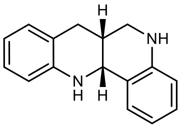

Yellow solid; m.p. : 128-130 ºC, (35.4 mg, 75% yield); ^{1}H NMR (400 MHz, $CDCl_3$) δ 7.21 – 7.12 (m, 2H), 7.07 – 7.00 (m, 2H), 6.74 – 6.66 (m, 2H), 6.59 (d, *J* = 8.0 Hz, 1H), 6.47 (d, *J* = 8.2 Hz, 1H), 4.41 (d, *J* = 3.0 Hz, 1H), 4.02 (s, 1H), 3.86 (s, 1H), 3.37 (t, *J* = 11.4 Hz, 1H), 3.24 (dd, *J* = 16.8, 6.2 Hz, 1H), 3.16 (dd, *J* = 11.3, 4.2 Hz, 1H), 2.69 (dd, *J* = 16.9, 2.6 Hz, 1H), 2.50 – 2.40 (m, 1H). ^{13}C NMR (101 MHz, $CDCl_3$) δ 143.8, 141.8, 129.5, 129.3, 129.0, 127.1, 122.1, 118.6, 117.1, 116.6, 114.1, 113.8, 50.6, 41.9, 29.8, 29.1. HRMS (ESI): Calcd. for $C_{16}H_{17}N_2$ $[M+H]^+$: 237.1386; found: 237.1382.

(85) (*cis*)-12-ethyl-10b-methyl-4b,5,6,10b,11,12-hexahydrodibenzo[c,h][1,6]naphthyridine (**P85**)

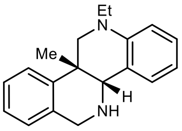

Brown solid; m.p. : 119-121 ºC, (37.2 mg, 67% yield); ^{1}H NMR (500 MHz, $CDCl_3$) δ 7.30 (d, *J* = 7.5 Hz, 1H), 7.18 – 7.11 (m, 2H), 7.07 (td, *J* = 7.7, 2.2 Hz, 2H), 6.94 (d, *J* = 7.6 Hz, 1H), 6.60 – 6.54 (m, 2H), 4.19 (d, *J* = 15.2 Hz, 1H), 3.93 (d, *J* = 15.5 Hz, 1H), 3.55 – 3.36 (m, 3H), 3.22 (dqd, *J* = 14.3, 7.1, 1.9 Hz, 1H), 2.78 (dt, *J* = 11.6, 1.9 Hz, 1H), 1.14 (d, *J* = 2.3 Hz, 3H), 1.06 (td, *J* = 7.1, 2.0 Hz, 3H).^{13}C NMR (126 MHz, $CDCl_3$) δ 143.5, 141.2, 135.9, 131.6, 128.7, 126.8, 126.6, 126.2, 126.1, 122.5, 115.8, 110.6, 61.4, 55.9, 49.1, 45.3, 35.1, 22.7, 11.1. HRMS (ESI): Calcd. for $C_{19}H_{23}N_2$ $[M+H]^+$: 279.1856; found: 279.1854.

Supplementary Figure

NMR spectra of the obtained compounds

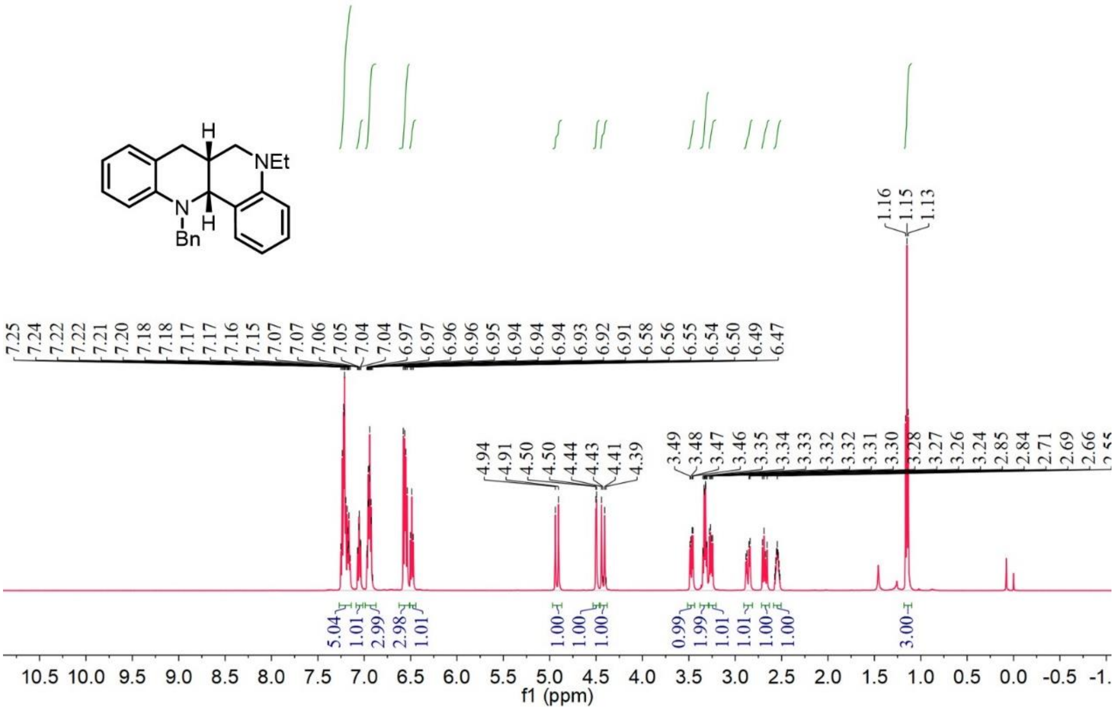

Supplementary Fig. 18 ^{1}H-NMR spectrum of P1

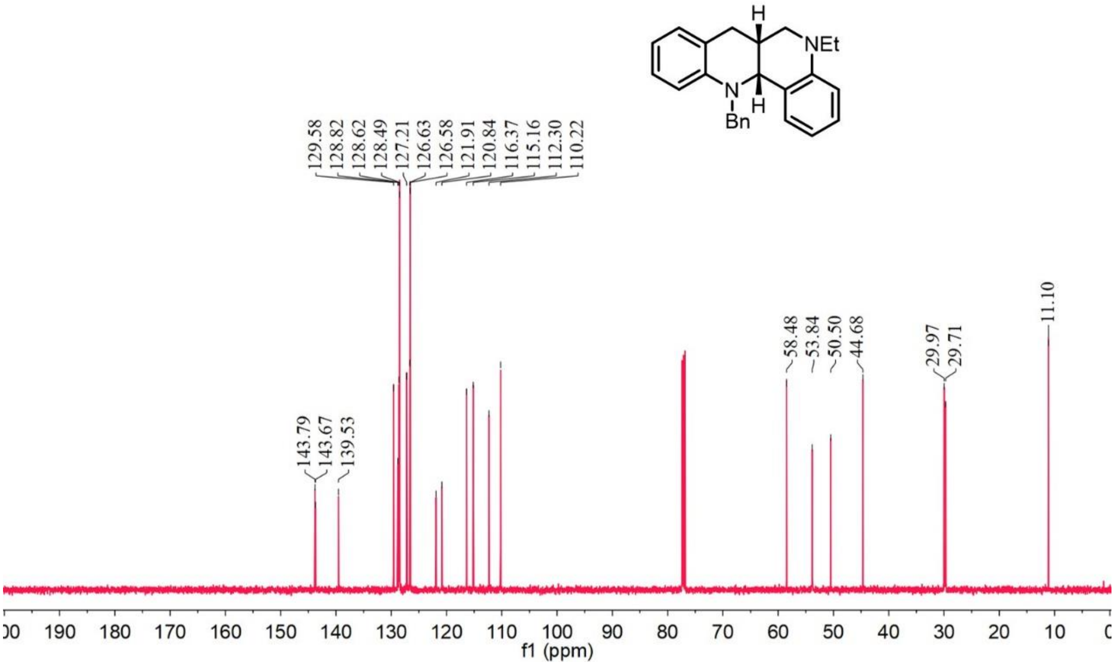

Supplementary Fig. 19 ^{13}C-NMR spectrum of P1

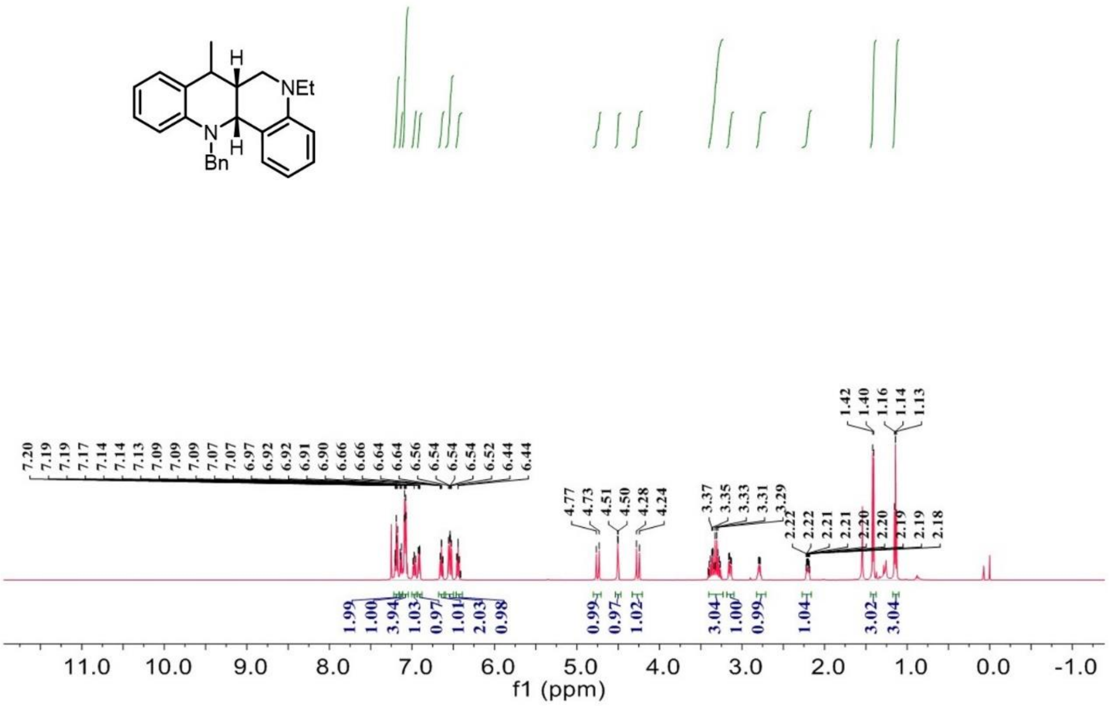

Supplementary Fig. 20 ^{1}H-NMR spectrum of P2

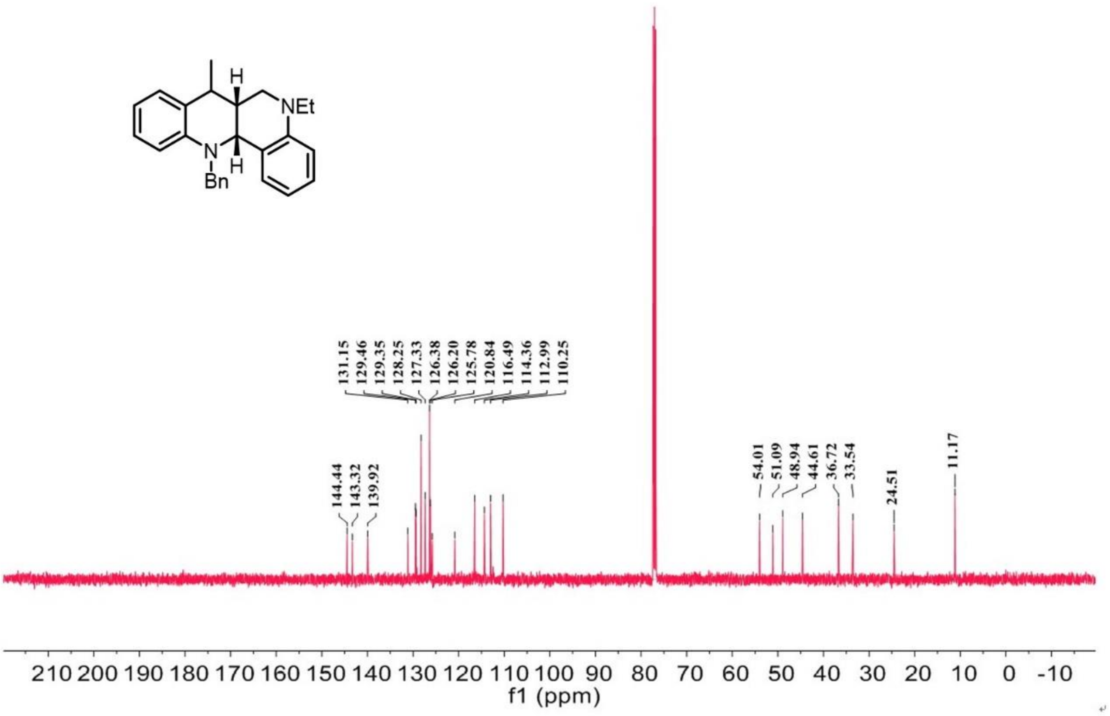

Supplementary Fig. 21 ^{13}C-NMR spectrum of P2

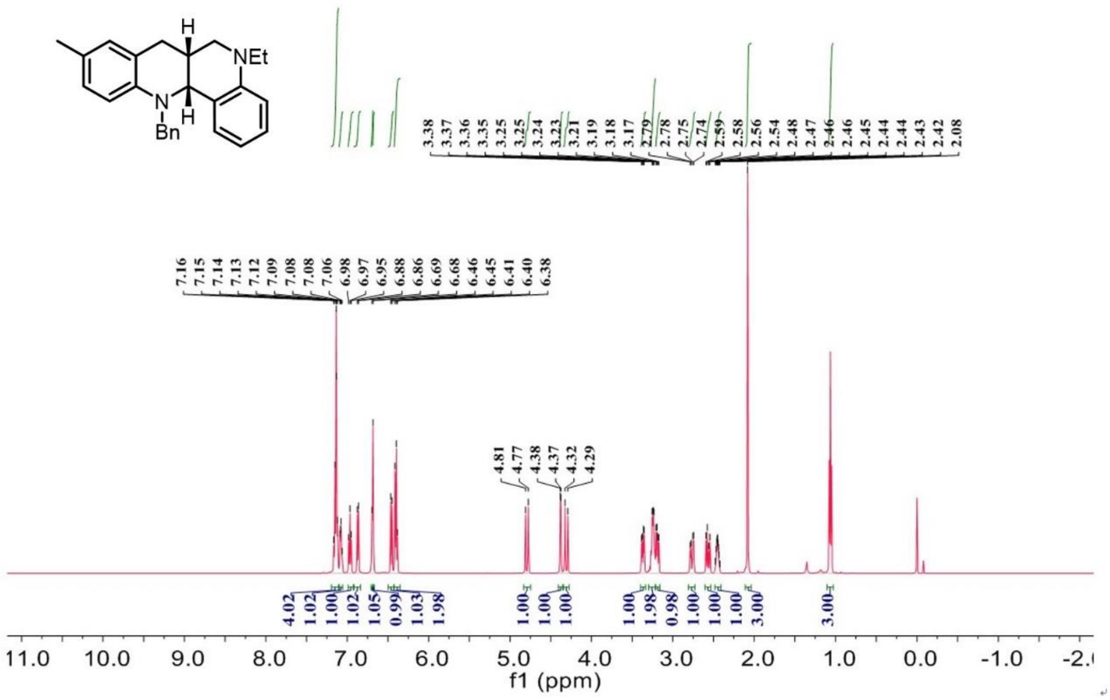

Supplementary Fig. 22 ^{1}H-NMR spectrum of P3

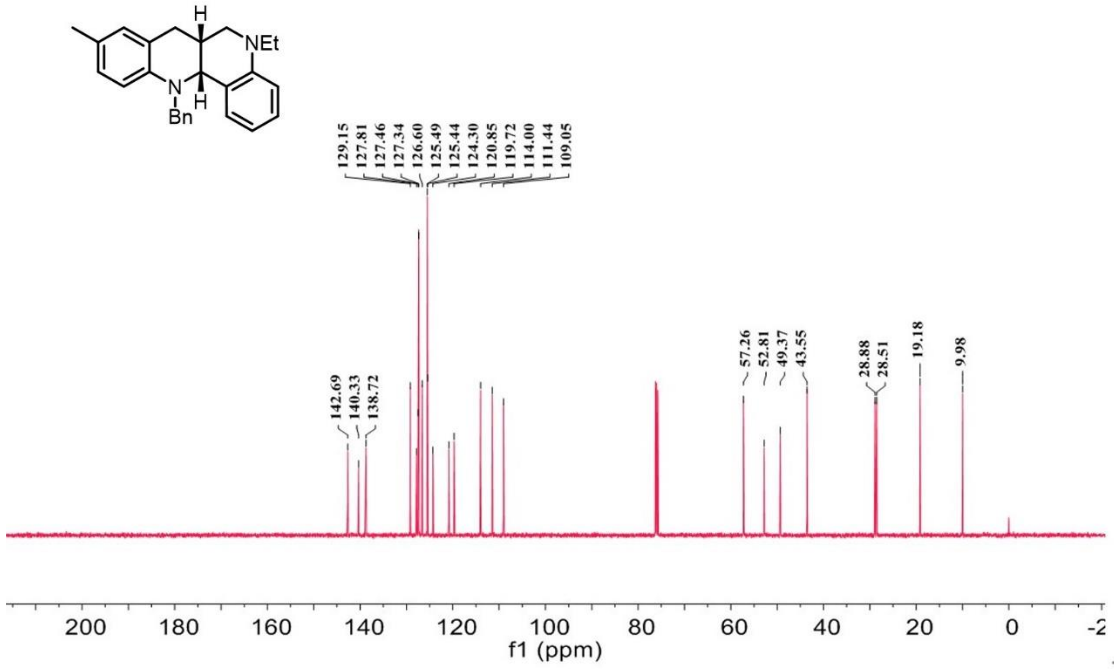

Supplementary Fig. 23 ^{13}C-NMR spectrum of P3

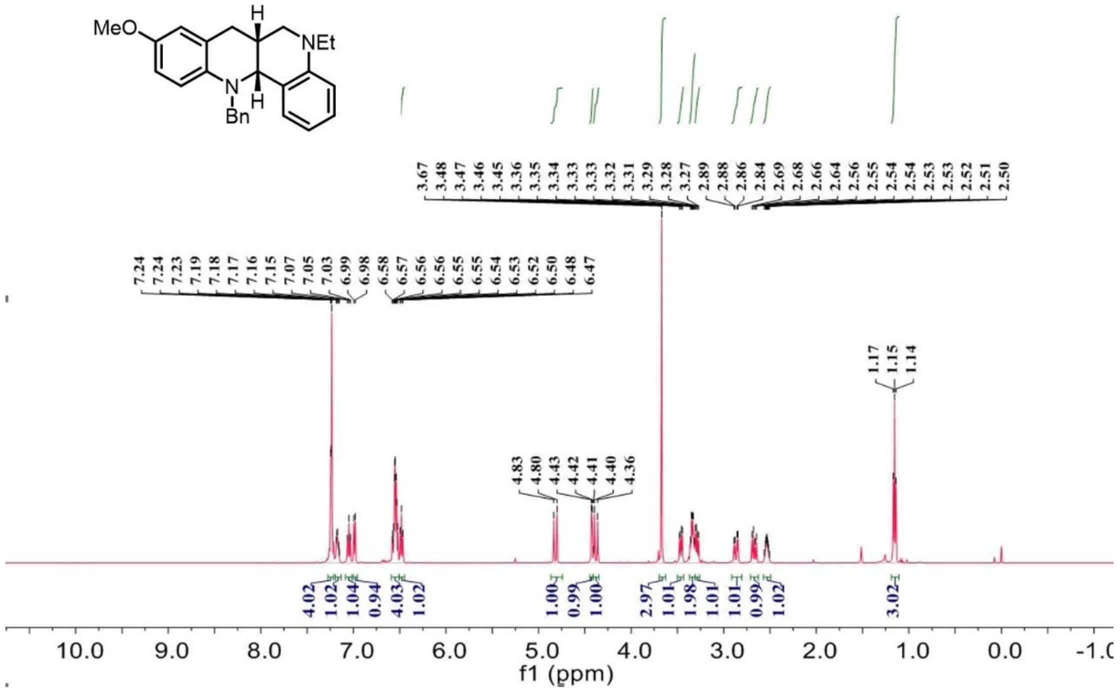

Supplementary Fig. 24 ^{1}H-NMR spectrum of P4

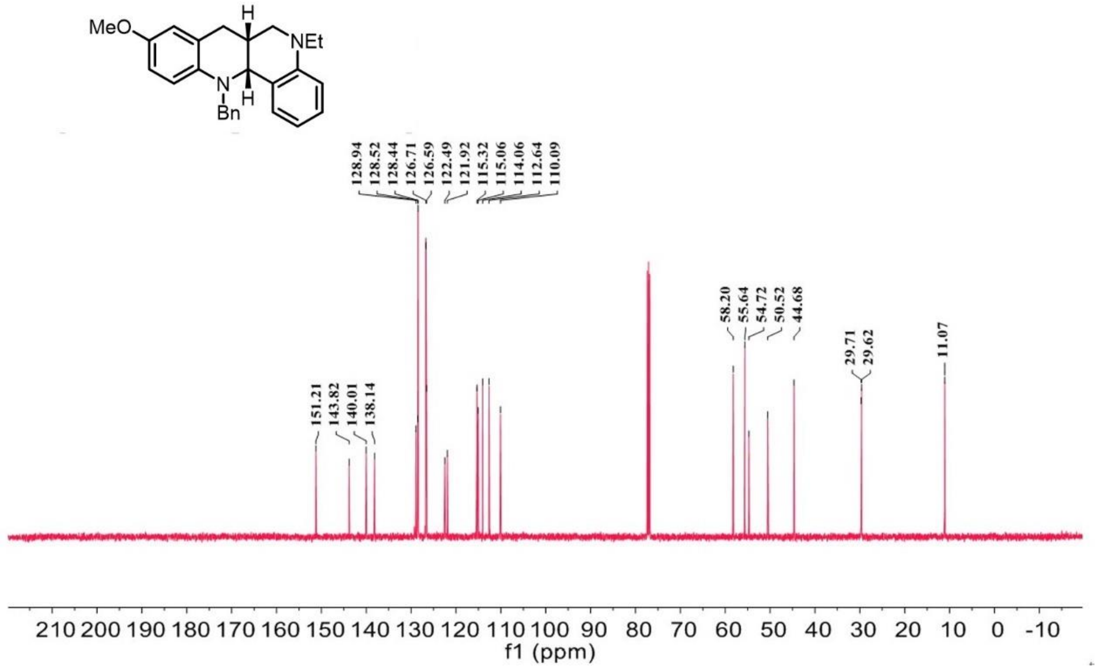

Supplementary Fig. 25 ^{13}C-NMR spectrum of P4

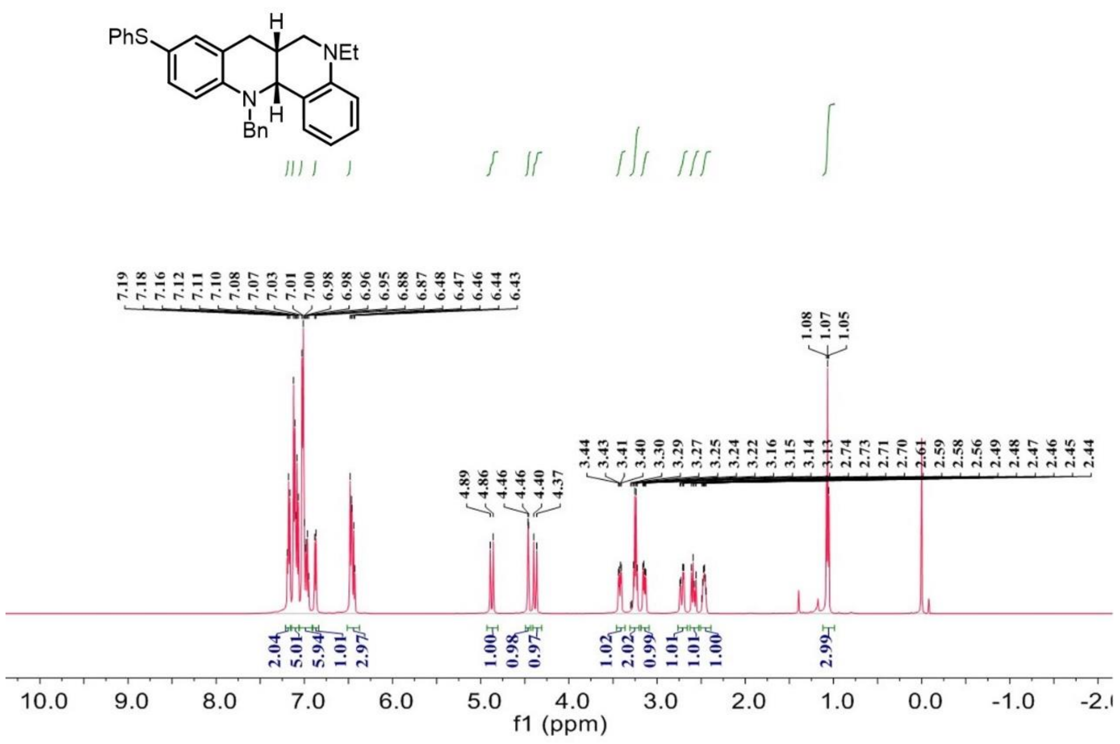

Supplementary Fig. 26 ^{1}H-NMR spectrum of P5

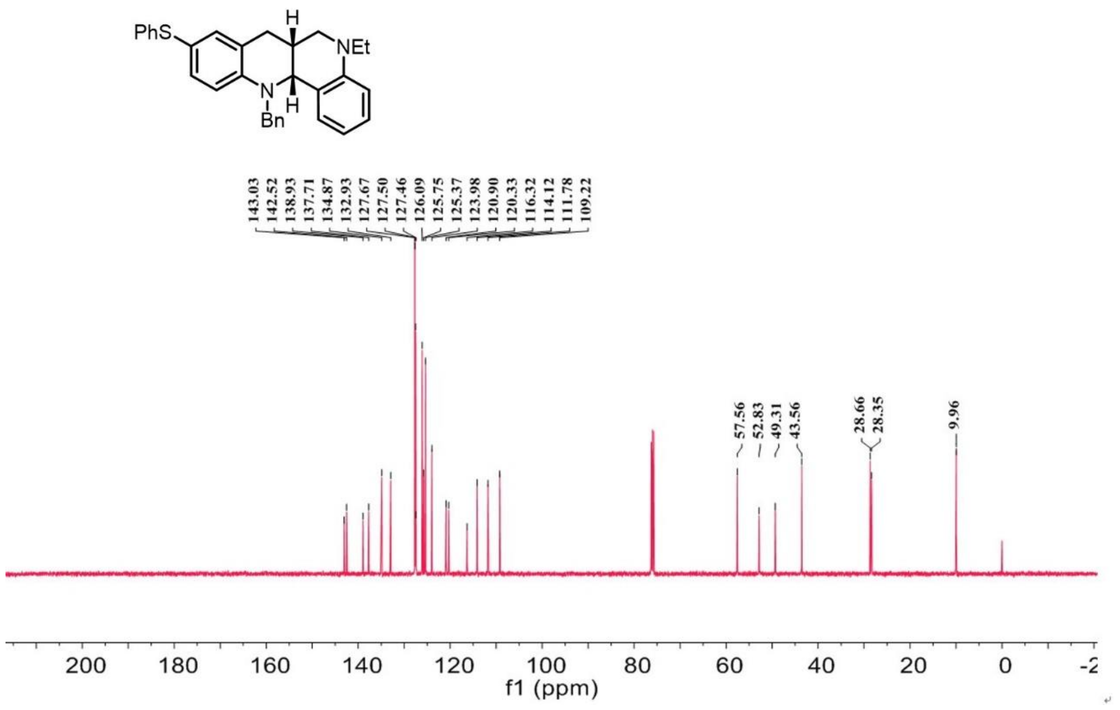

Supplementary Fig. 27 ^{13}C-NMR spectrum of P5

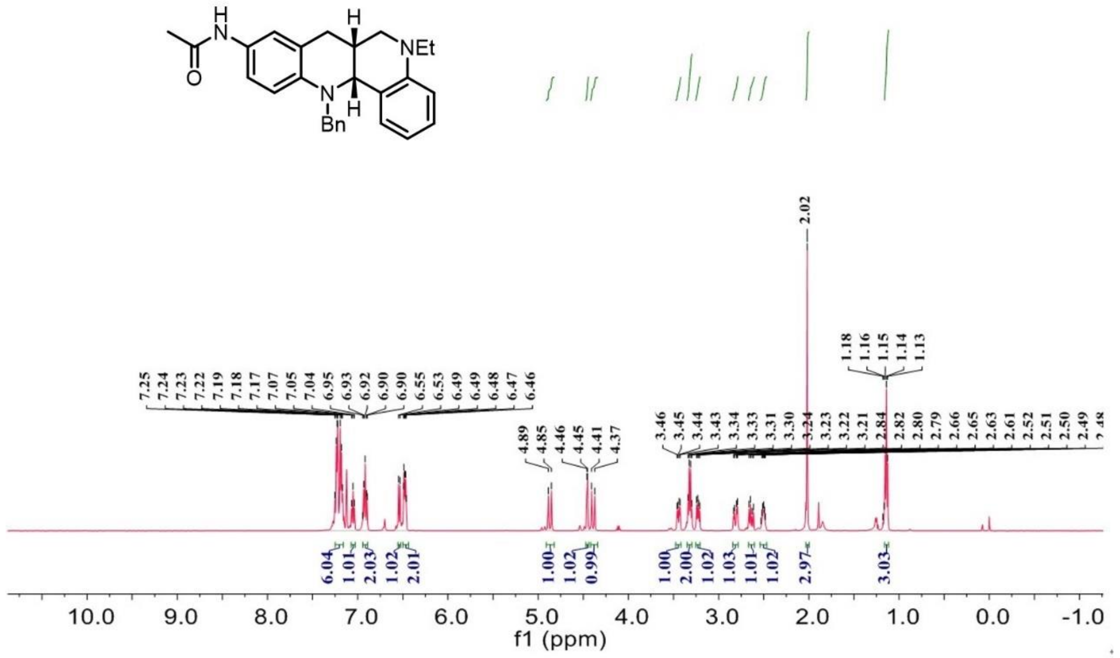

Supplementary Fig. 28 ^{1}H-NMR spectrum of P6

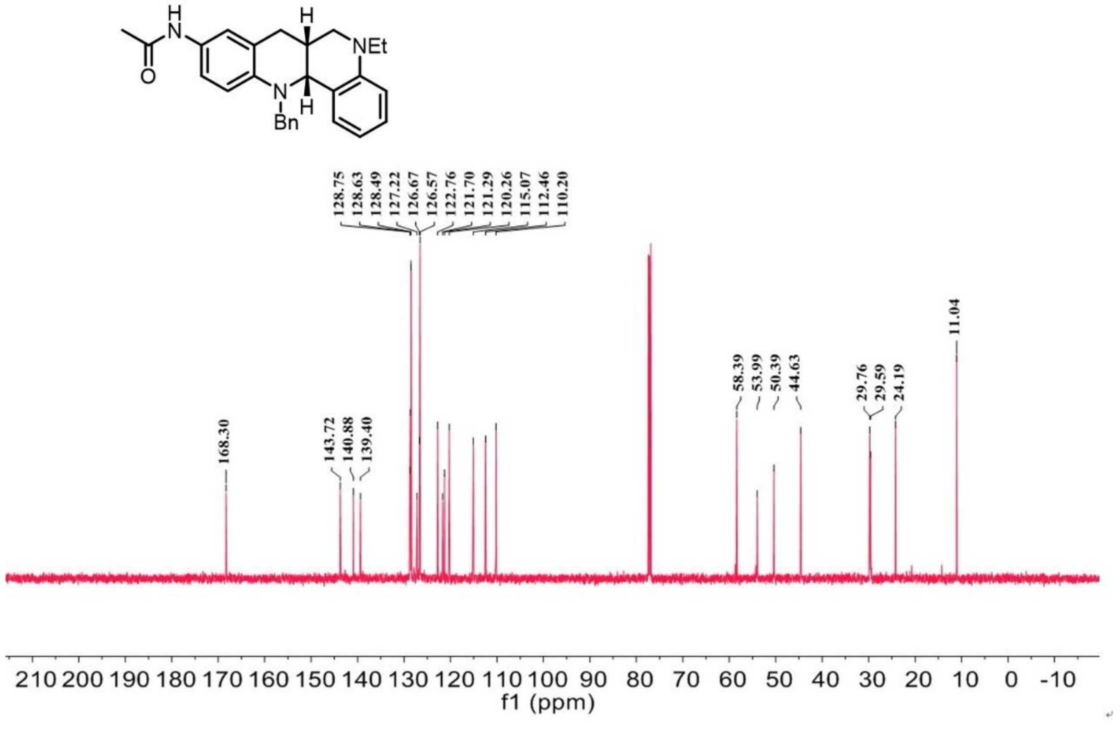

Supplementary Fig. 29 ^{13}C-NMR spectrum of P6

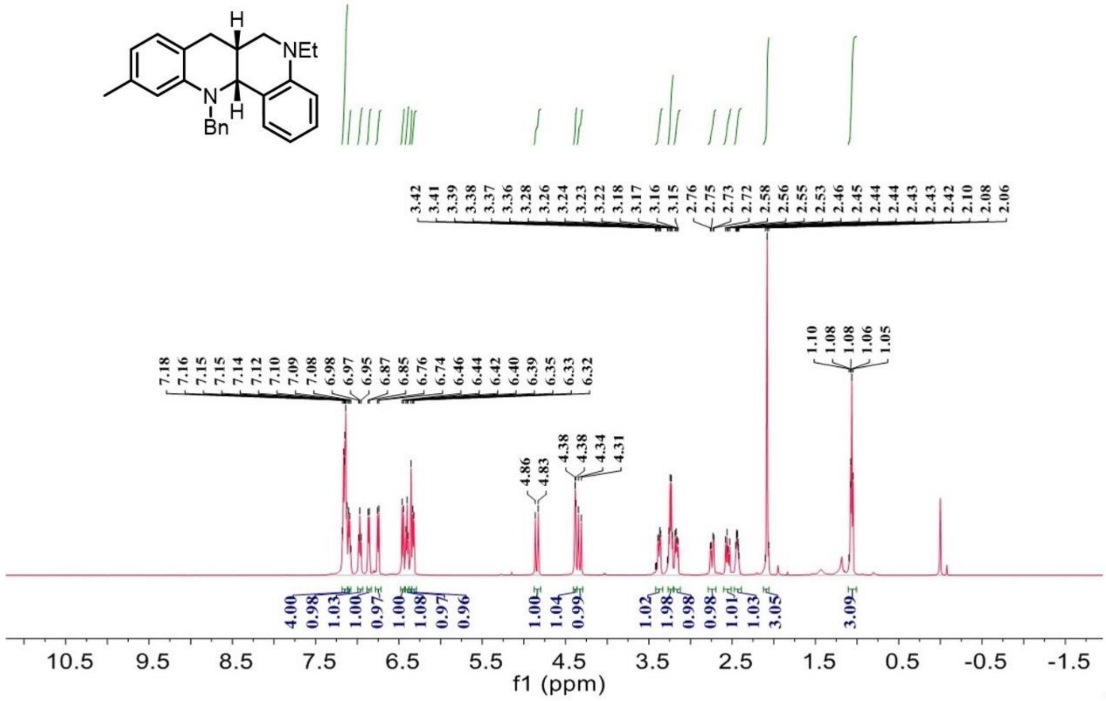

Supplementary Fig. 30 ^{1}H-NMR spectrum of P7

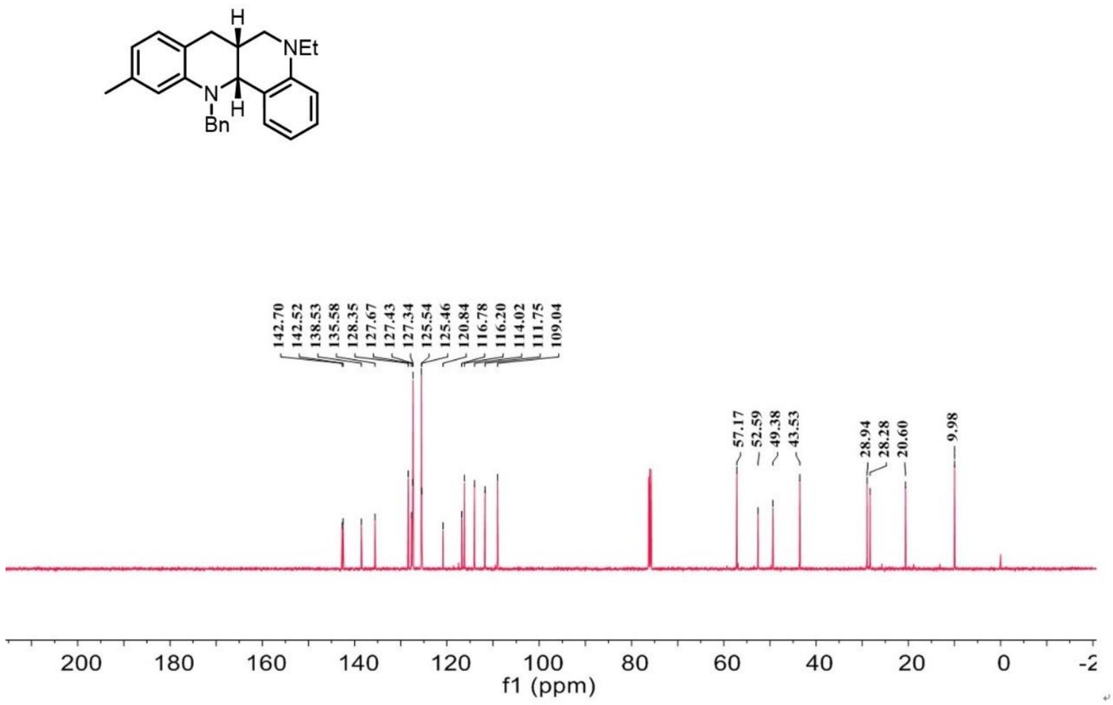

Supplementary Fig. 31 ^{13}C-NMR spectrum of P7

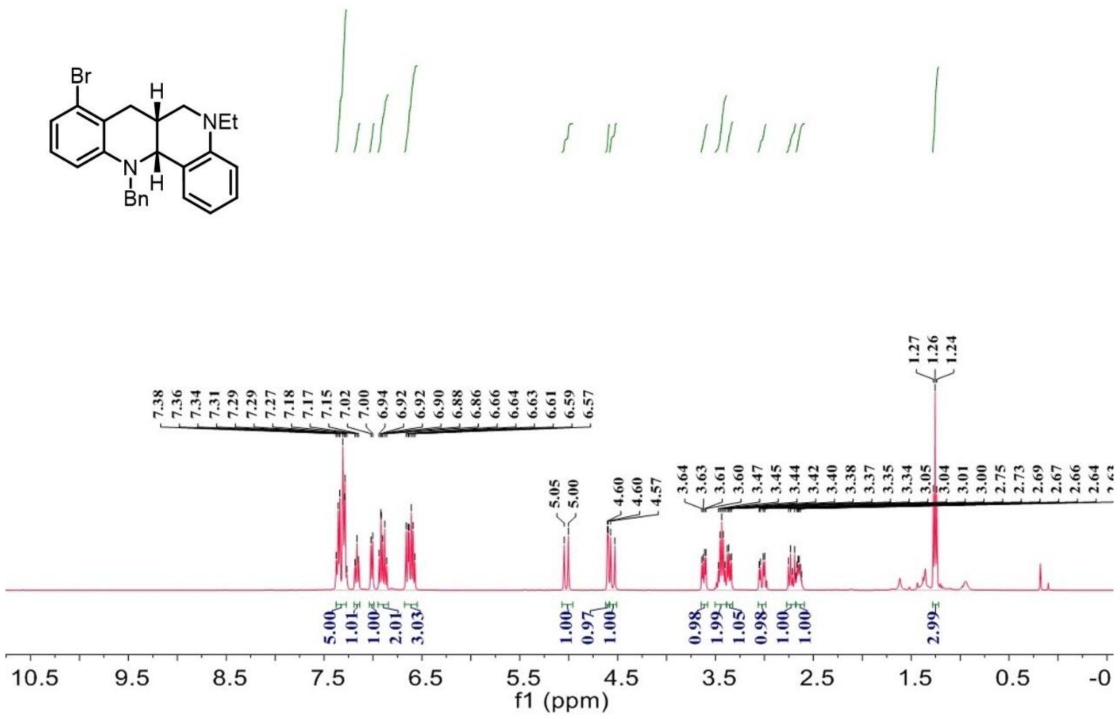

Supplementary Fig. 32 ^{1}H-NMR spectrum of P8

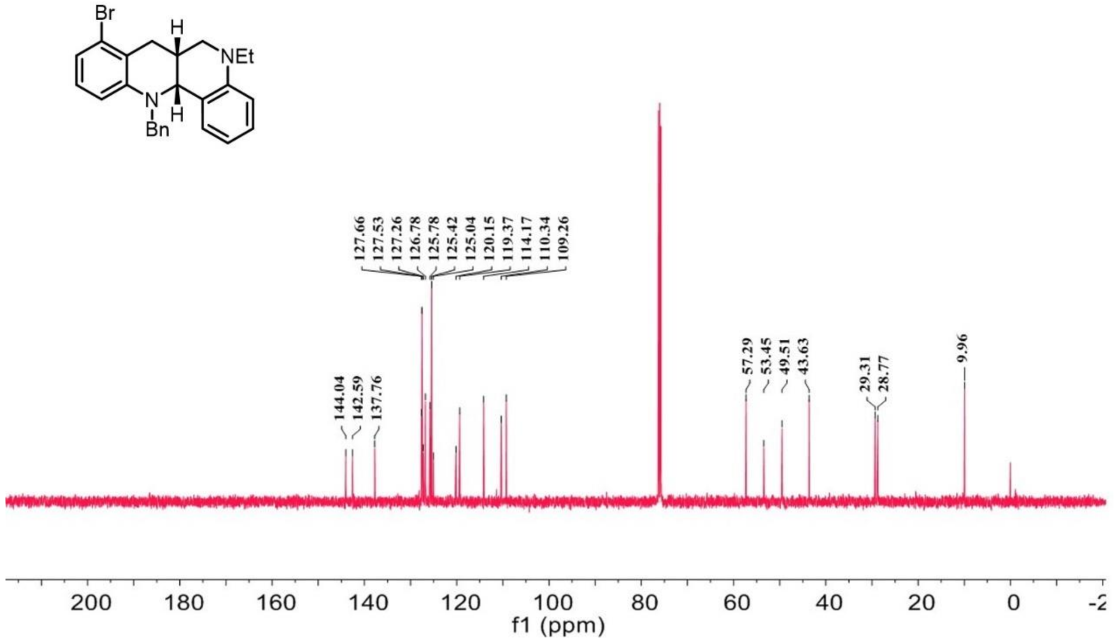

Supplementary Fig. 33 ^{13}C-NMR spectrum of P8

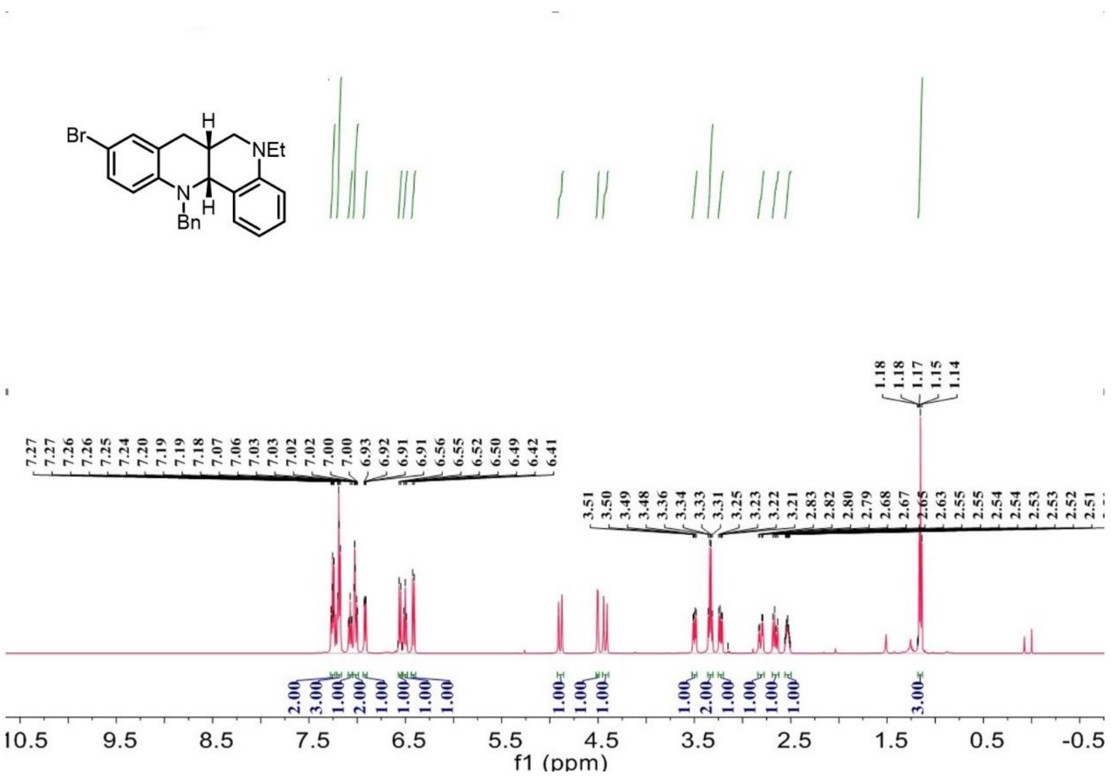

Supplementary Fig. 34 ^{1}H-NMR spectrum of P9

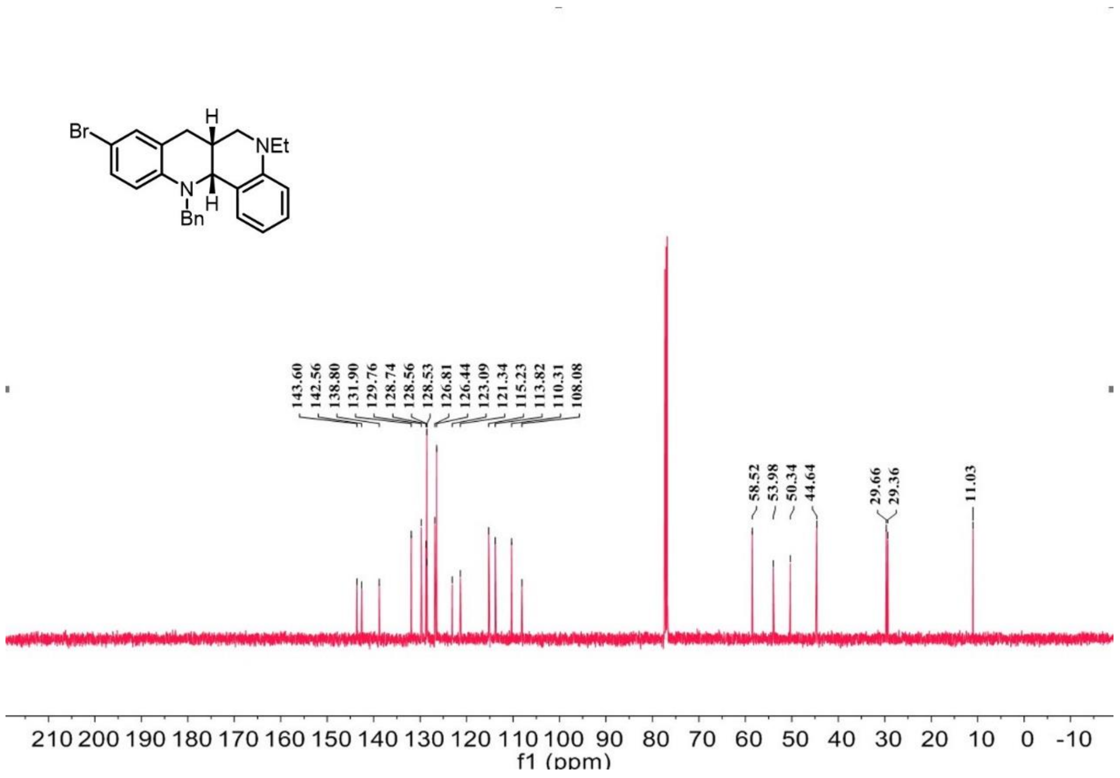

Supplementary Fig. 35 ^{13}C-NMR spectrum of P9

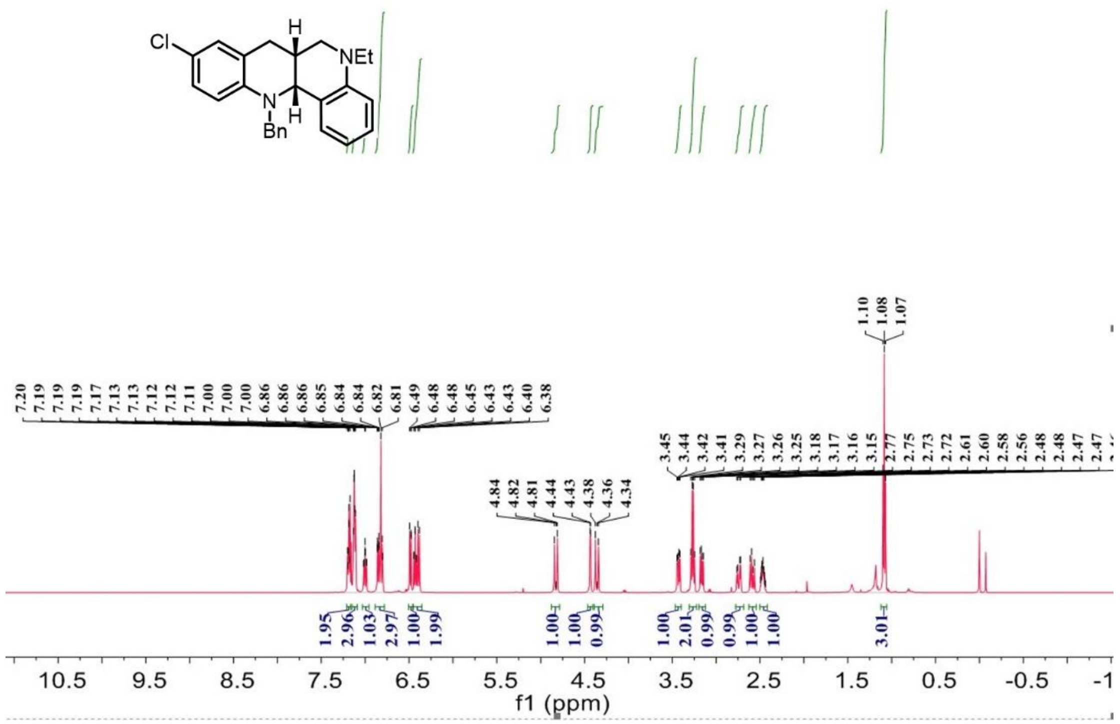

Supplementary Fig. 36 ^{1}H-NMR spectrum of P10

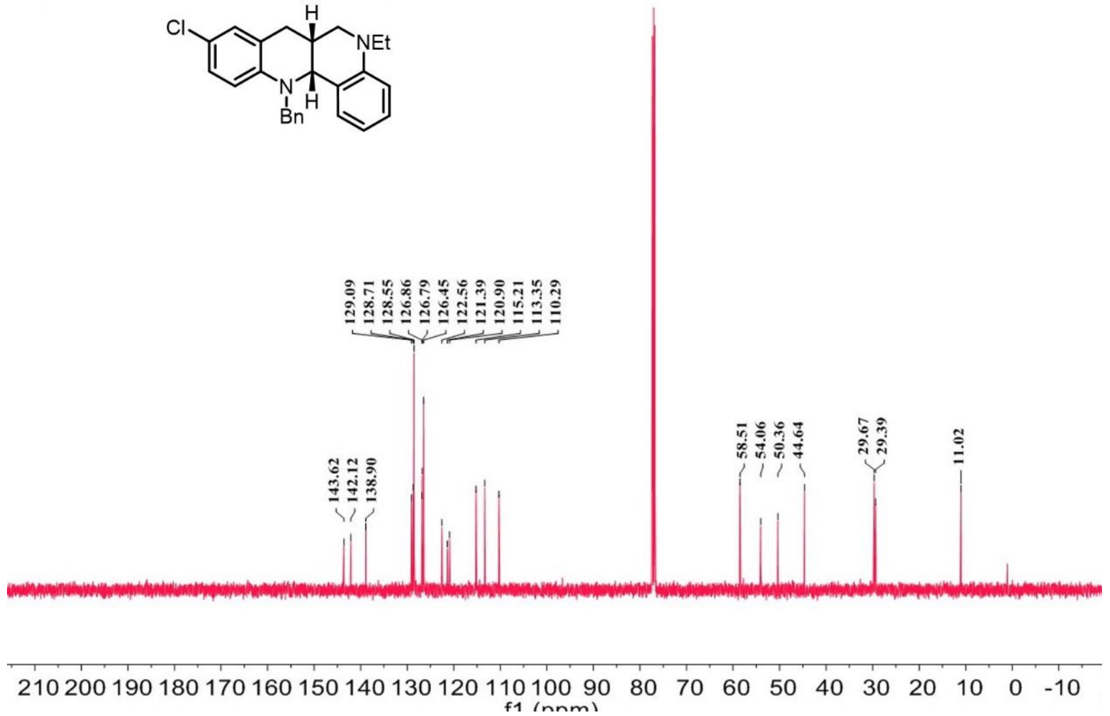

Supplementary Fig. 37 ^{13}C-NMR spectrum of P10

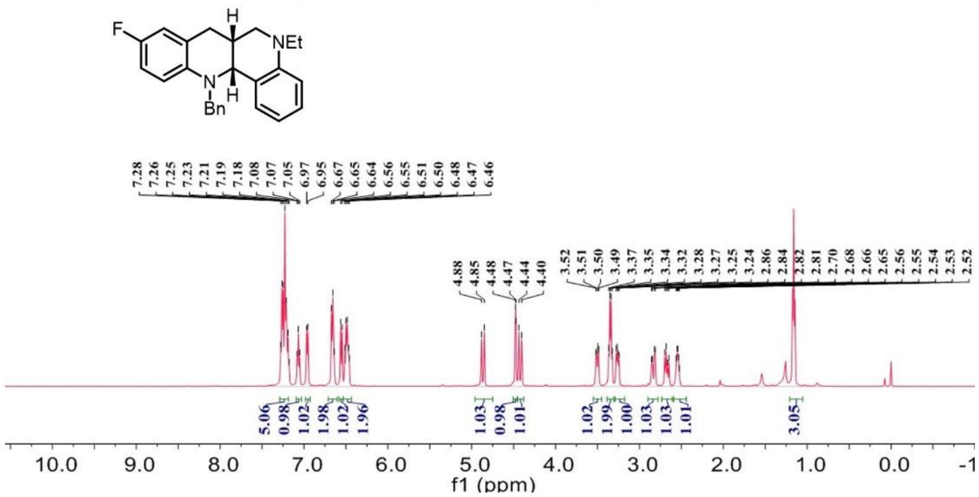

Supplementary Fig. 38 ^{1}H-NMR spectrum of P11

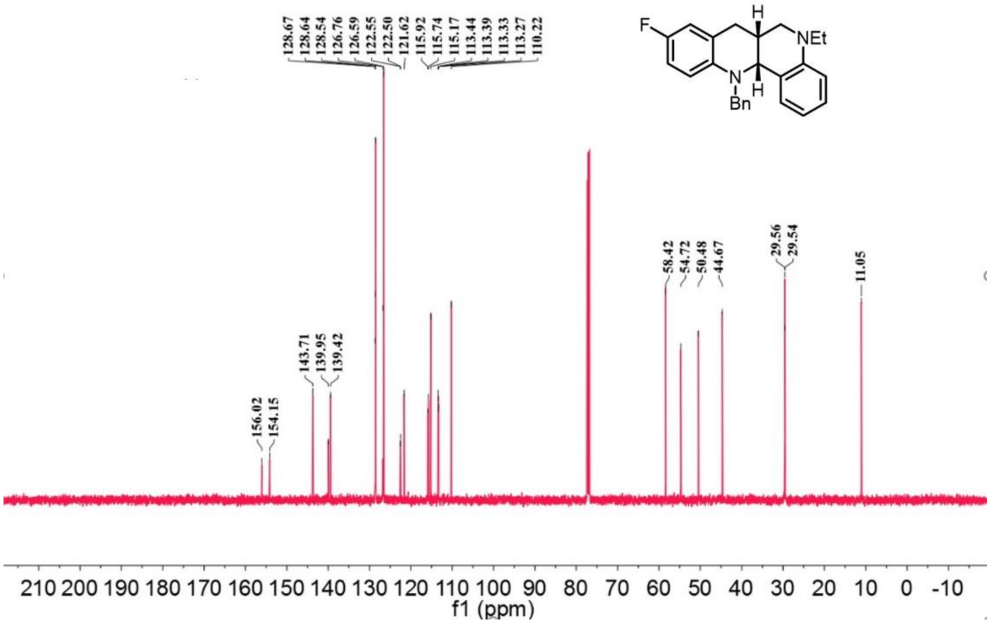

Supplementary Fig. 39 ^{13}C-NMR spectrum of P11

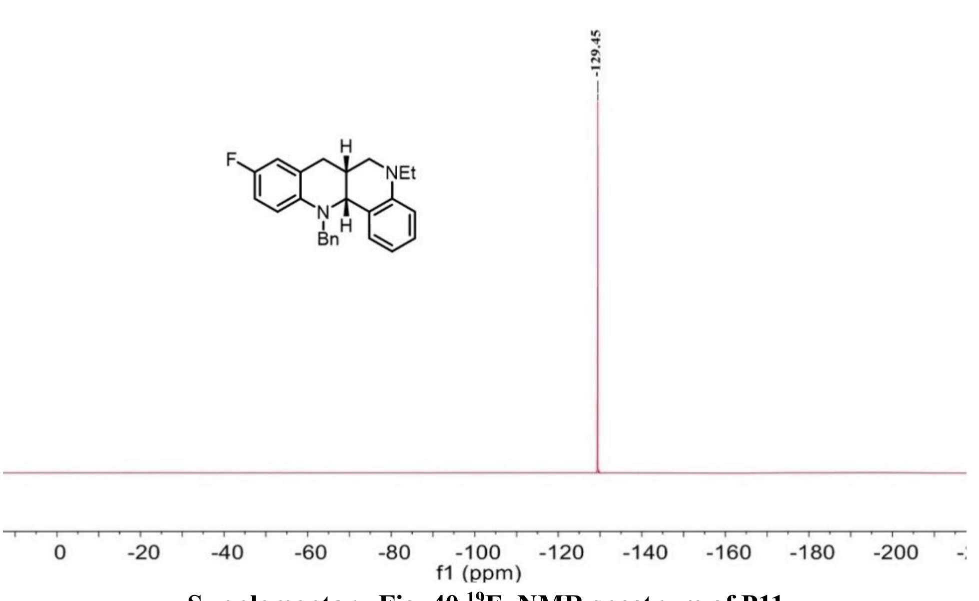

Supplementary Fig. 40 ^{19}F -NMR spectrum of P11

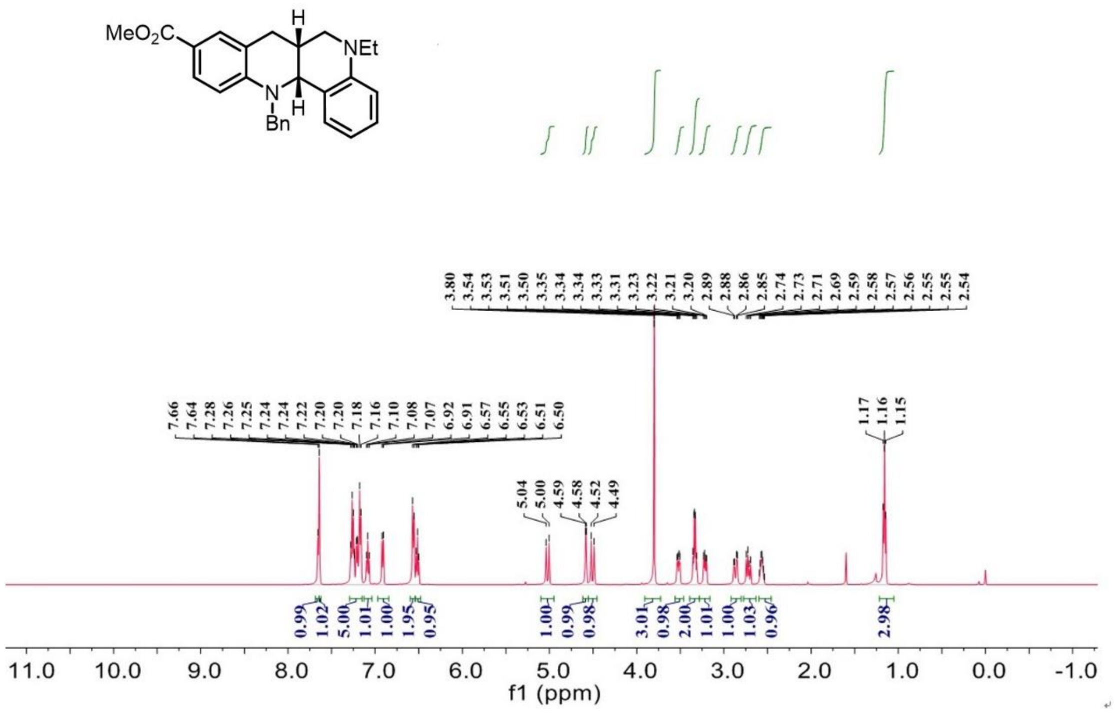

Supplementary Fig. 41 ^{1}H-NMR spectrum of P12

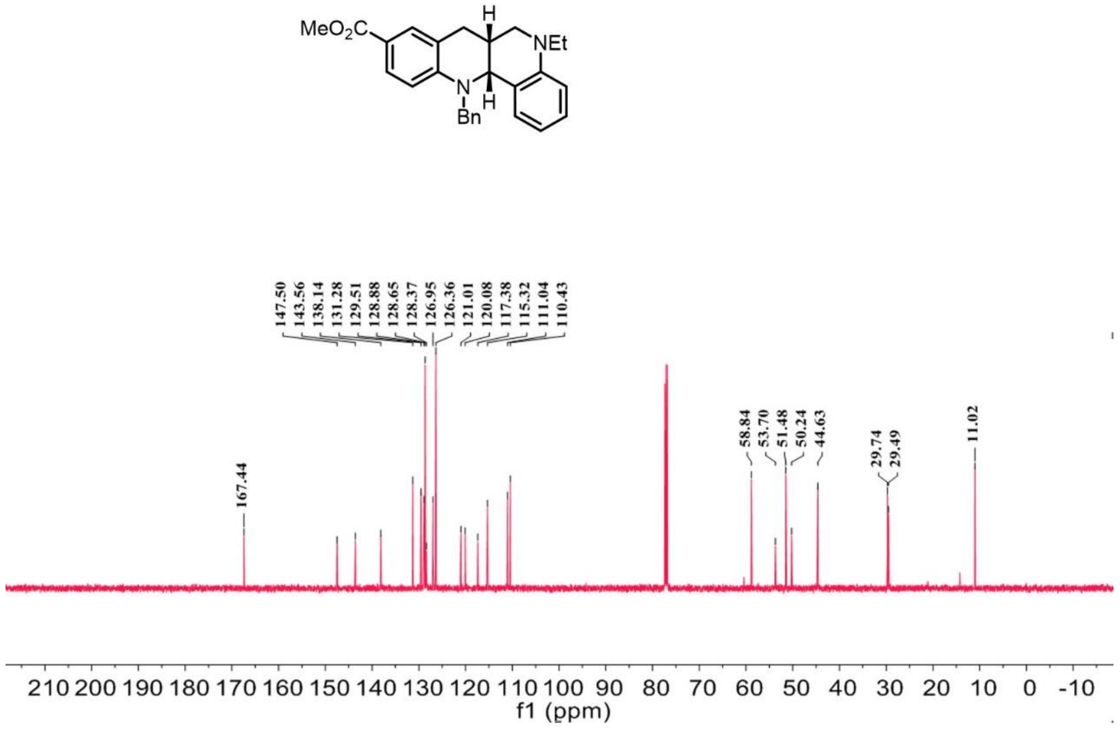

Supplementary Fig. 42 ^{13}C-NMR spectrum of P12

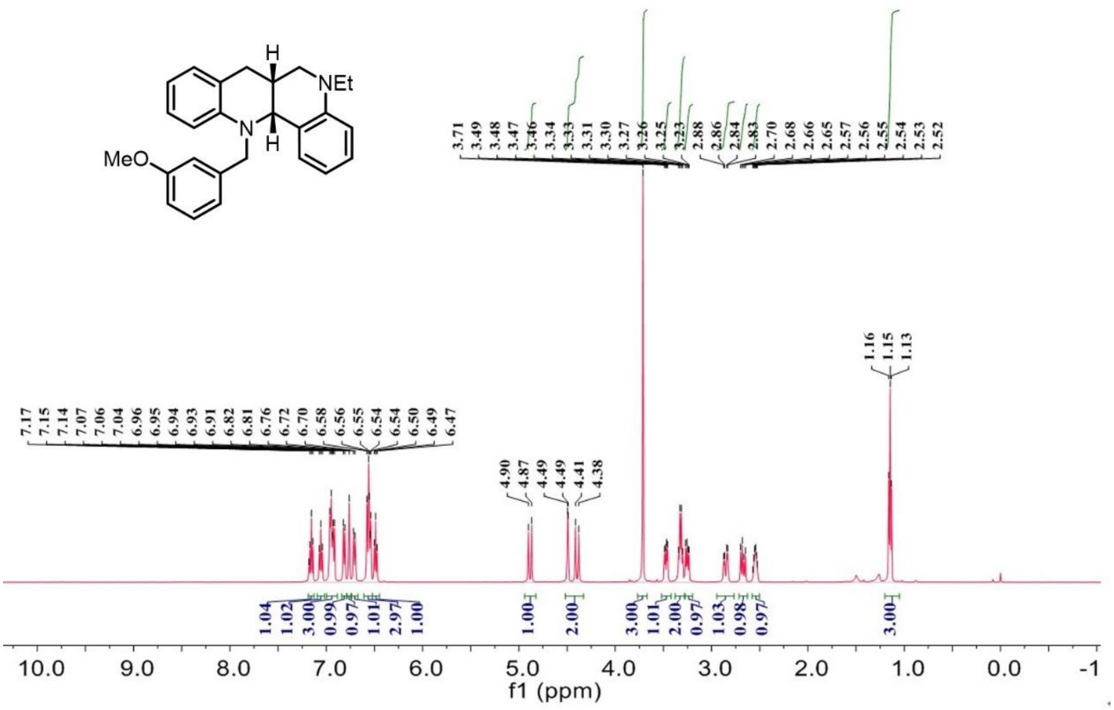

Supplementary Fig. 43 ^{1}H-NMR spectrum of P13

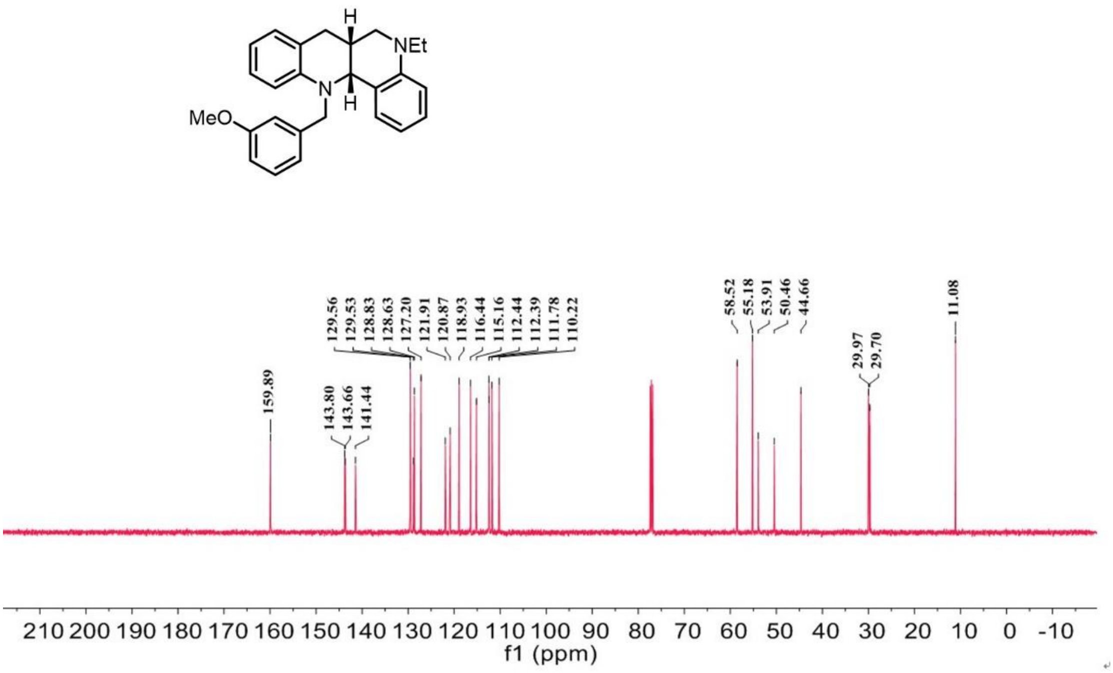

Supplementary Fig. 44 ^{13}C-NMR spectrum of P13

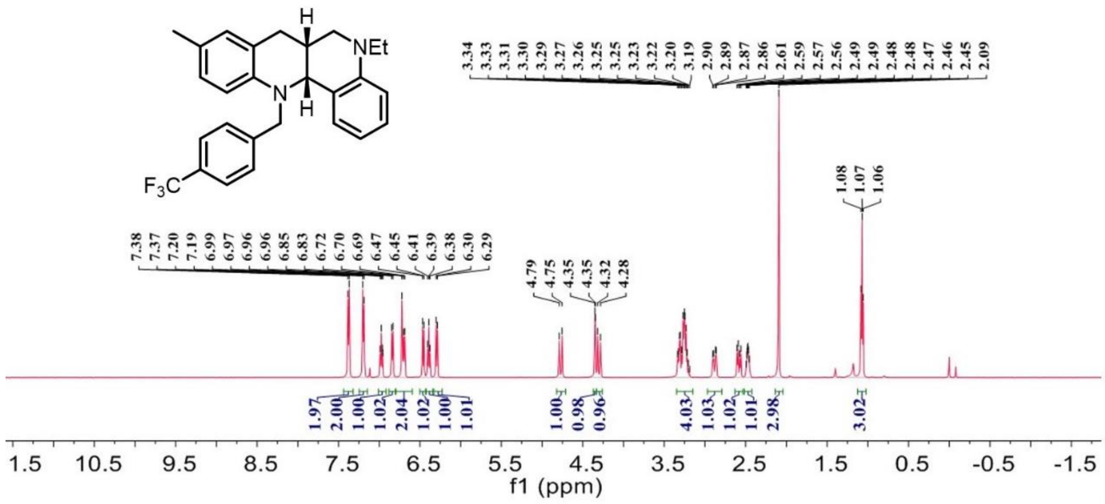

Supplementary Fig. 45 ^{1}H-NMR spectrum of P14

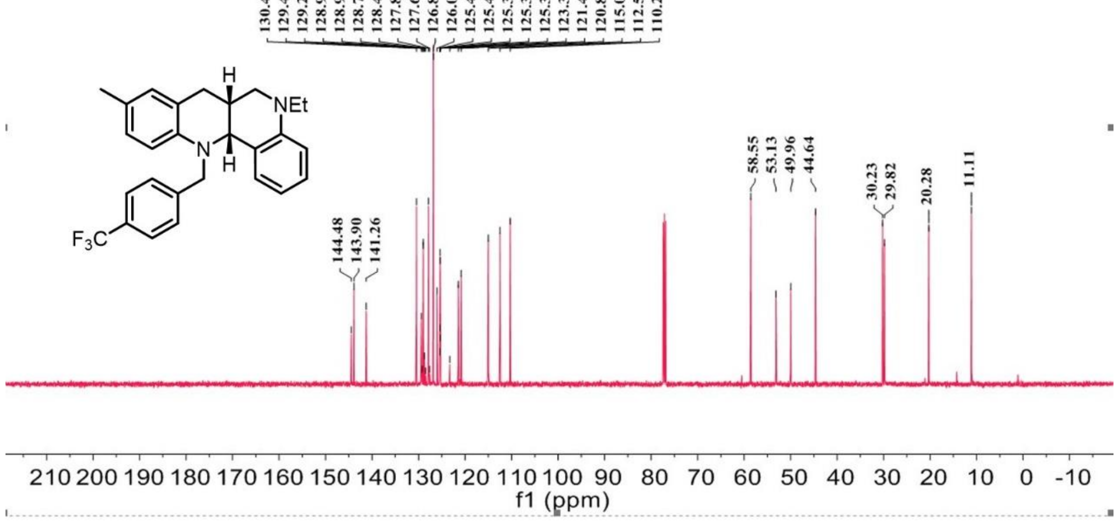

Supplementary Fig. 46 ^{13}C-NMR spectrum of P14

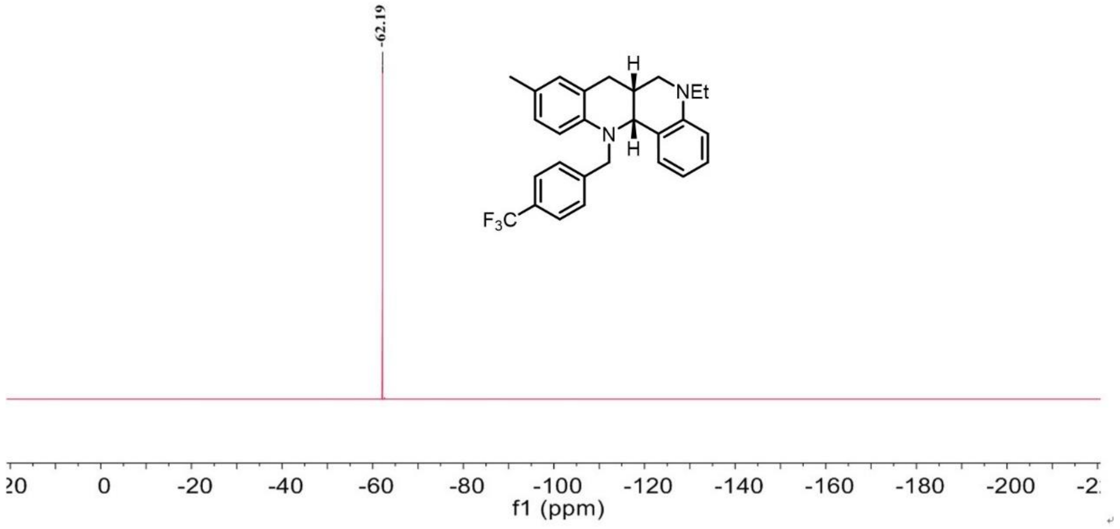

Supplementary Fig. 47 ^{19}F -NMR spectrum of P14

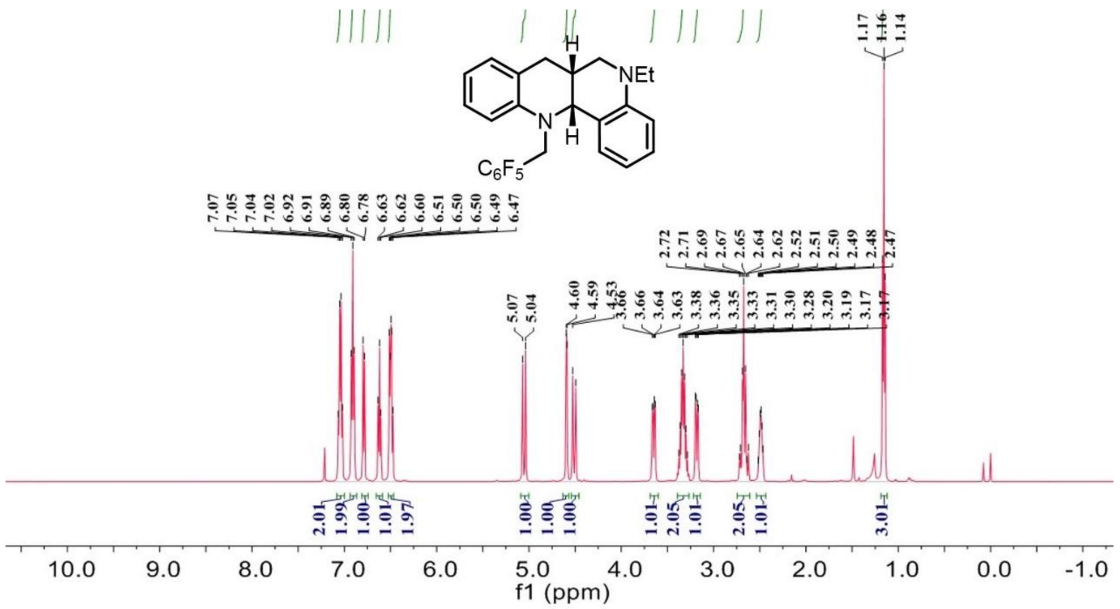

Supplementary Fig. 48 ^{1}H-NMR spectrum of P15

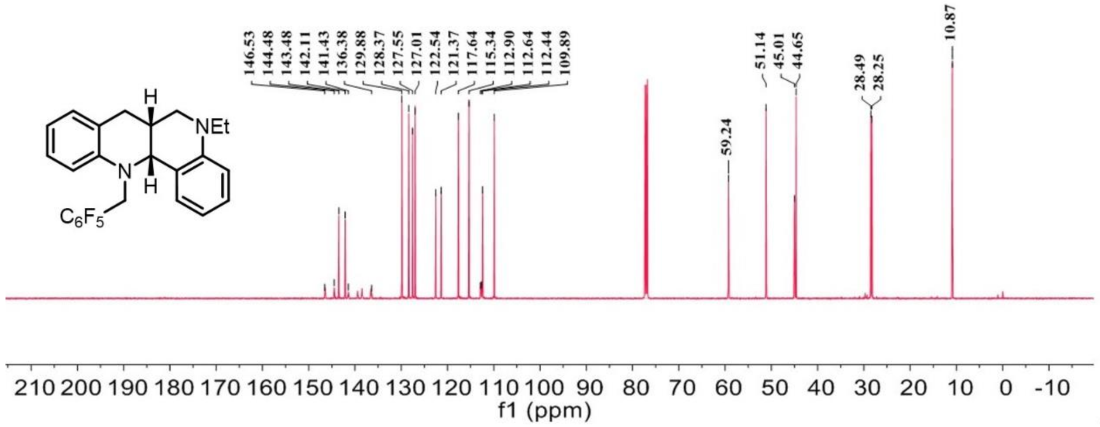

Supplementary Fig. 49 ^{13}C-NMR spectrum of P15

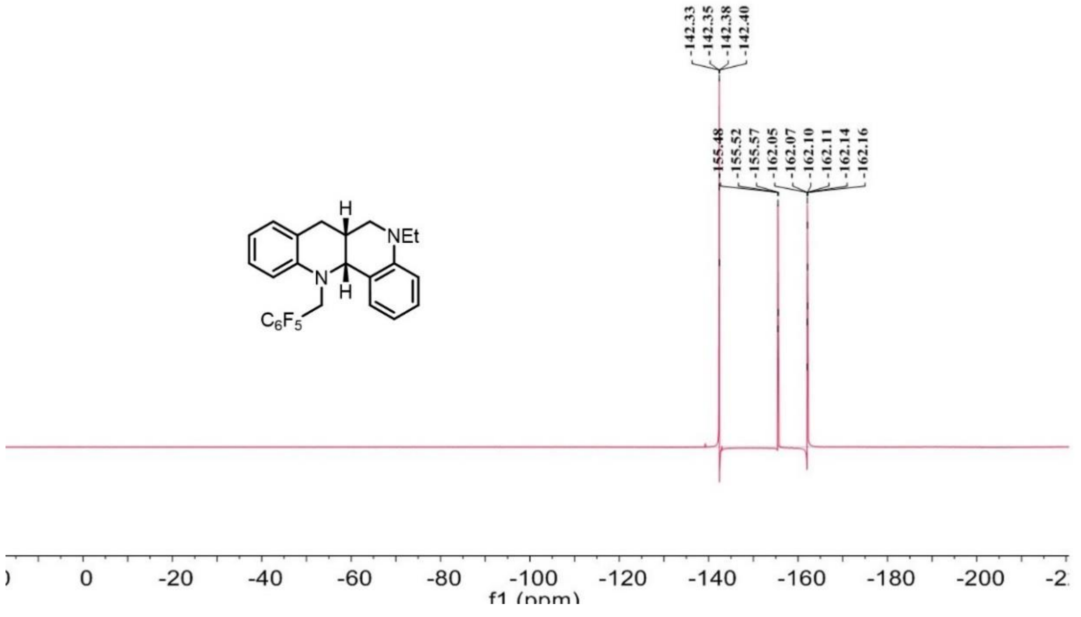

Supplementary Fig. 50 ^{19}F -NMR spectrum of P15

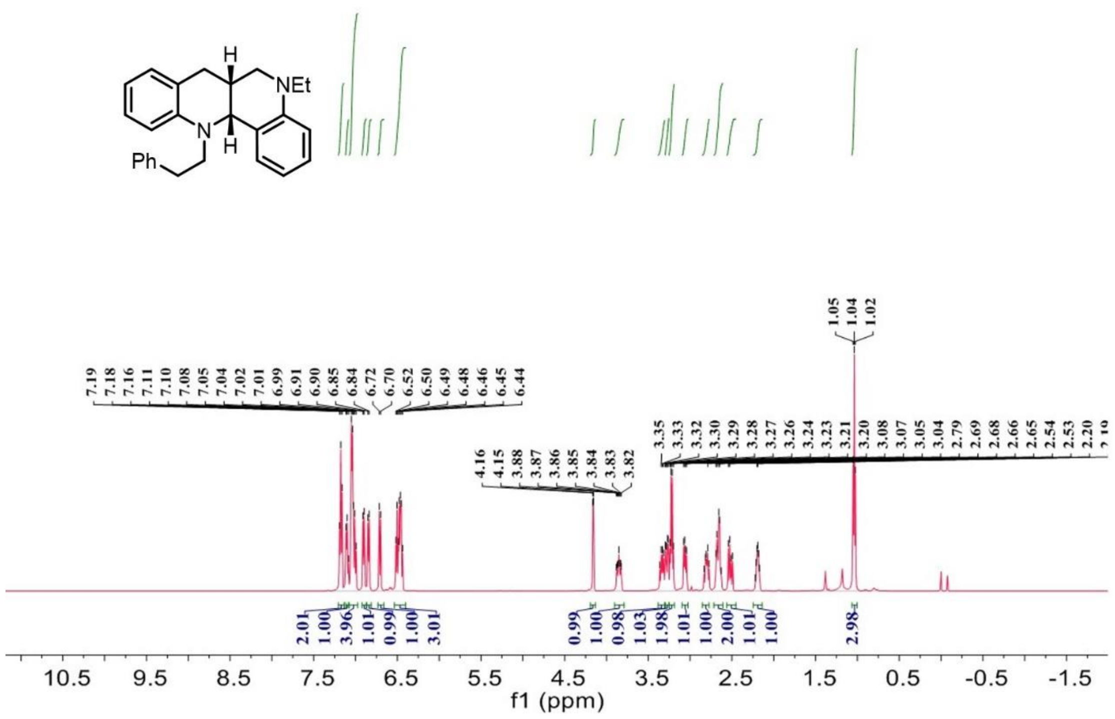

Supplementary Fig. 51 ^{1}H-NMR spectrum of P16

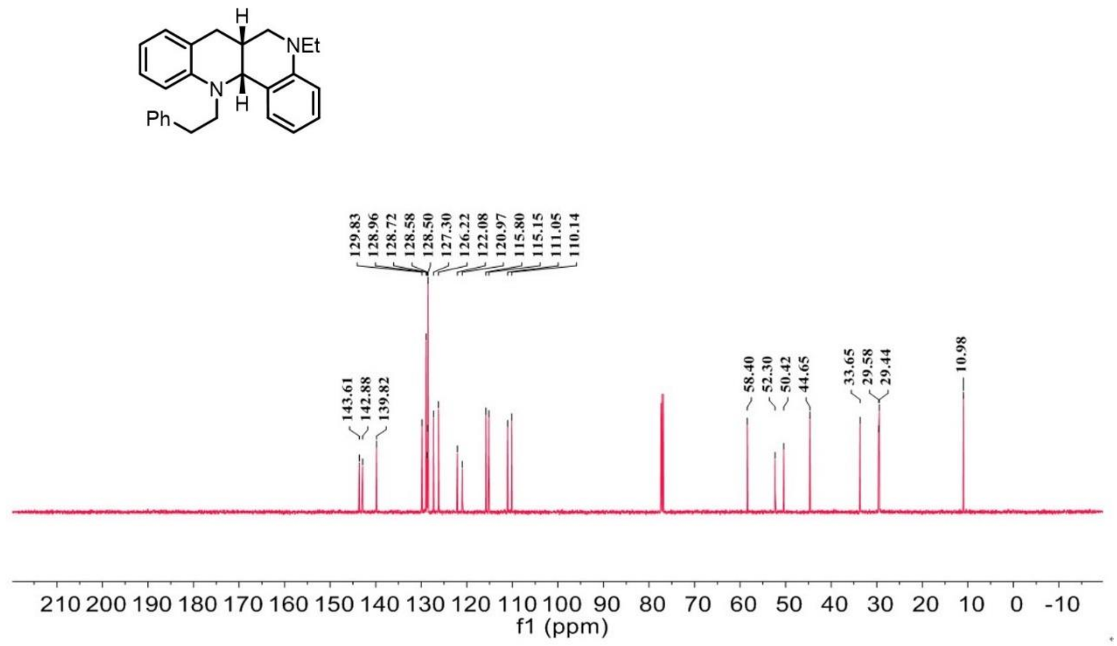

Supplementary Fig. 52 ^{13}C-NMR spectrum of P16

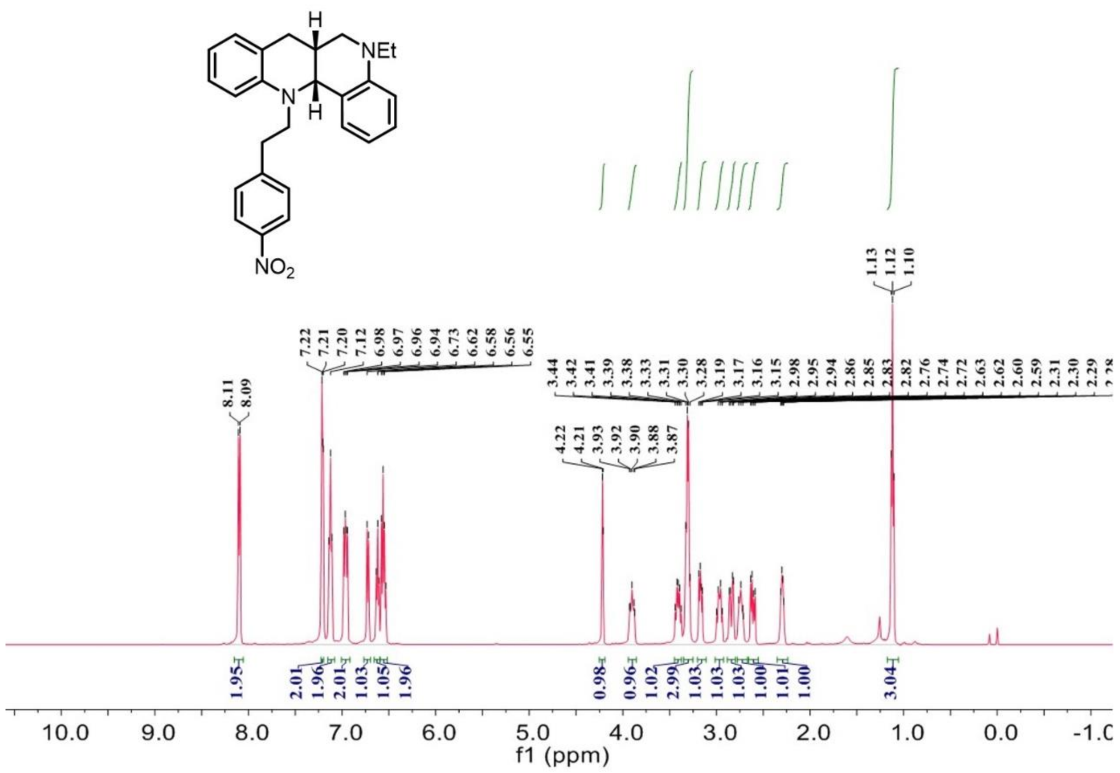

Supplementary Fig. 53 ^{1}H-NMR spectrum of P17

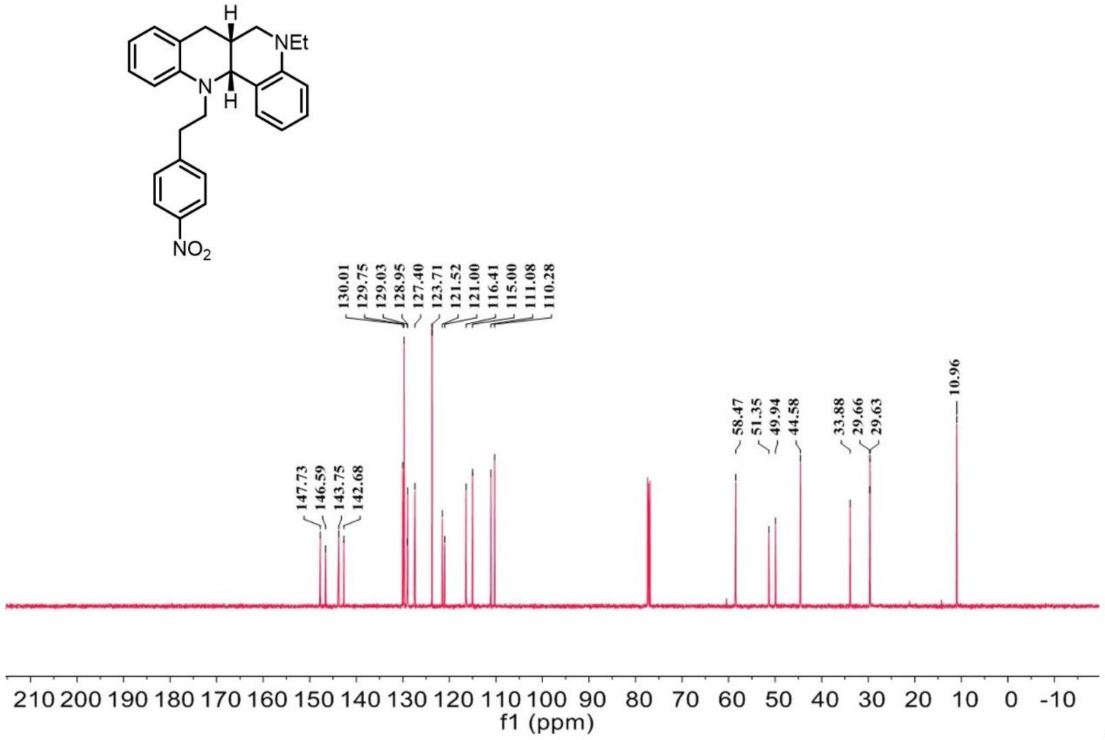

Supplementary Fig. 54 ^{13}C-NMR spectrum of P17

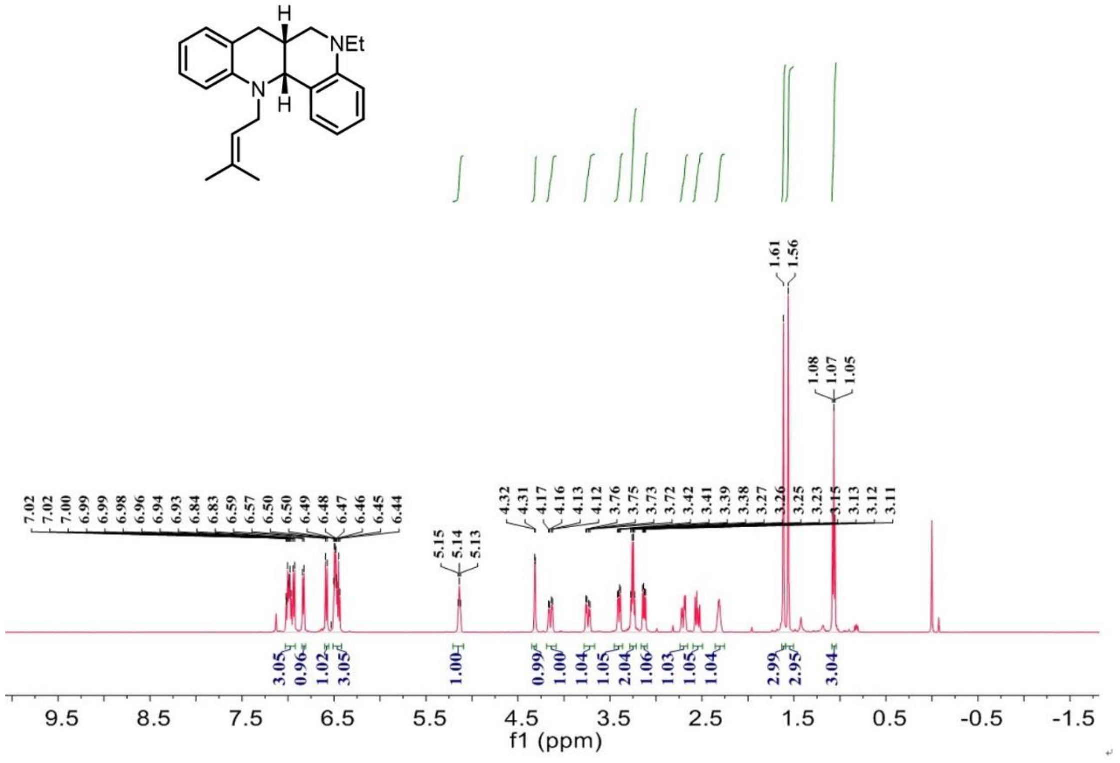

Supplementary Fig. 55 ^{1}H-NMR spectrum of P18

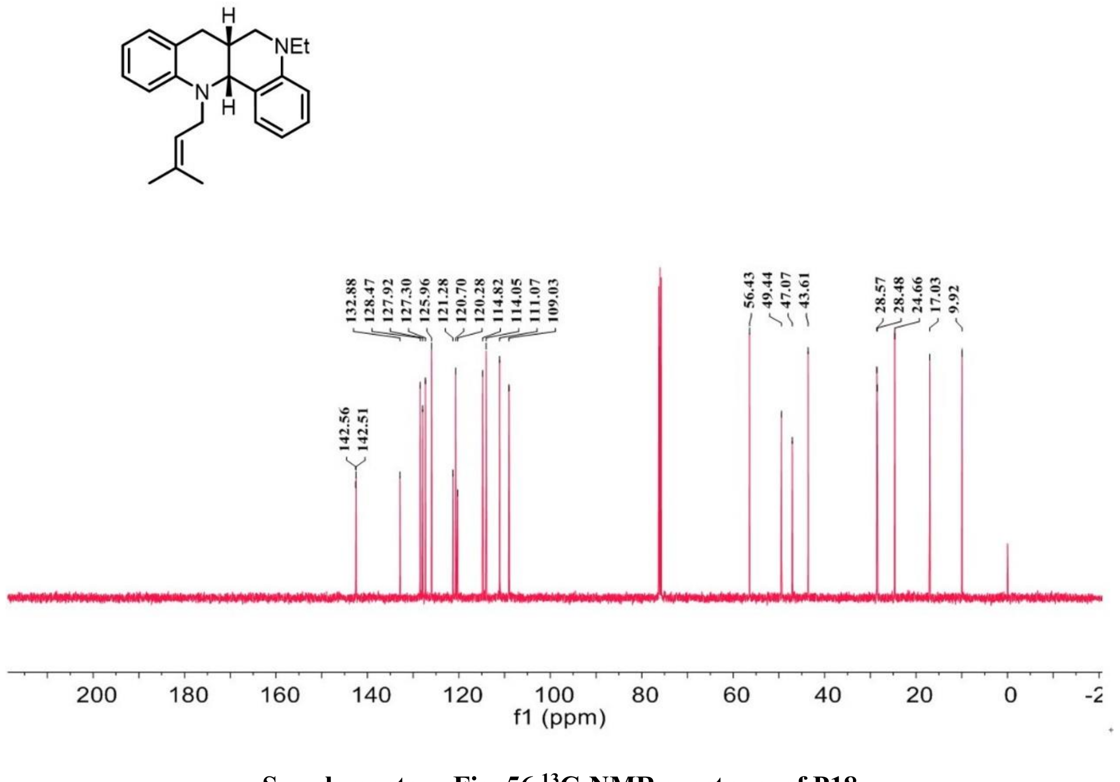

Supplementary Fig. 56 ^{13}C-NMR spectrum of P18

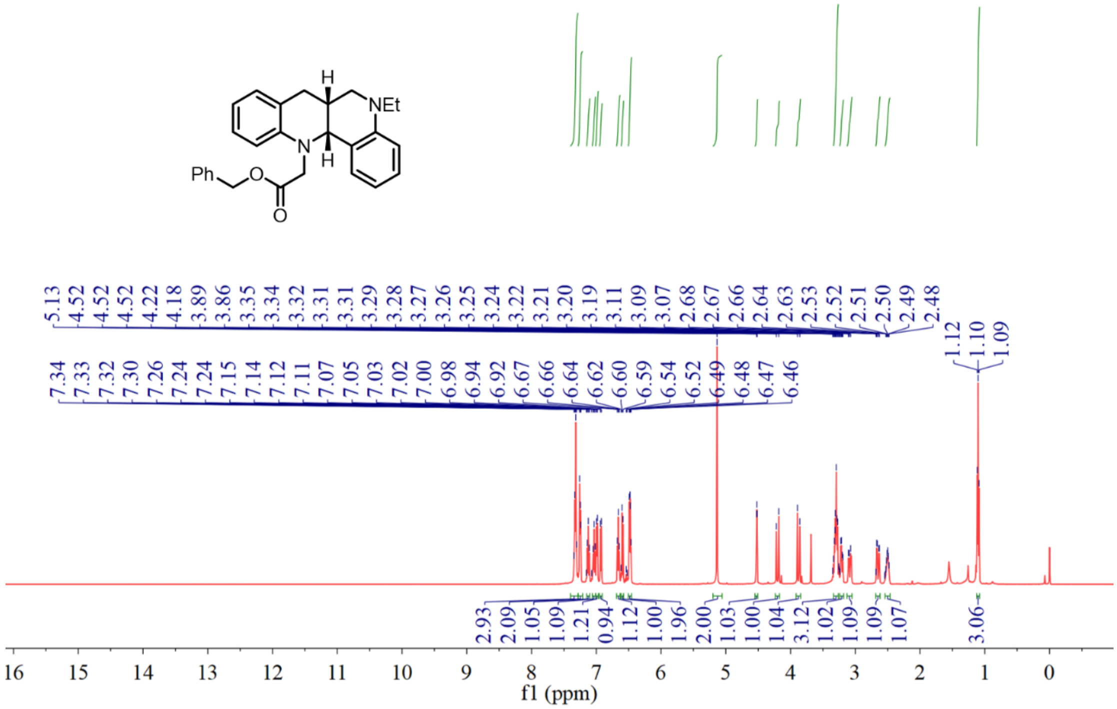

Supplementary Fig. 57 ^{1}H-NMR spectrum of P19

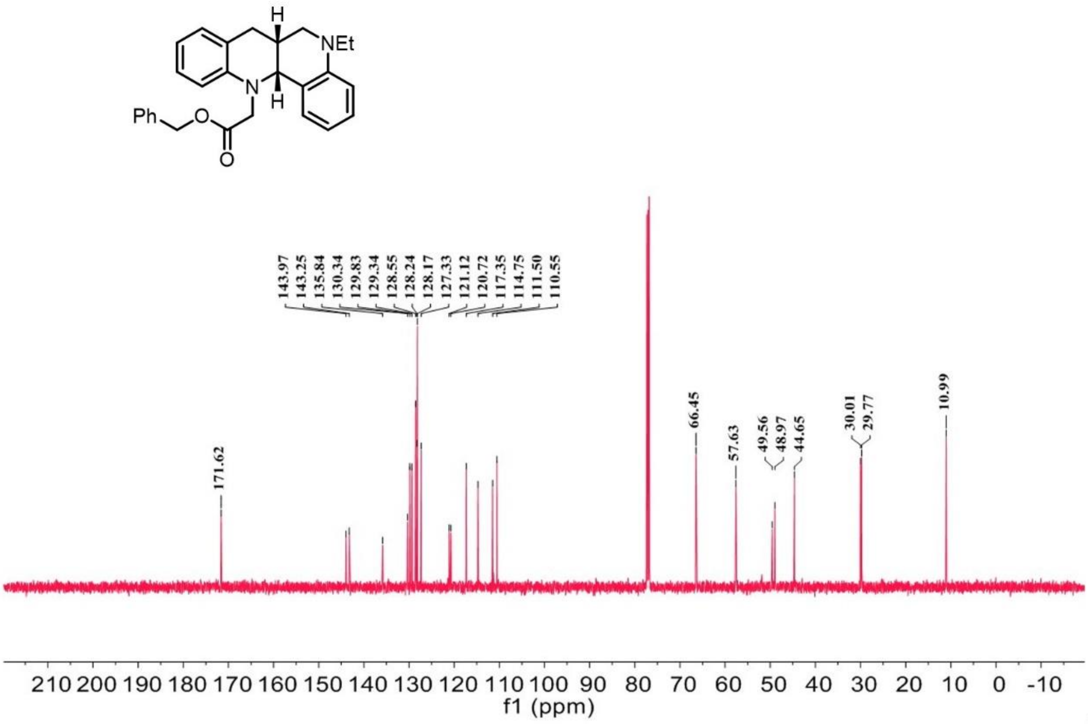

Supplementary Fig. 58 ^{13}C-NMR spectrum of P19

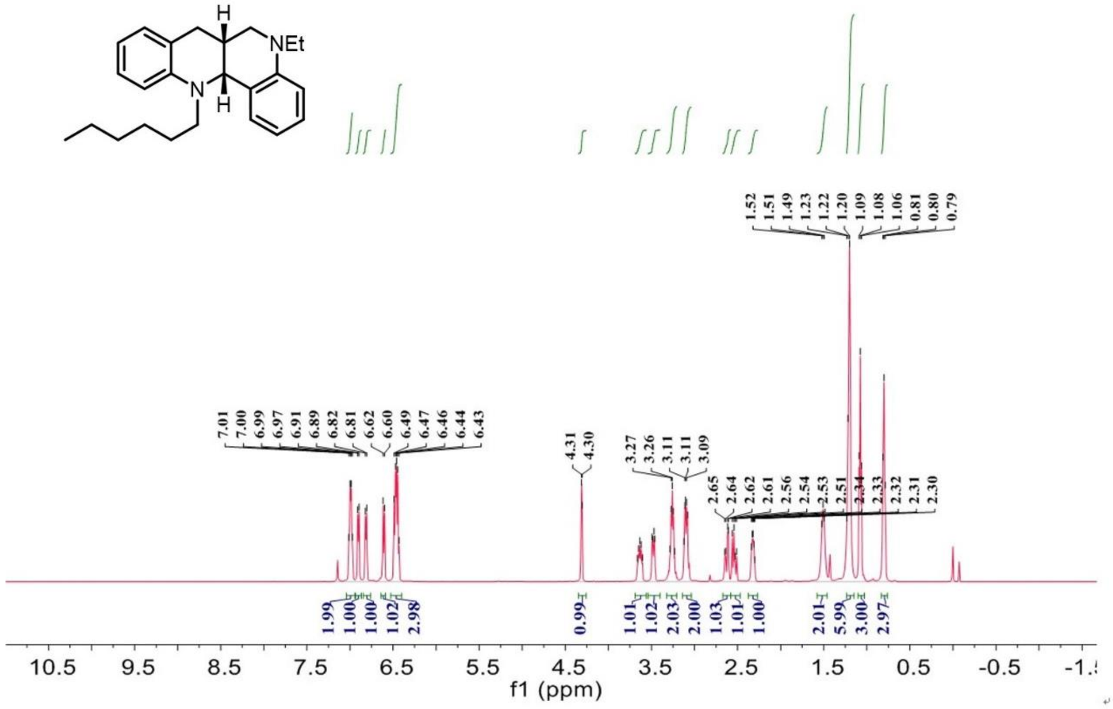

Supplementary Fig. 59 ^{1}H-NMR spectrum of P20

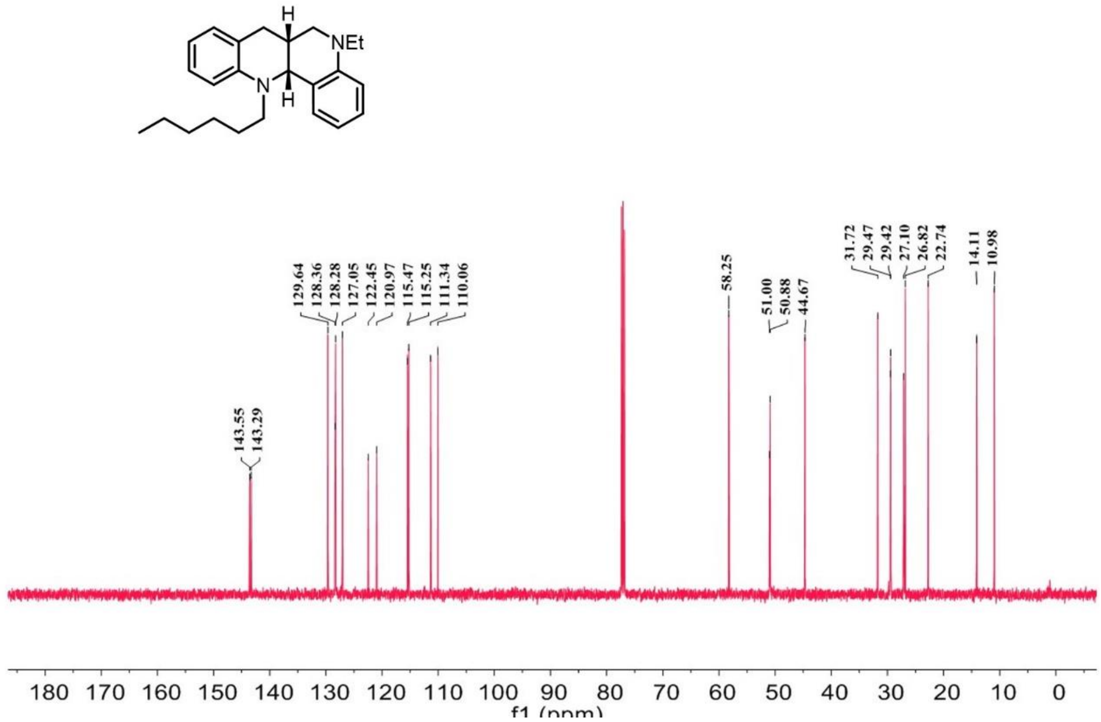

Supplementary Fig. 60 ^{13}C-NMR spectrum of P20

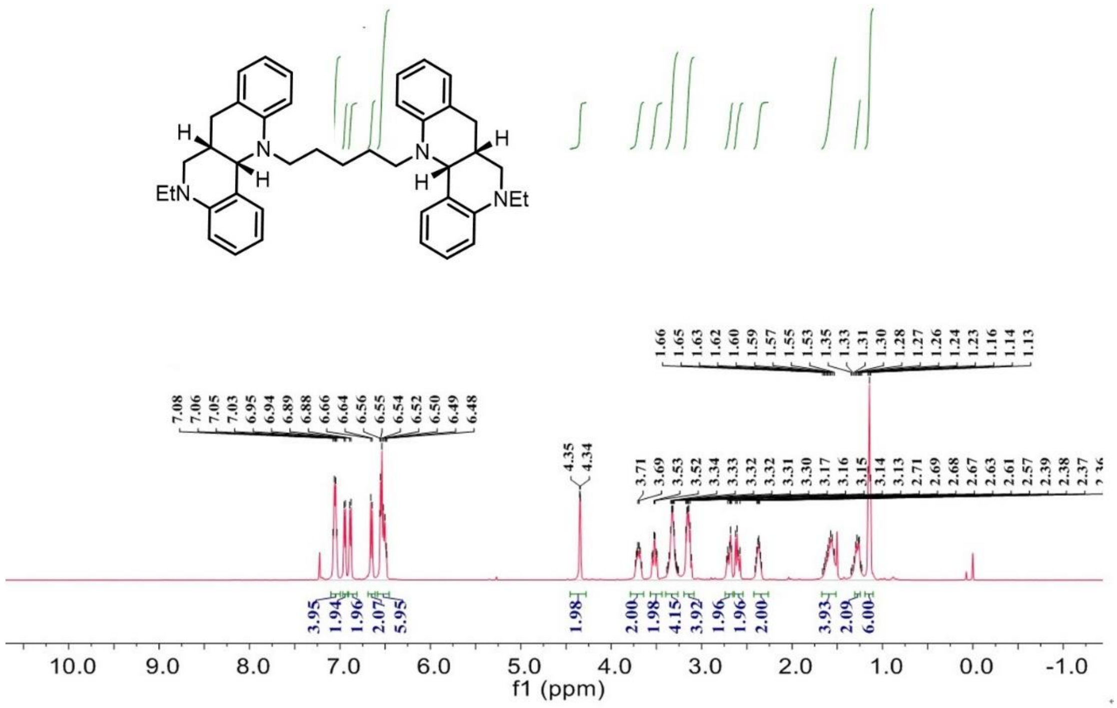

Supplementary Fig. 61 ^{1}H-NMR spectrum of P21

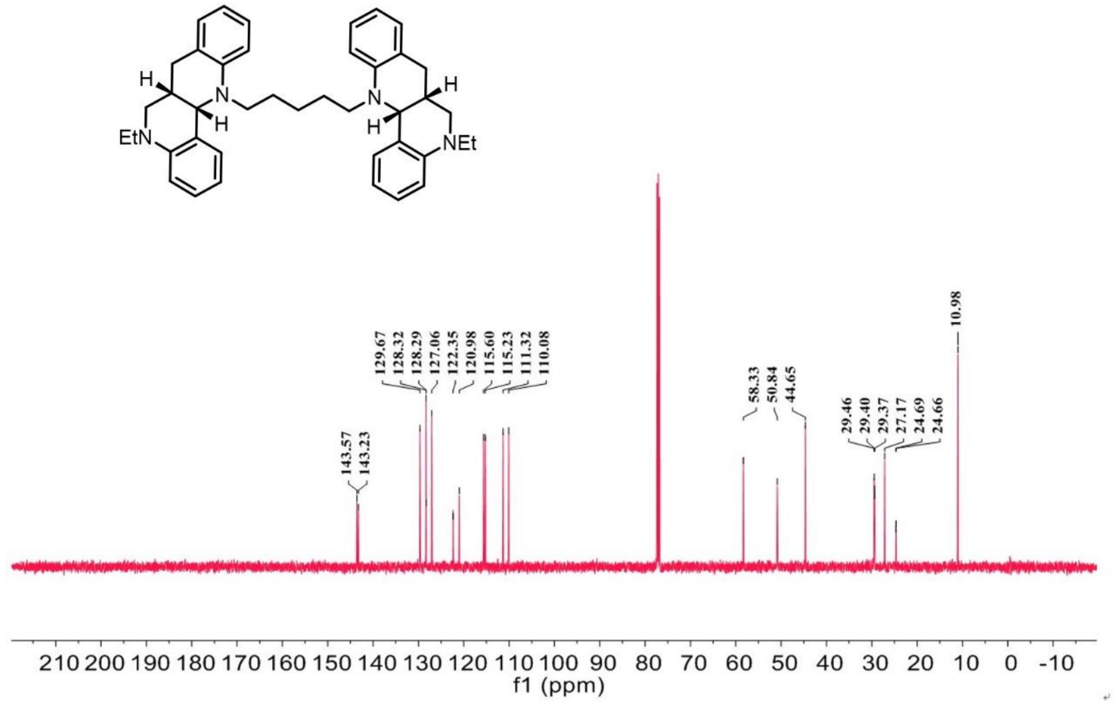

Supplementary Fig. 62 ^{13}C-NMR spectrum of P21

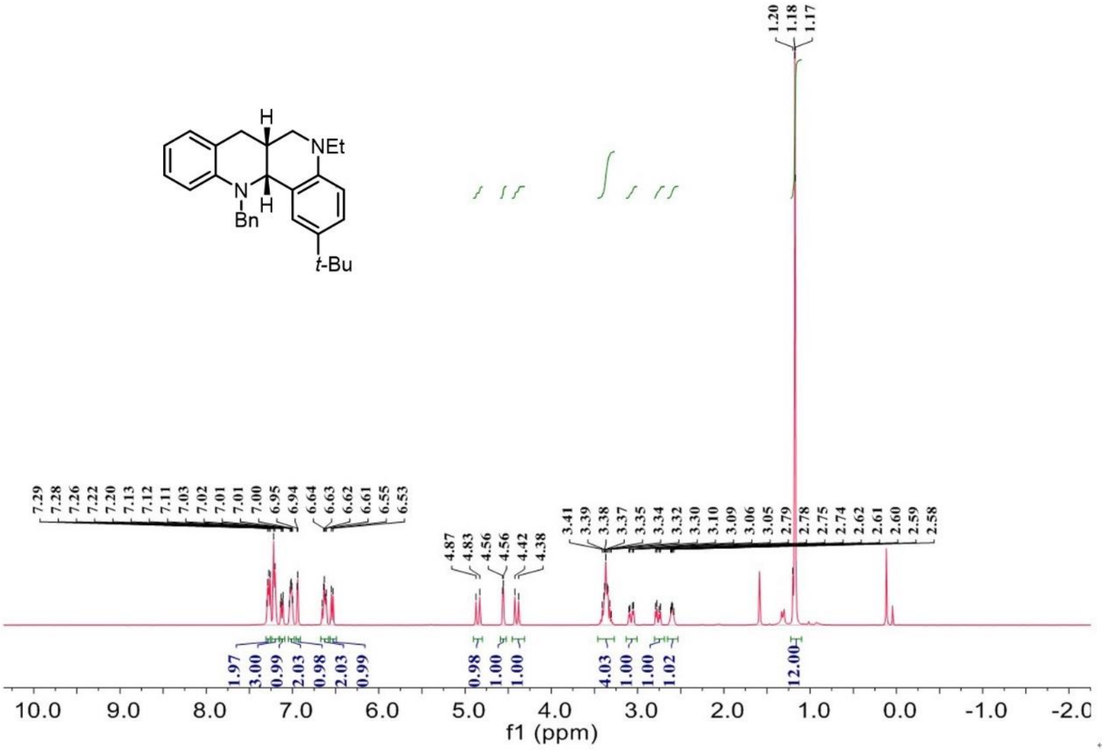

Supplementary Fig. 63 ^{1}H-NMR spectrum of P22

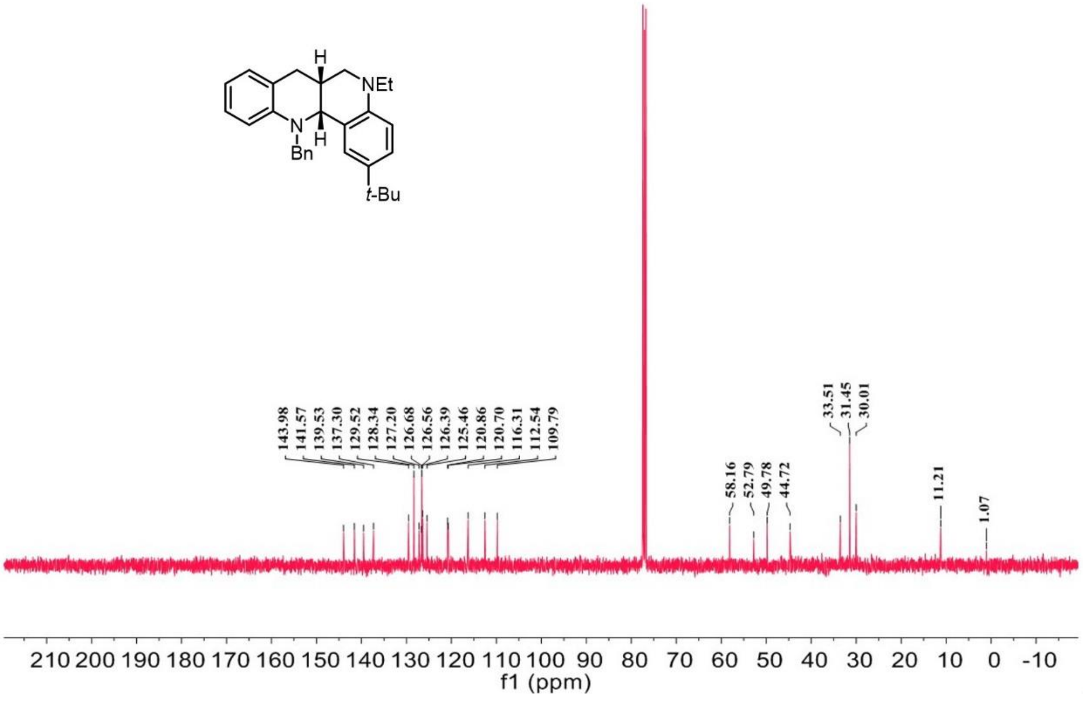

Supplementary Fig. 64 ^{13}C-NMR spectrum of P22

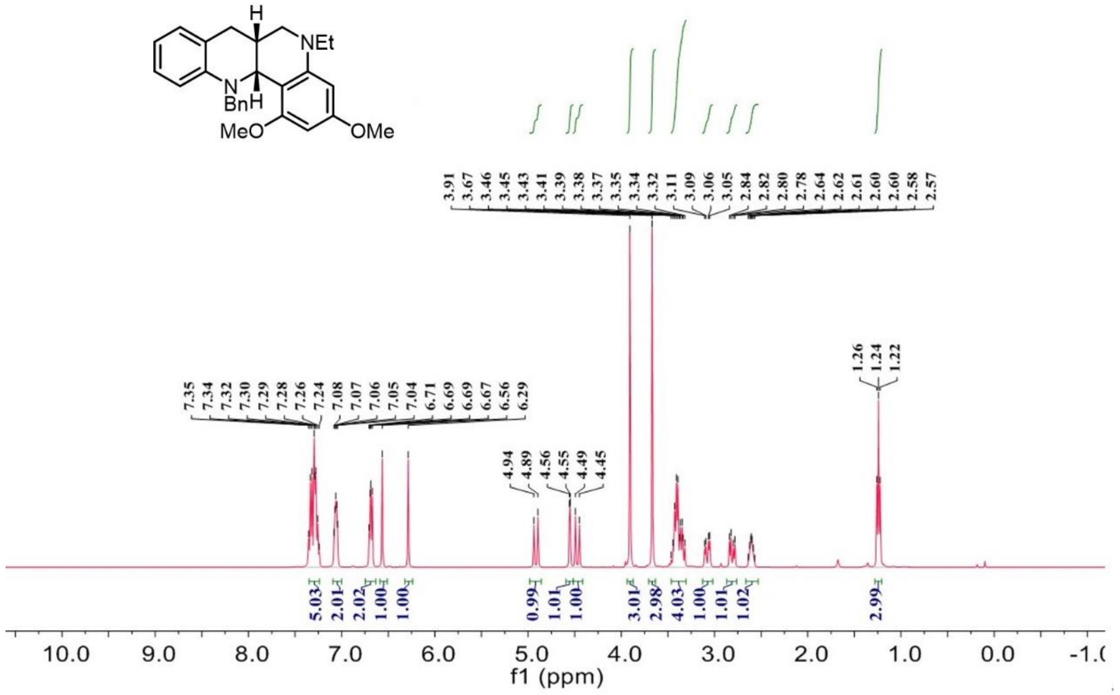

Supplementary Fig. 65 ^{1}H-NMR spectrum of P23

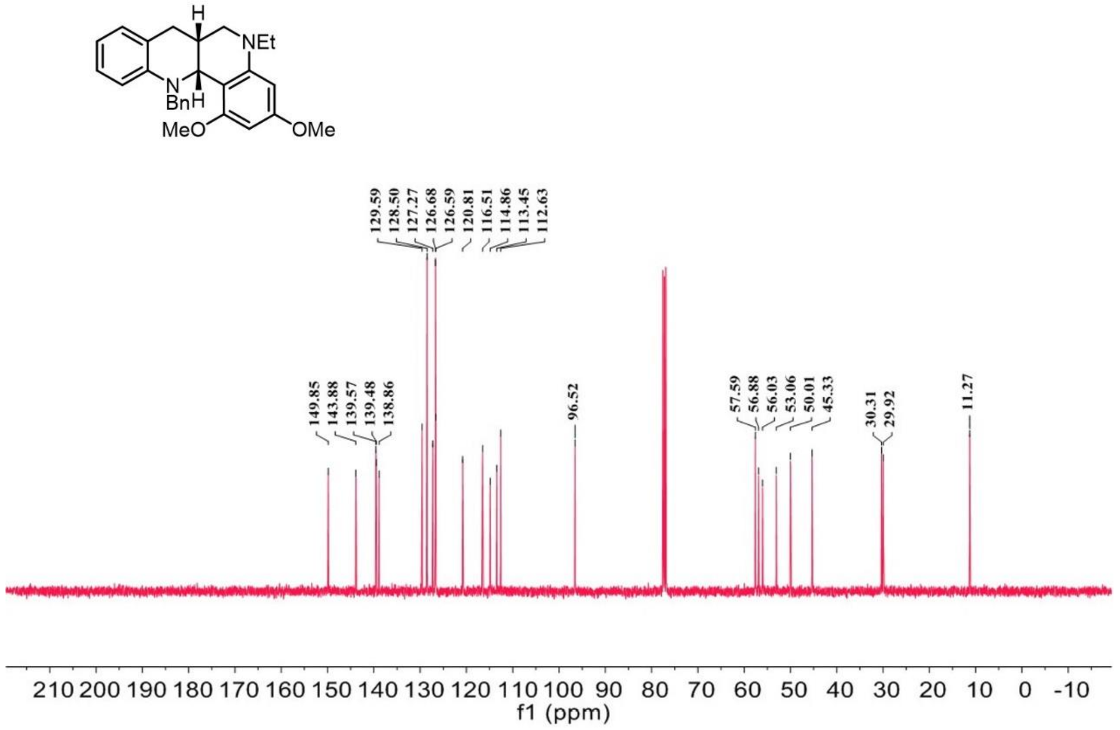

Supplementary Fig. 66 ^{13}C-NMR spectrum of P23

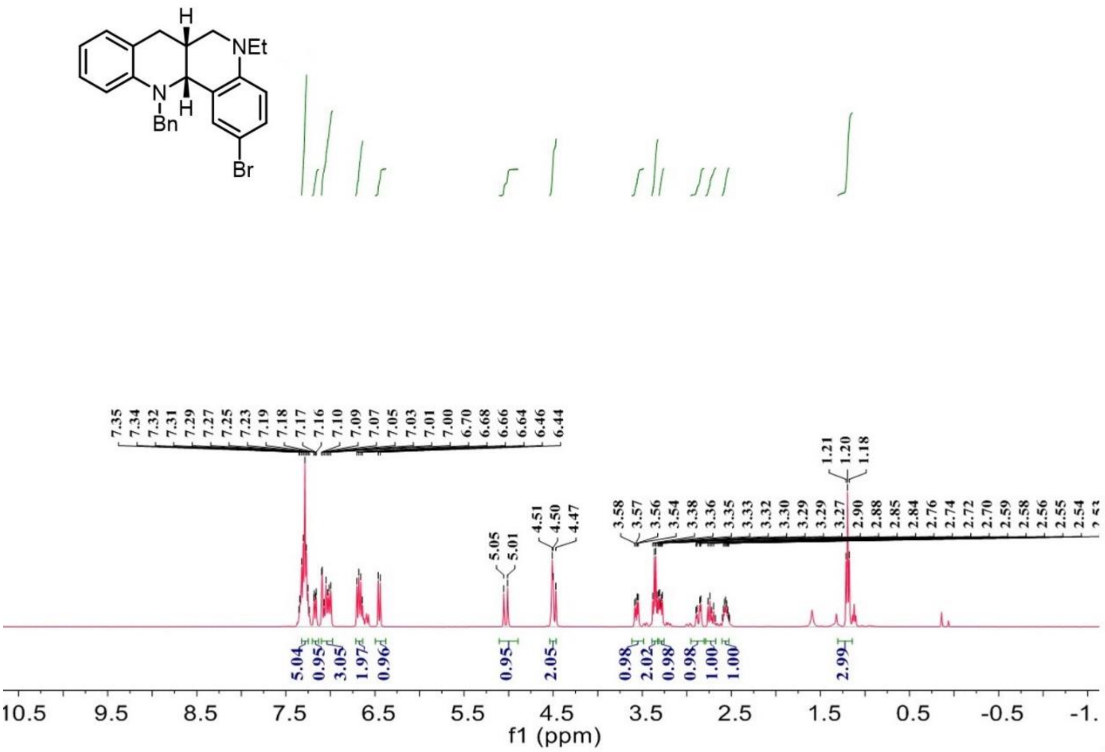

Supplementary Fig. 67 ^{1}H-NMR spectrum of P24

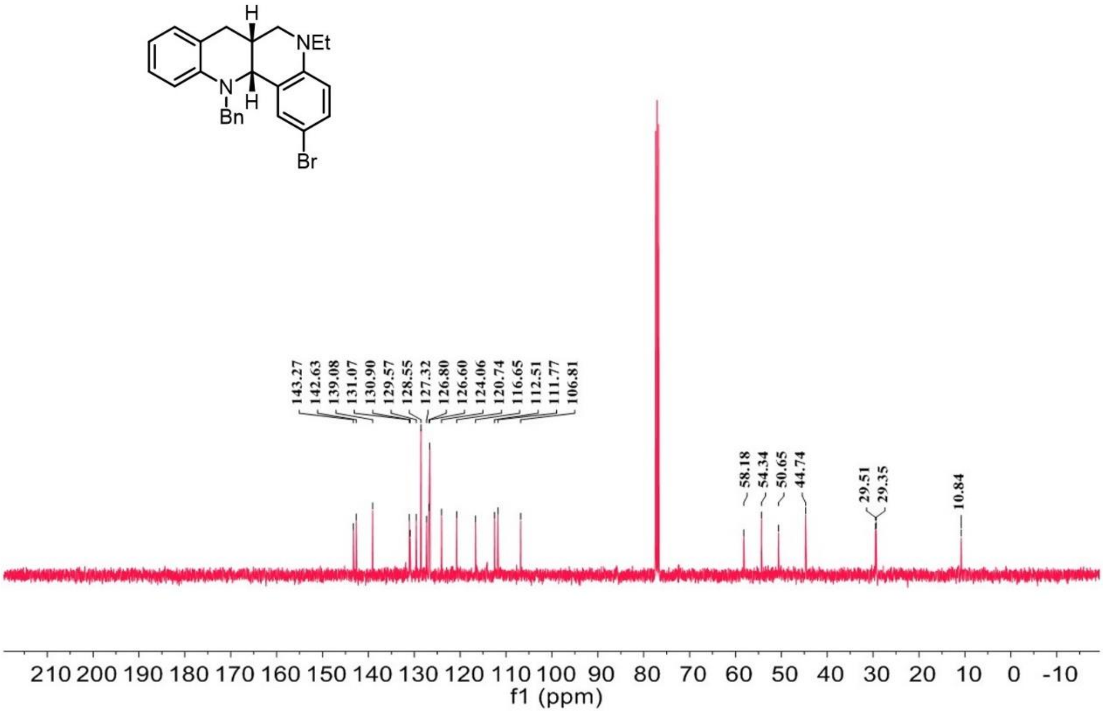

Supplementary Fig. 68 ^{13}C-NMR spectrum of P24

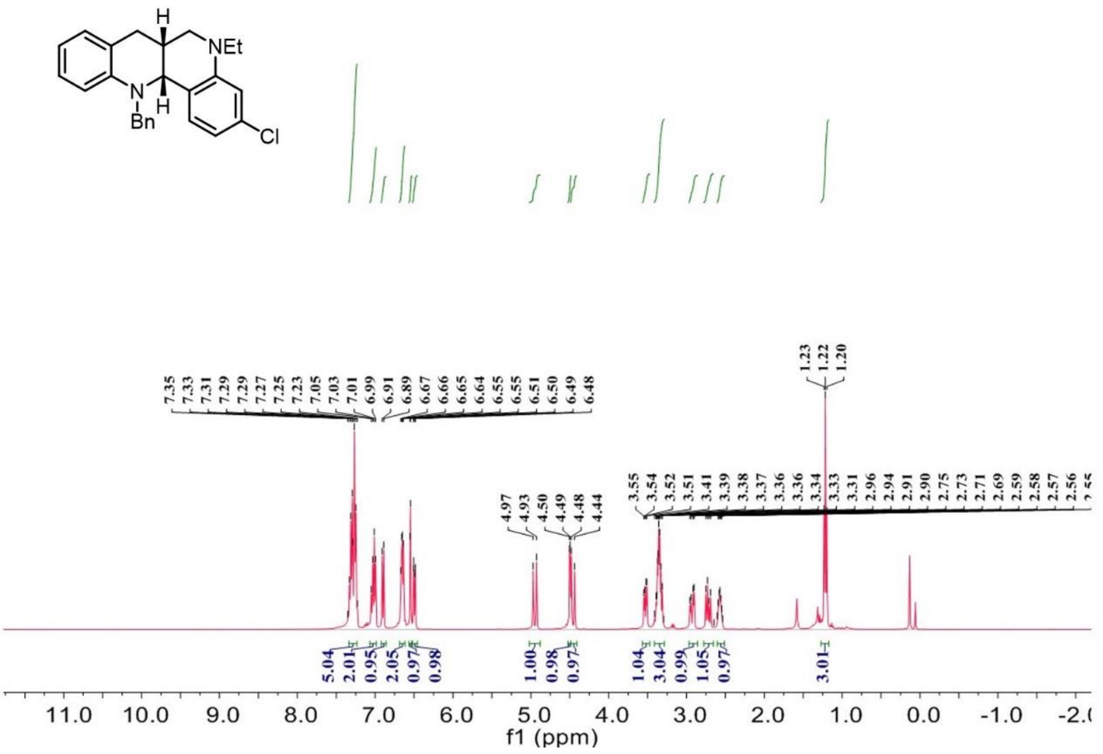

Supplementary Fig. 69 ^{1}H-NMR spectrum of P25

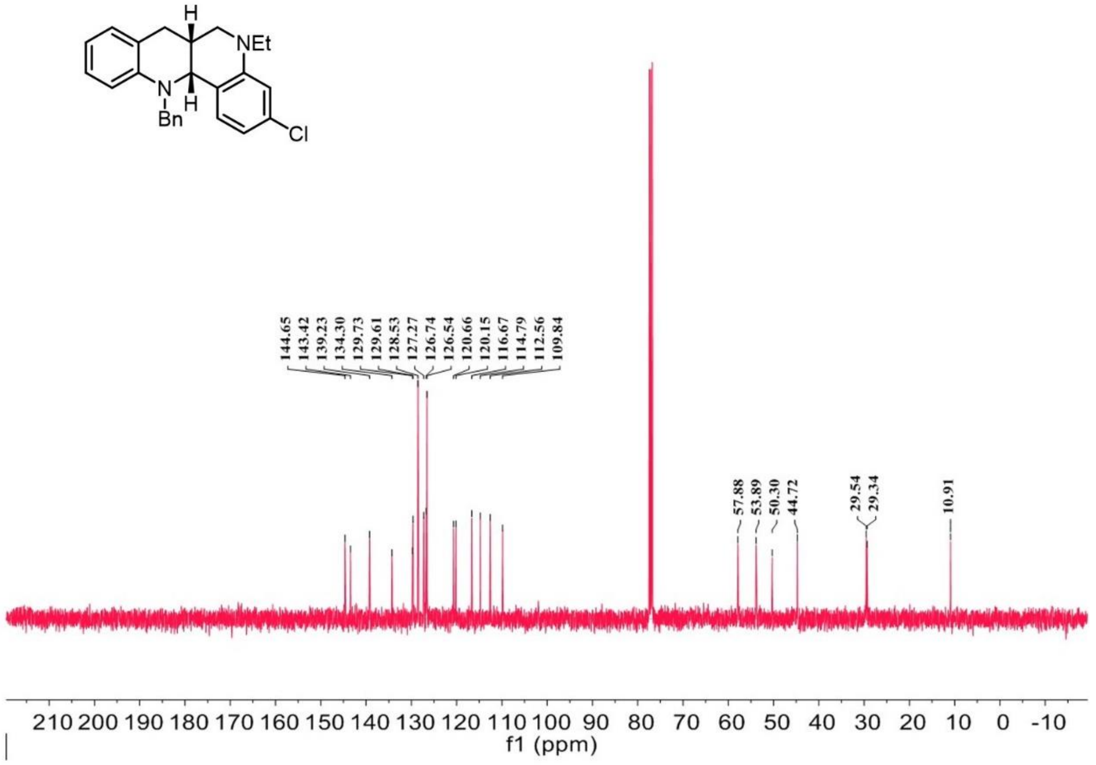

Supplementary Fig. 70 ^{13}C-NMR spectrum of P25

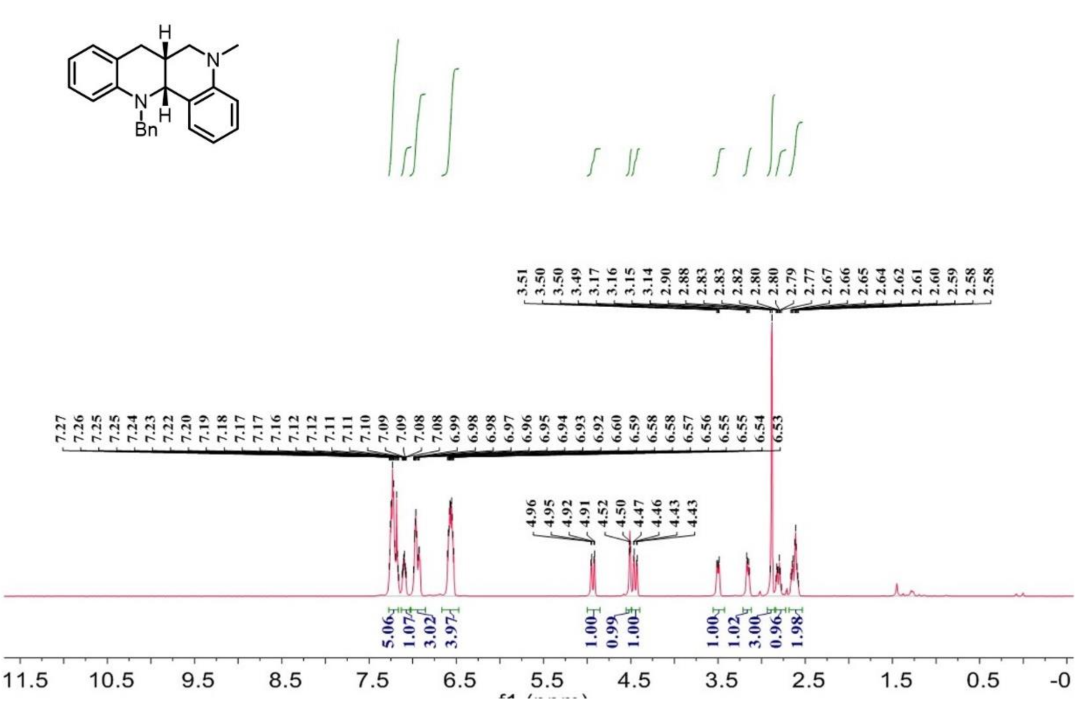

Supplementary Fig. 71 ^{1}H-NMR spectrum of P26

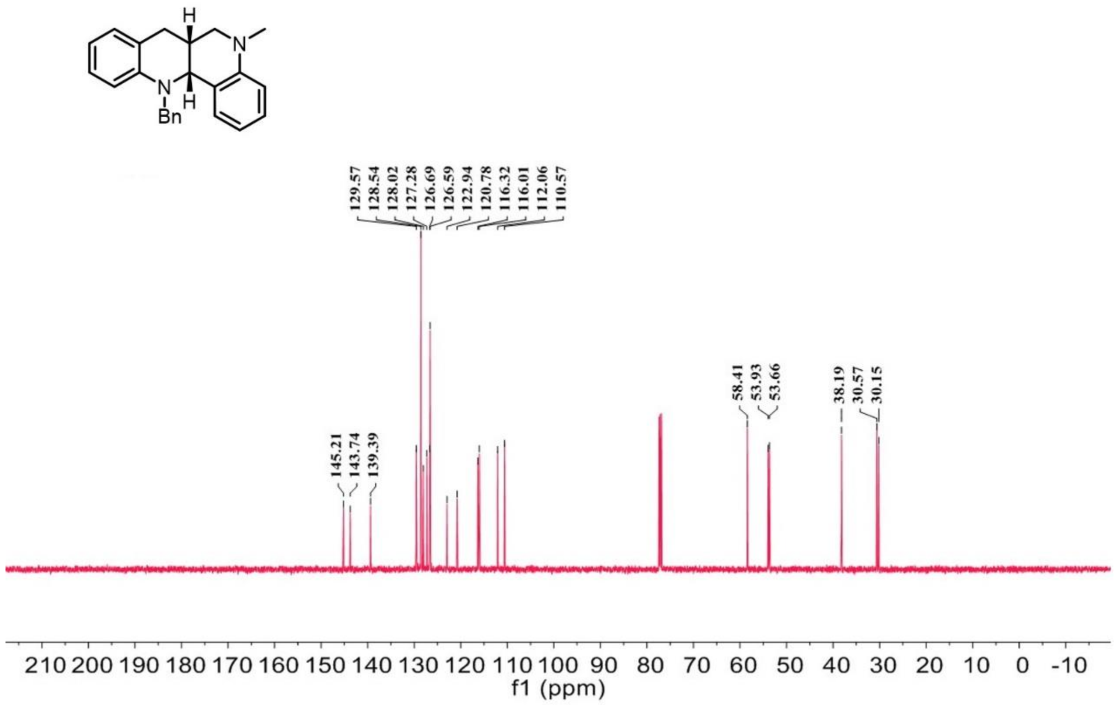

Supplementary Fig. 72 ^{13}C-NMR spectrum of P26

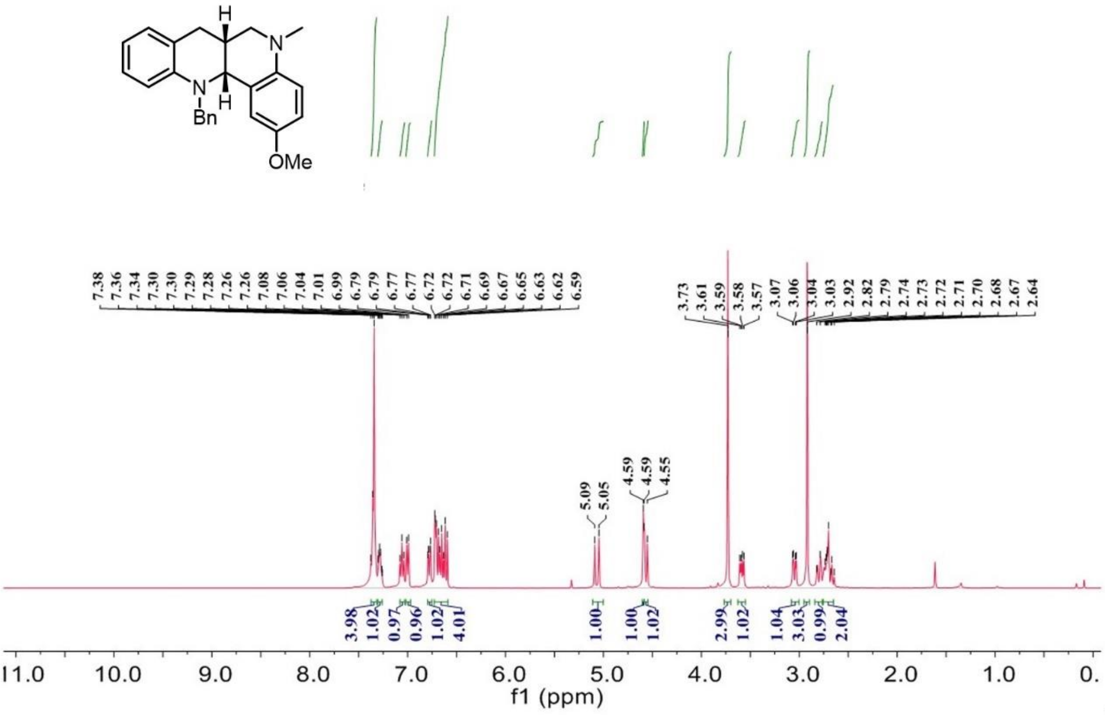

Supplementary Fig. 73 ^{1}H-NMR spectrum of P27

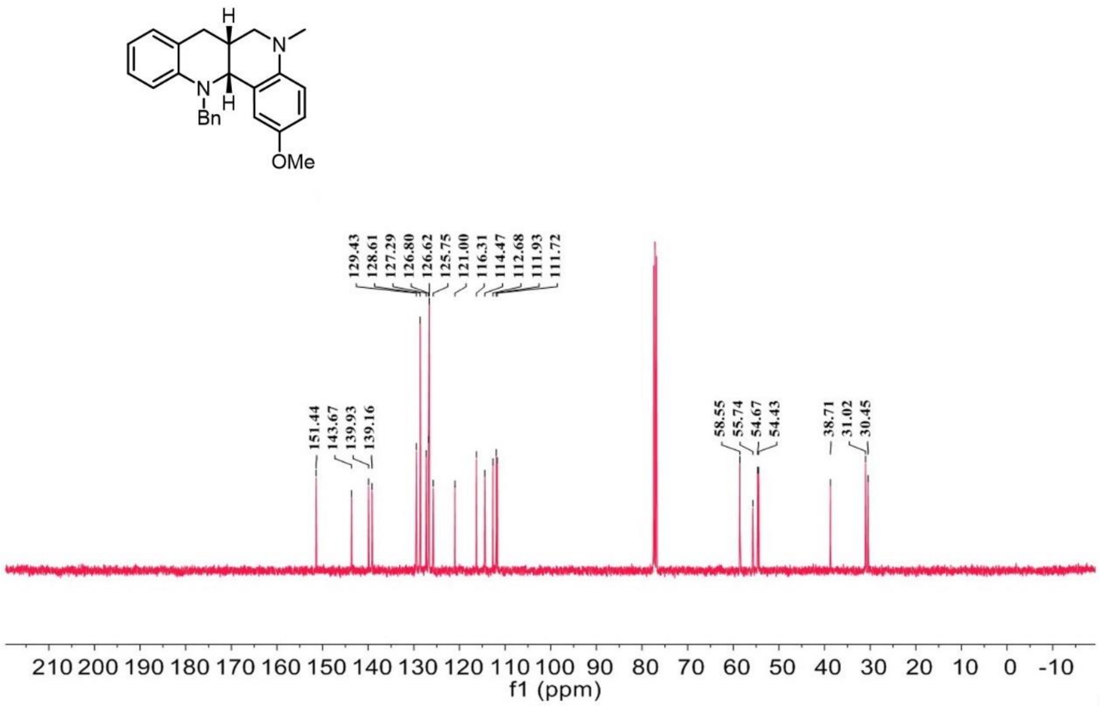

Supplementary Fig. 74 ^{13}C-NMR spectrum of P27

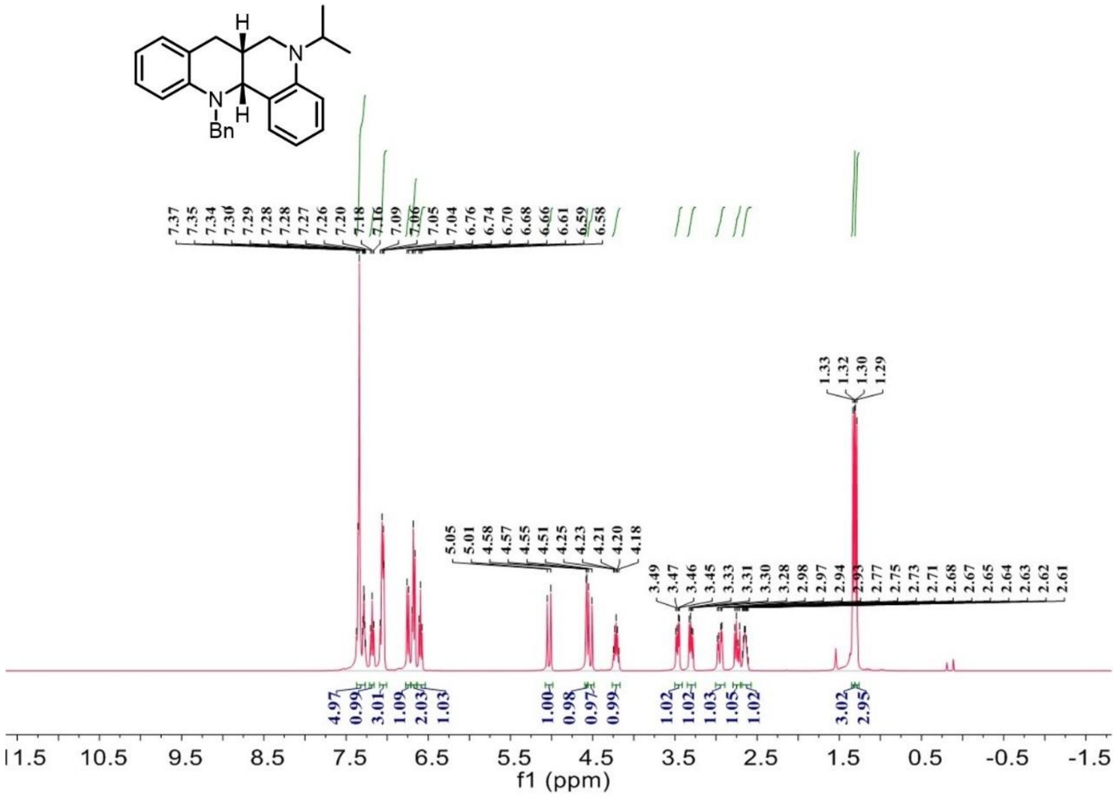

Supplementary Fig. 75 ^{1}H-NMR spectrum of P28

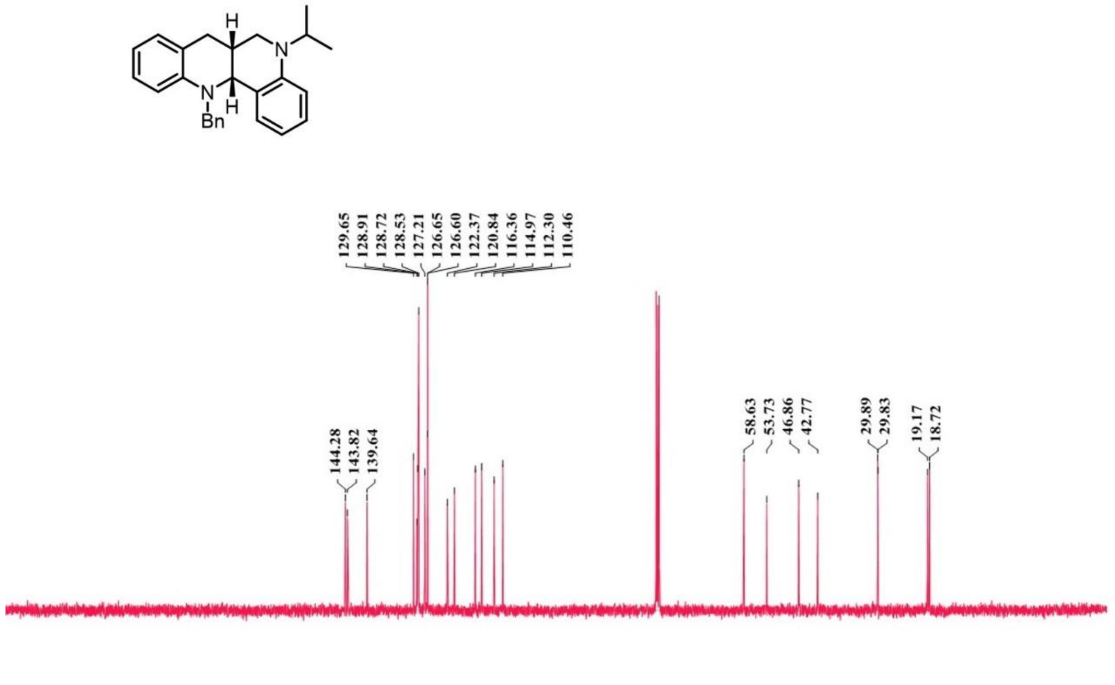

Supplementary Fig. 76 ^{13}C-NMR spectrum of P28

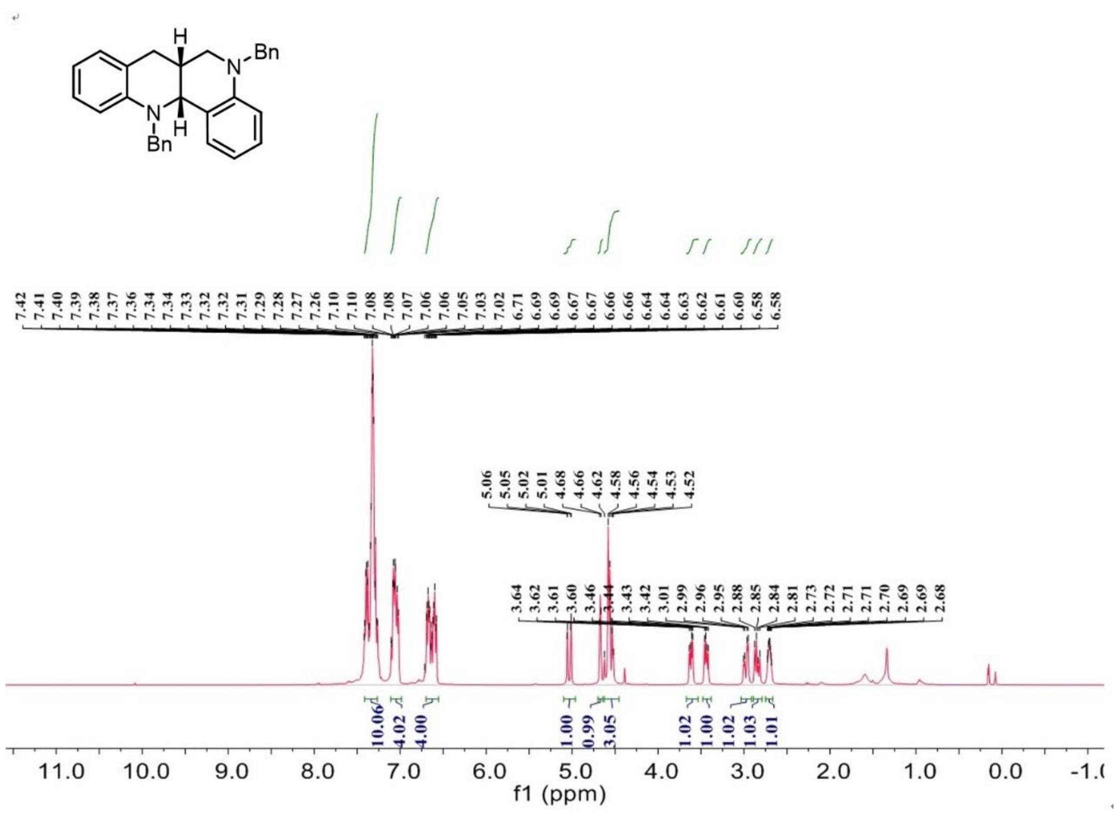

Supplementary Fig. 77 ^{1}H-NMR spectrum of P29

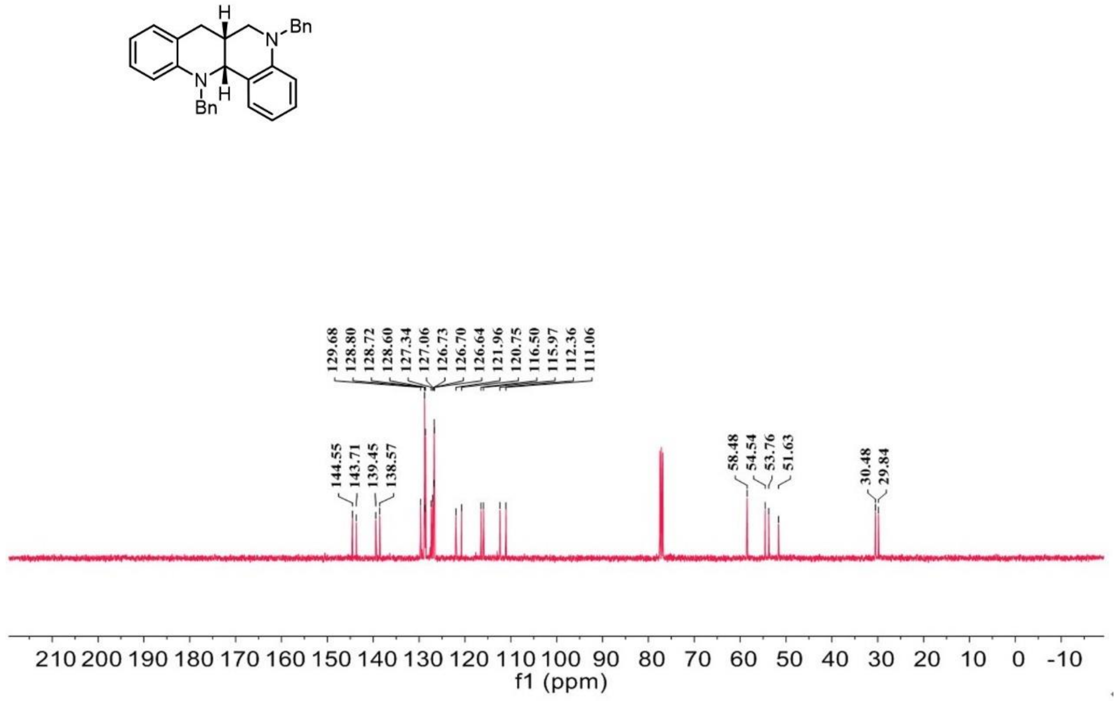

Supplementary Fig. 78 ^{13}C-NMR spectrum of P29

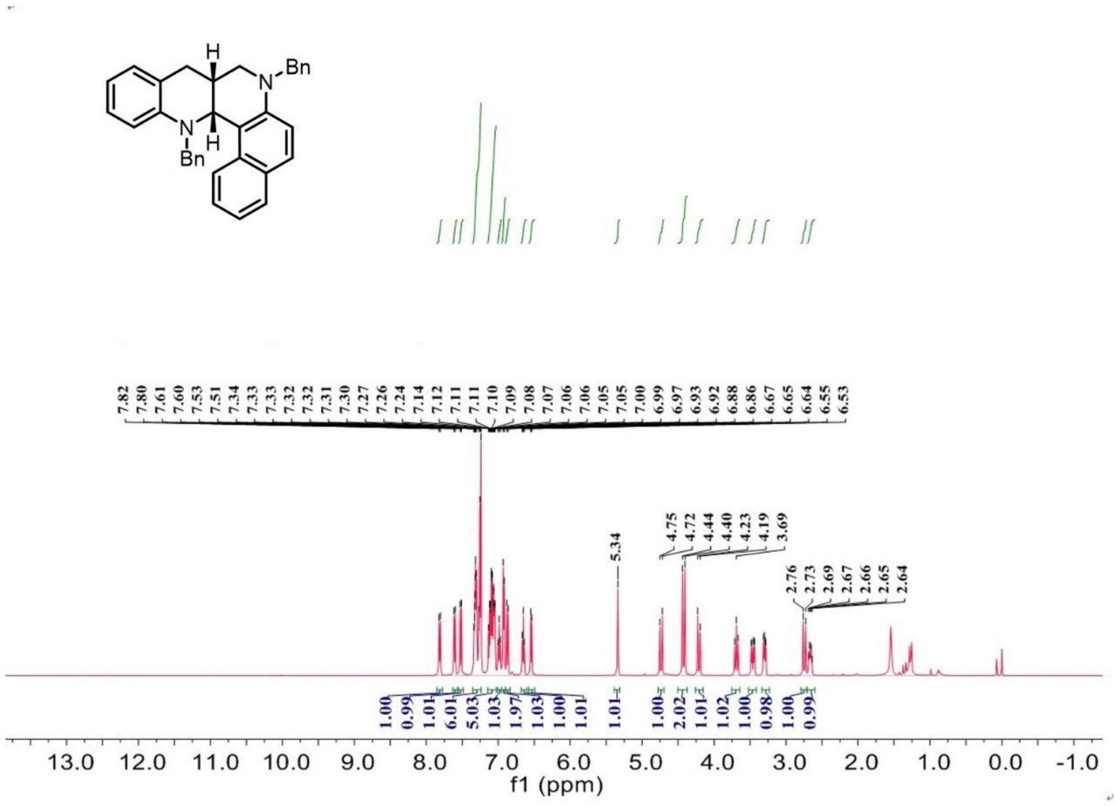

Supplementary Fig. 79 ^{1}H-NMR spectrum of P30

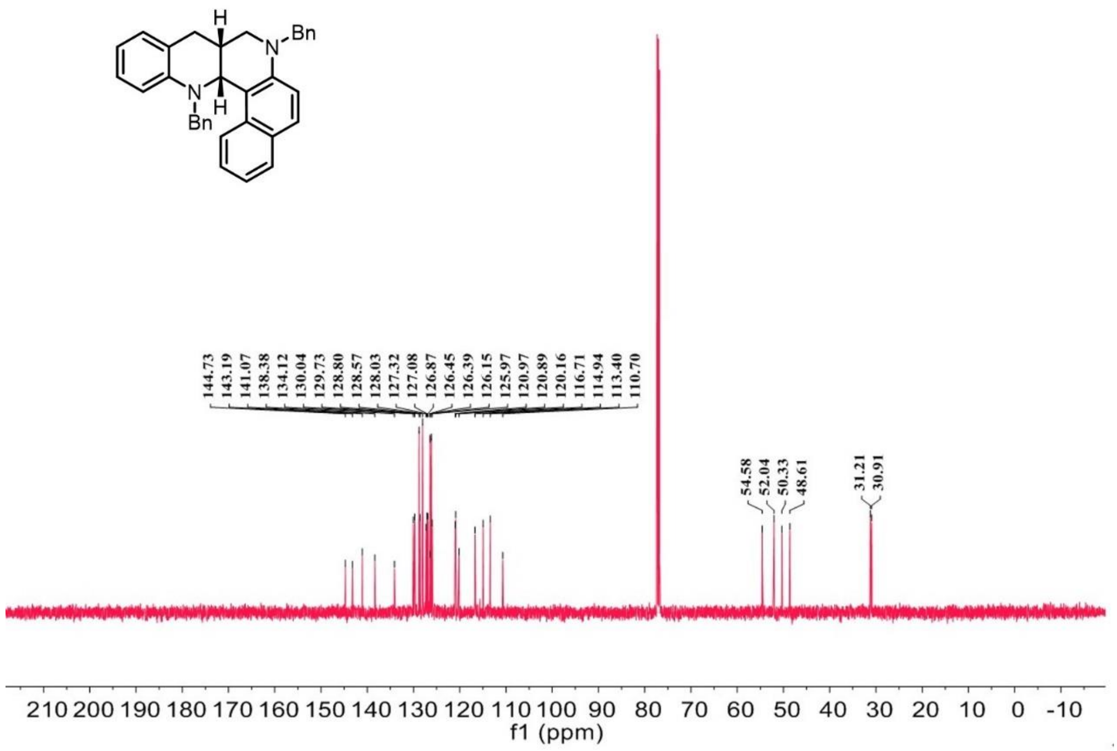

Supplementary Fig. 80 ^{13}C-NMR spectrum of P30

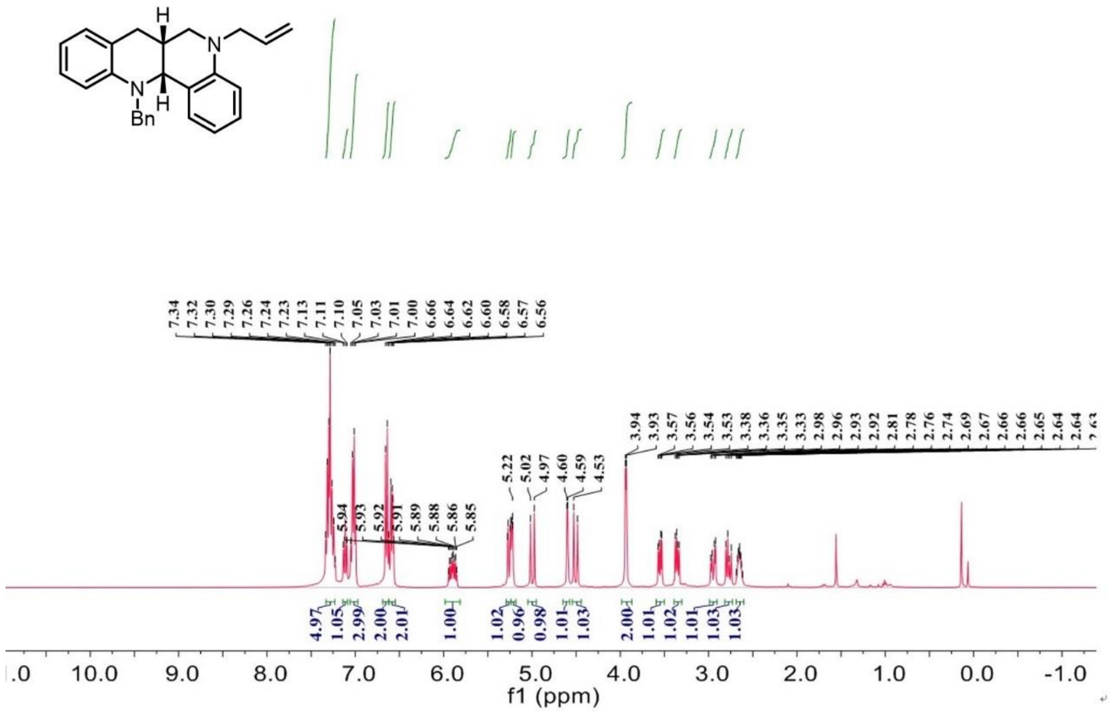

Supplementary Fig. 81 ^{1}H-NMR spectrum of P31

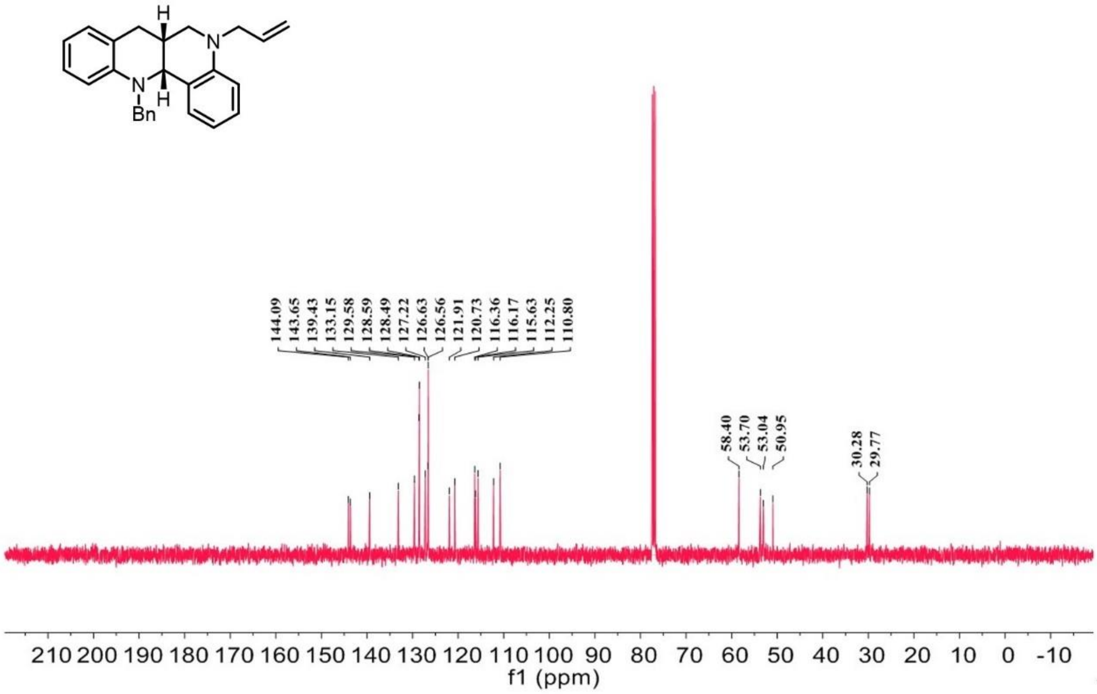

Supplementary Fig. 82 ^{13}C-NMR spectrum of P31

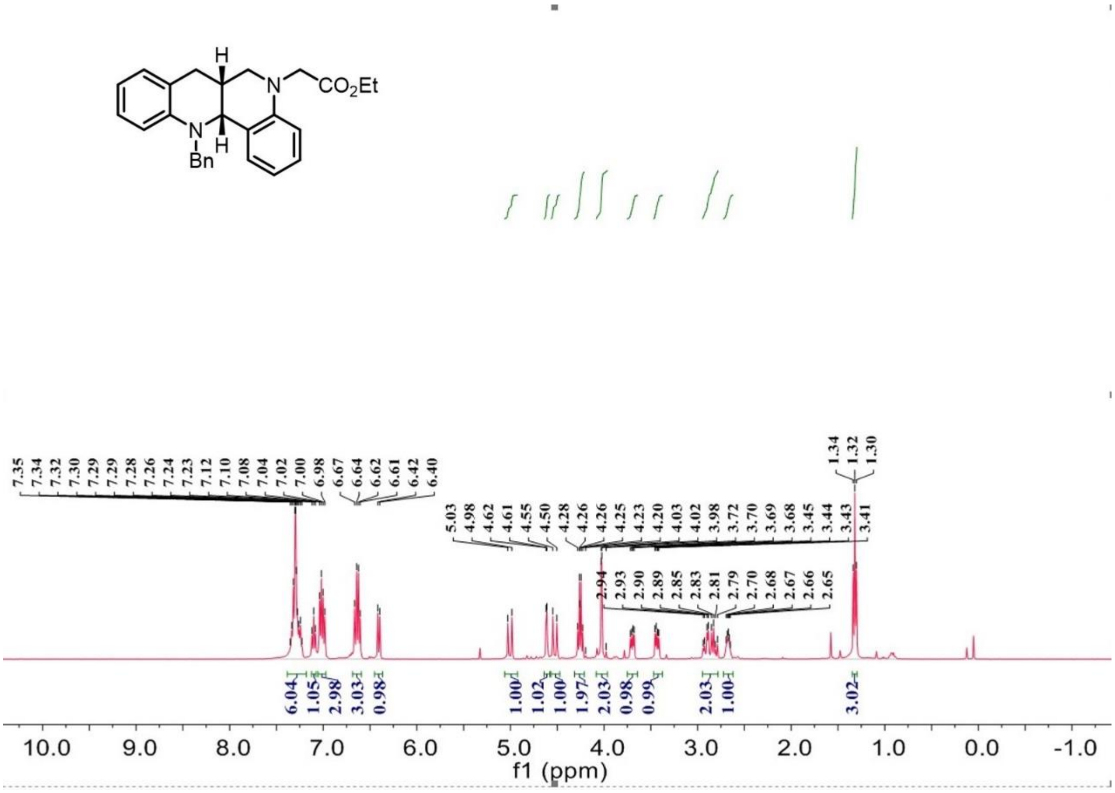

Supplementary Fig. 83 ^{1}H-NMR spectrum of P32

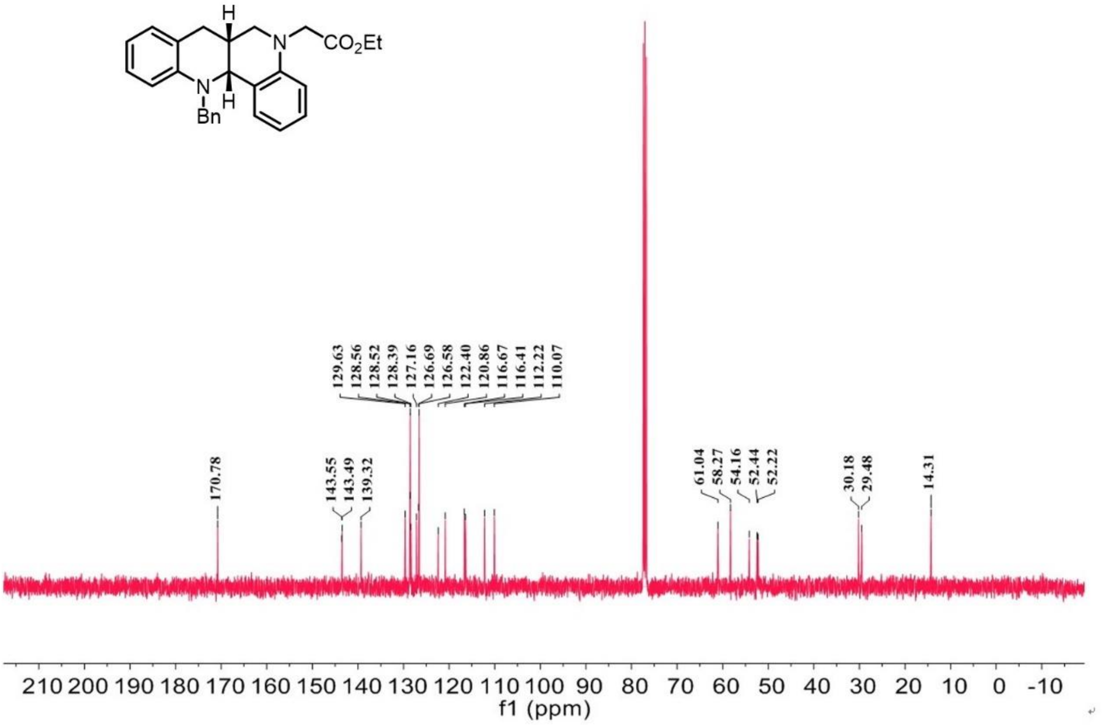

Supplementary Fig. 84 ^{13}C-NMR spectrum of P32

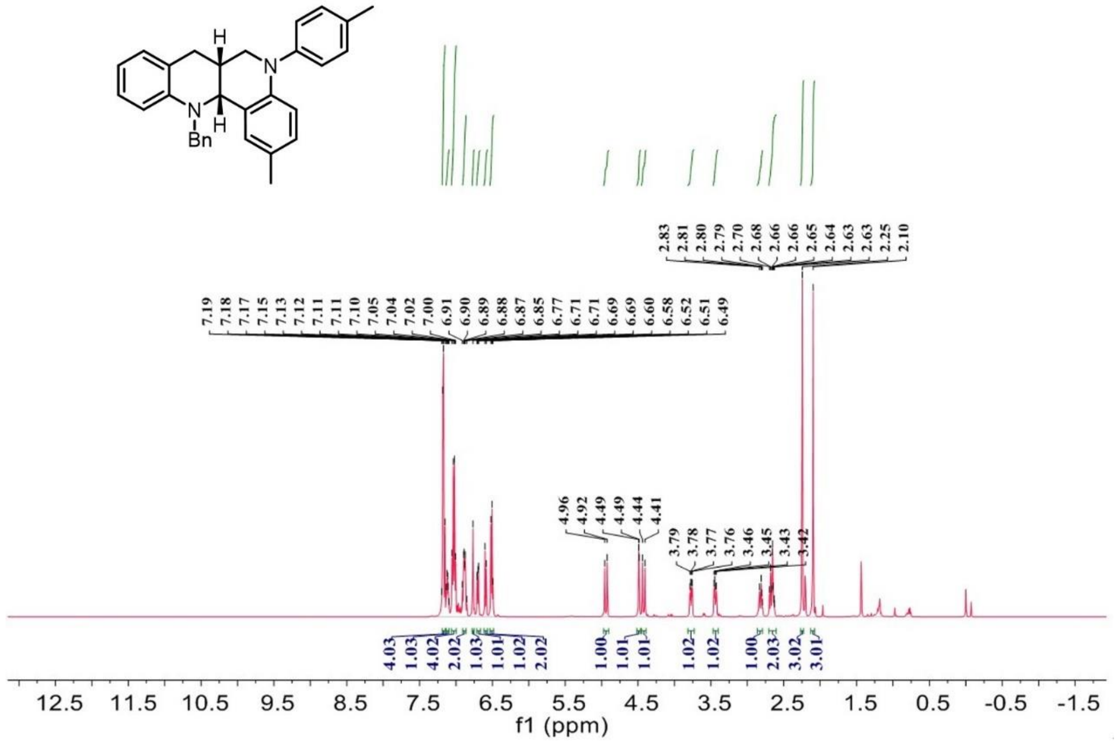

Supplementary Fig. 85 ^{1}H-NMR spectrum of P33

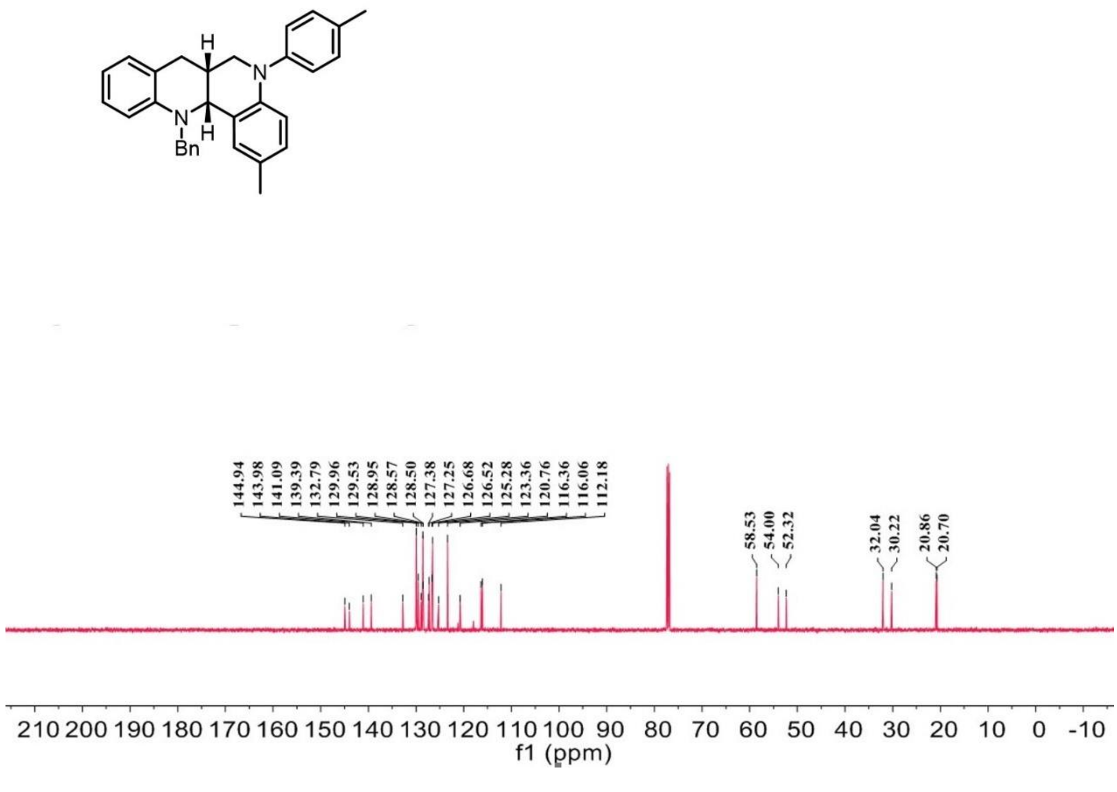

Supplementary Fig. 86 ^{13}C-NMR spectrum of P33

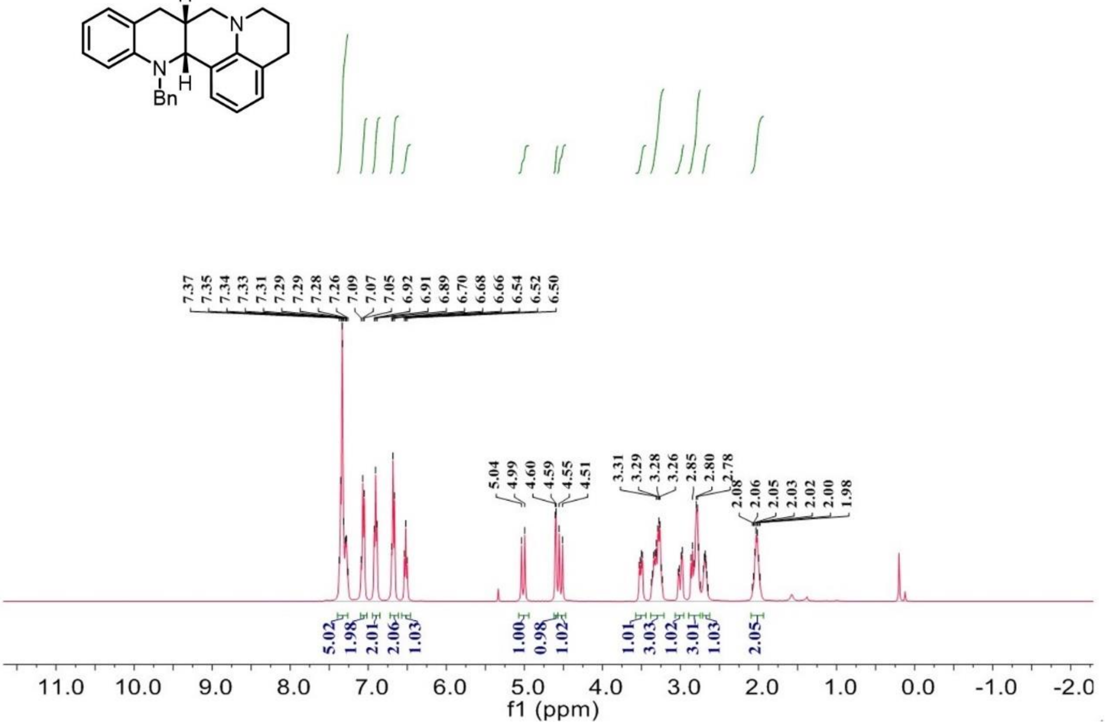

Supplementary Fig. 87 ^{1}H-NMR spectrum of P34

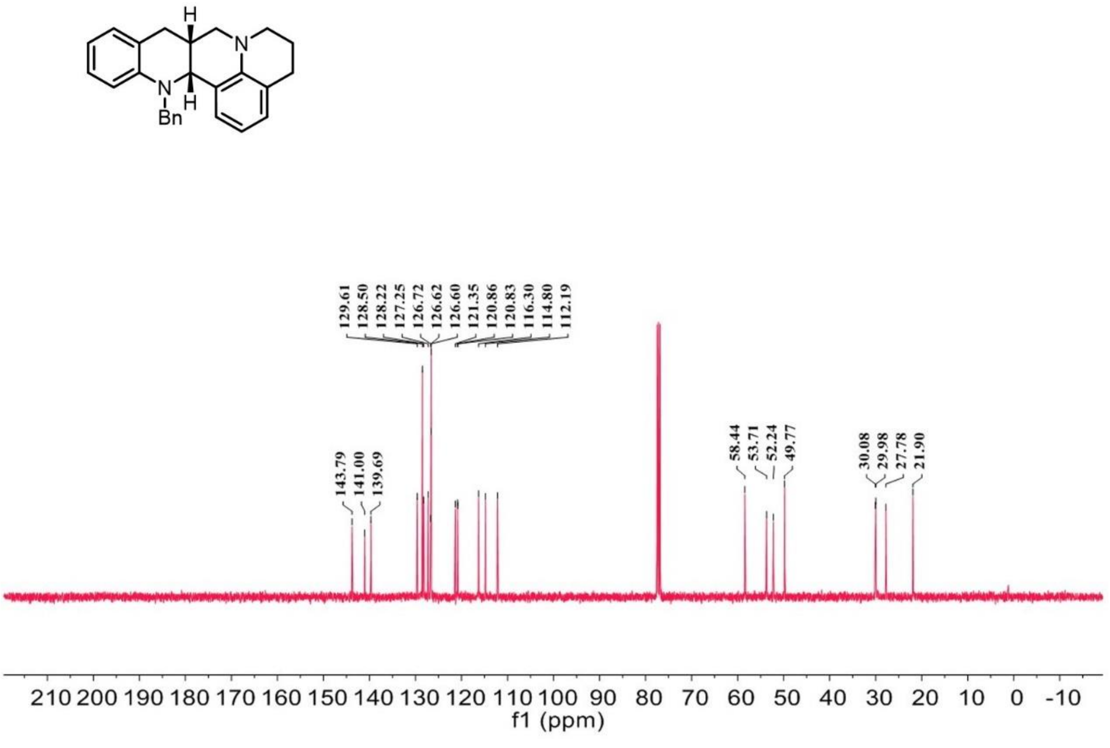

Supplementary Fig. 88 ^{13}C-NMR spectrum of P34

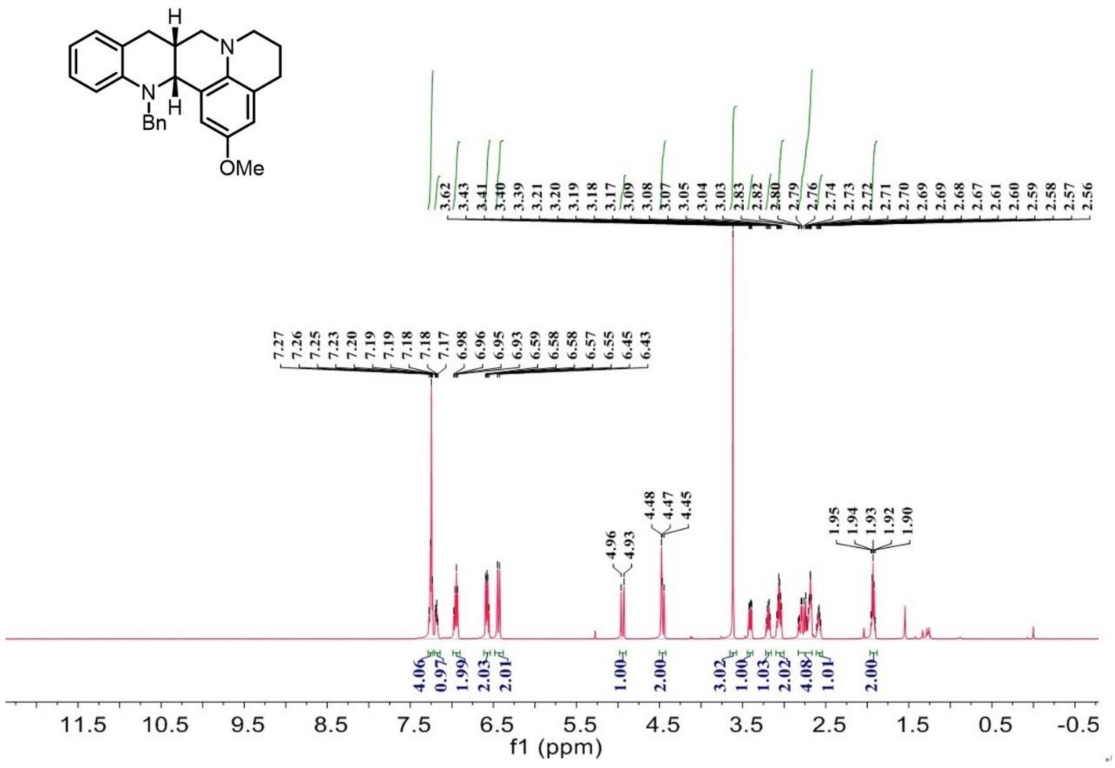

Supplementary Fig. 89 ^{1}H-NMR spectrum of P35

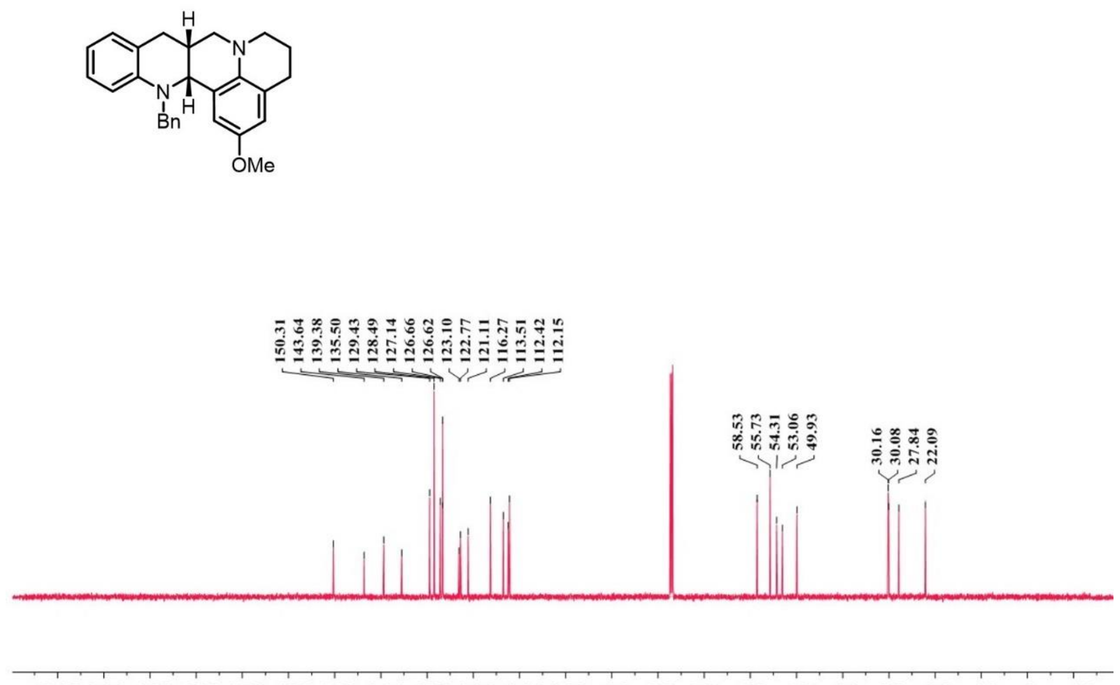

Supplementary Fig. 90 ^{13}C-NMR spectrum of P35

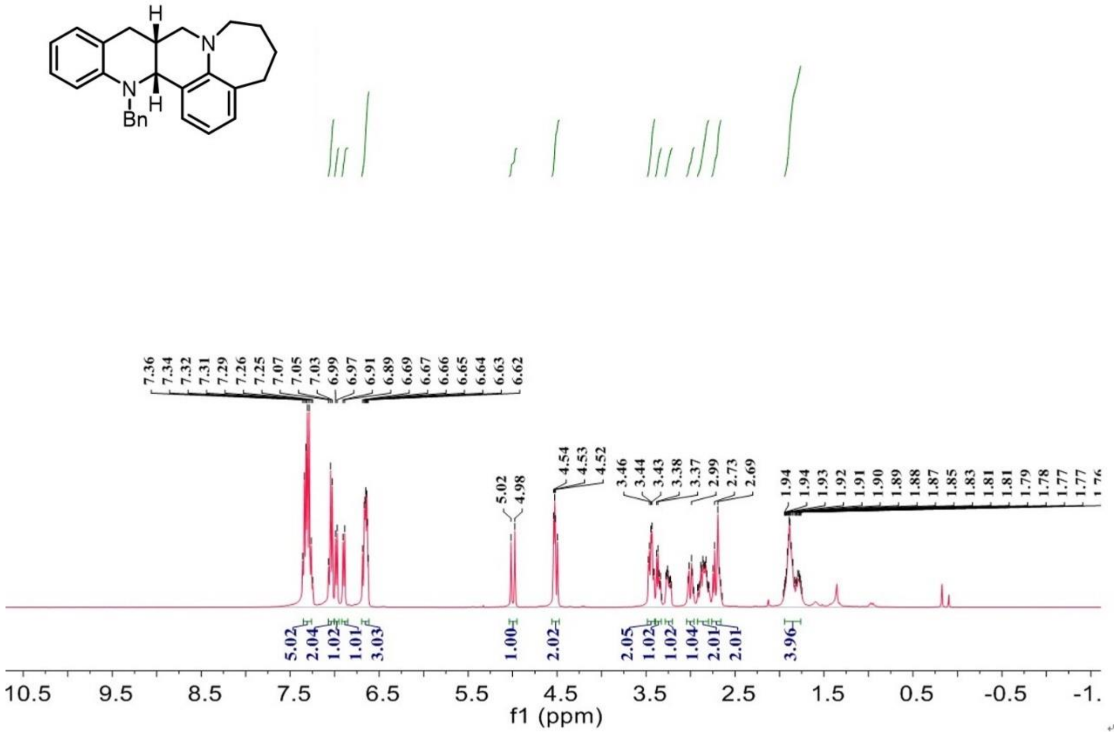

Supplementary Fig. 91 ^{1}H-NMR spectrum of P36

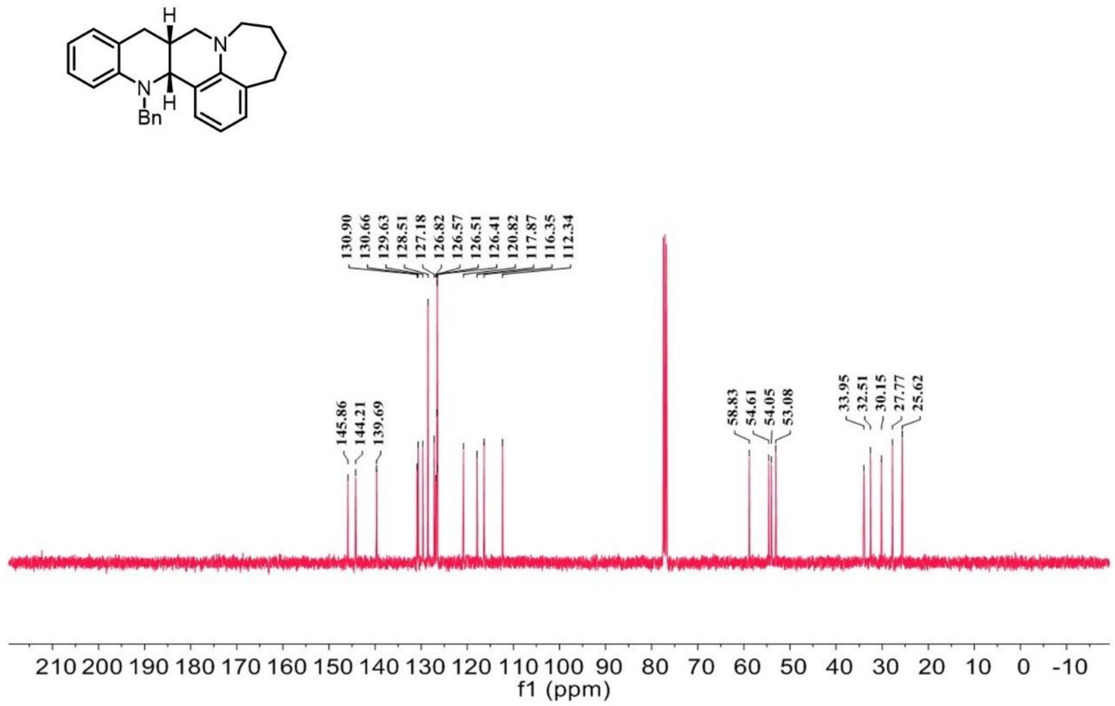

Supplementary Fig. 92 ^{13}C-NMR spectrum of P36

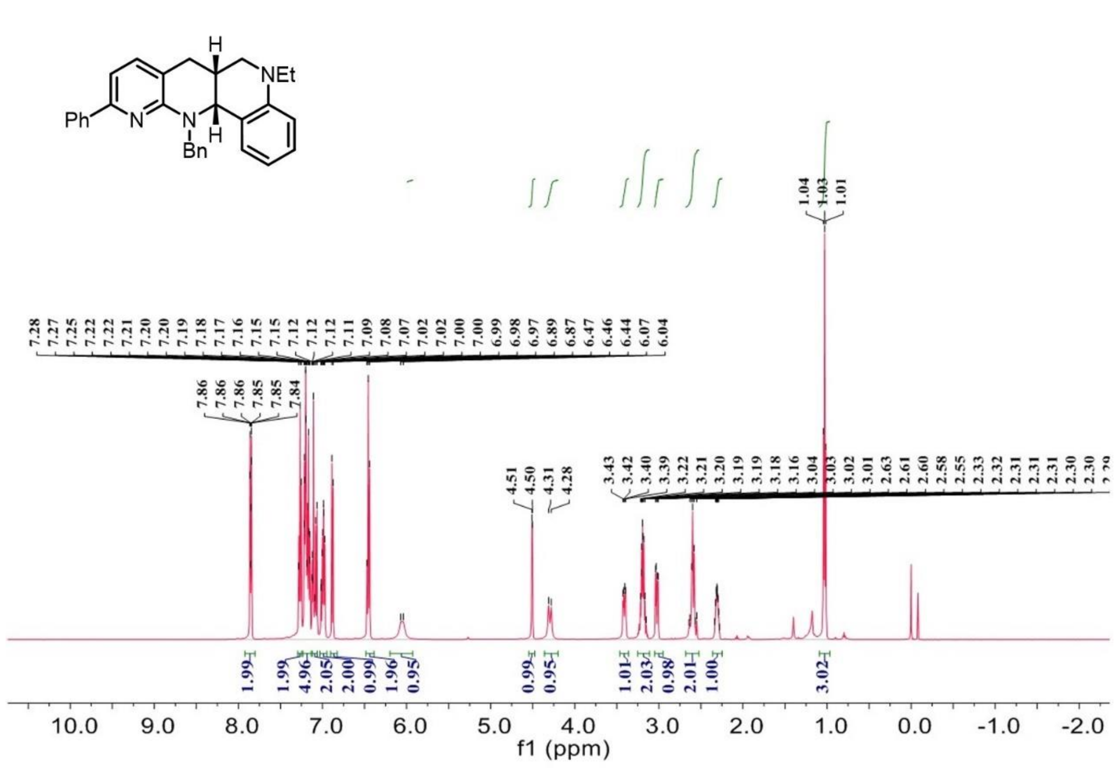

Supplementary Fig. 93 ^{1}H-NMR spectrum of P37

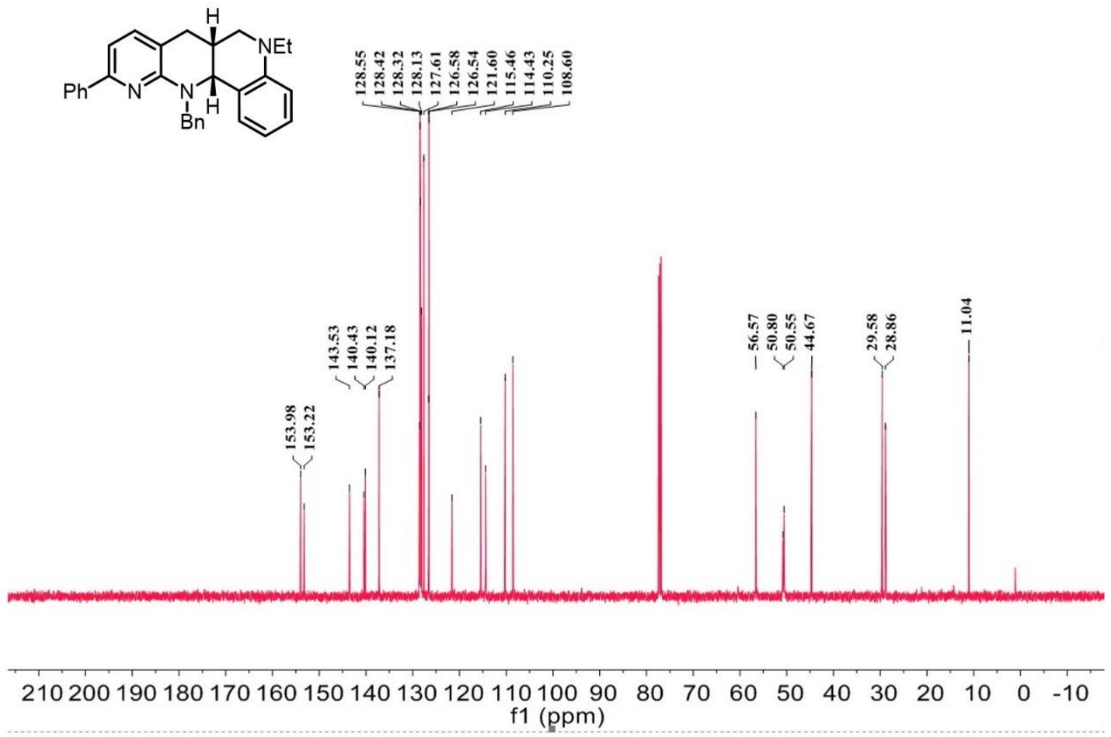

Supplementary Fig. 94 ^{13}C-NMR spectrum of P37

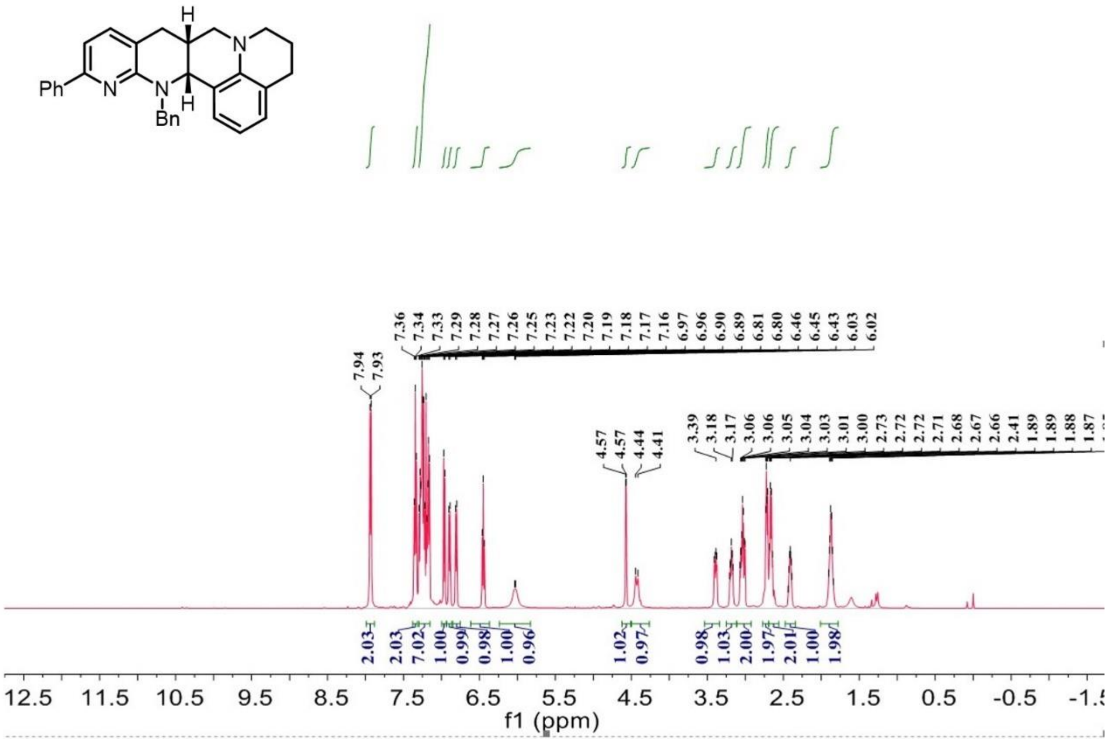

Supplementary Fig. 95 ^{1}H-NMR spectrum of P38

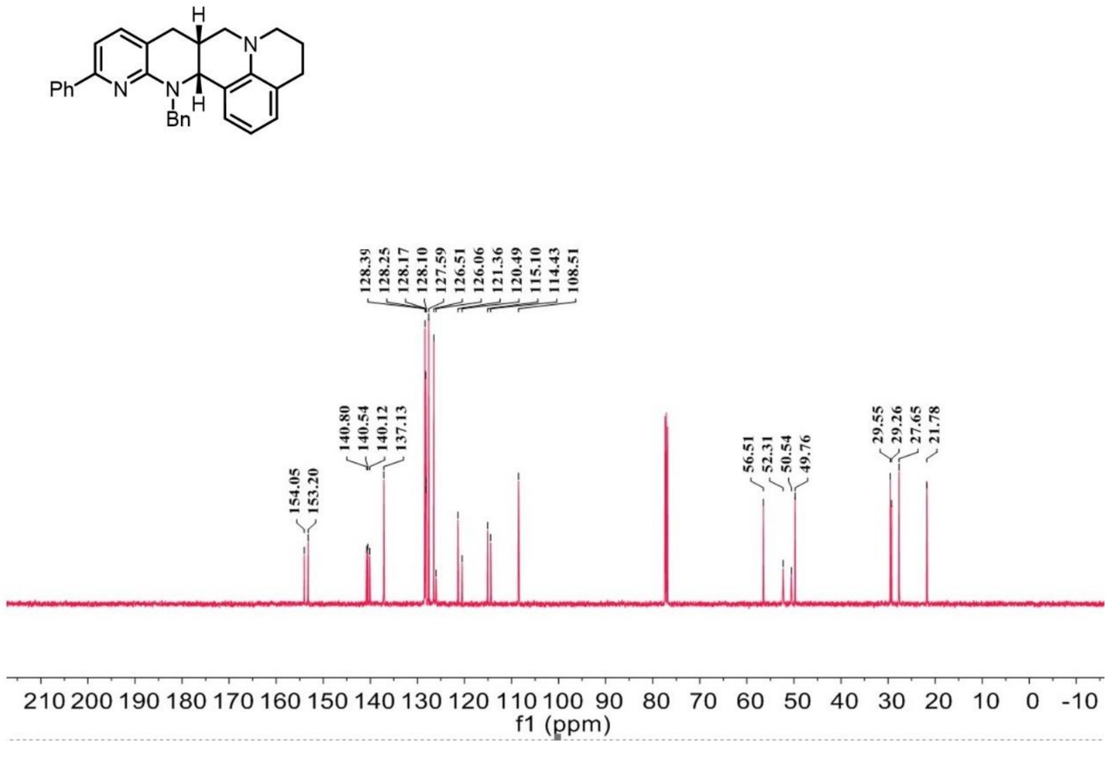

Supplementary Fig. 96 ^{13}C-NMR spectrum of P38

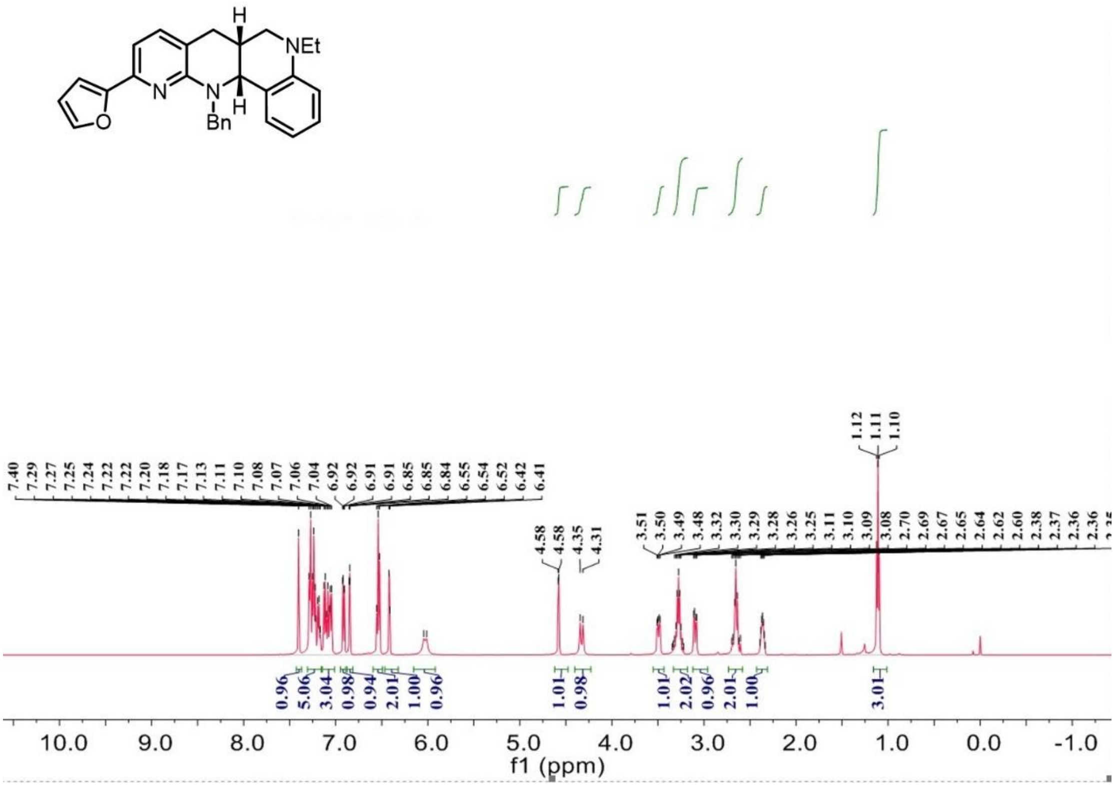

Supplementary Fig. 97 ^{1}H-NMR spectrum of P39

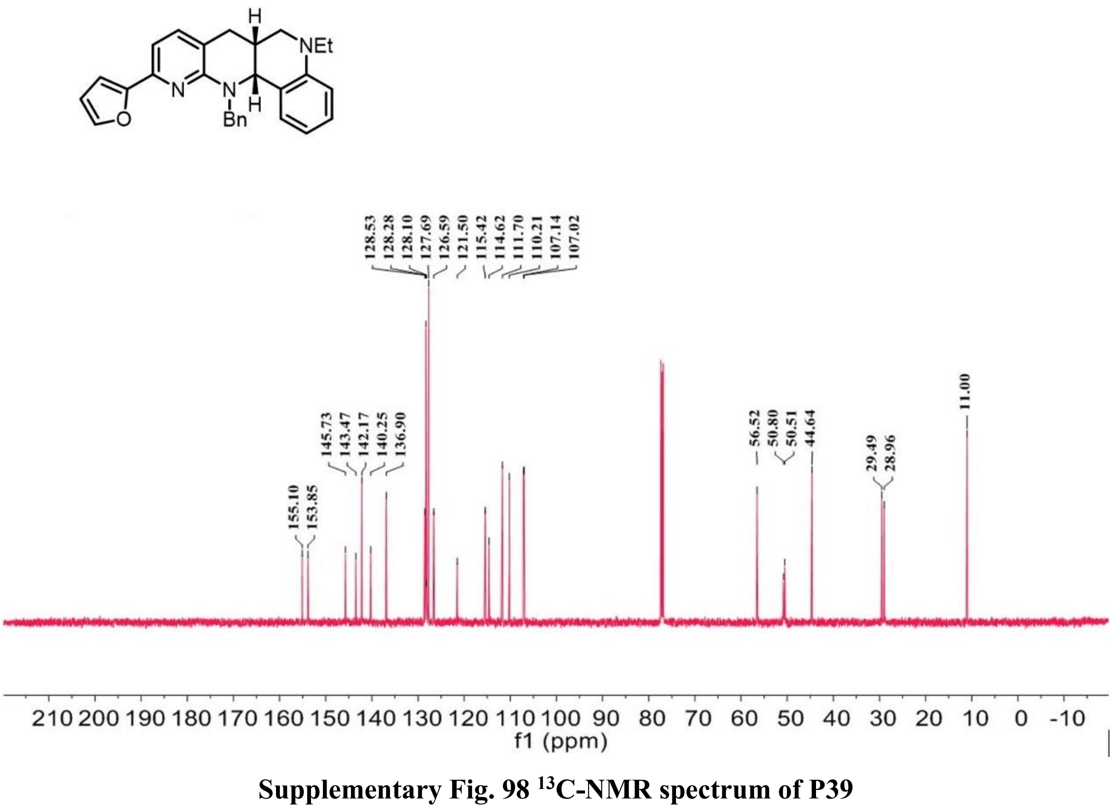

Supplementary Fig. 98 ^{13}C-NMR spectrum of P39

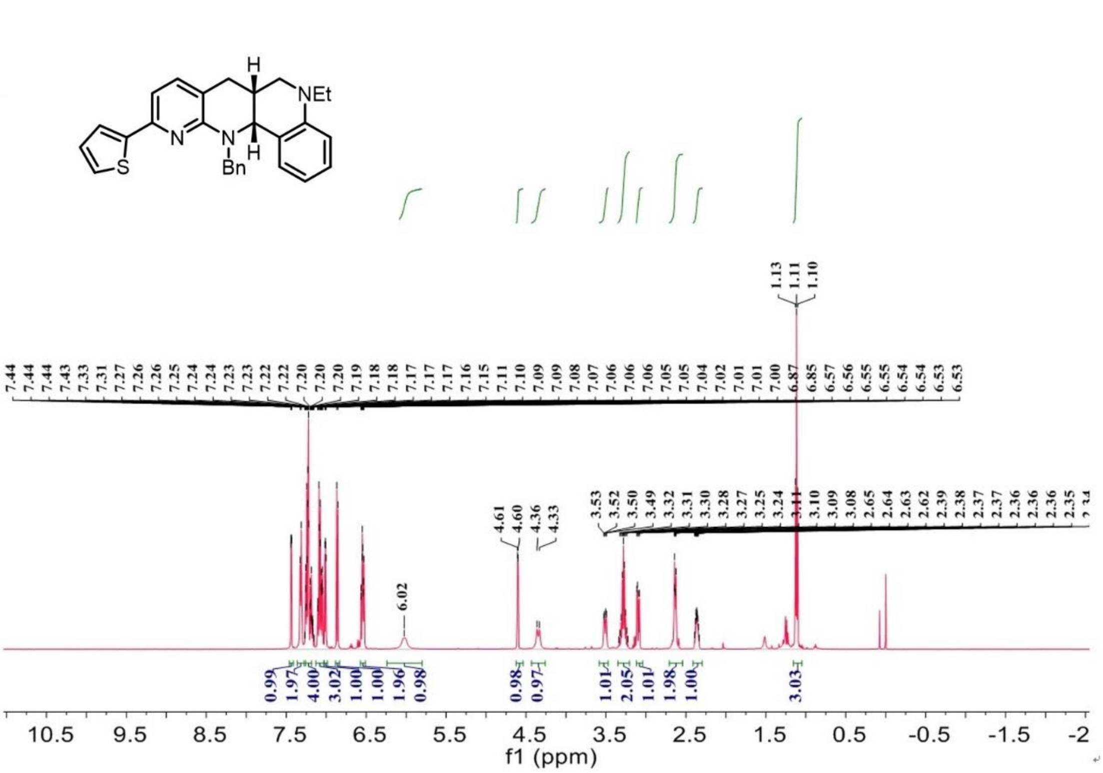

Supplementary Fig. 99 ^{1}H-NMR spectrum of P40

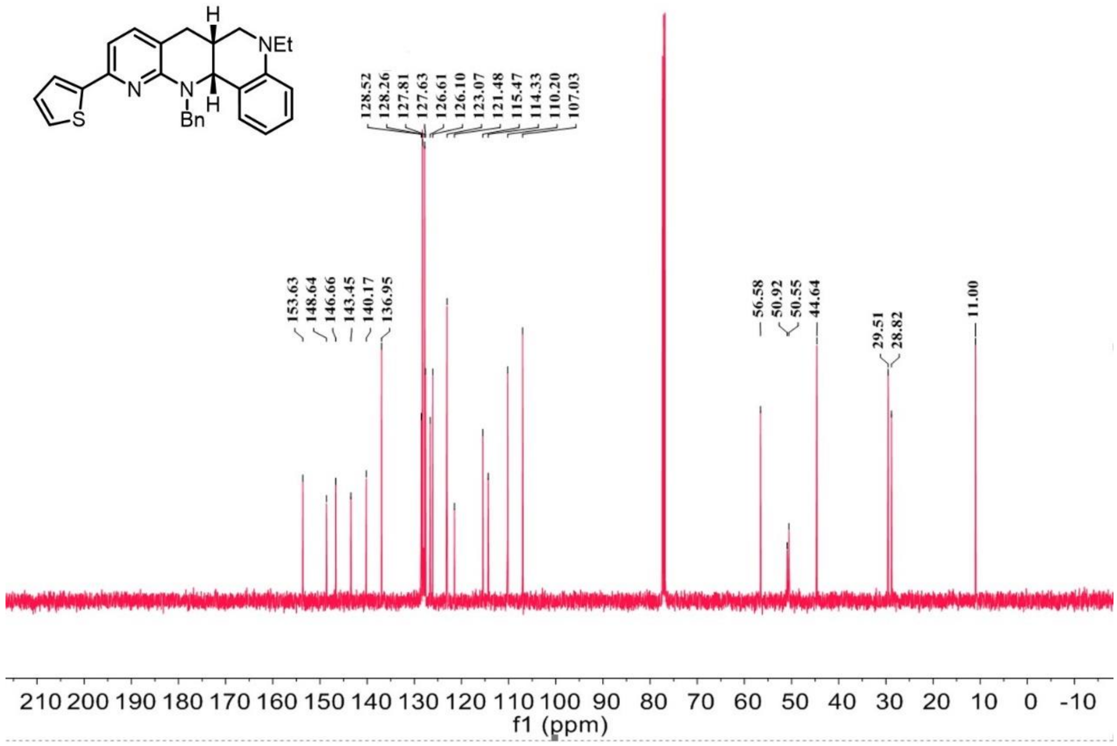

Supplementary Fig. 100 ^{13}C-NMR spectrum of P40

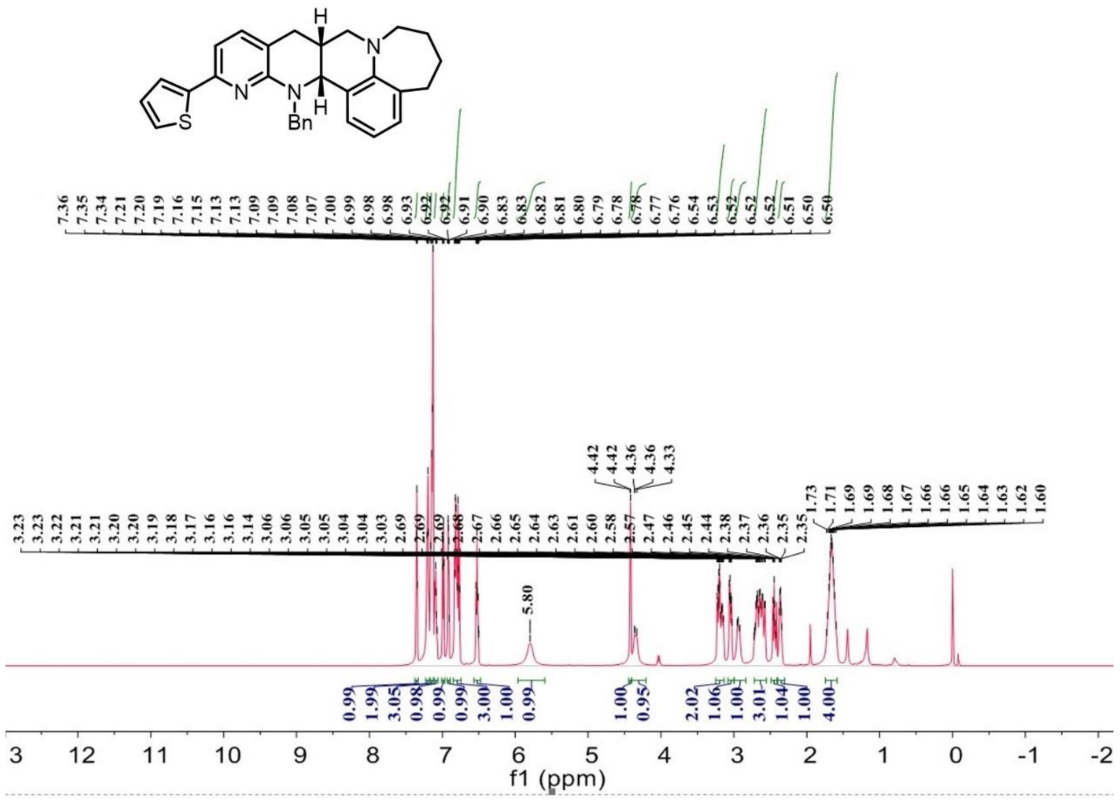

Supplementary Fig. 101 ^{1}H-NMR spectrum of P41

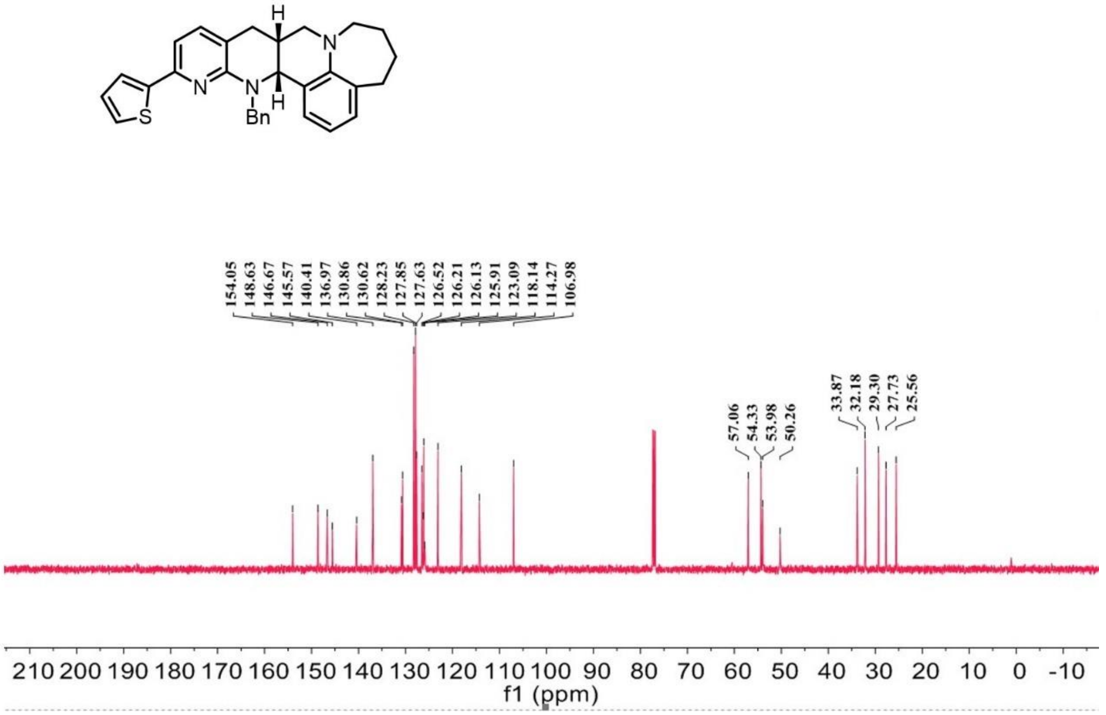

Supplementary Fig. 102 ^{13}C-NMR spectrum of P41

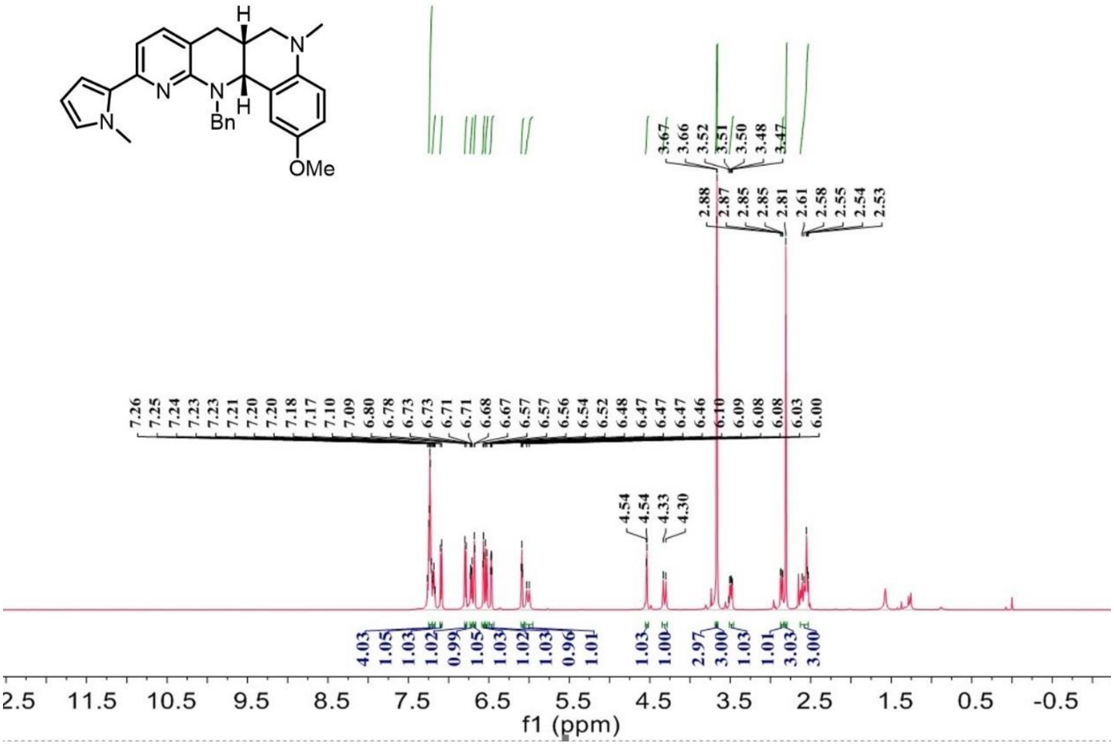

Supplementary Fig. 103 ^{1}H-NMR spectrum of P42

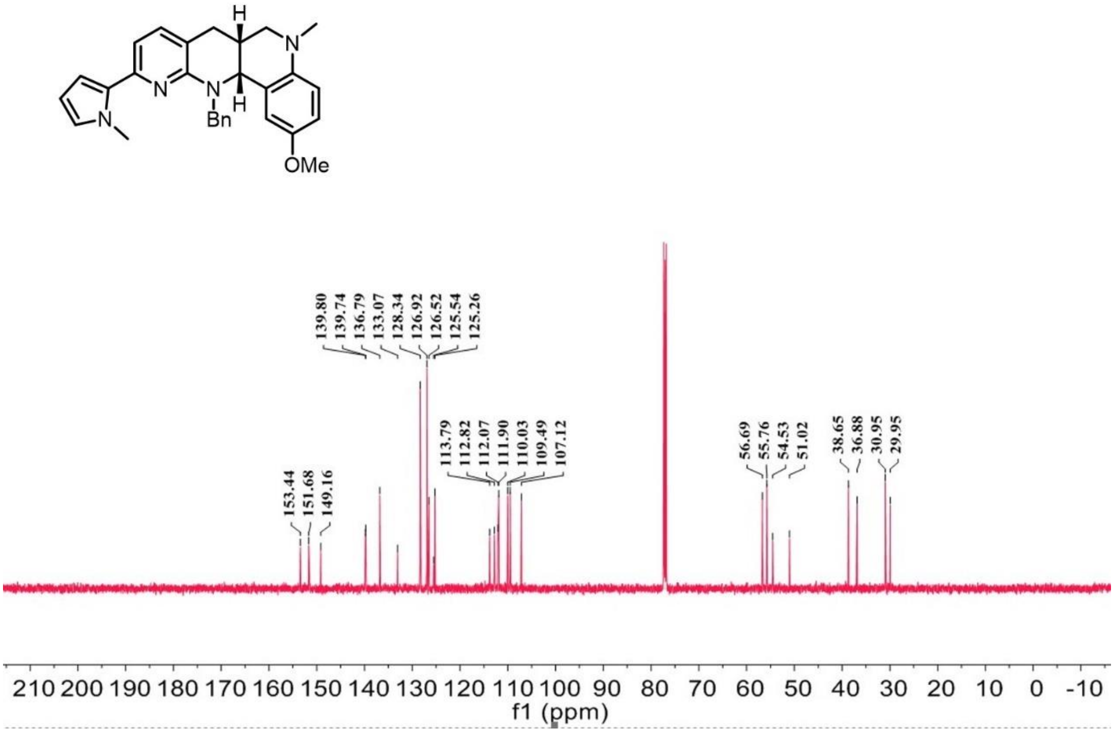

Supplementary Fig. 104 ^{13}C-NMR spectrum of P42

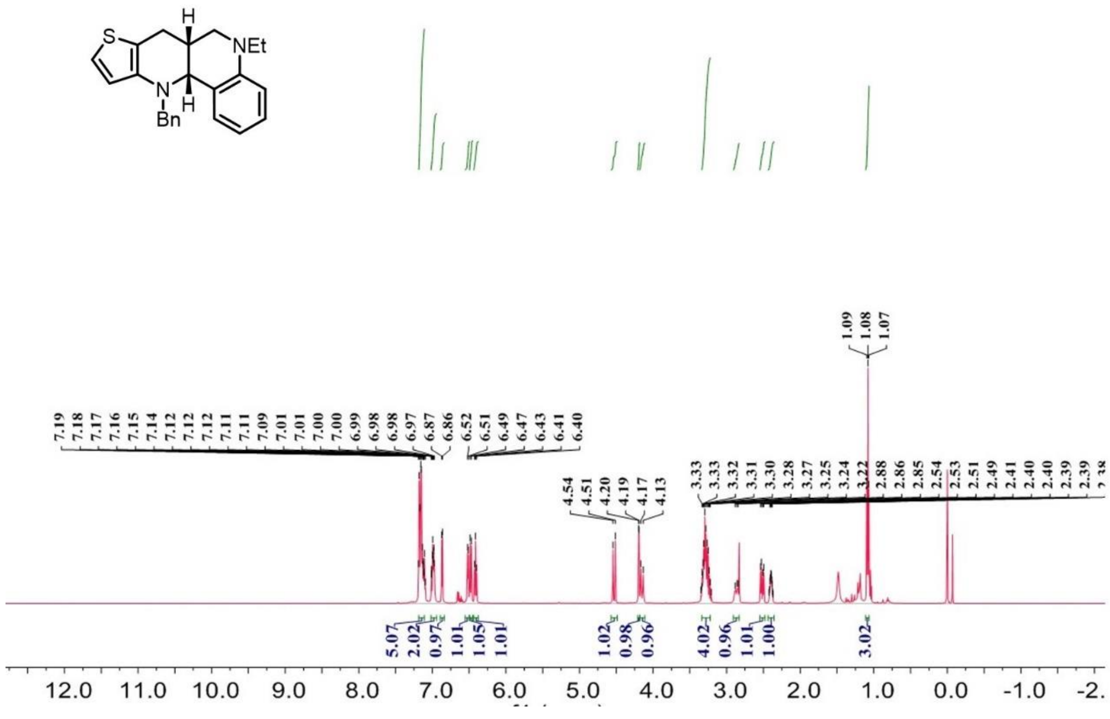

Supplementary Fig. 105 ^{1}H-NMR spectrum of P43

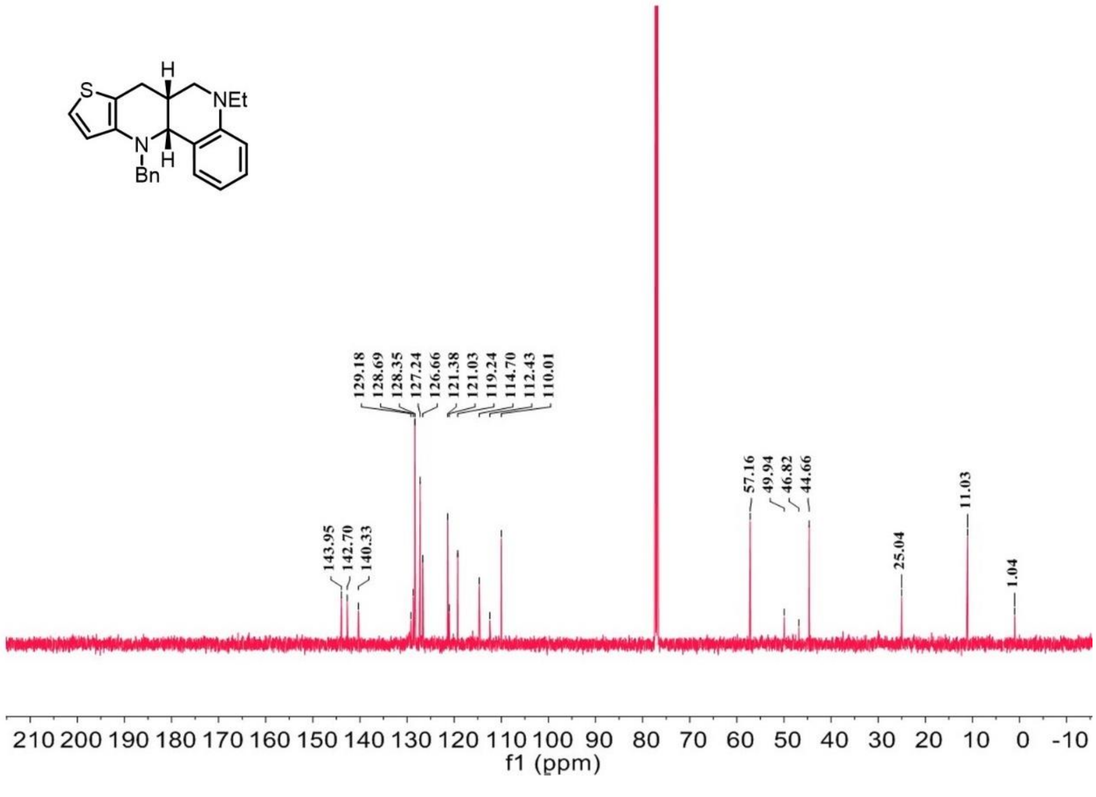

Supplementary Fig. 106 ^{13}C-NMR spectrum of P43

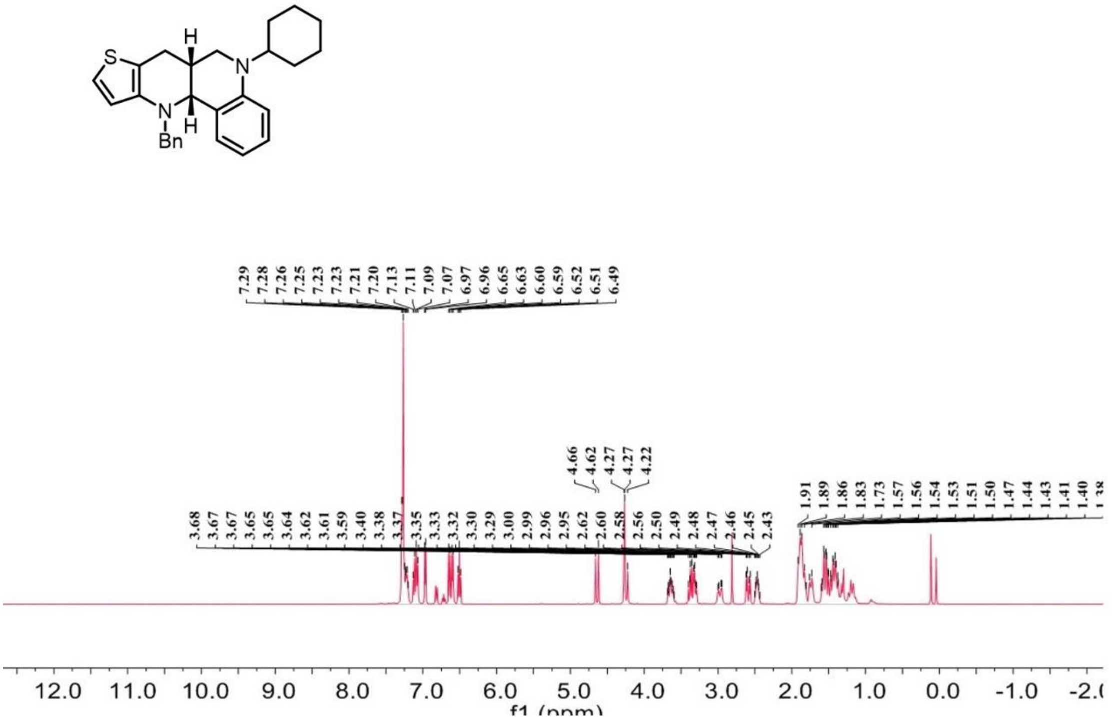

Supplementary Fig. 107 ^{1}H-NMR spectrum of P44

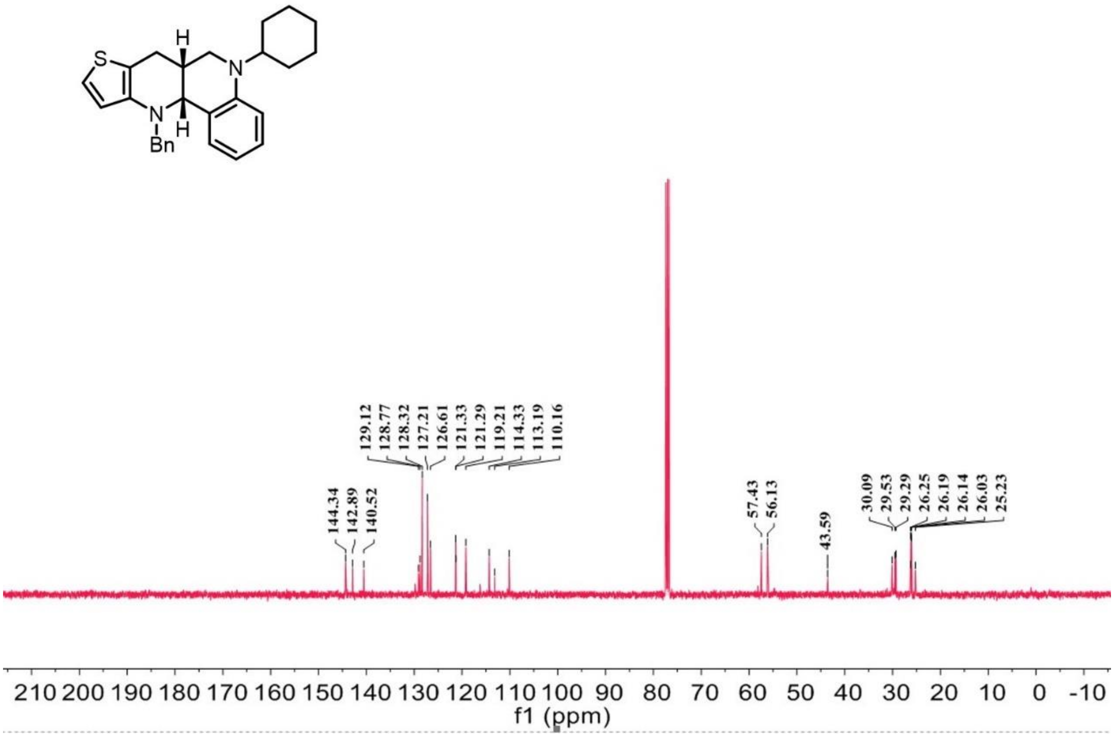

Supplementary Fig. 108 ^{13}C-NMR spectrum of P44

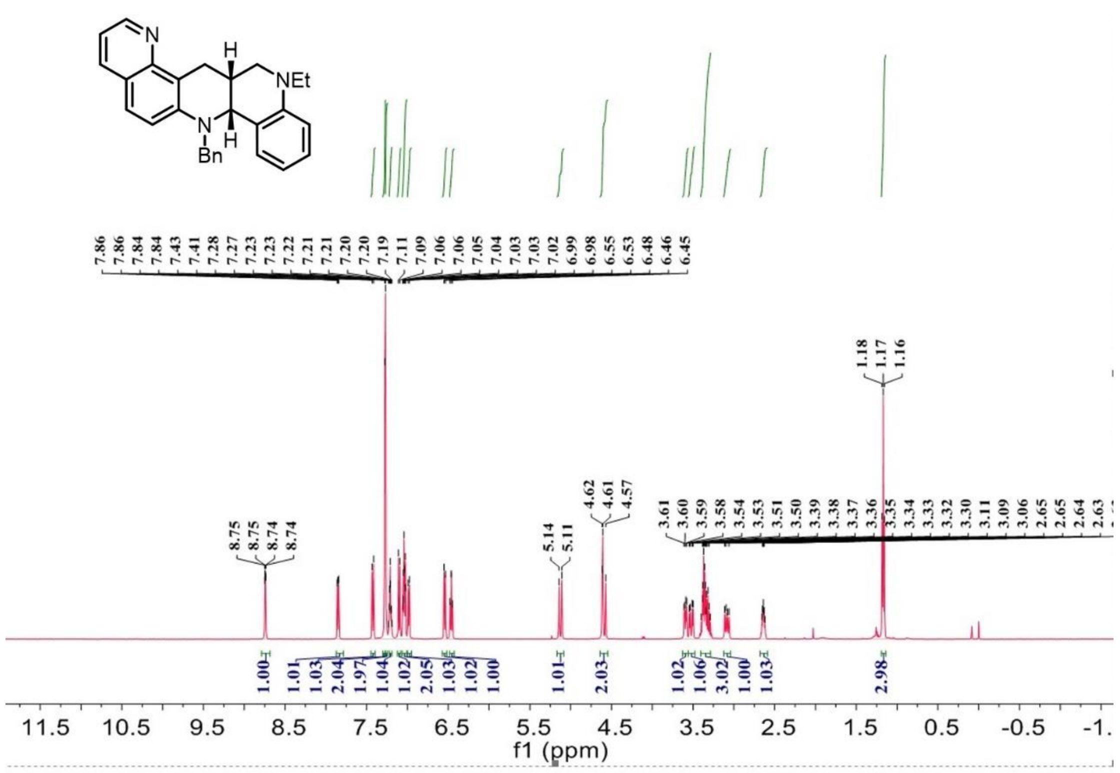

Supplementary Fig. 109 ^{1}H-NMR spectrum of P45

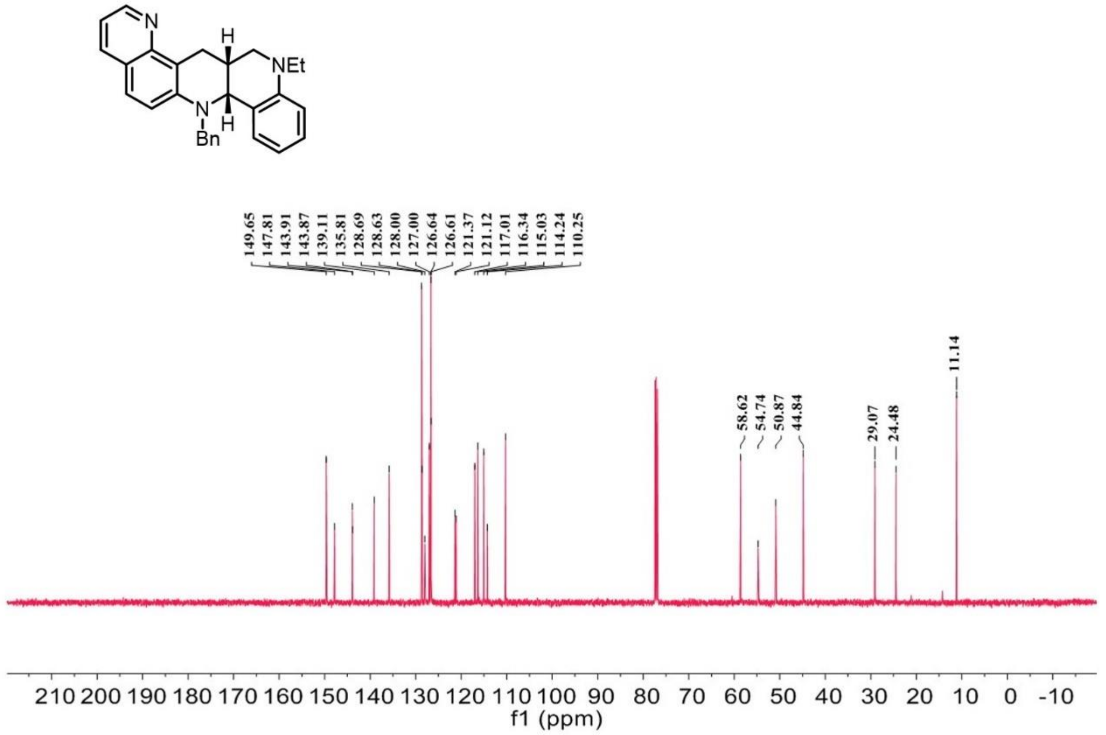

Supplementary Fig. 110 ^{13}C-NMR spectrum of P45

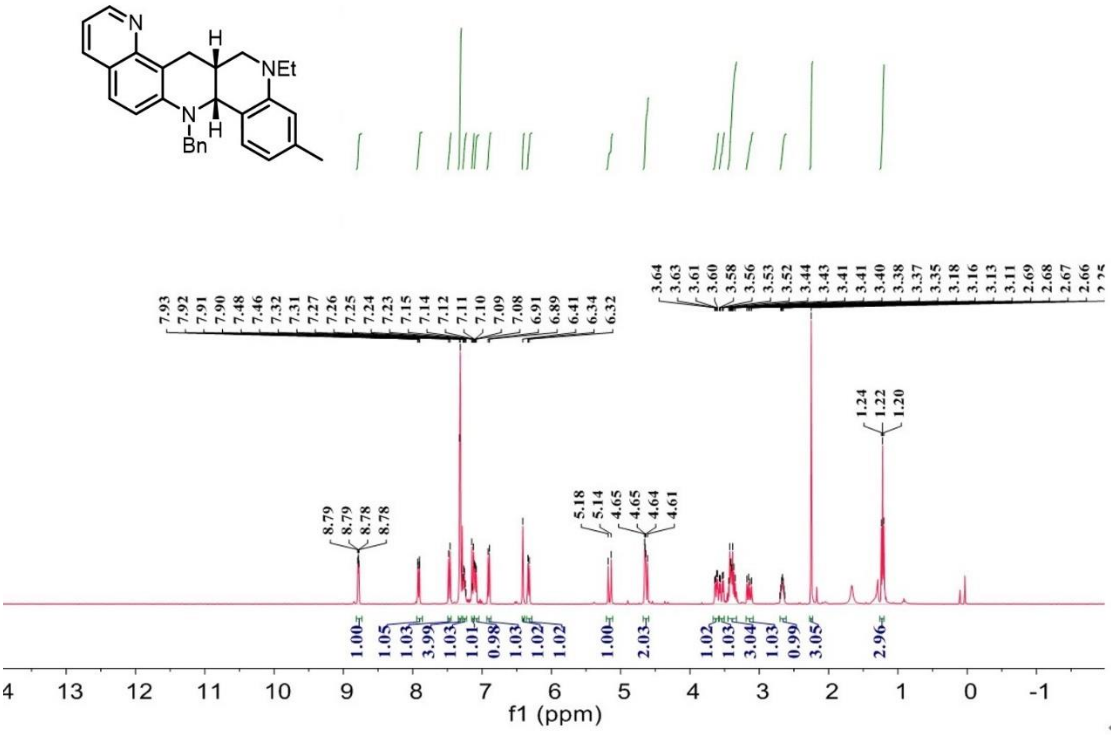

Supplementary Fig. 111 ^{1}H-NMR spectrum of P46

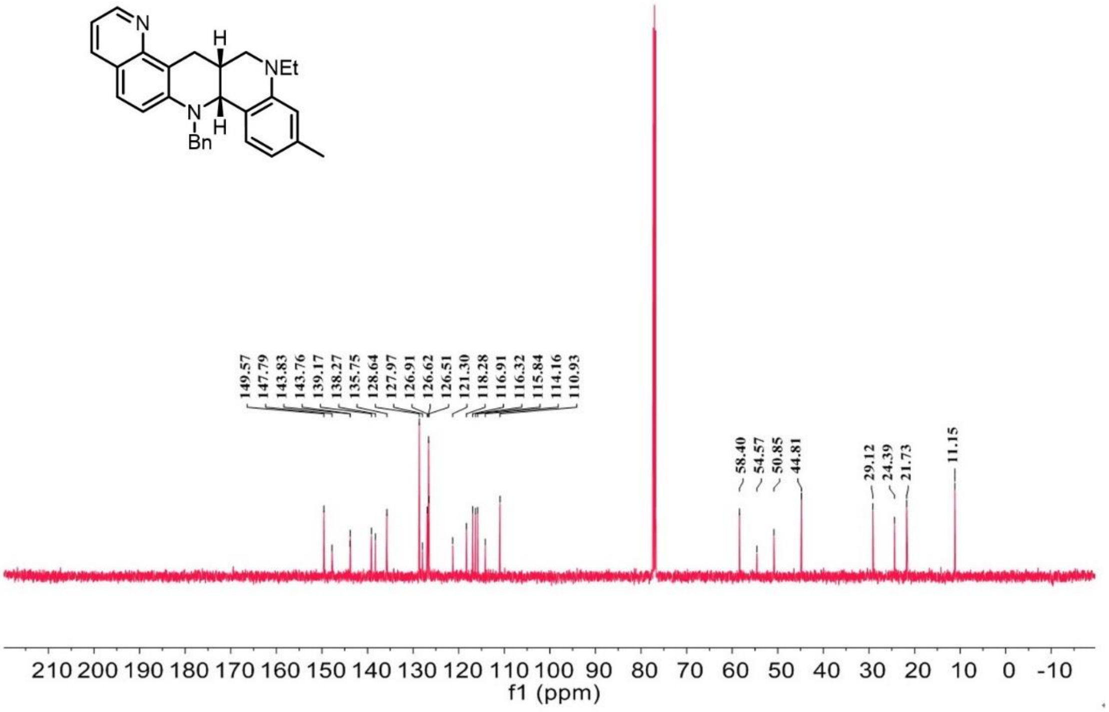

Supplementary Fig. 112 ^{13}C-NMR spectrum of P46

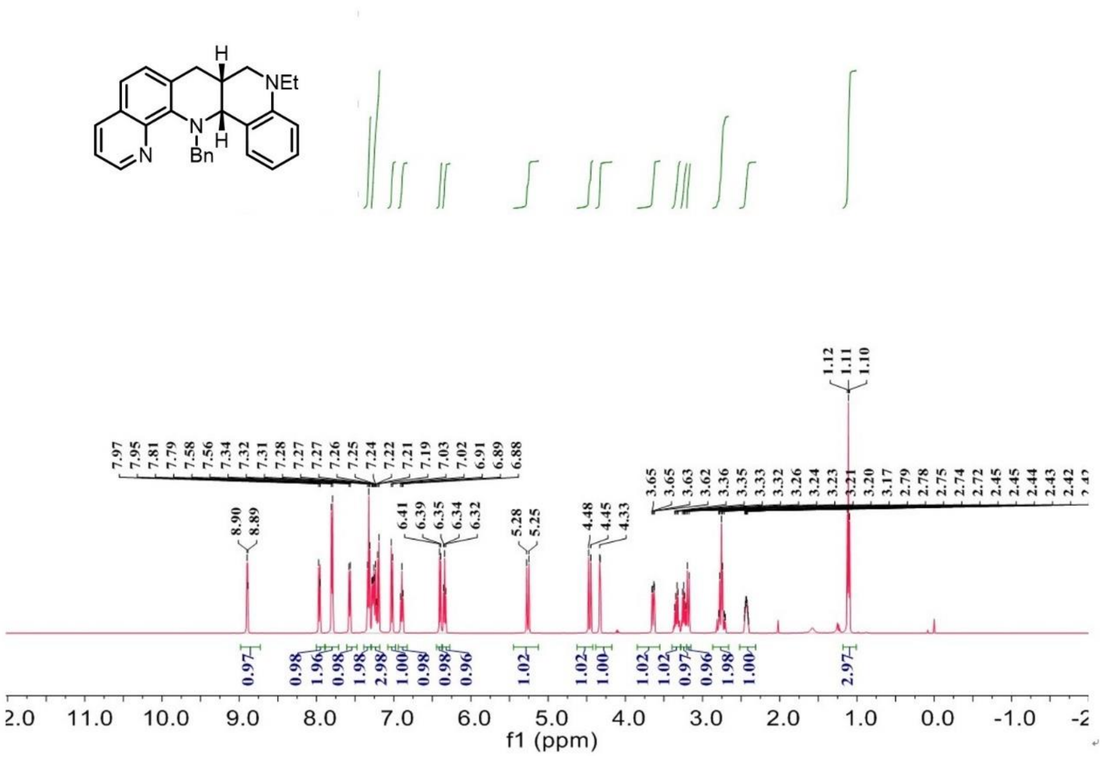

Supplementary Fig. 113 ^{1}H-NMR spectrum of P47

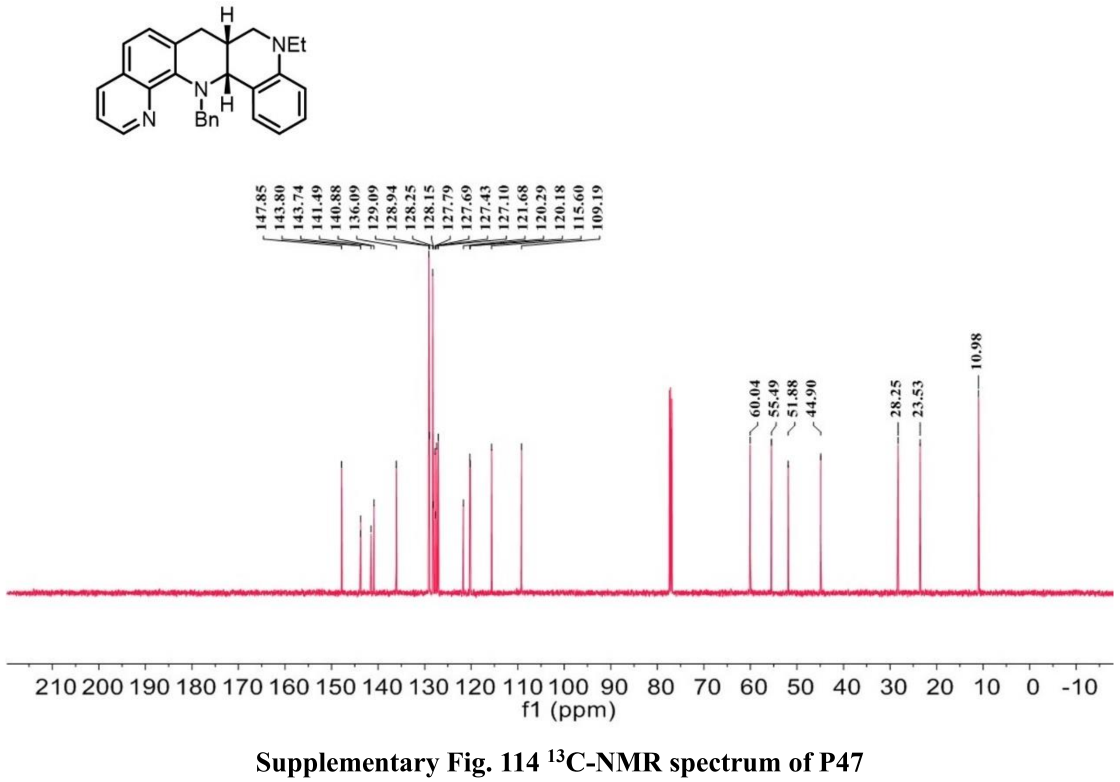

Supplementary Fig. 114 ^{13}C-NMR spectrum of P47

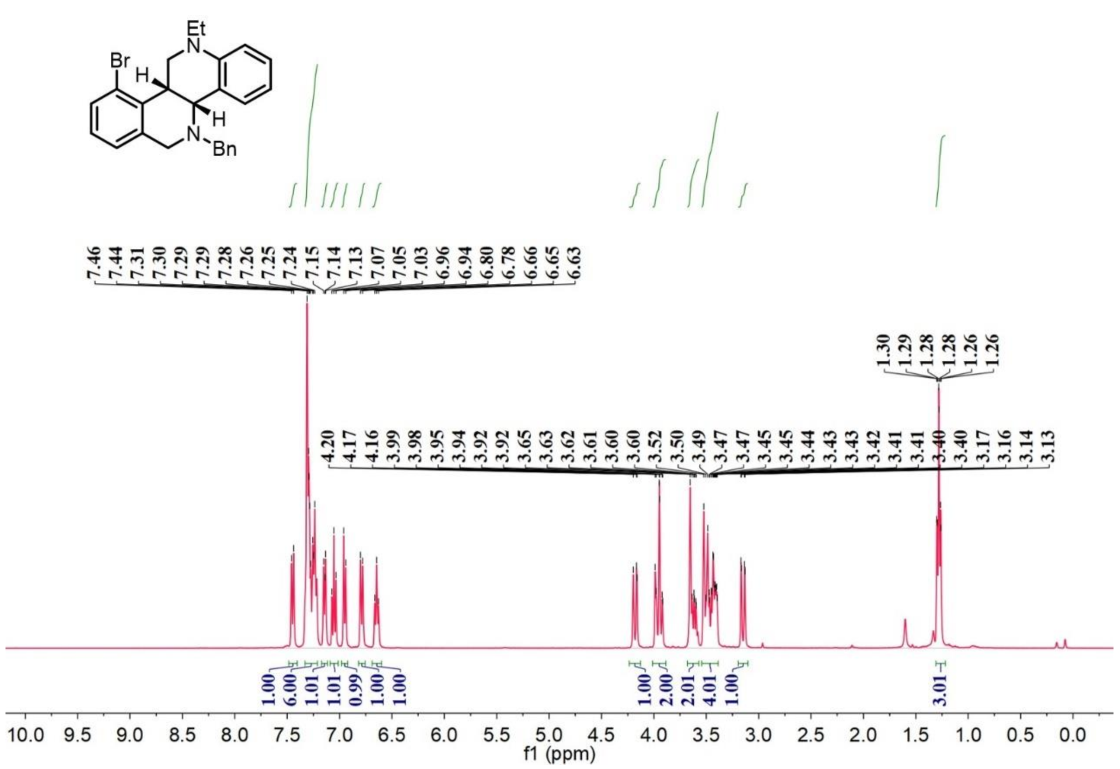

Supplementary Fig. 115 ^{1}H-NMR spectrum of P48

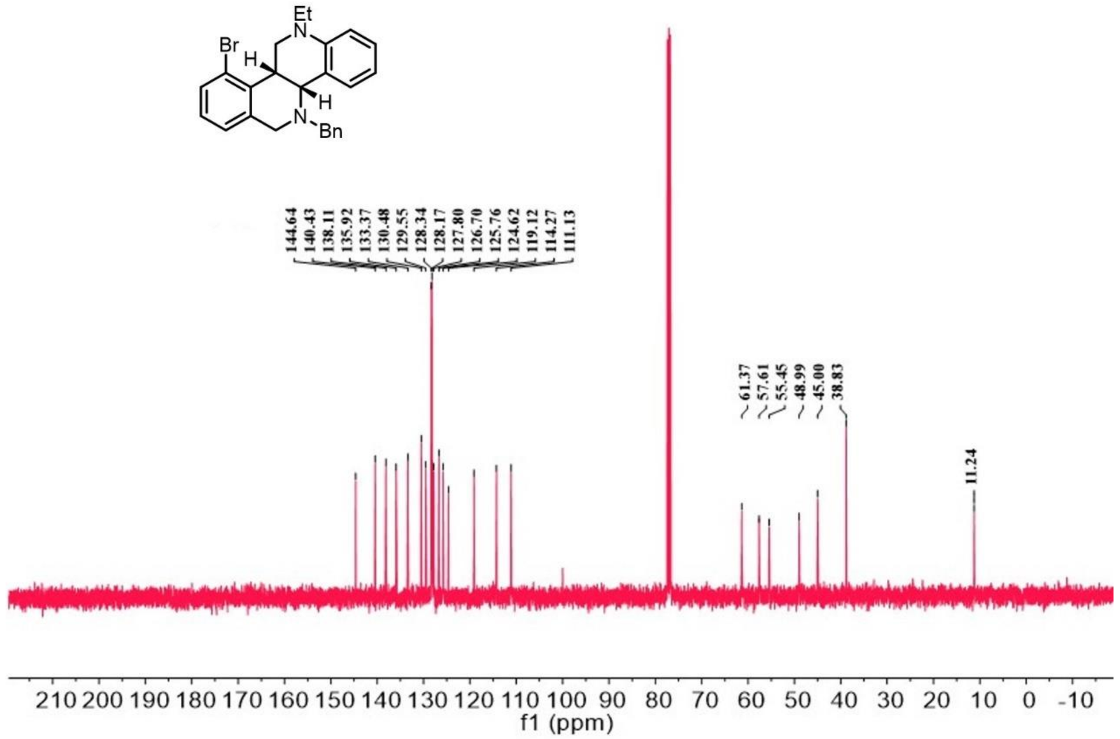

Supplementary Fig. 116 ^{13}C-NMR spectrum of P48

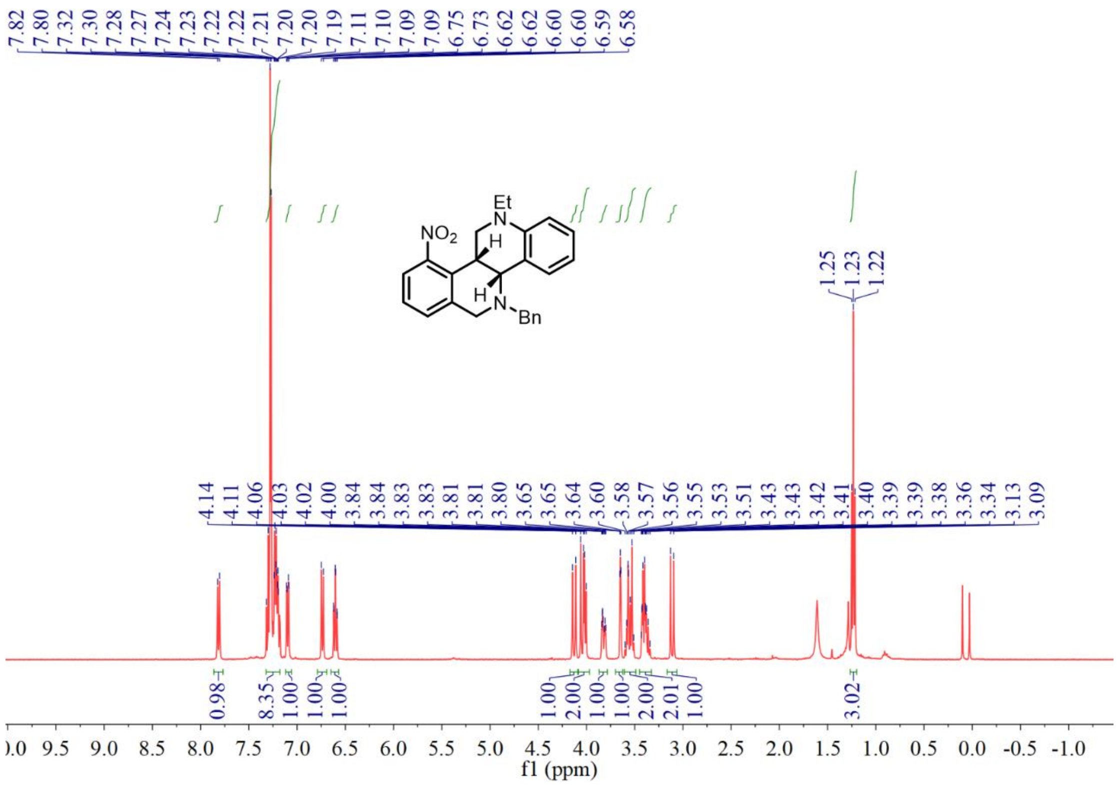

Supplementary Fig. 117 ^{1}H-NMR spectrum of P49

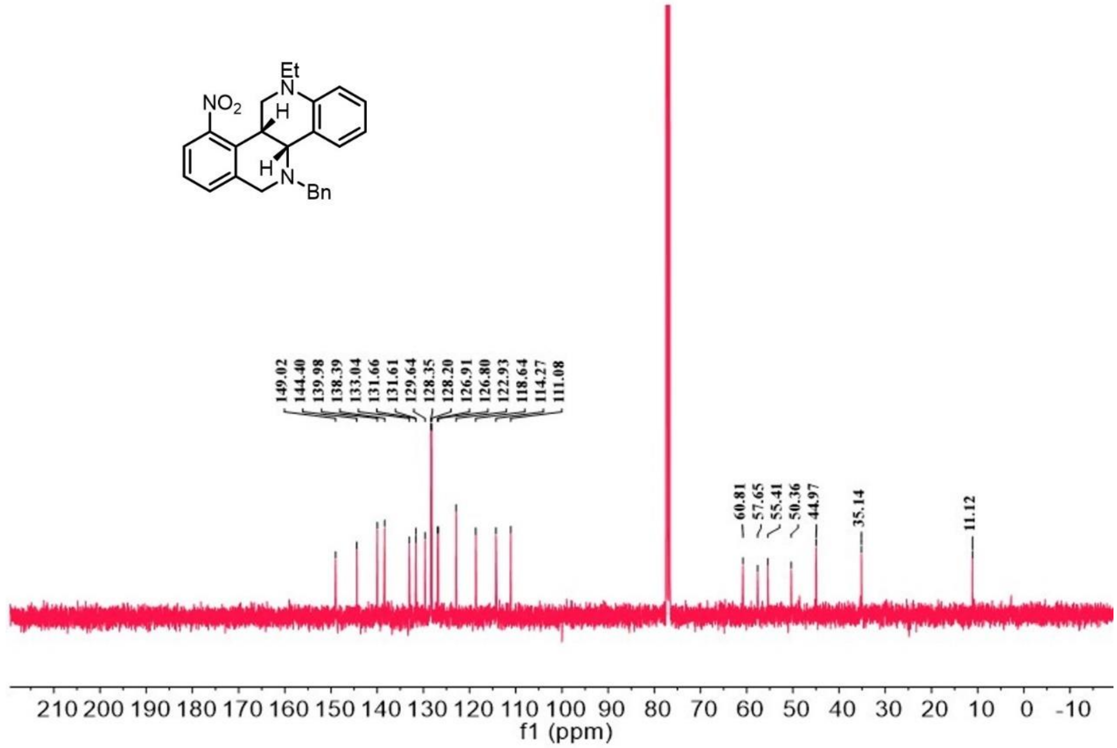

Supplementary Fig. 118 ^{13}C-NMR spectrum of P49

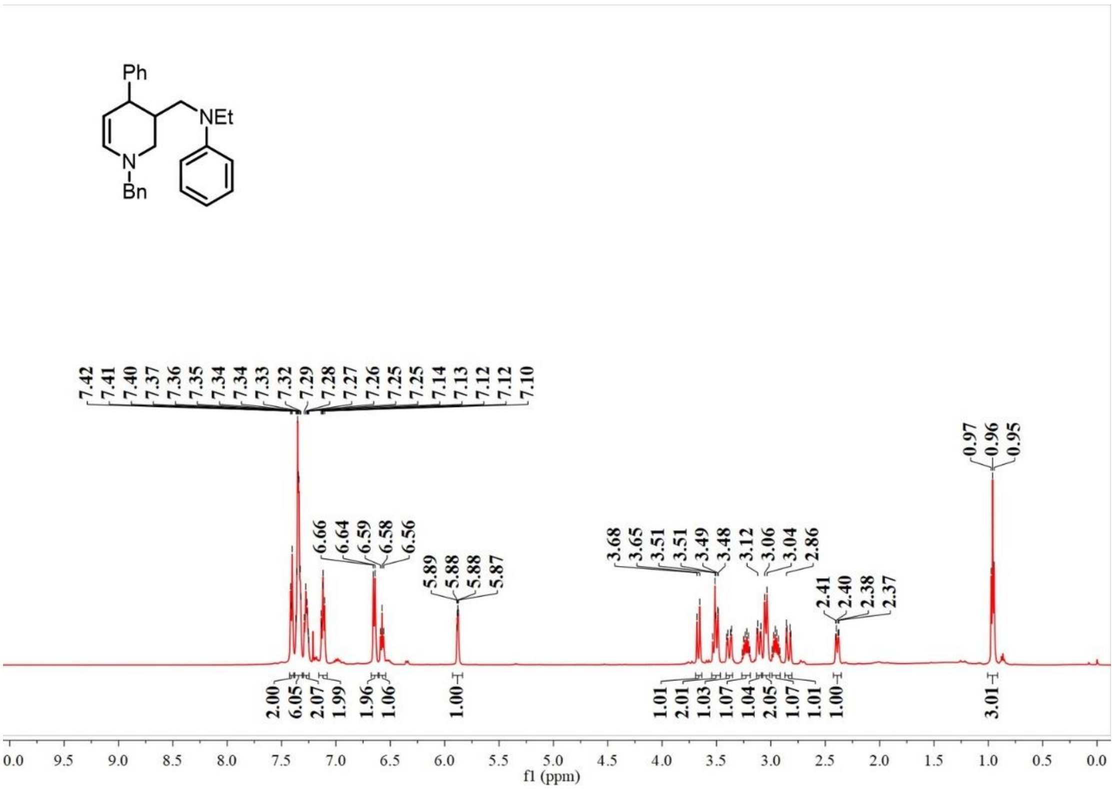

Supplementary Fig. 119 ^{1}H-NMR spectrum of P50

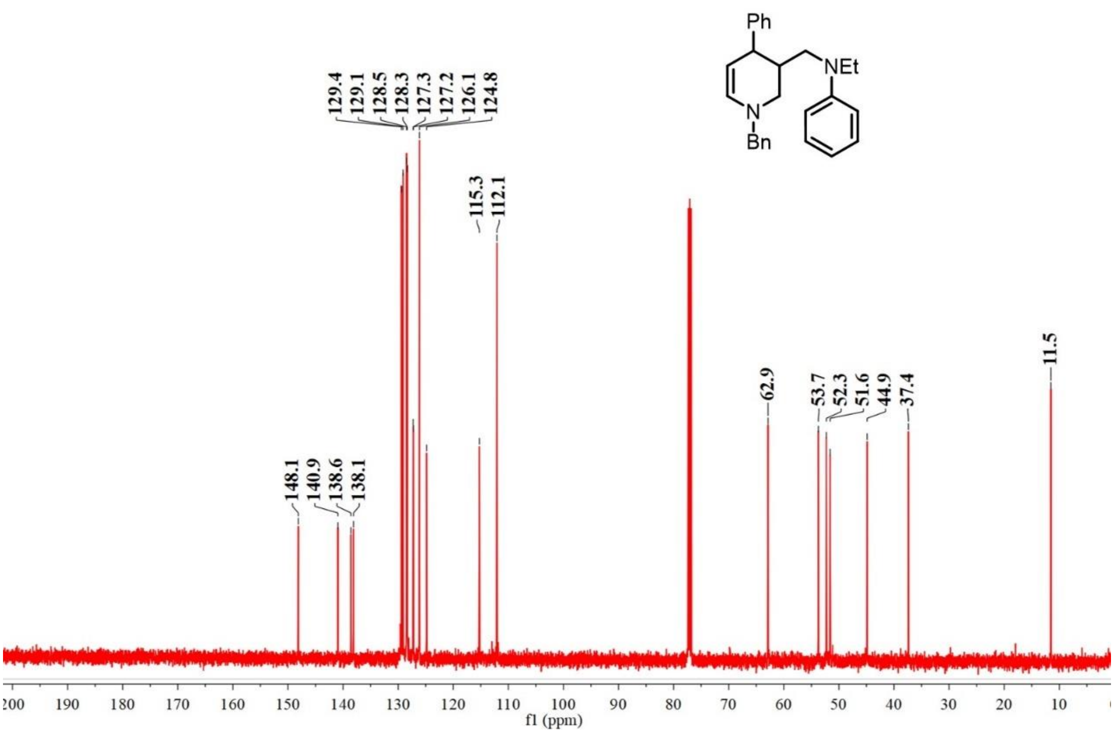

Supplementary Fig. 120 ^{13}C-NMR spectrum of P50

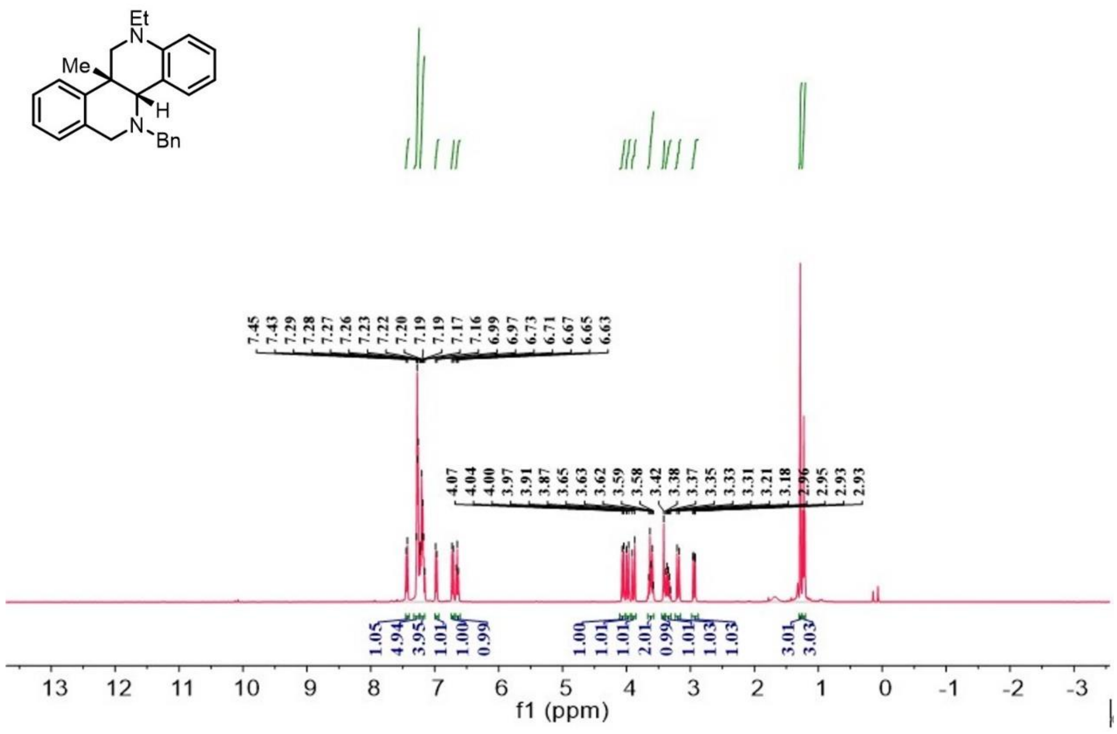

Supplementary Fig. 121 ^{1}H-NMR spectrum of P51

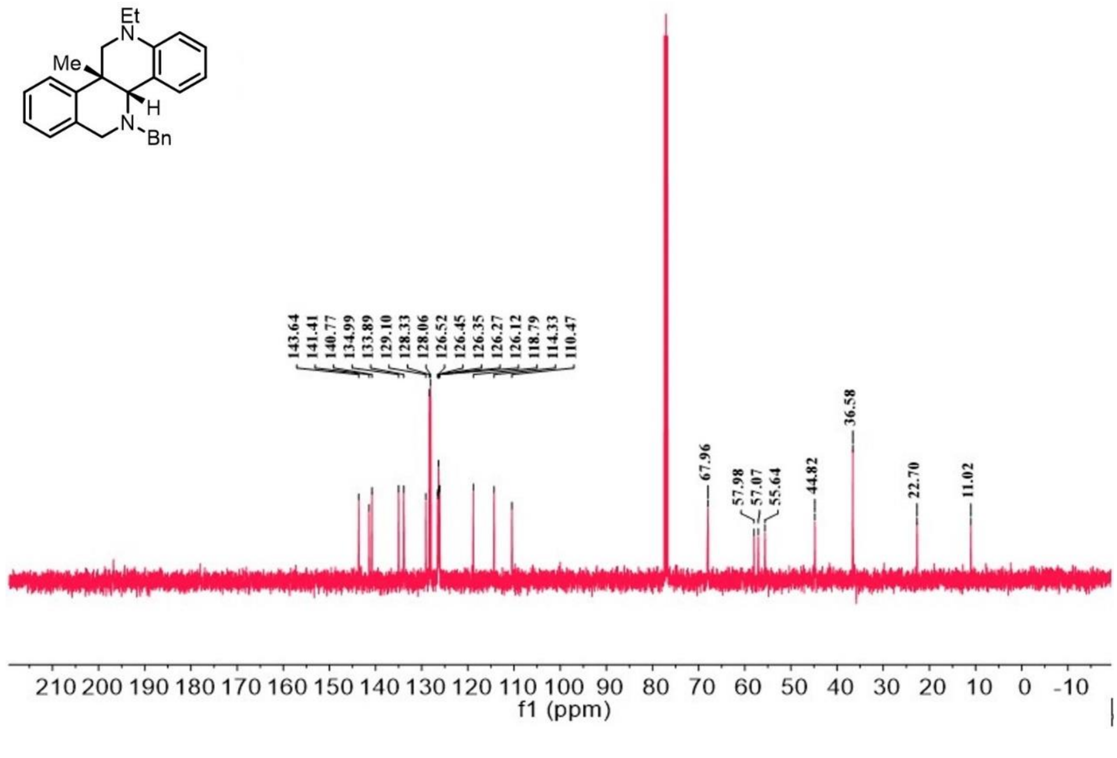

Supplementary Fig. 122 ^{13}C-NMR spectrum of P51

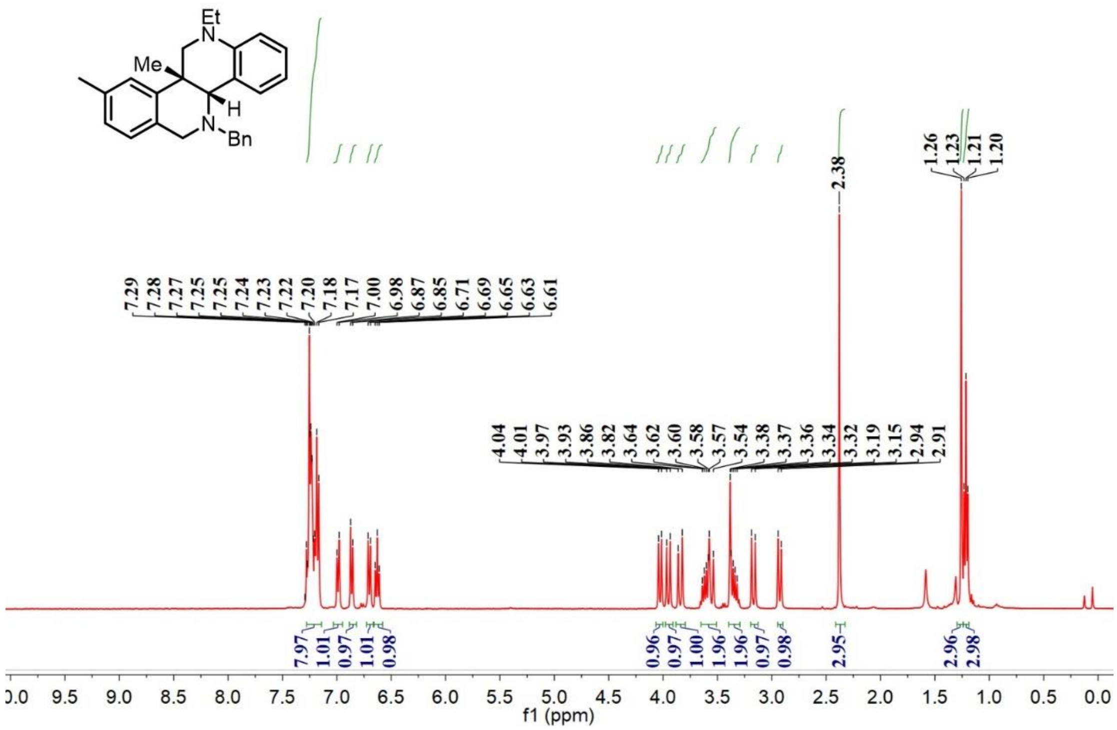

Supplementary Fig. 123 ^{1}H-NMR spectrum of P52

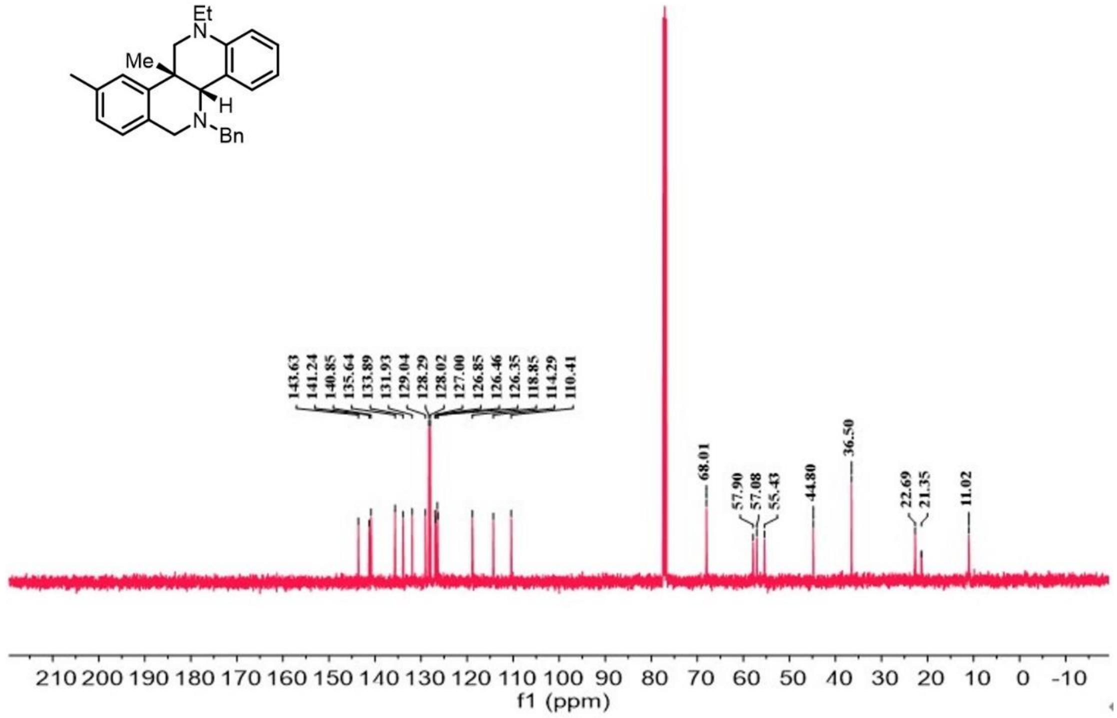

Supplementary Fig. 124 ^{13}C-NMR spectrum of P52

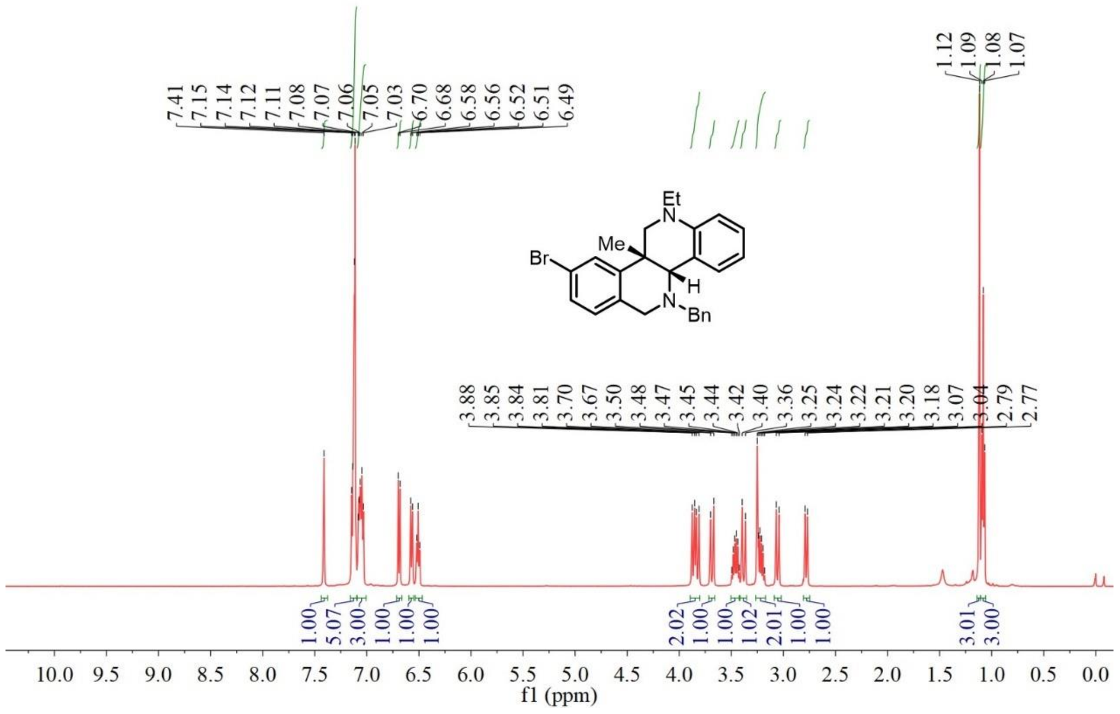

Supplementary Fig. 125 ^{1}H-NMR spectrum of P53

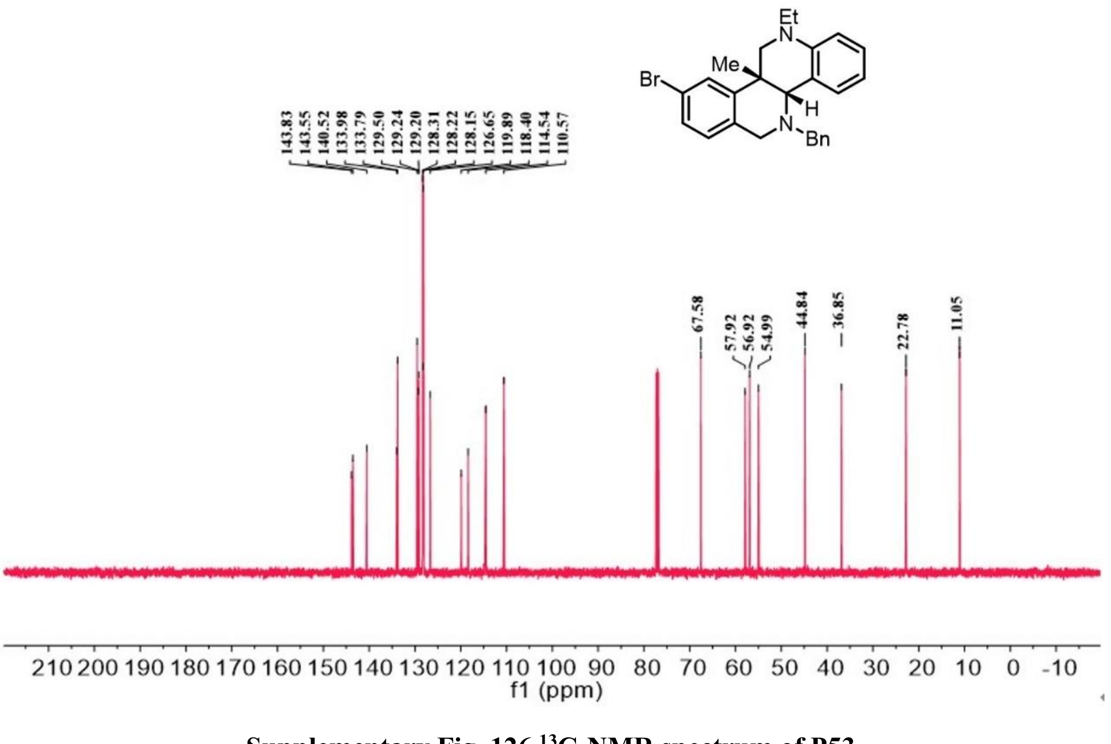

Supplementary Fig. 126 ^{13}C-NMR spectrum of P53

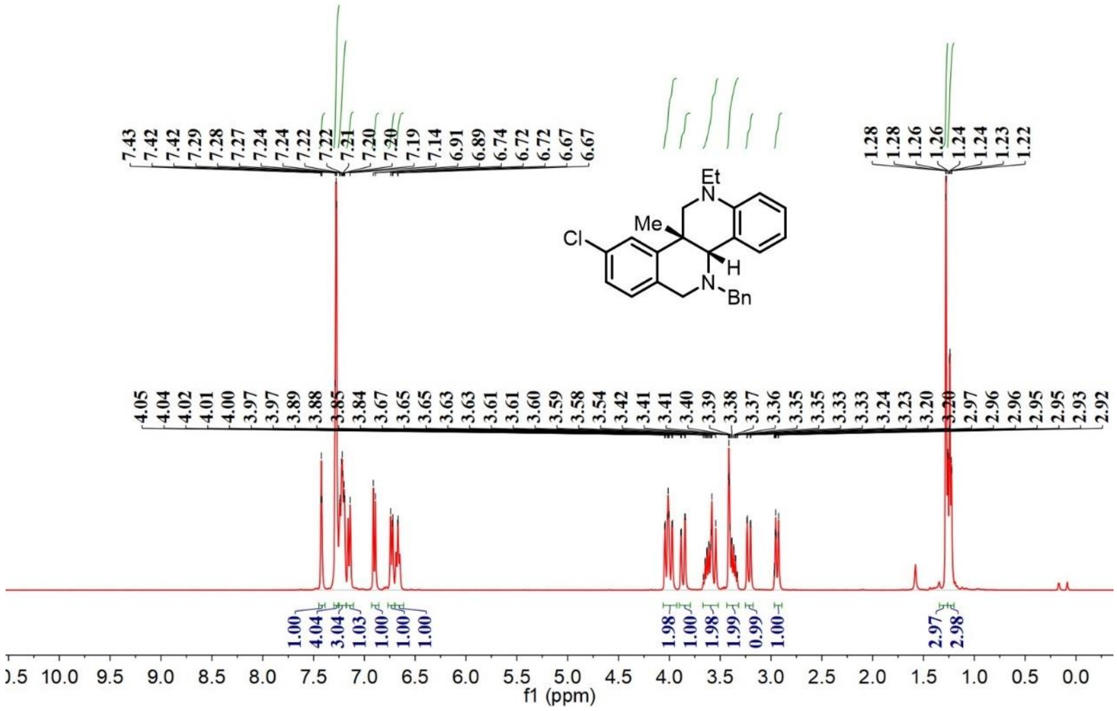

Supplementary Fig. 127 ^{1}H-NMR spectrum of P54

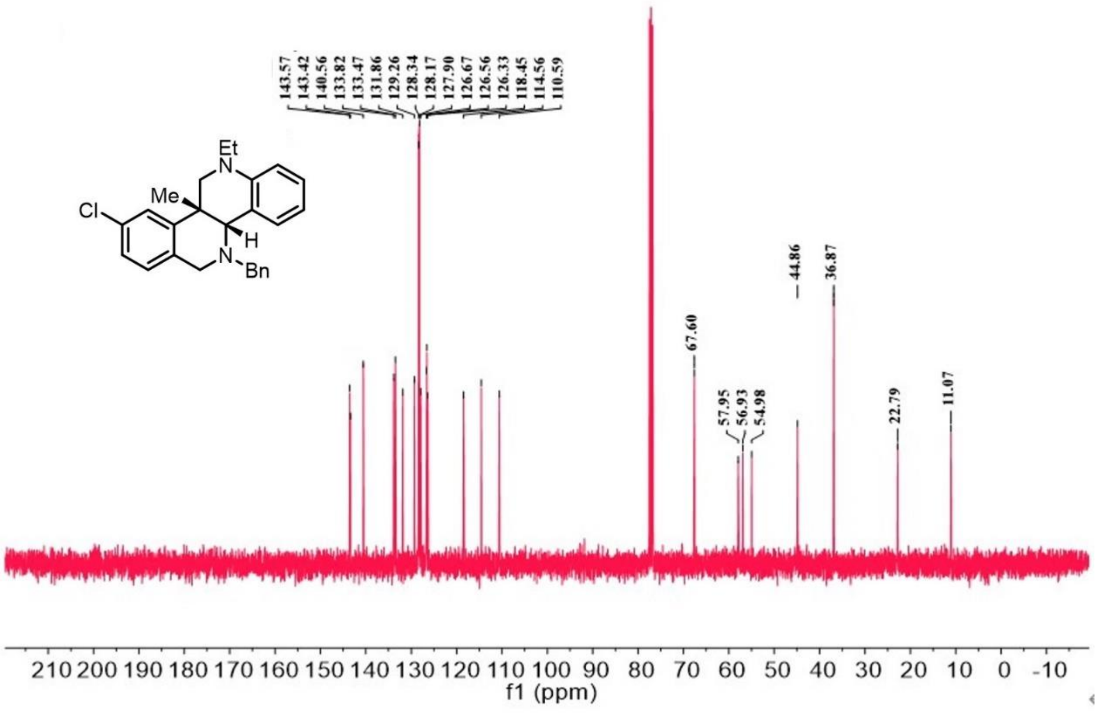

Supplementary Fig. 128 ^{13}C-NMR spectrum of P54

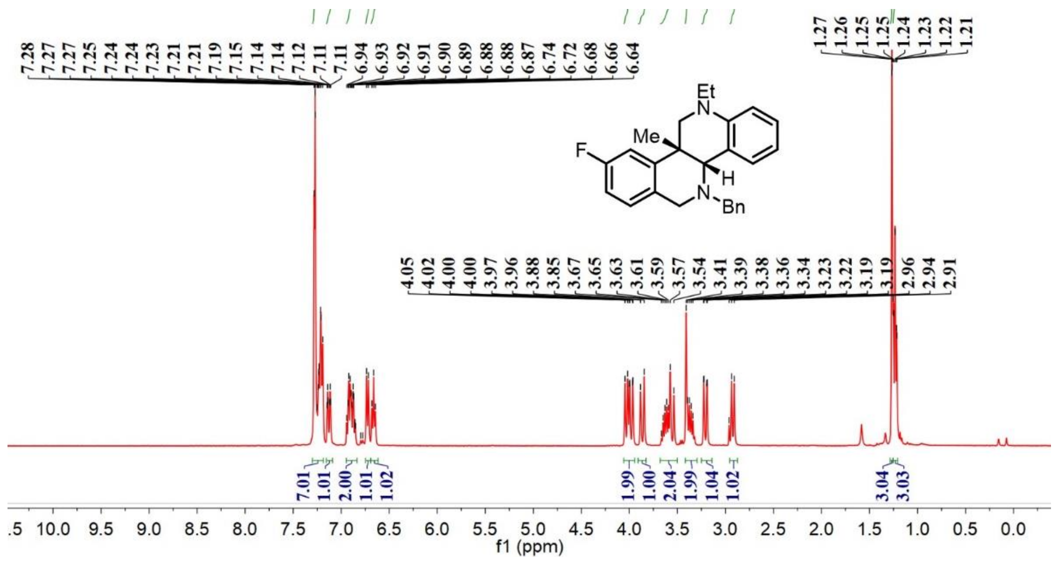

Supplementary Fig. 129 ^{1}H-NMR spectrum of P55

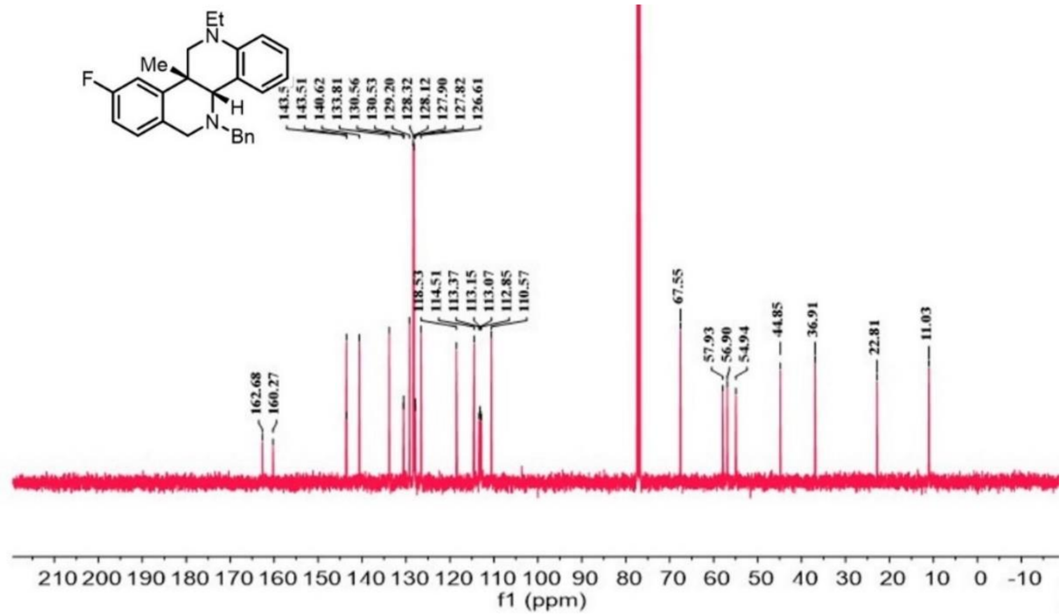

Supplementary Fig. 130 ^{13}C-NMR spectrum of P55

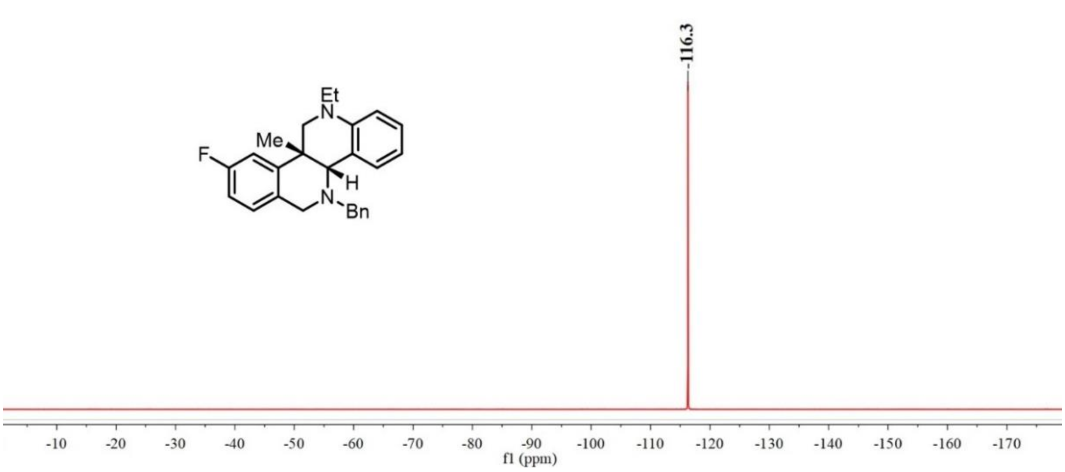

Supplementary Fig. 131 ^{19}F -NMR spectrum of P55

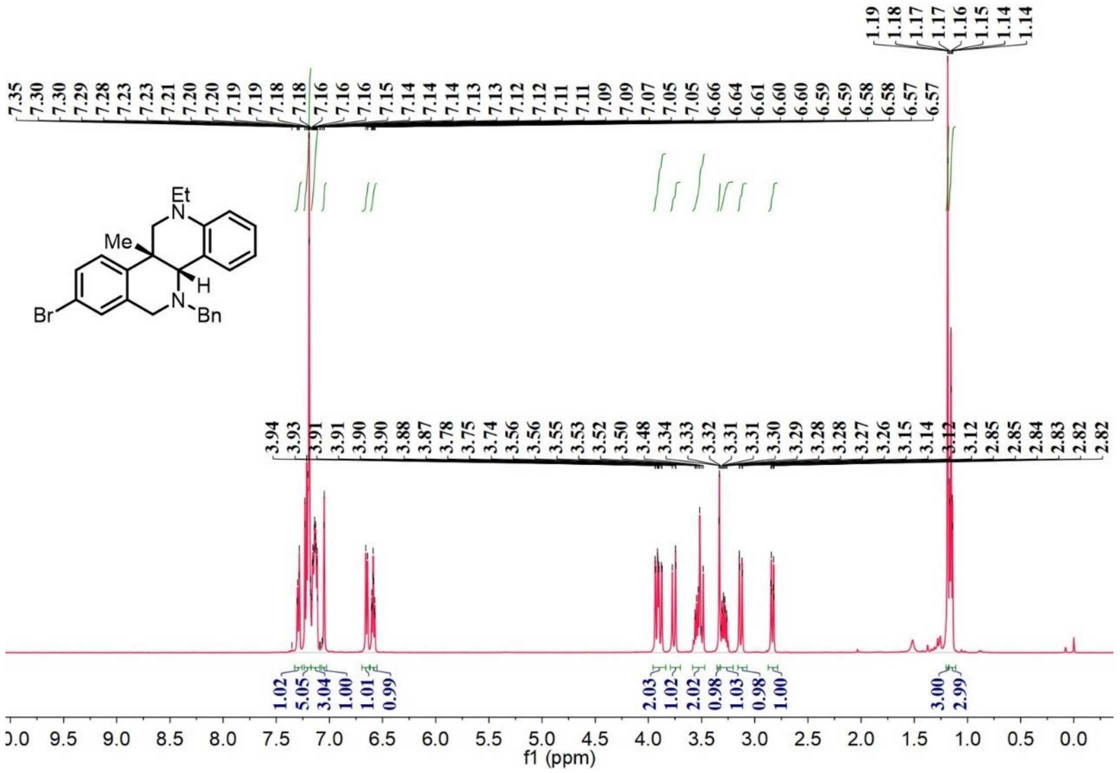

Supplementary Fig. 132 ^{1}H-NMR spectrum of P56

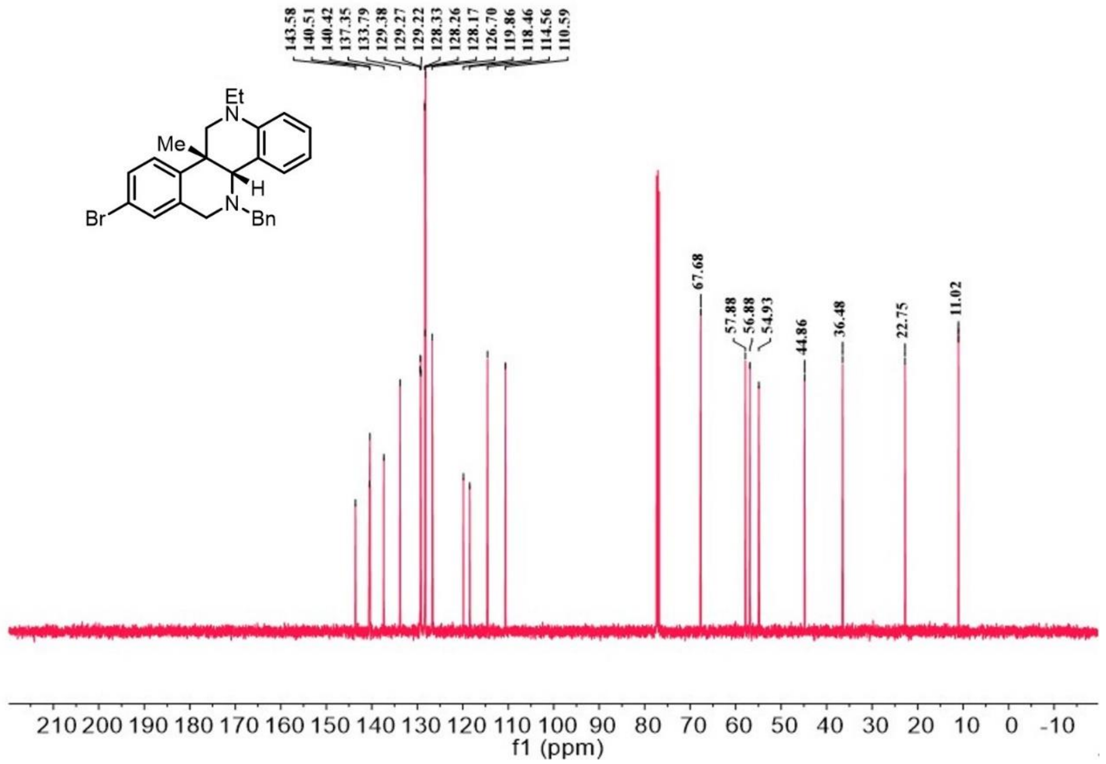

Supplementary Fig. 133 ^{13}C-NMR spectrum of P56

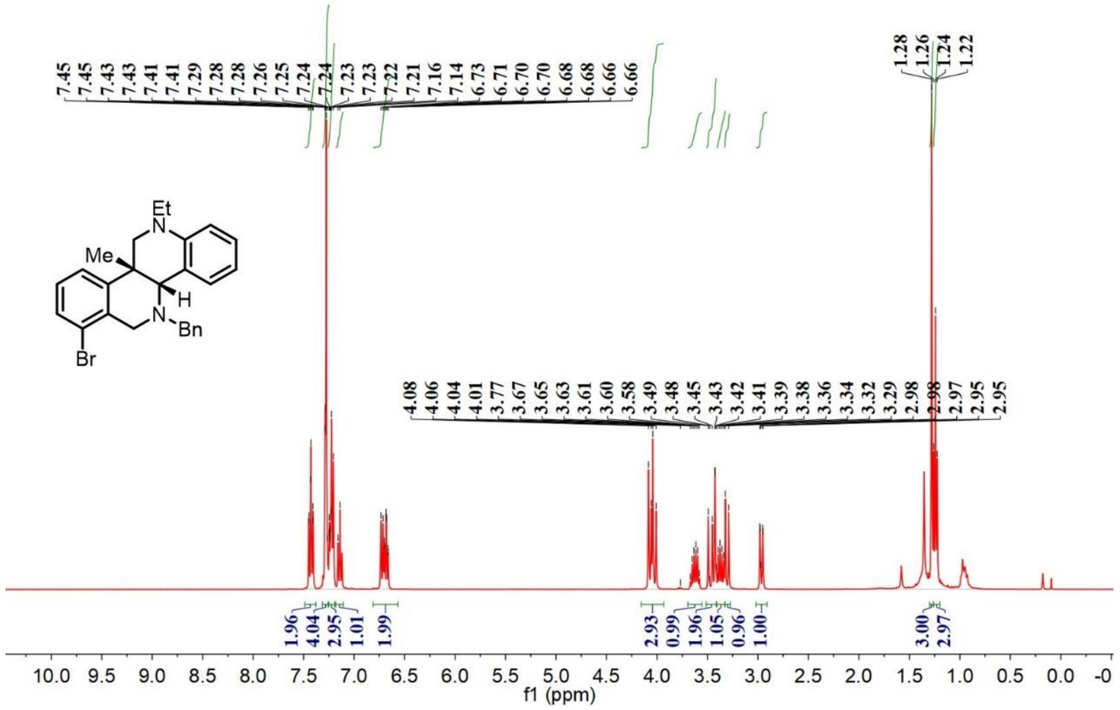

Supplementary Fig. 134 ^{1}H-NMR spectrum of P57

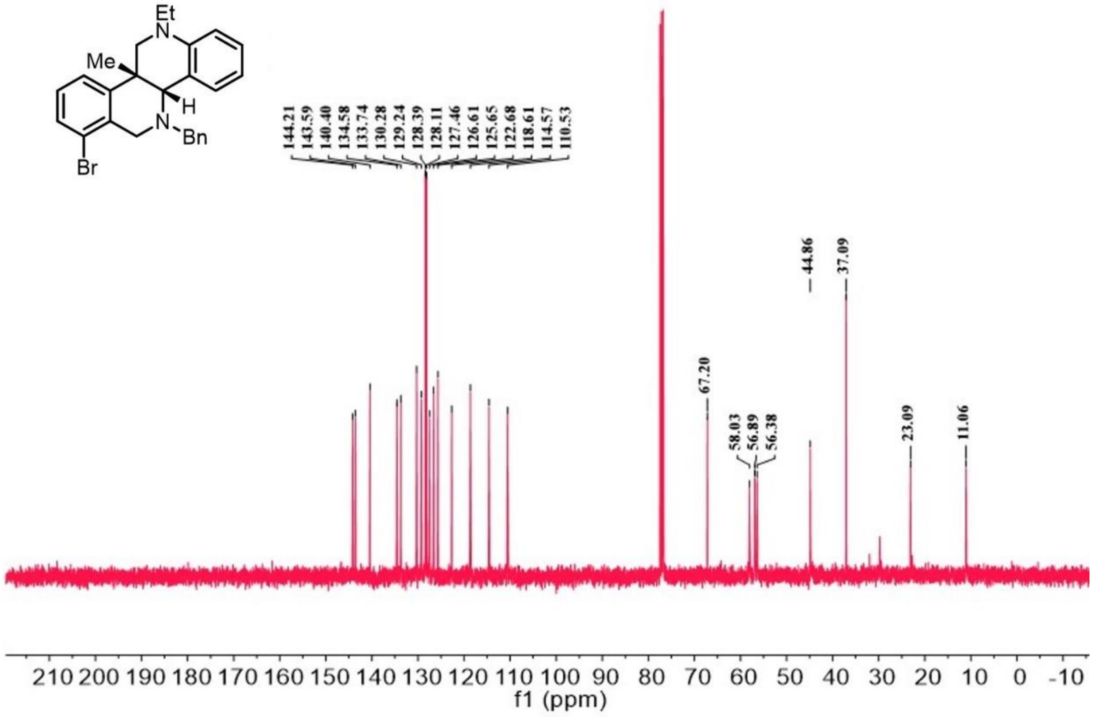

Supplementary Fig. 135 ^{13}C-NMR spectrum of P57

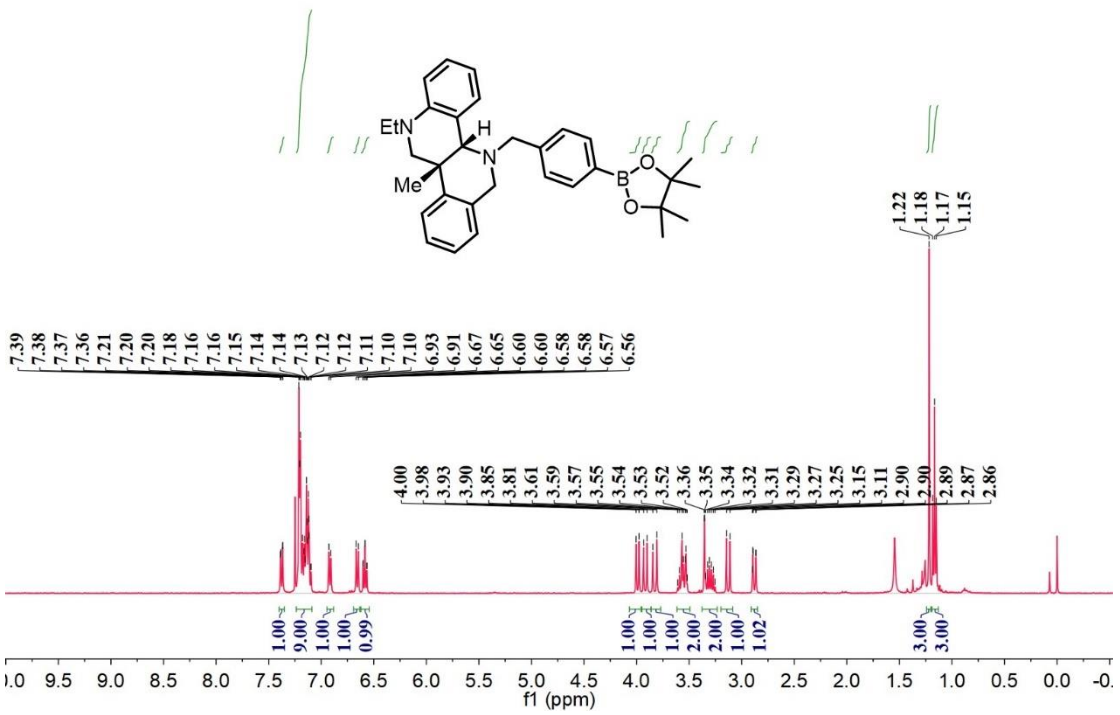

Supplementary Fig. 136 ^{1}H-NMR spectrum of P58

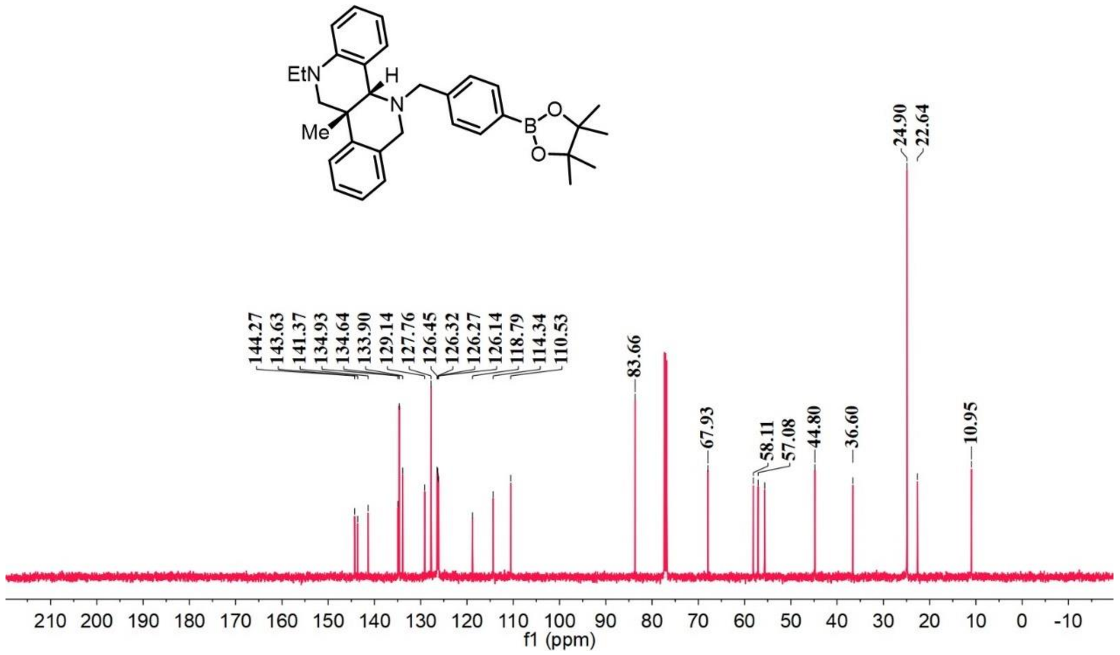

Supplementary Fig. 137 ^{13}C-NMR spectrum of P58

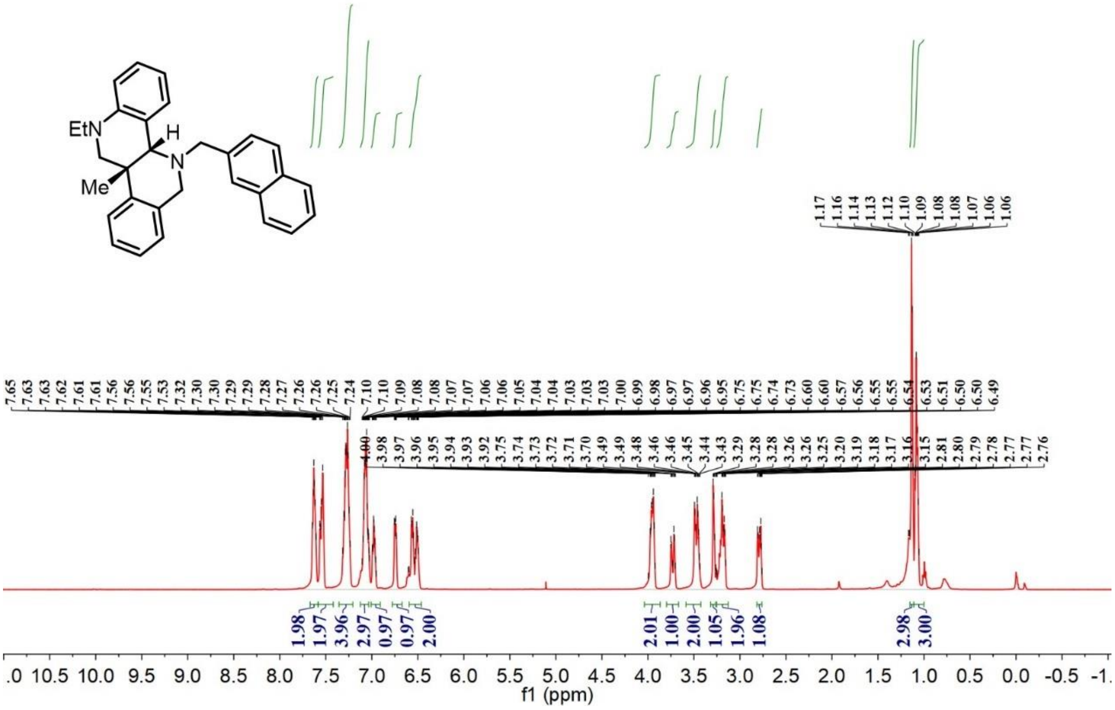

Supplementary Fig. 138 ^{1}H-NMR spectrum of P59

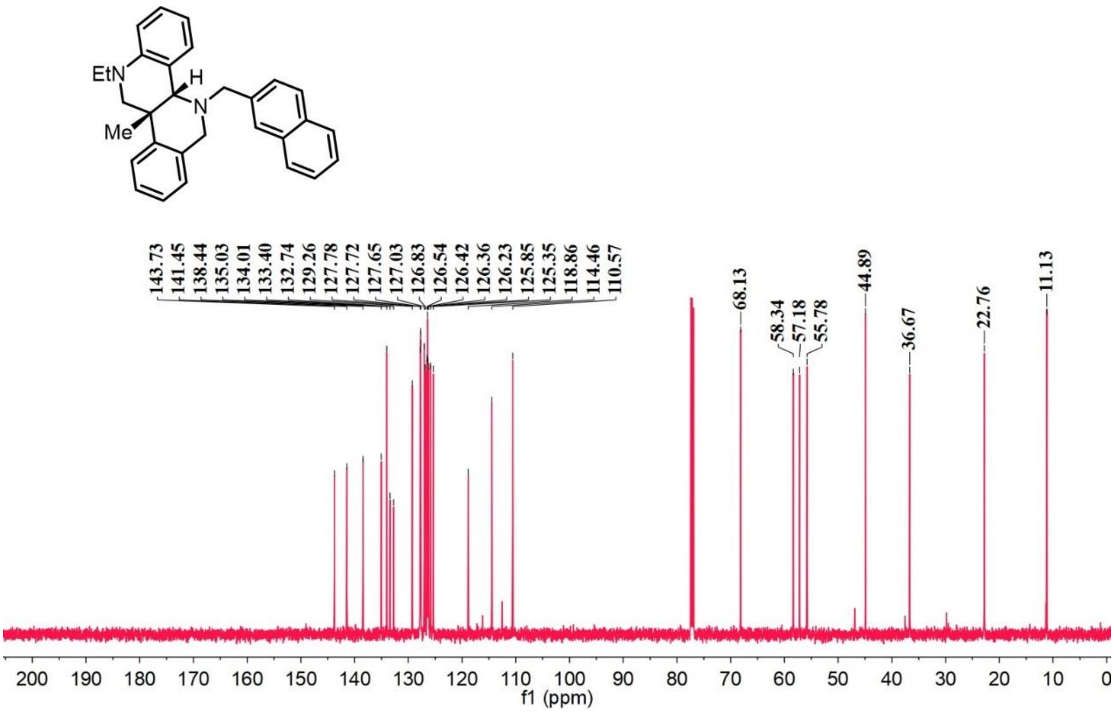

Supplementary Fig. 139 ^{13}C-NMR spectrum of P59

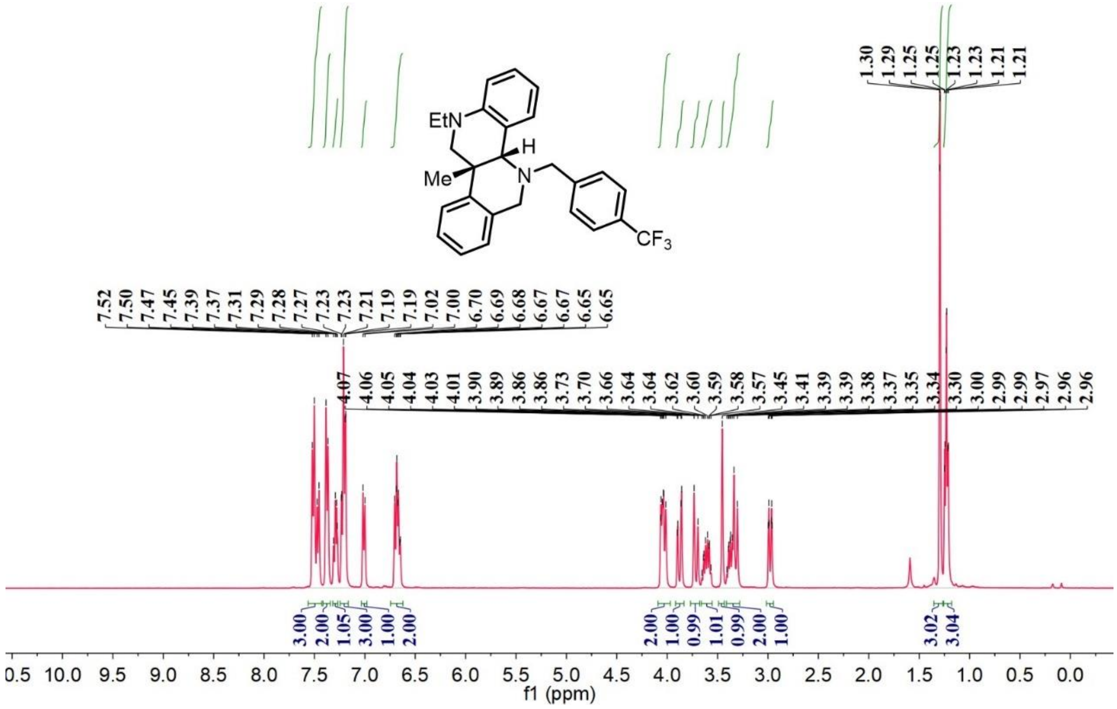

Supplementary Fig. 140 ^{1}H-NMR spectrum of P60

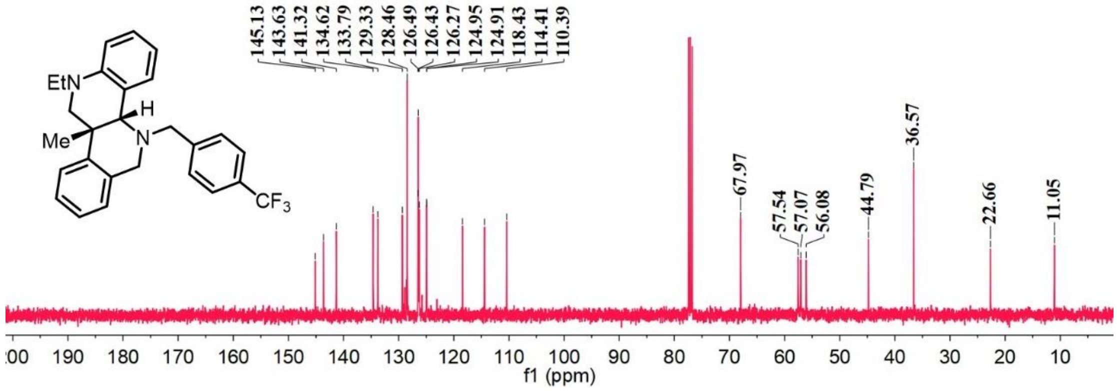

Supplementary Fig. 141 ^{13}C-NMR spectrum of P60

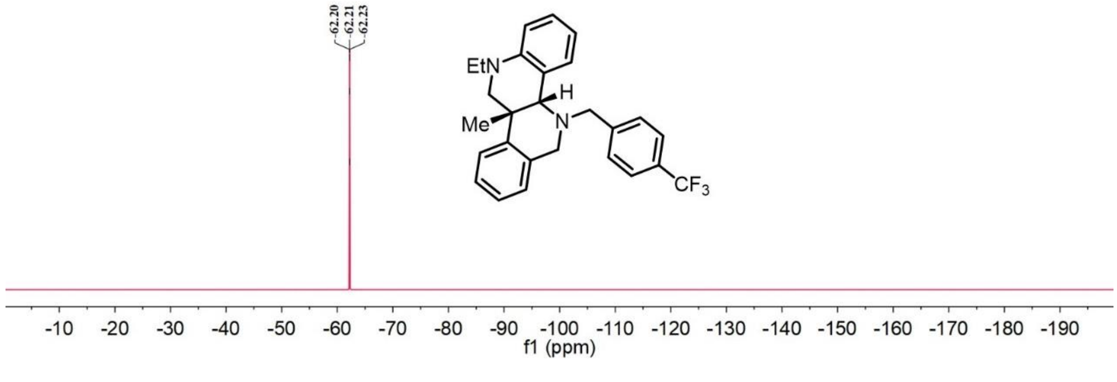

Supplementary Fig. 142 ^{19}F-NMR spectrum of P60

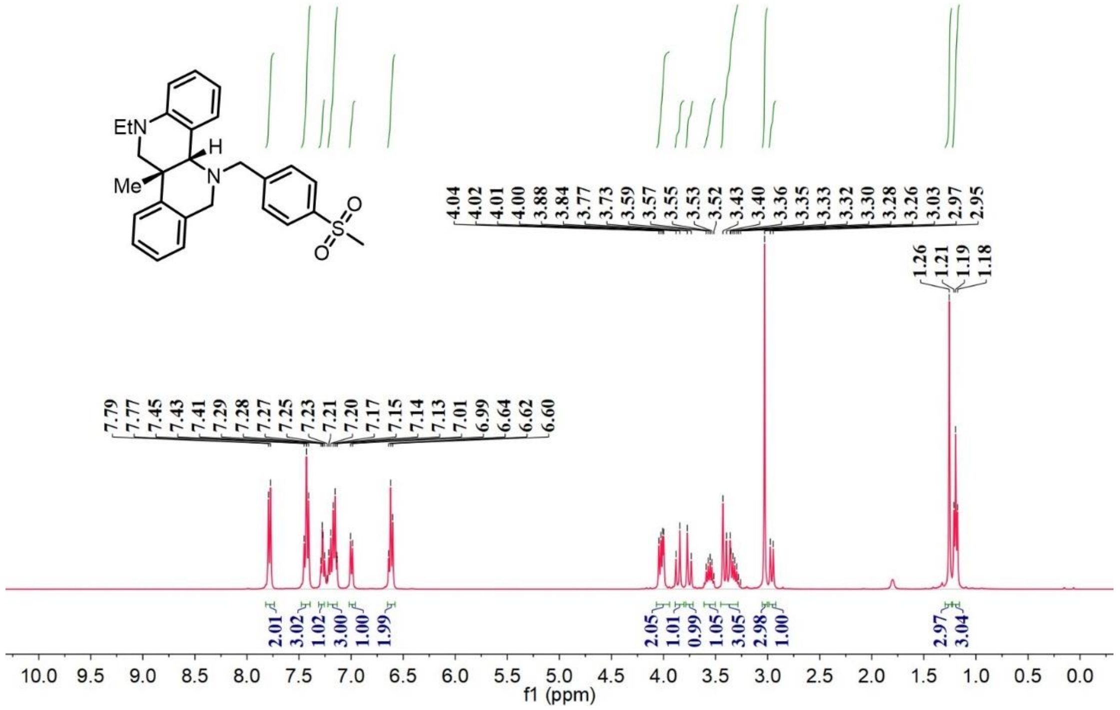

Supplementary Fig. 143 ^{1}H-NMR spectrum of P61

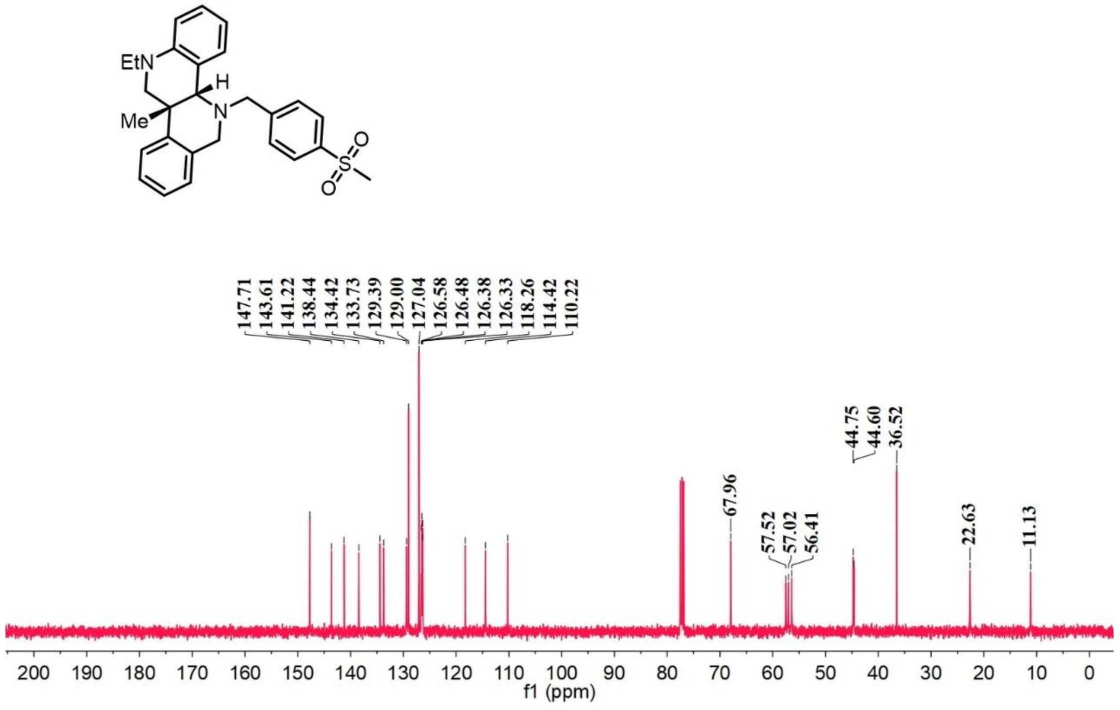

Supplementary Fig. 144 ^{13}C-NMR spectrum of P61

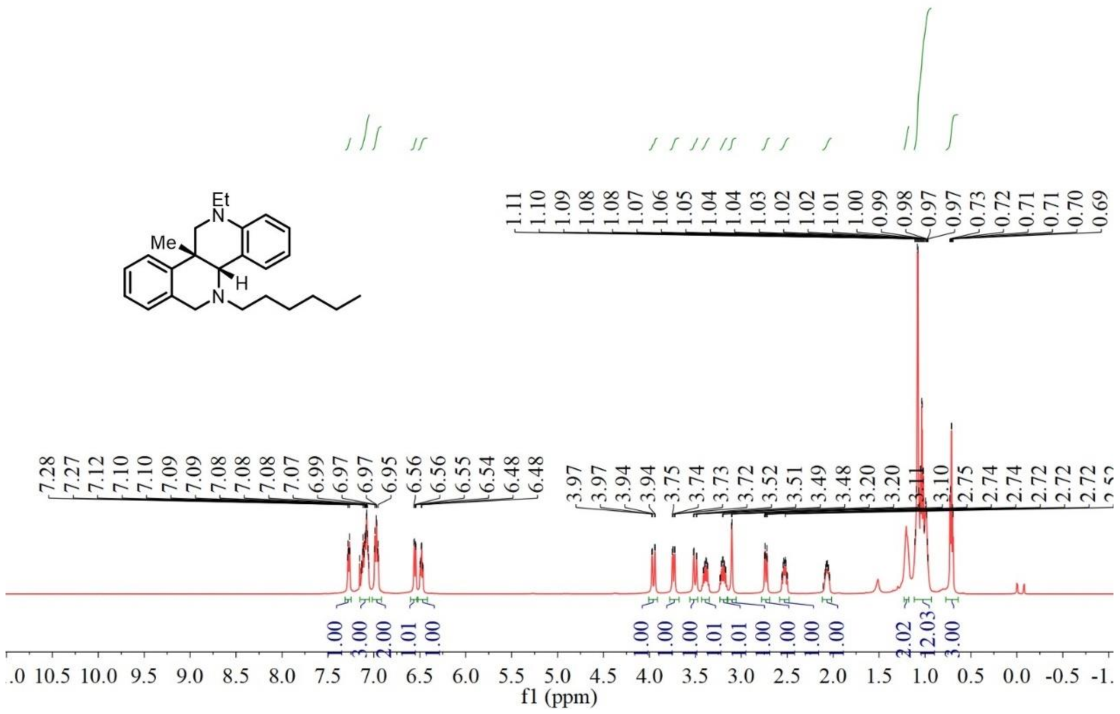

Supplementary Fig. 145 ^{1}H-NMR spectrum of P62

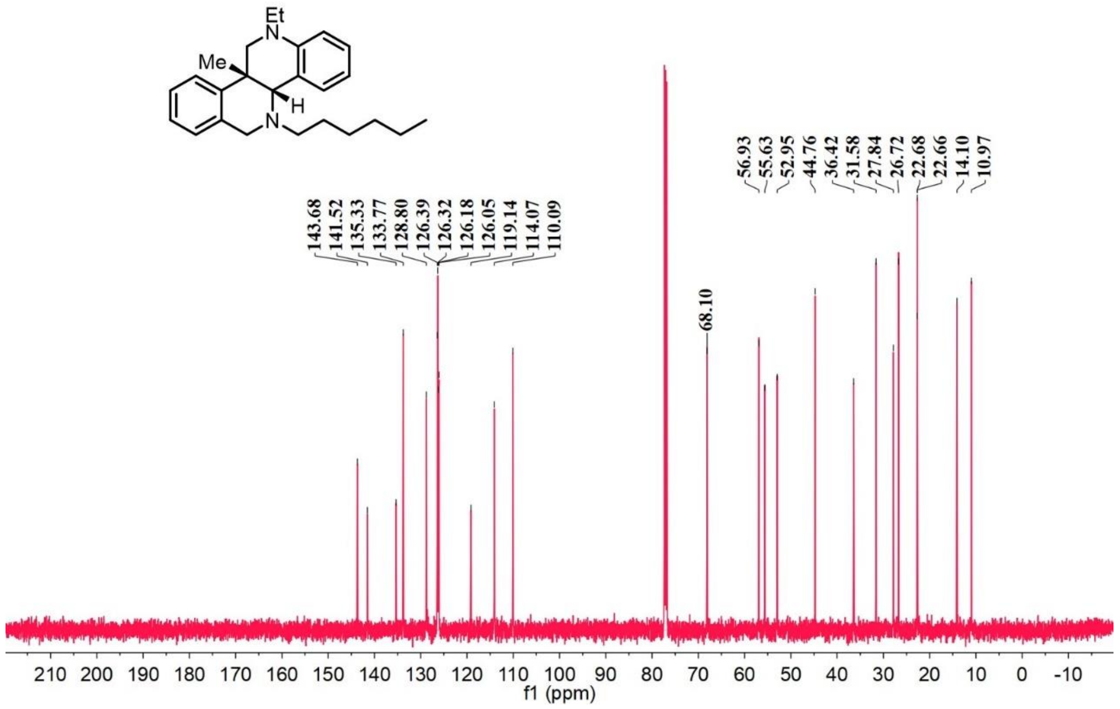

Supplementary Fig. 146 ^{13}C-NMR spectrum of P62

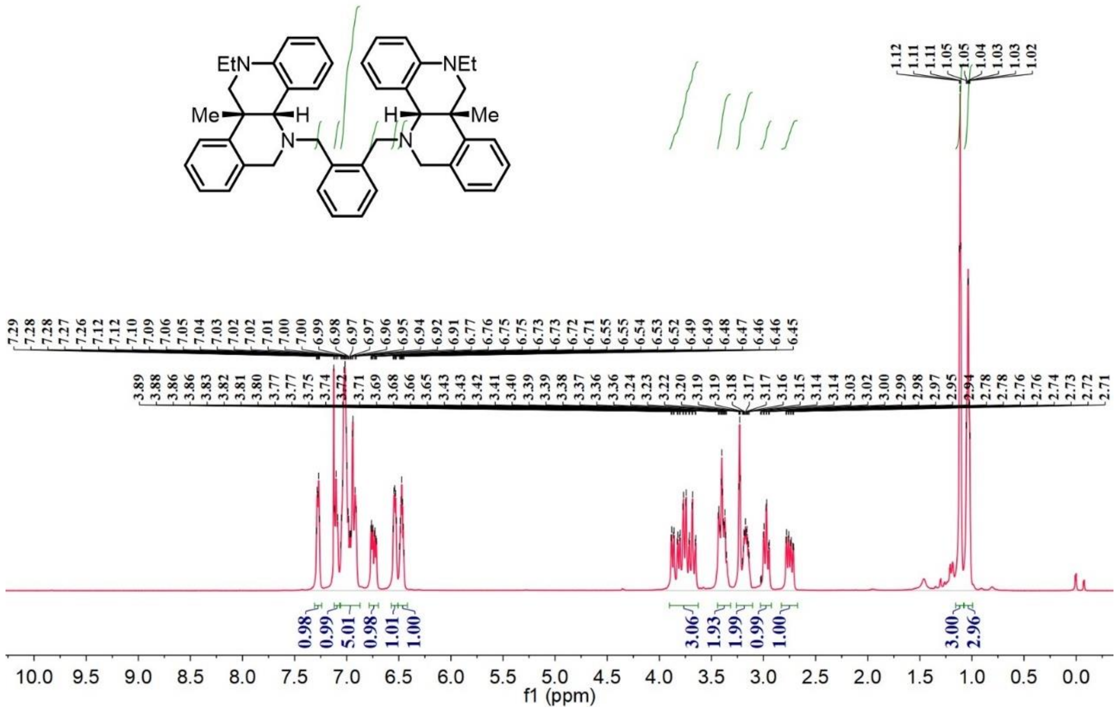

Supplementary Fig. 147 ^{1}H-NMR spectrum of P63

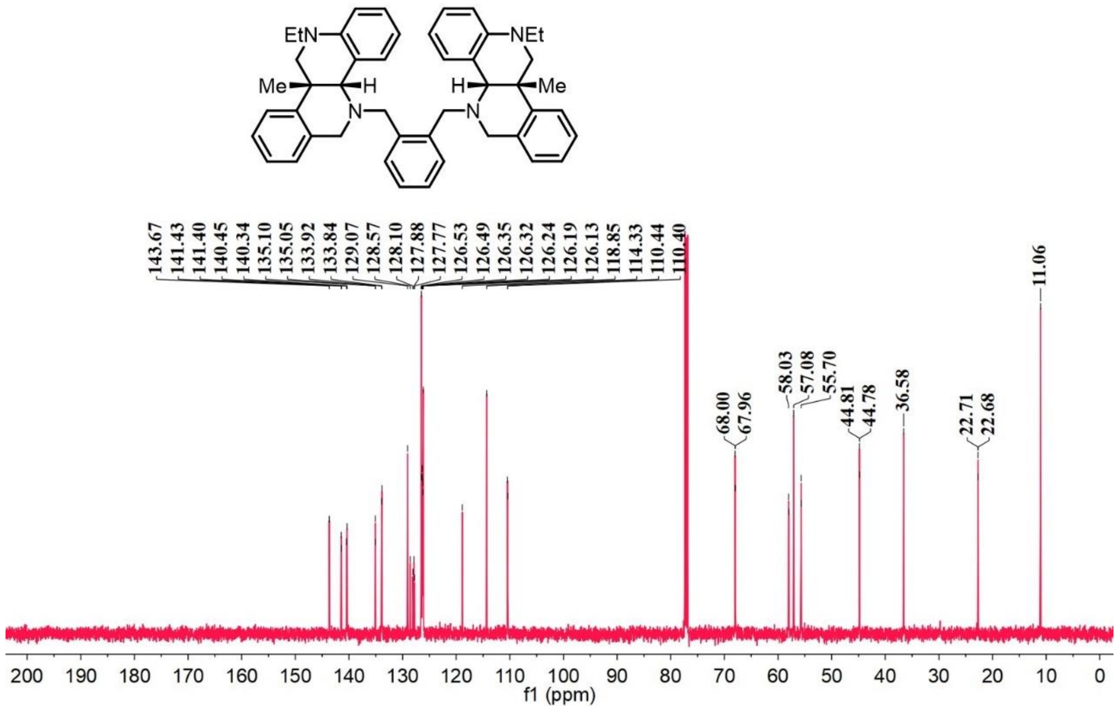

Supplementary Fig. 148 ^{13}C-NMR spectrum of P63

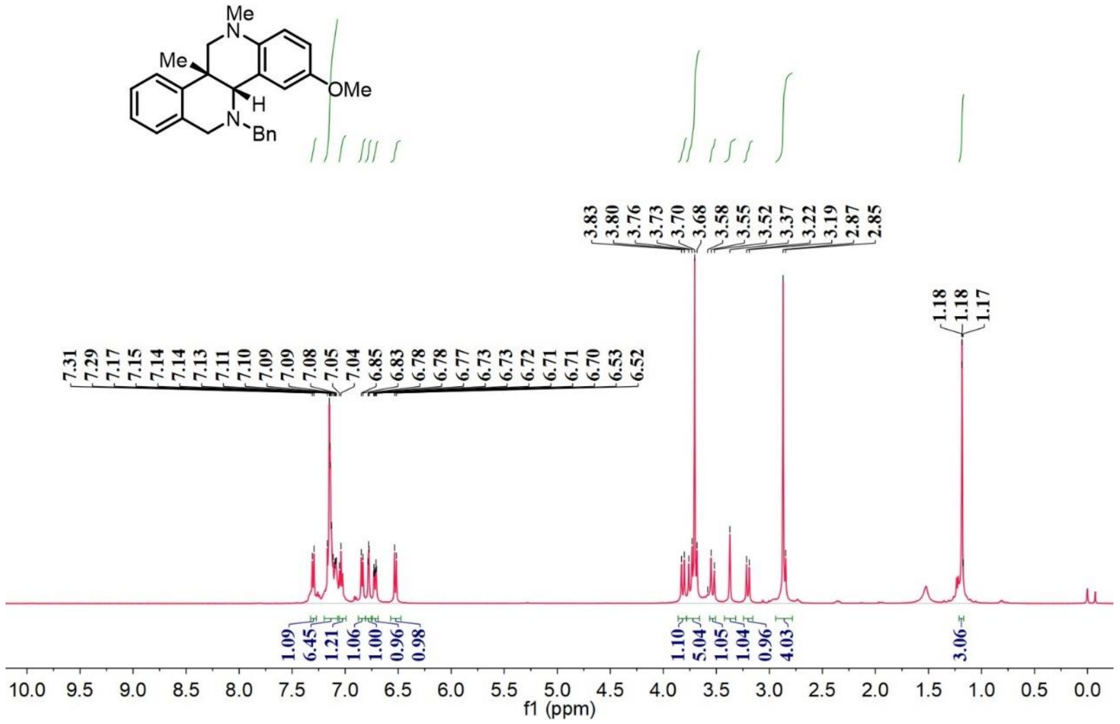

Supplementary Fig. 149 ^{1}H-NMR spectrum of P64

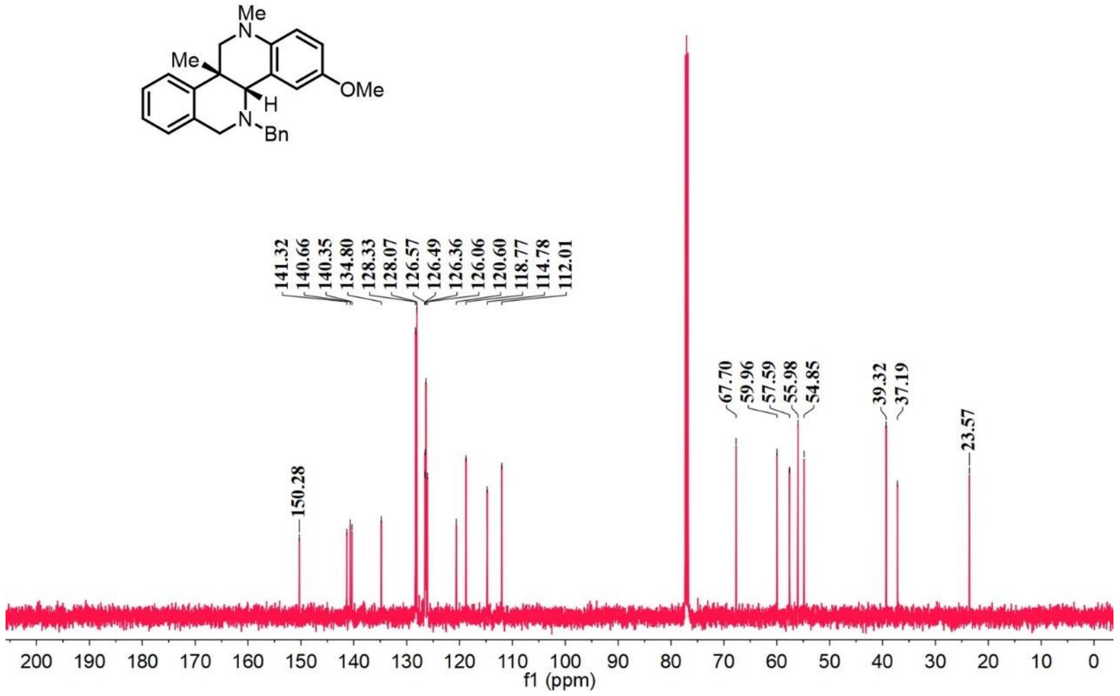

Supplementary Fig. 150 ^{13}C-NMR spectrum of P64

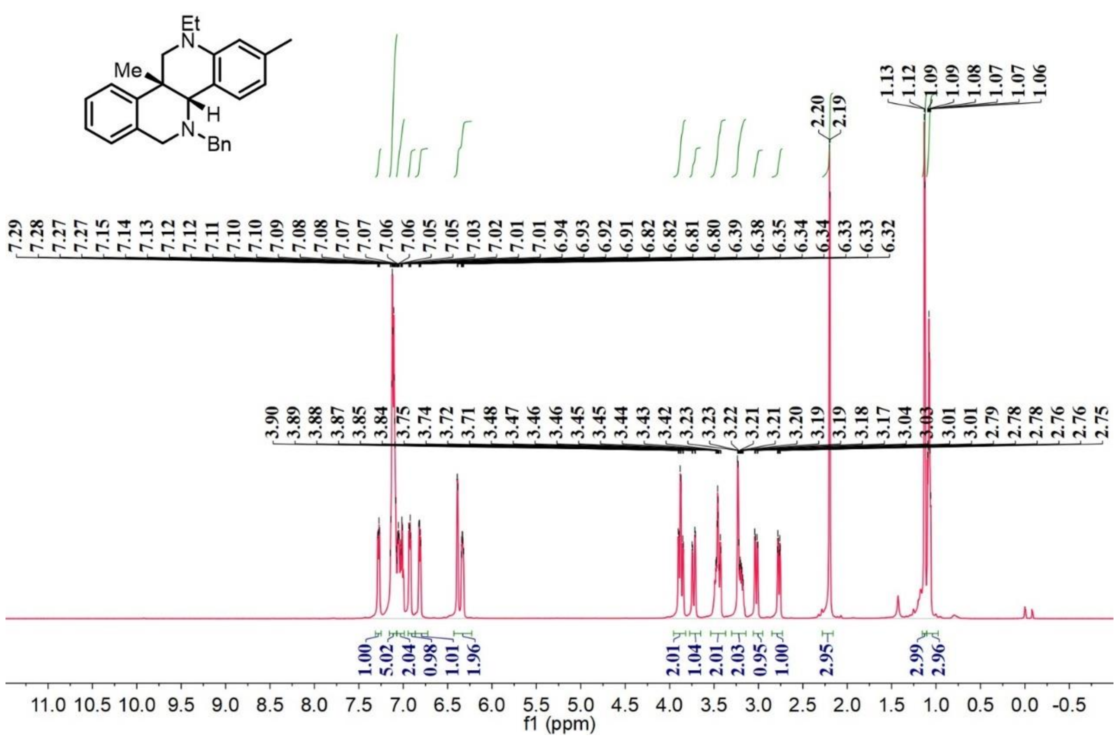

Supplementary Fig. 151 ^{1}H-NMR spectrum of P65

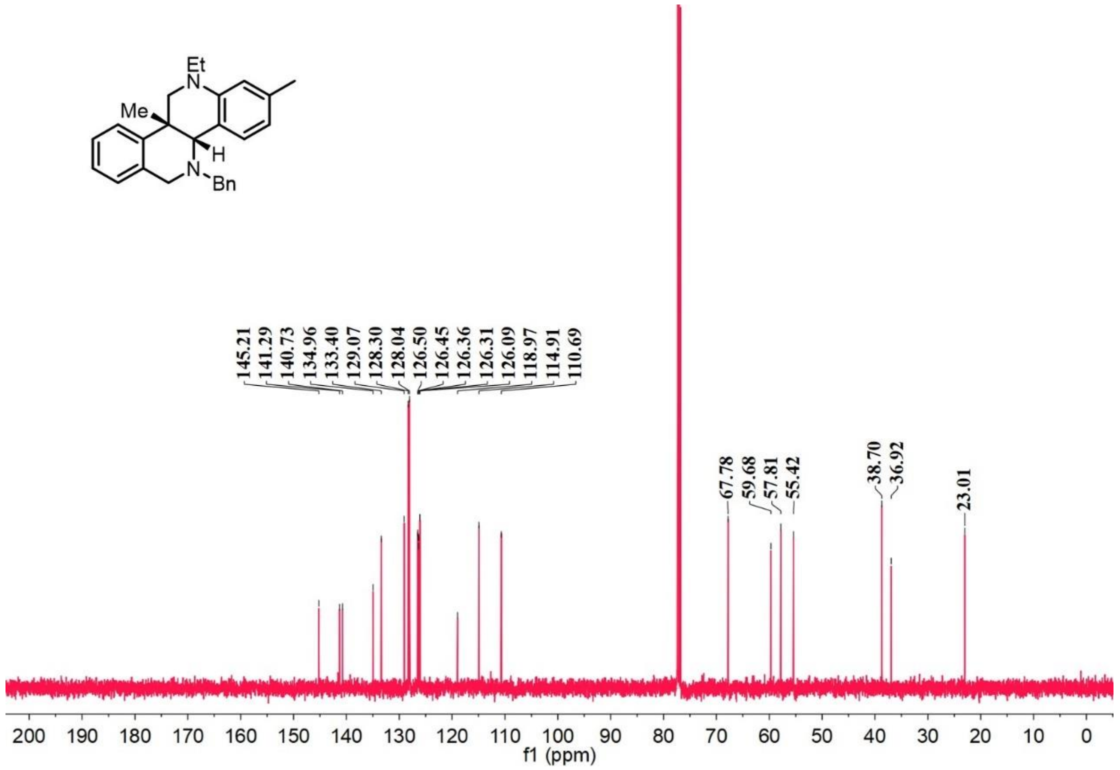

Supplementary Fig. 152 ^{13}C-NMR spectrum of P65

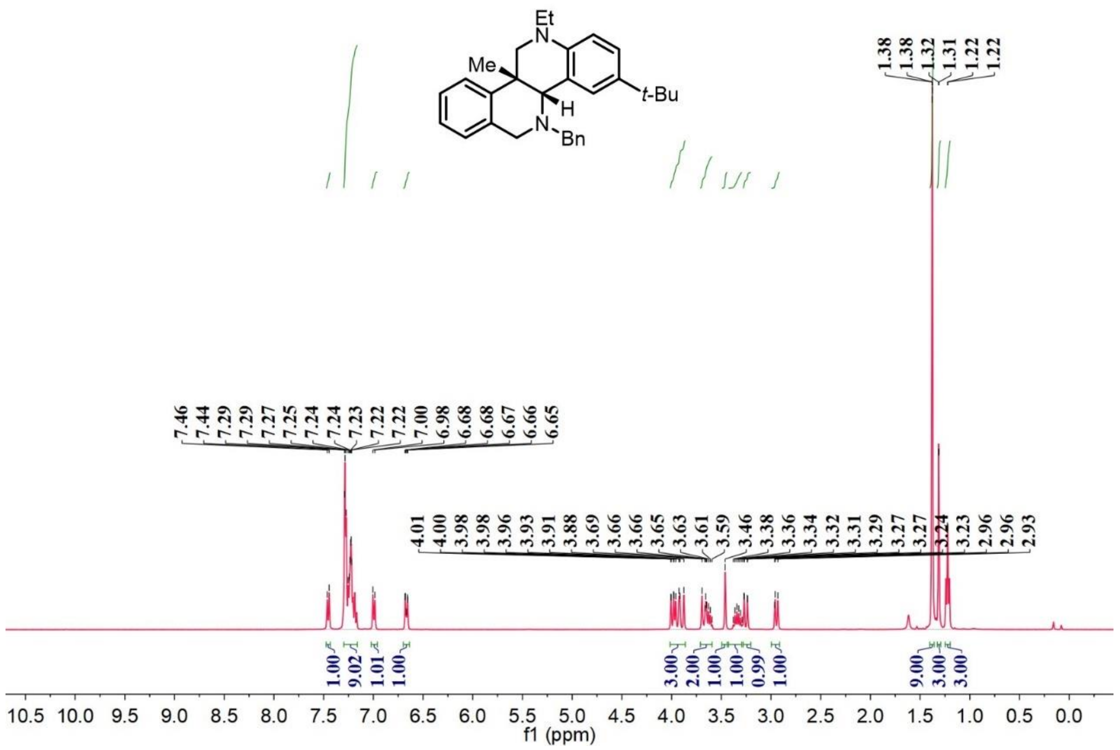

Supplementary Fig. 153 ¹H-NMR spectrum of P66

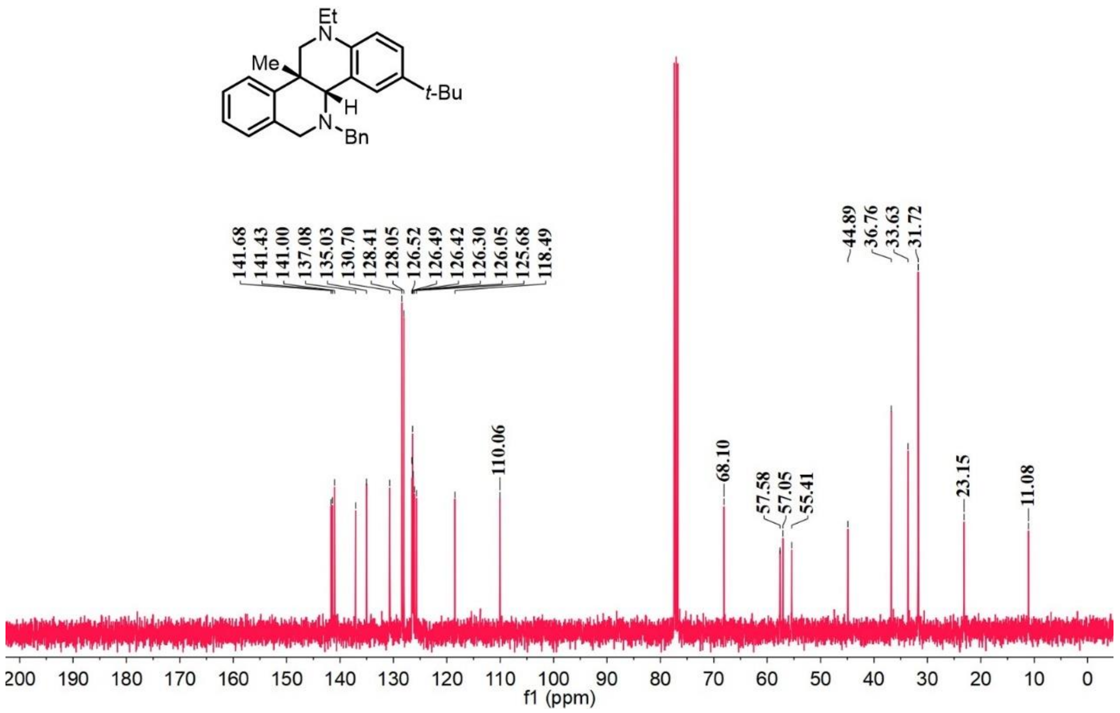

Supplementary Fig. 154 ¹³C-NMR spectrum of P66

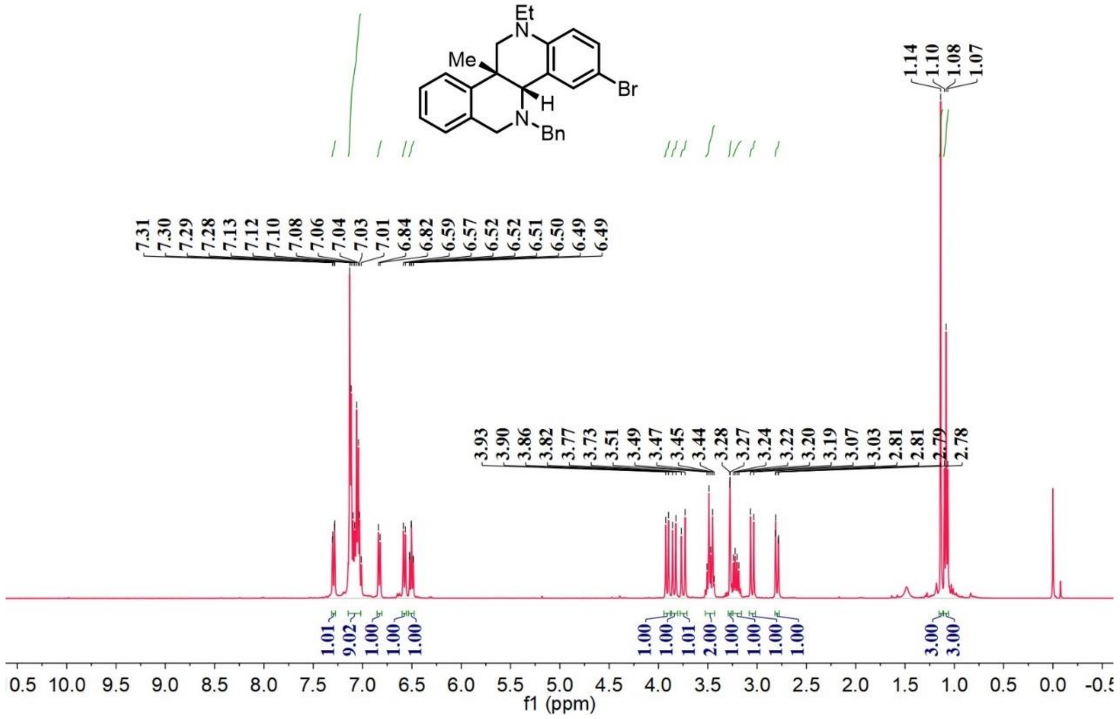

Supplementary Fig. 155 ¹H-NMR spectrum of P67

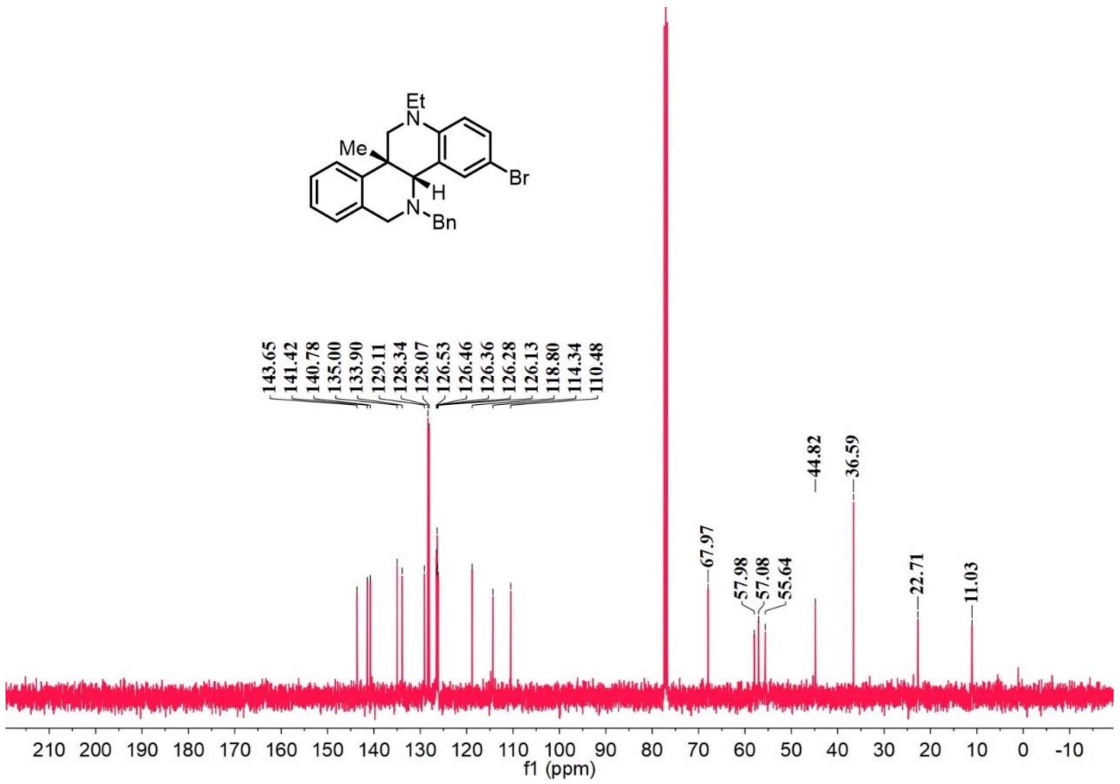

Supplementary Fig. 156 ¹³C-NMR spectrum of P67

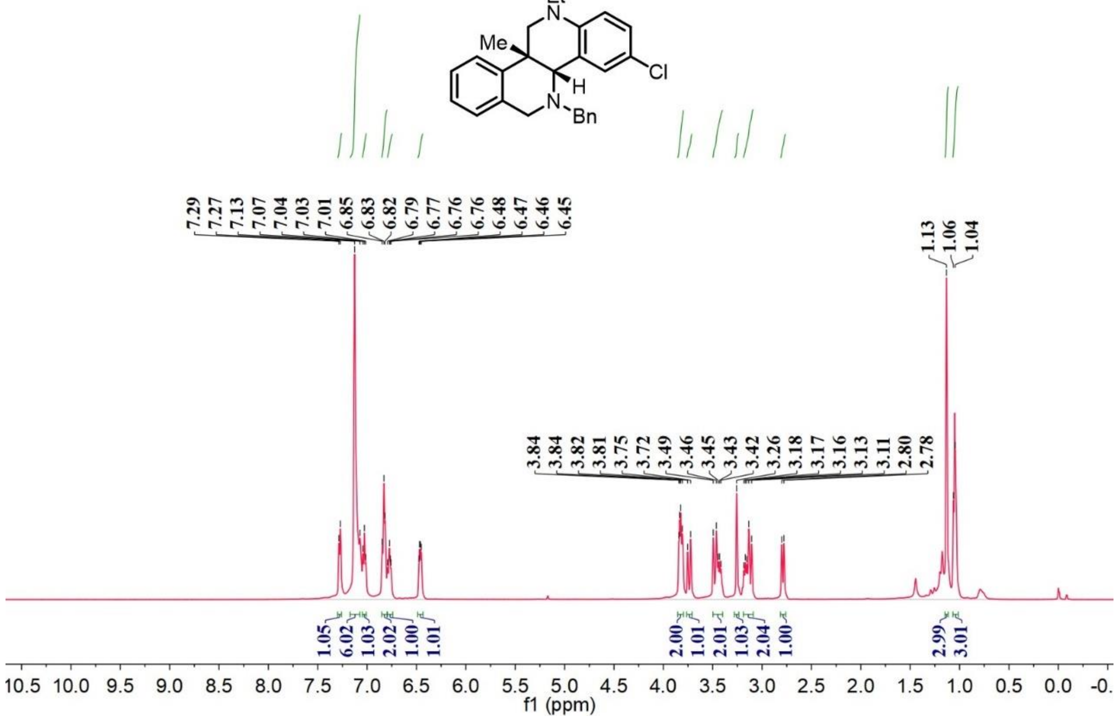

Supplementary Fig. 157 ^{1}H-NMR spectrum of P68

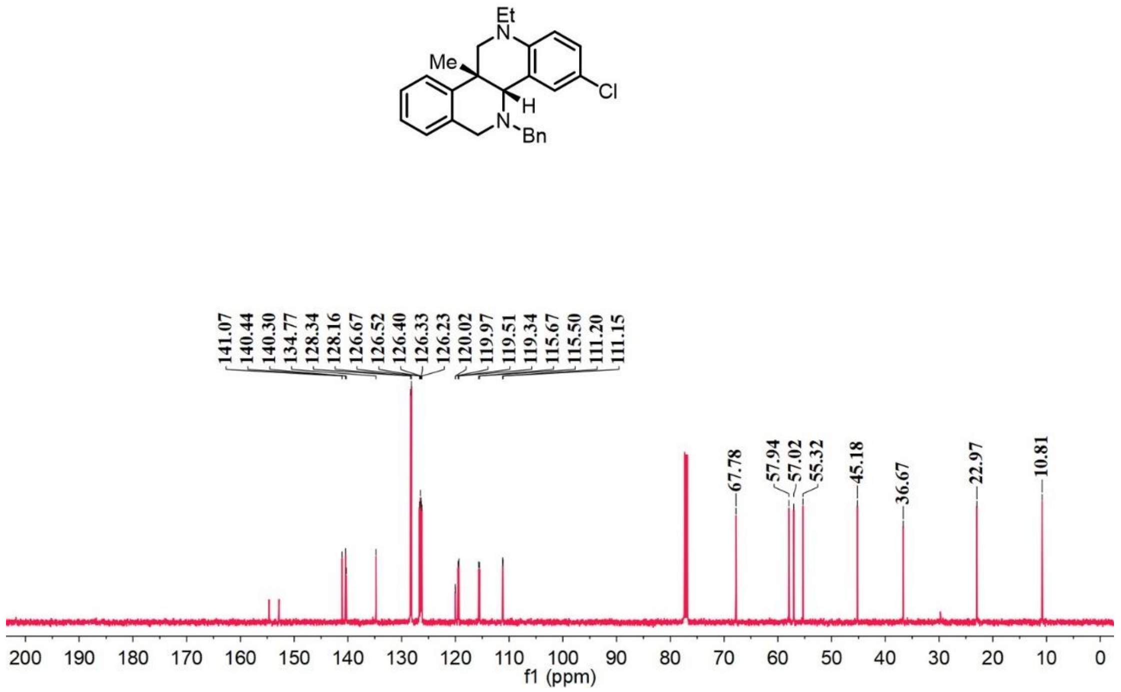

Supplementary Fig. 158 ^{13}C-NMR spectrum of P68

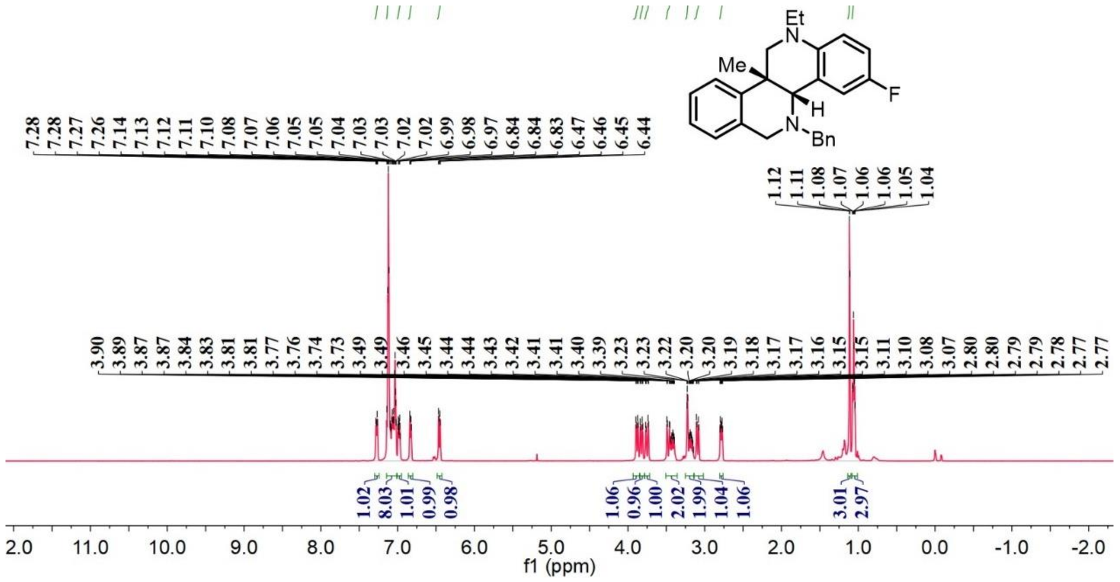

Supplementary Fig. 159 ^{1}H-NMR spectrum of P69

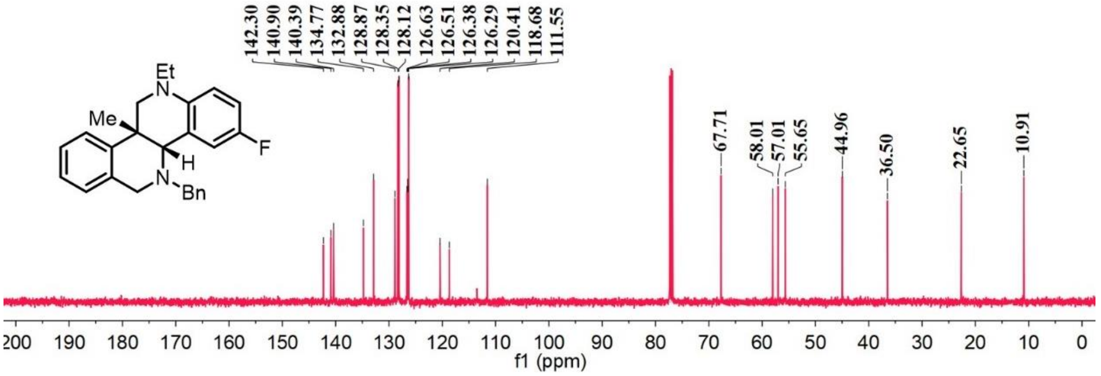

Supplementary Fig. 160 ^{13}C-NMR spectrum of P69

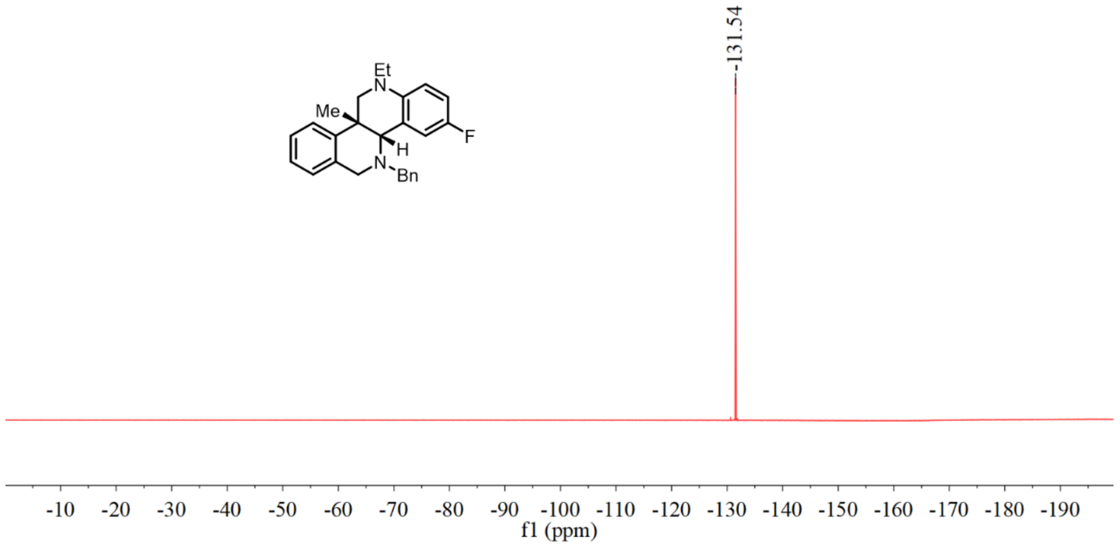

Supplementary Fig. 161 ^{19}F -NMR spectrum of P69

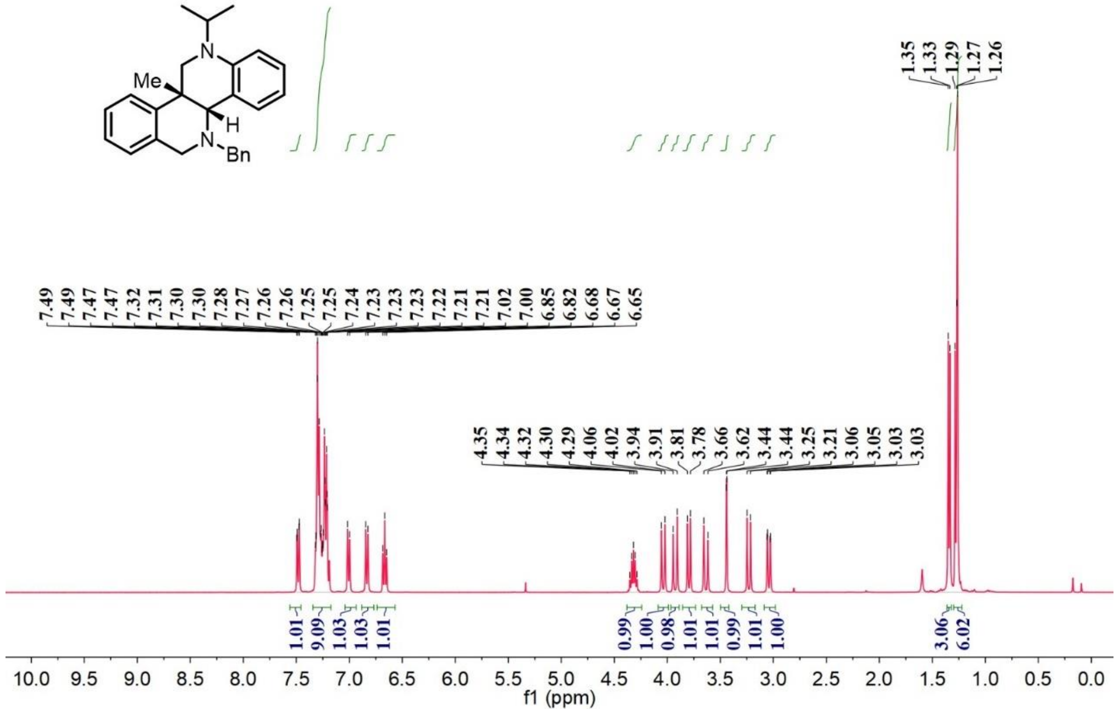

Supplementary Fig. 162 ^{1}H-NMR spectrum of P70

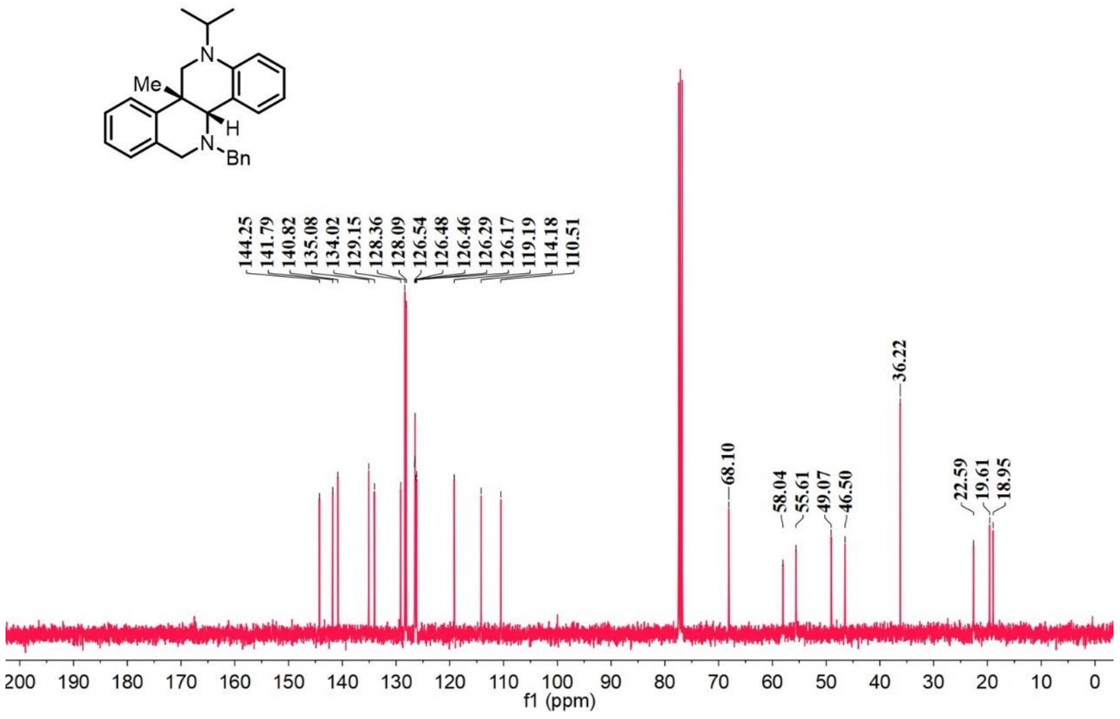

Supplementary Fig. 163 ^{13}C-NMR spectrum of P70

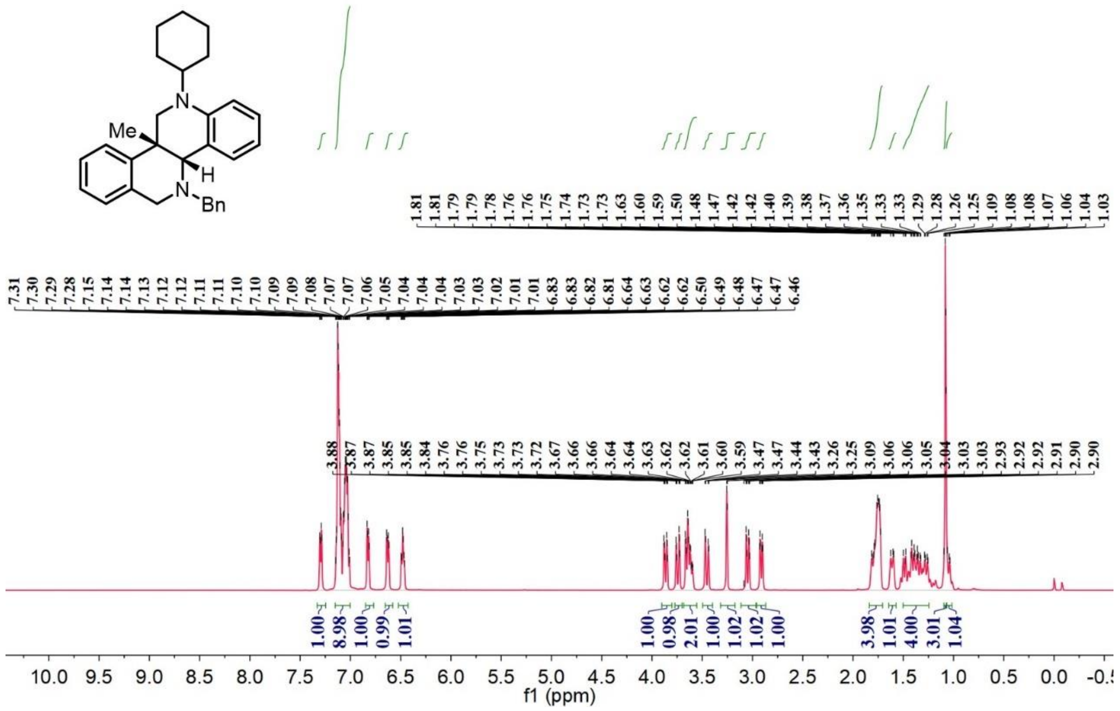

Supplementary Fig. 164 ^{1}H-NMR spectrum of P71

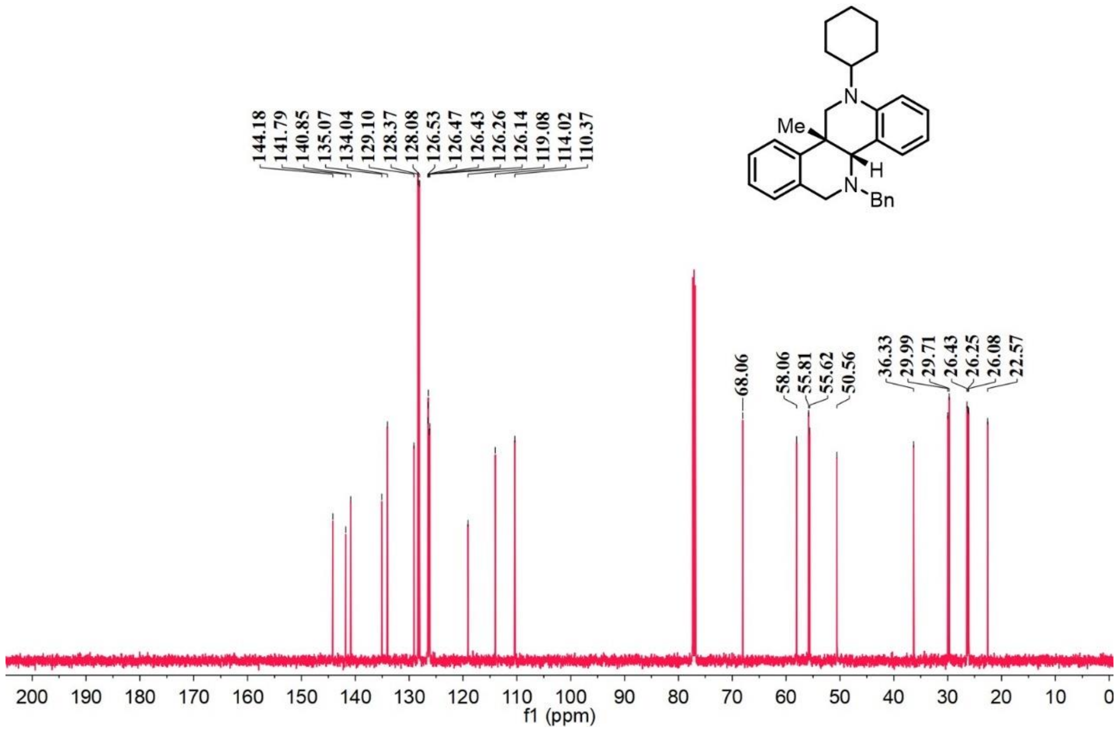

Supplementary Fig. 165 ^{13}C-NMR spectrum of P71

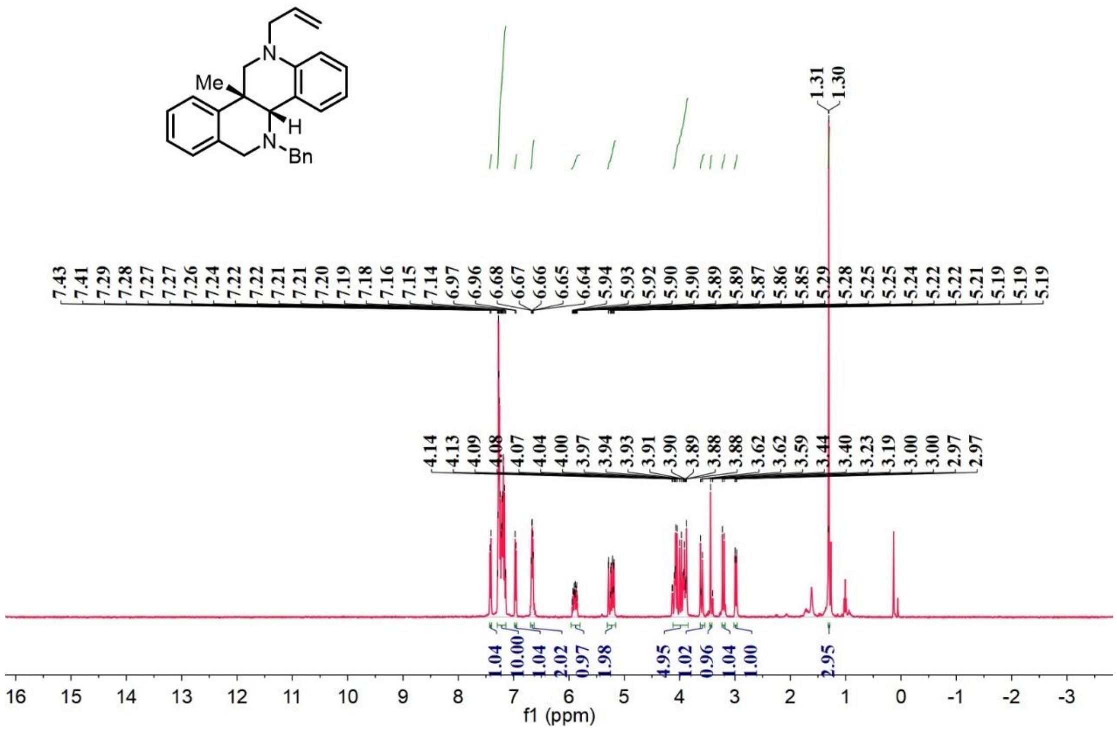

Supplementary Fig. 166 ^{1}H-NMR spectrum of P72

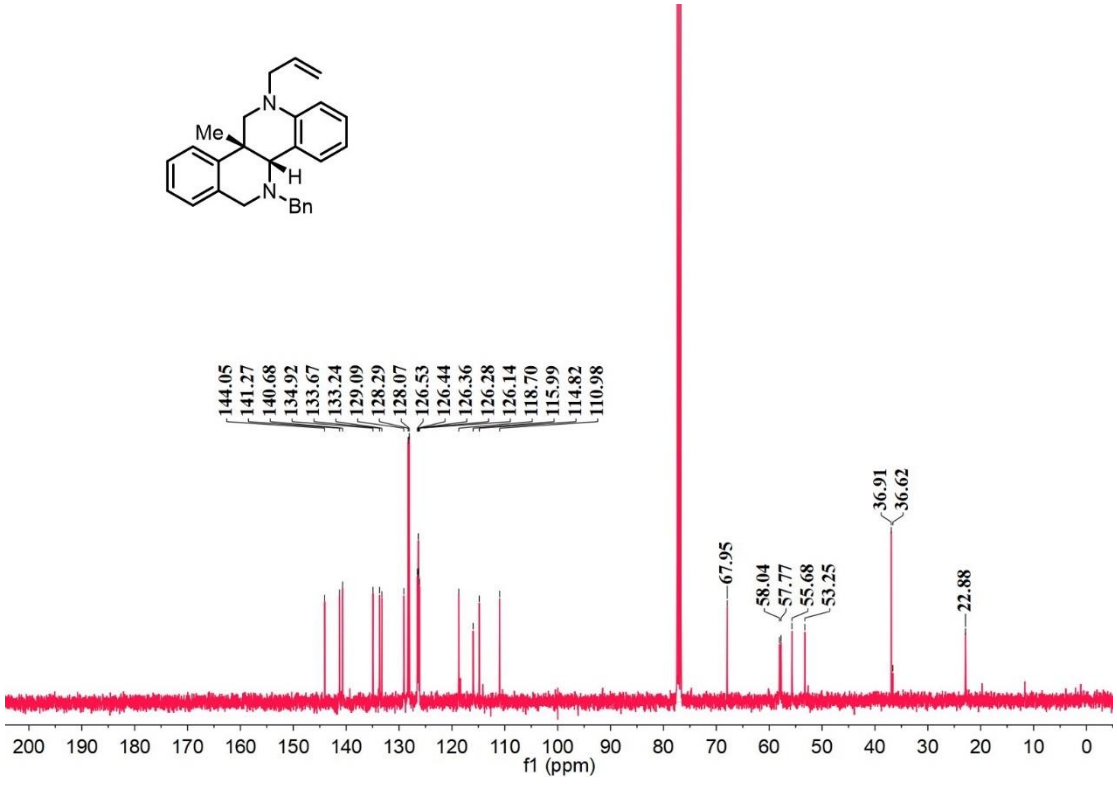

Supplementary Fig. 167 ^{13}C-NMR spectrum of P72

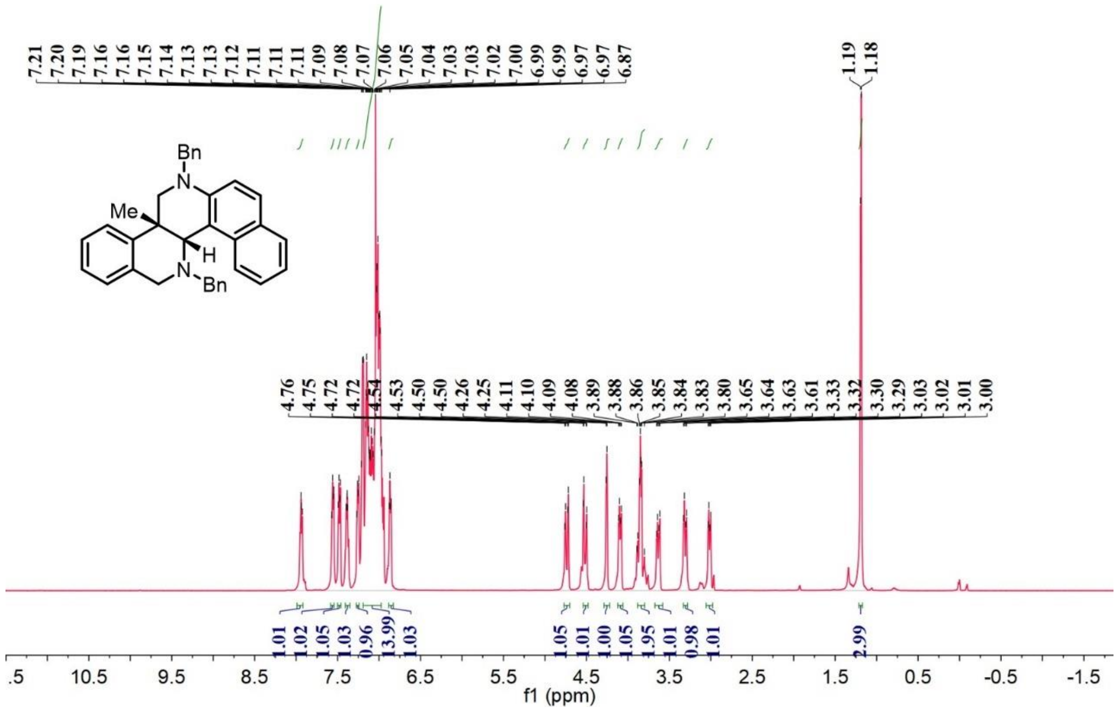

Supplementary Fig. 168 ^{1}H-NMR spectrum of P73

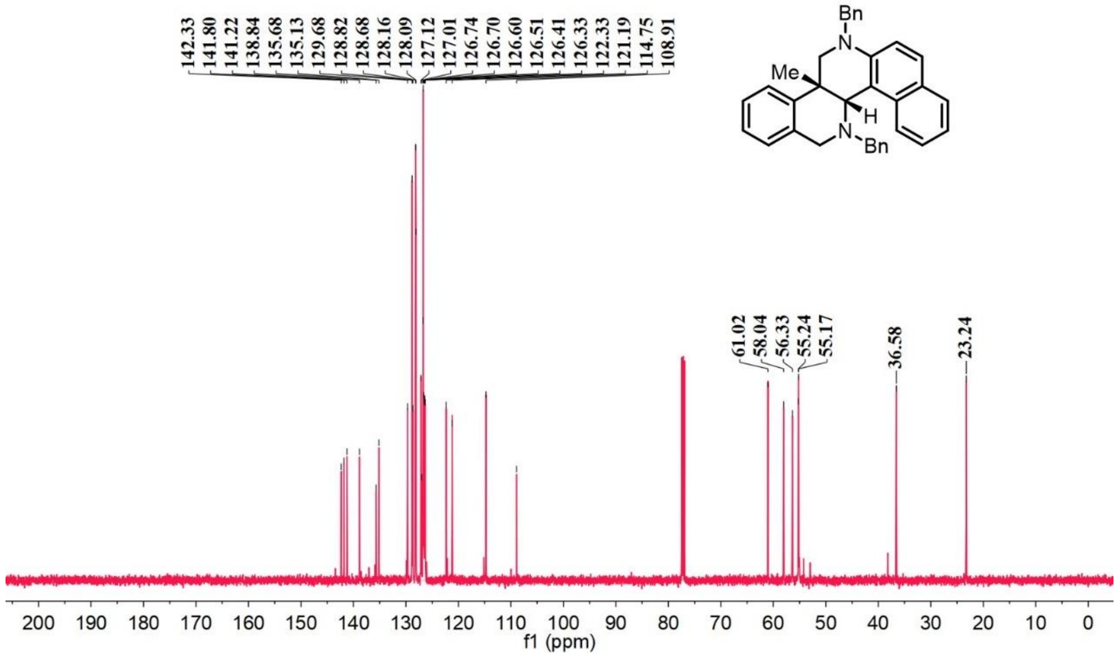

Supplementary Fig. 169 ^{13}C-NMR spectrum of P73

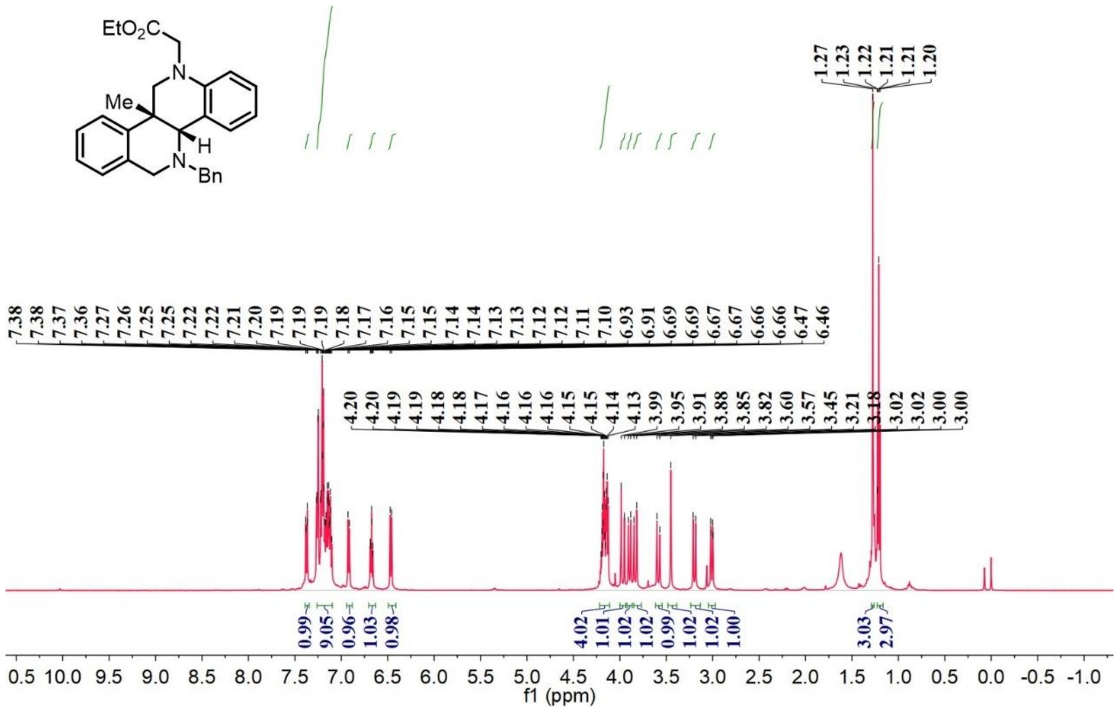

Supplementary Fig. 170 ^{1}H-NMR spectrum of P74

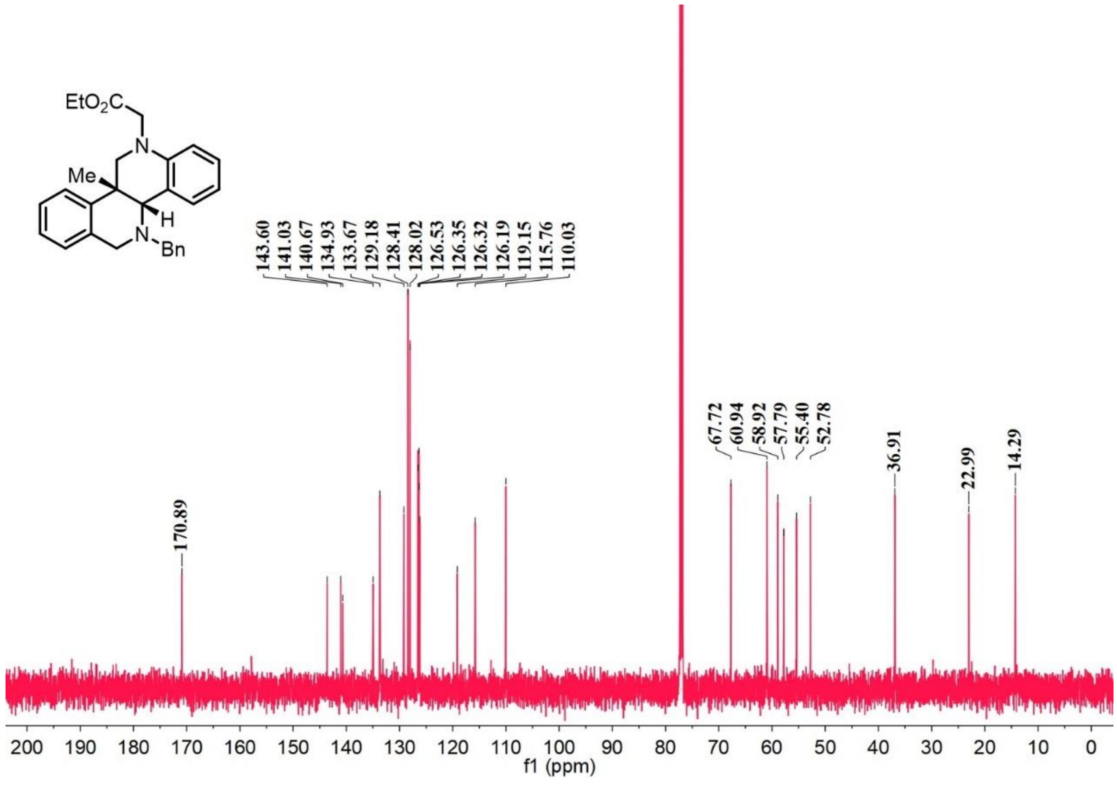

Supplementary Fig. 171 ^{13}C-NMR spectrum of P74

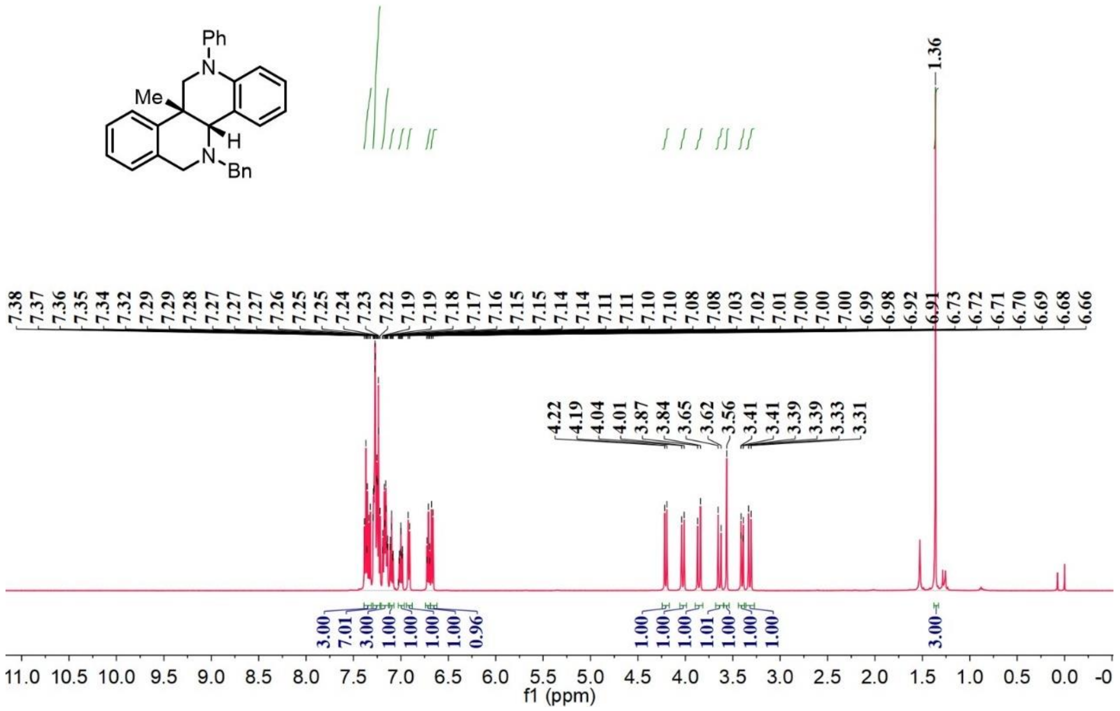

Supplementary Fig. 172 ^{1}H-NMR spectrum of P75

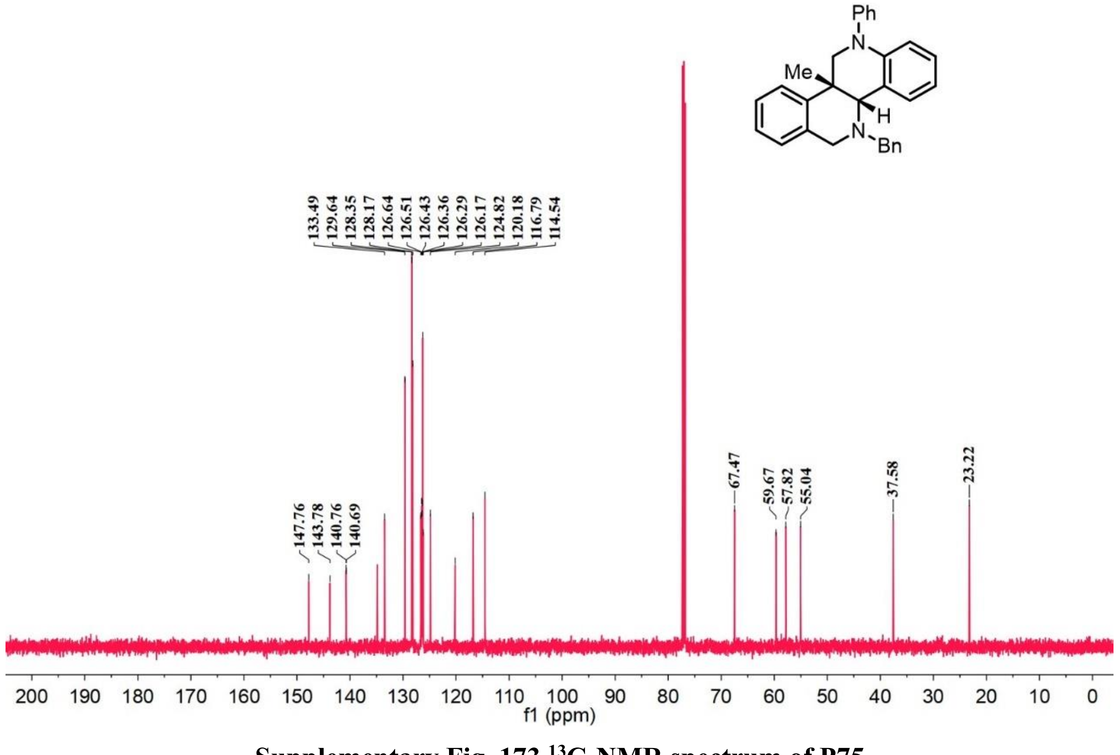

Supplementary Fig. 173 ^{13}C-NMR spectrum of P75

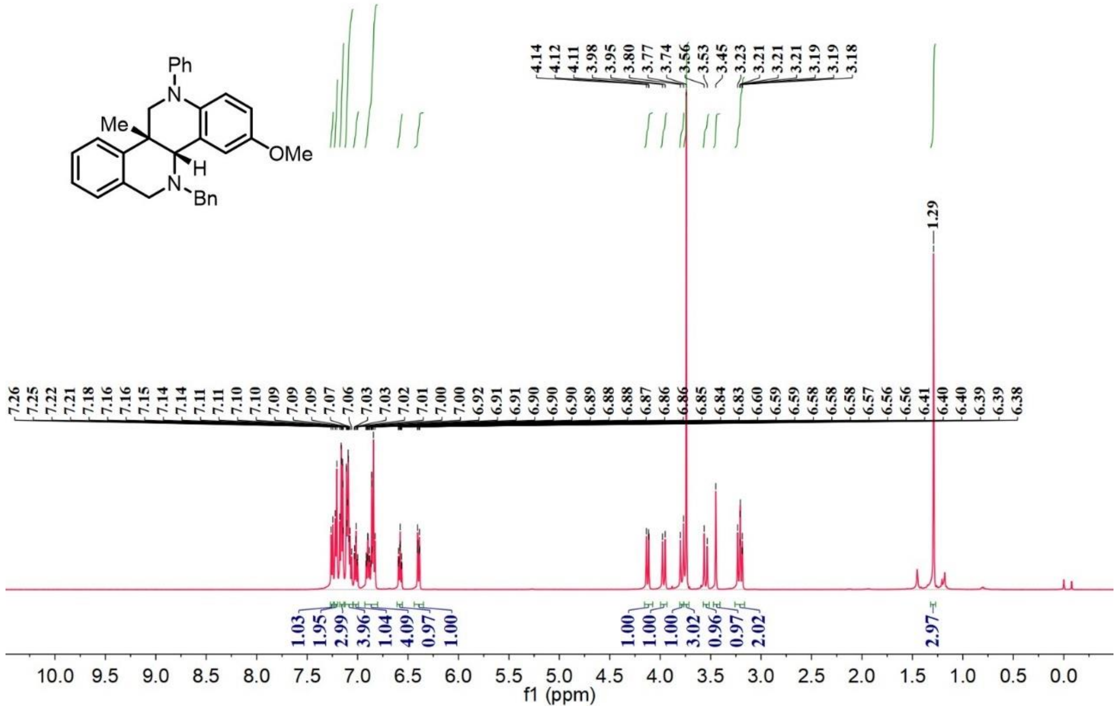

Supplementary Fig. 174 ^{1}H-NMR spectrum of P76

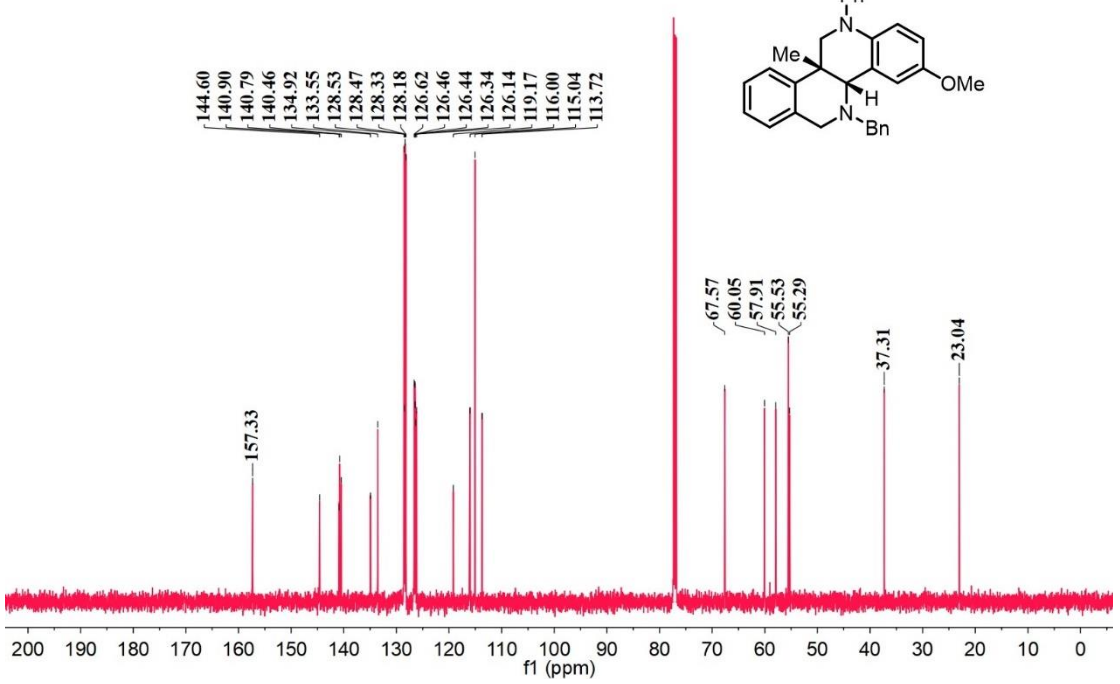

Supplementary Fig. 175 ^{13}C-NMR spectrum of P76

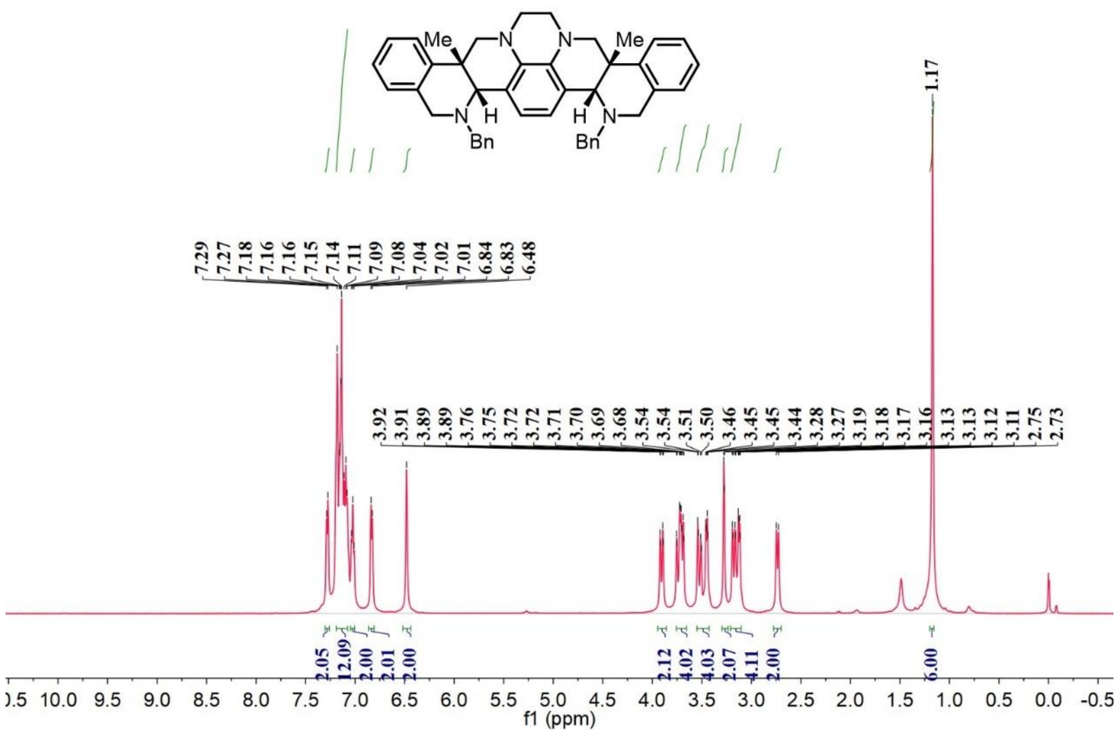

Supplementary Fig. 176 ^{1}H-NMR spectrum of P77

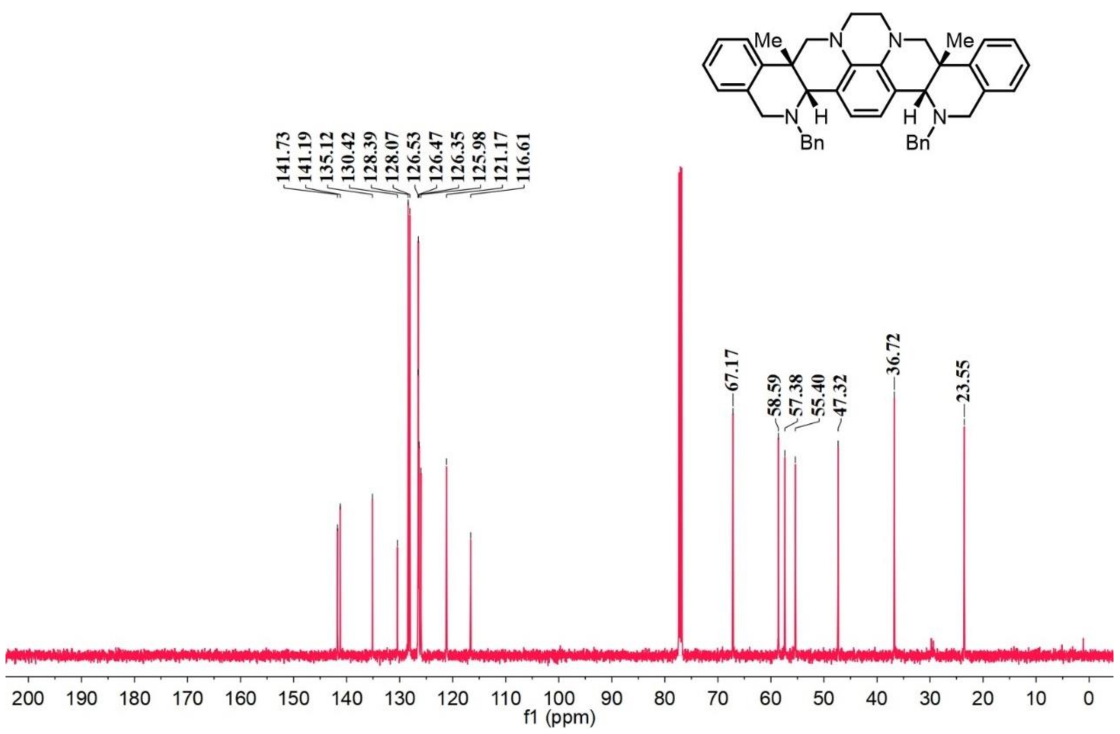

Supplementary Fig. 177 ^{13}C-NMR spectrum of P77

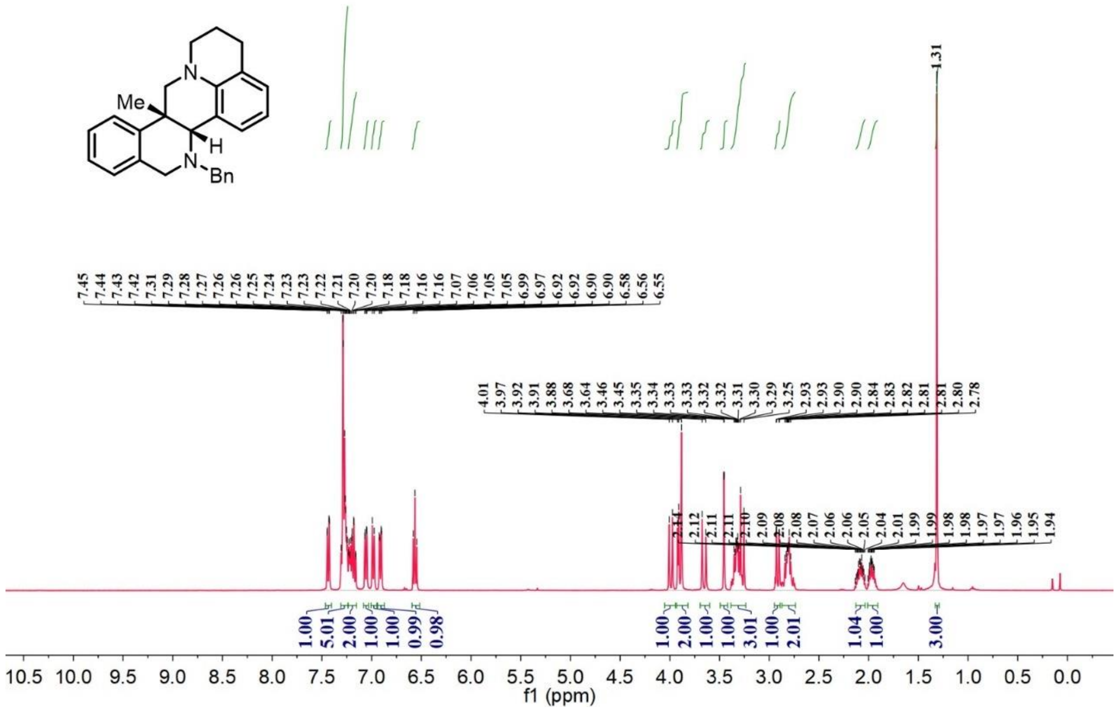

Supplementary Fig. 178 ^{1}H-NMR spectrum of P78

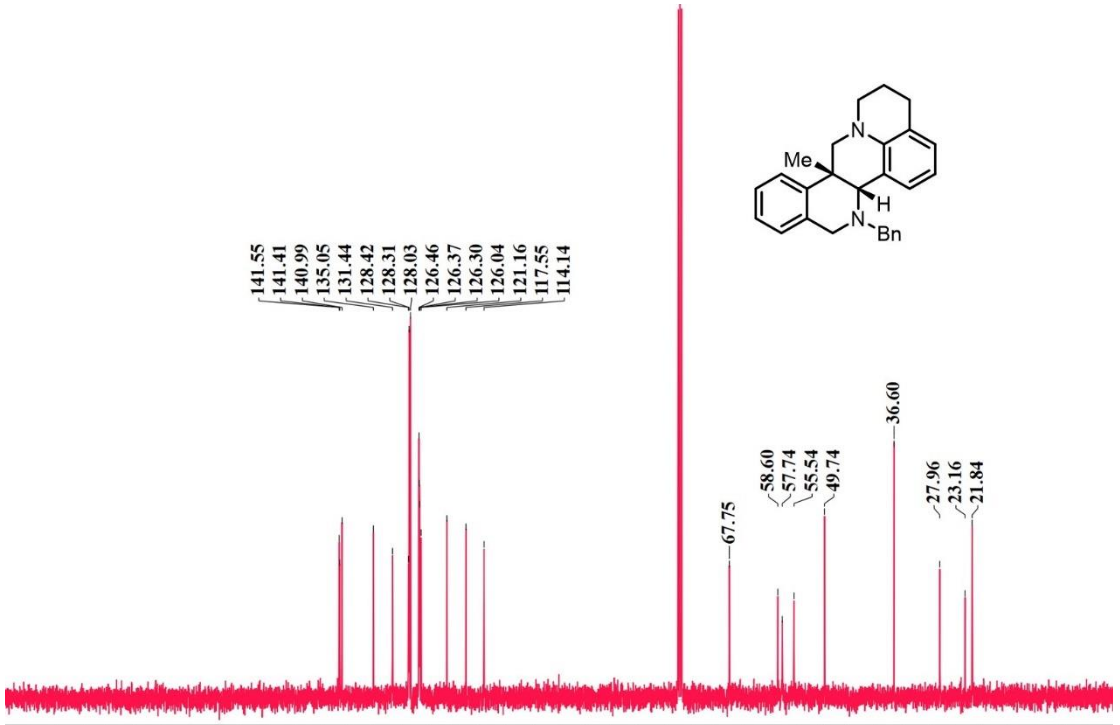

Supplementary Fig. 179 ^{13}C-NMR spectrum of P78

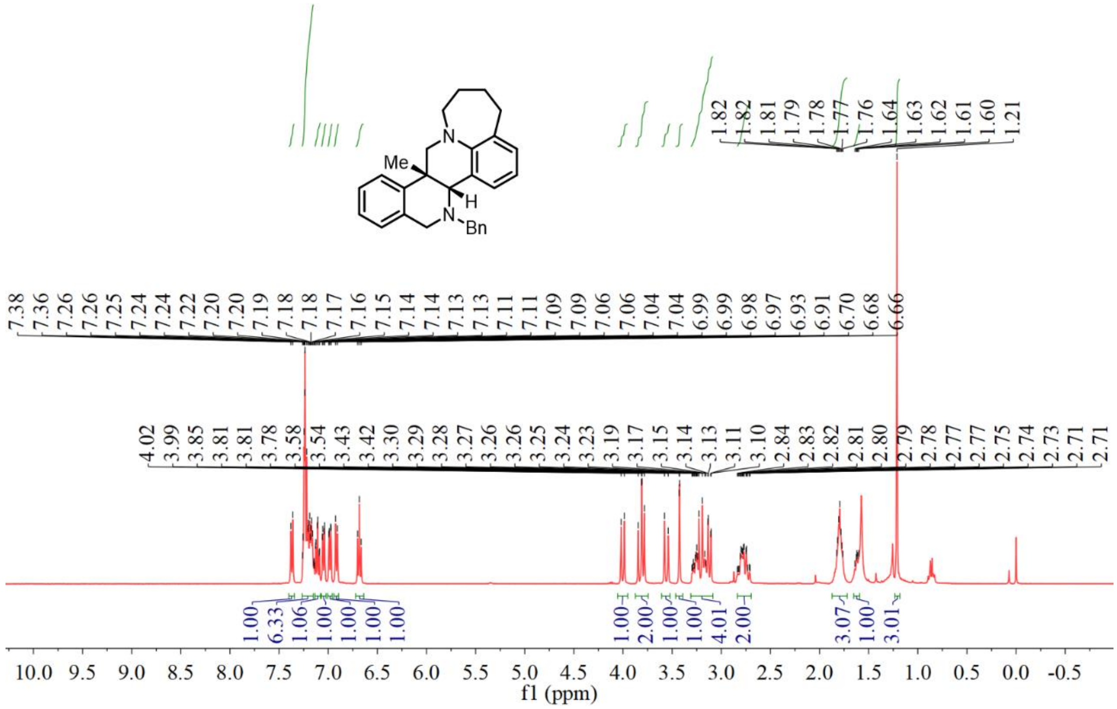

Supplementary Fig. 180 ^{1}H-NMR spectrum of P79

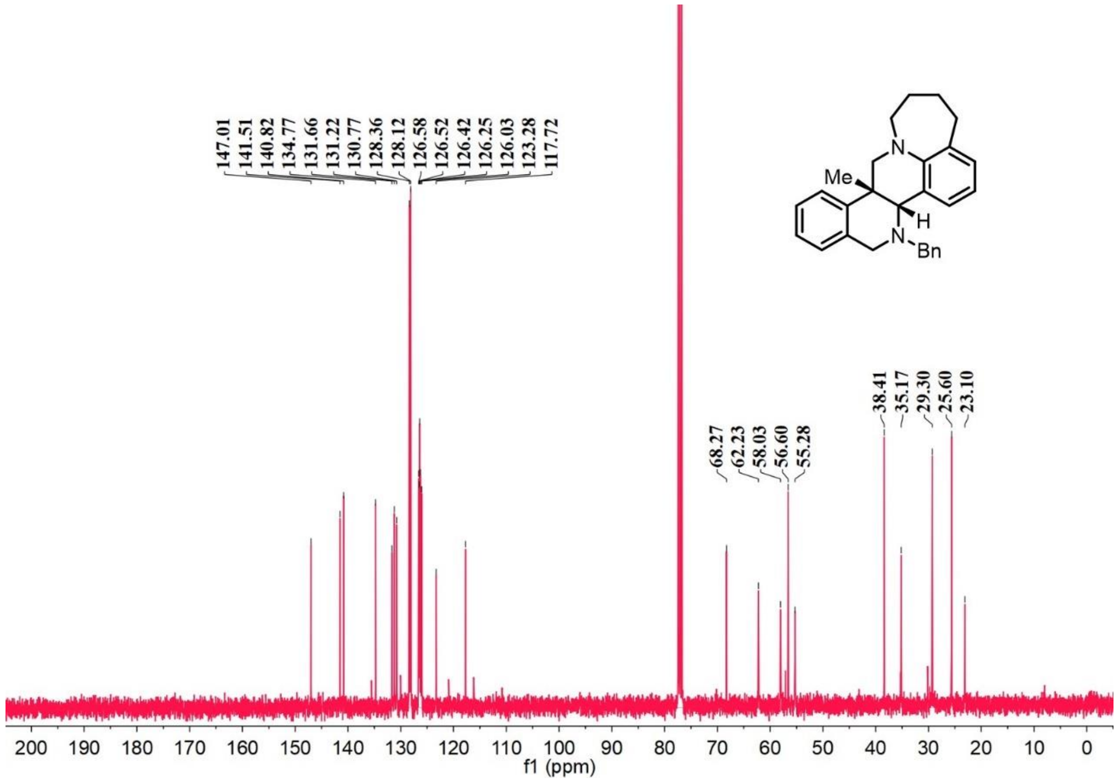

Supplementary Fig. 181 ^{13}C-NMR spectrum of P79

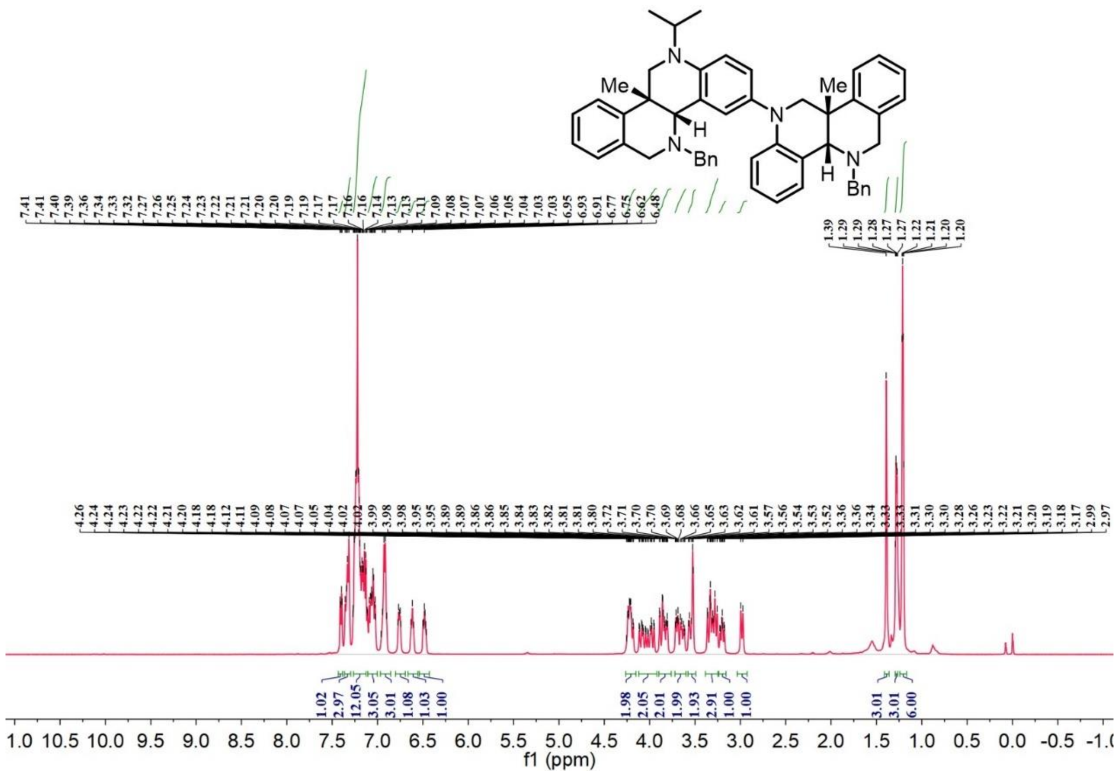

Supplementary Fig. 182 ^{1}H-NMR spectrum of P80

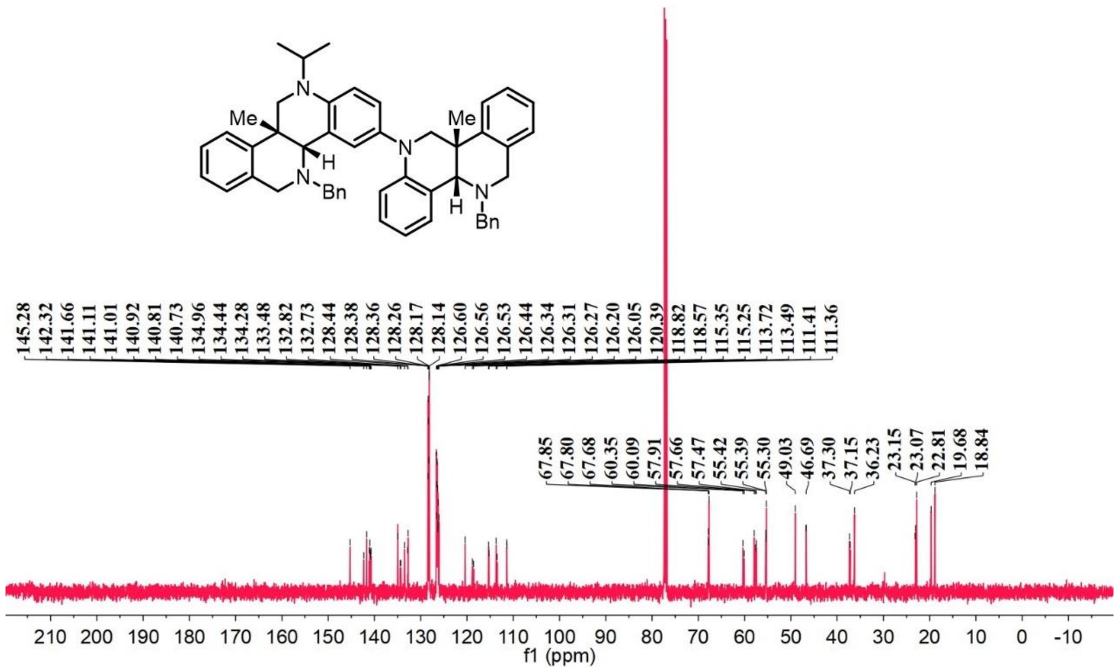

Supplementary Fig. 183 ^{13}C-NMR spectrum of P80

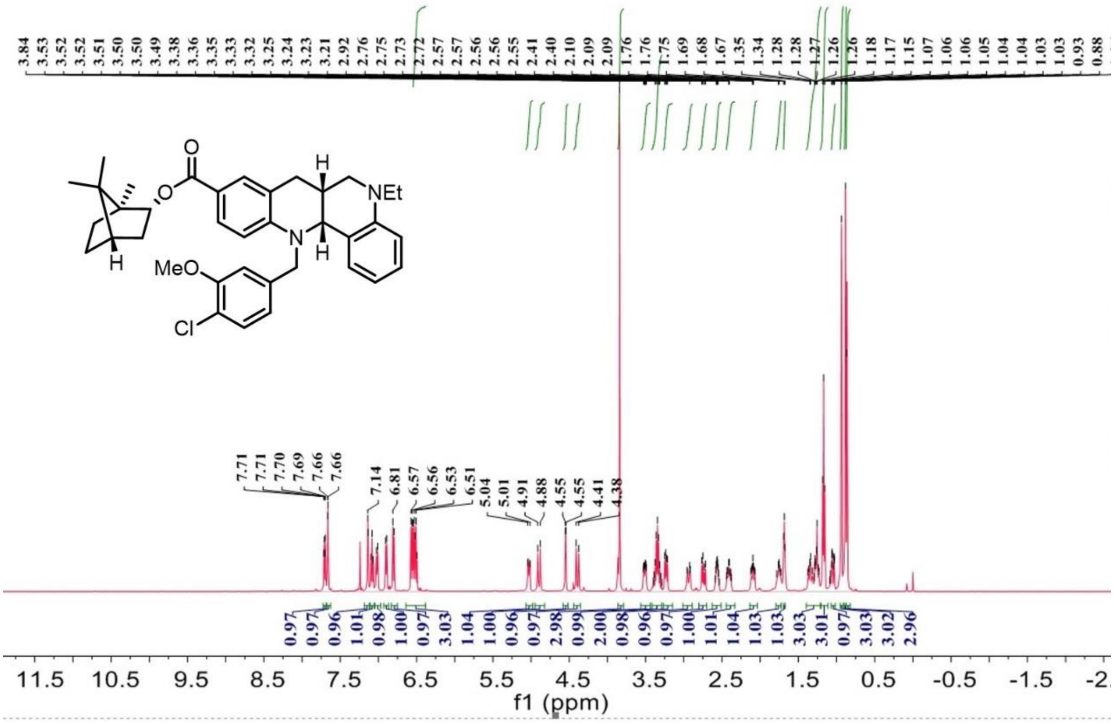

Supplementary Fig. 184 ^{1}H-NMR spectrum of P81

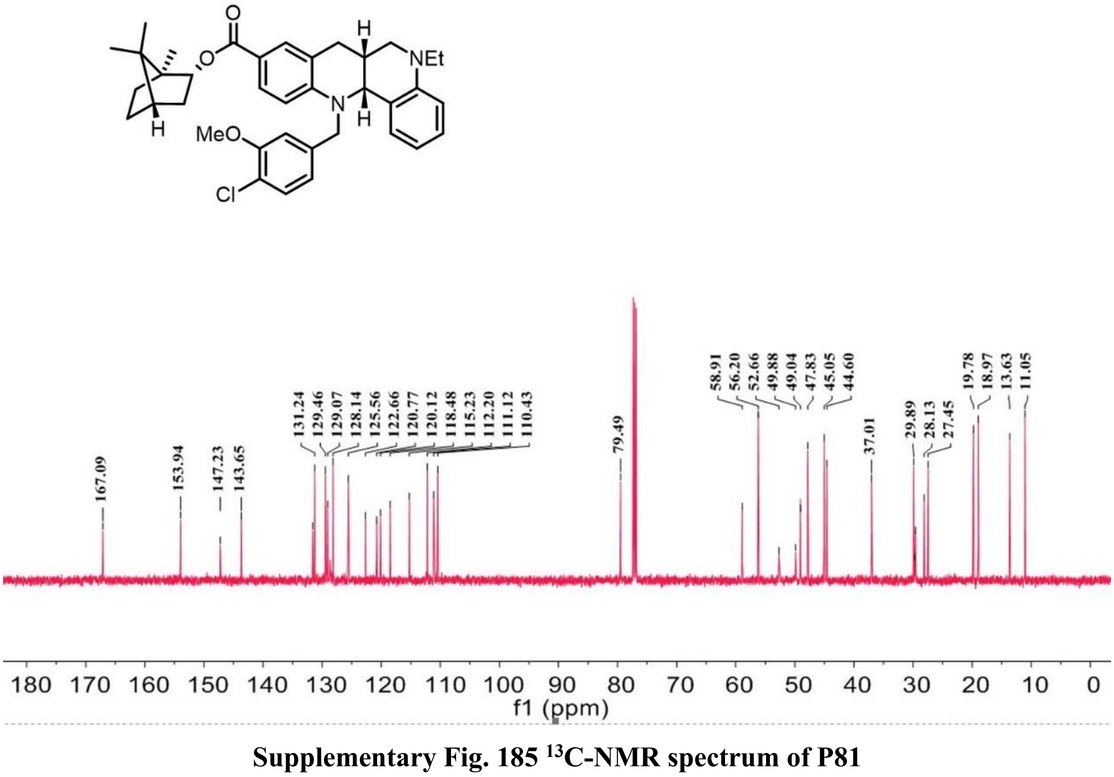

Supplementary Fig. 185 ^{13}C-NMR spectrum of P81

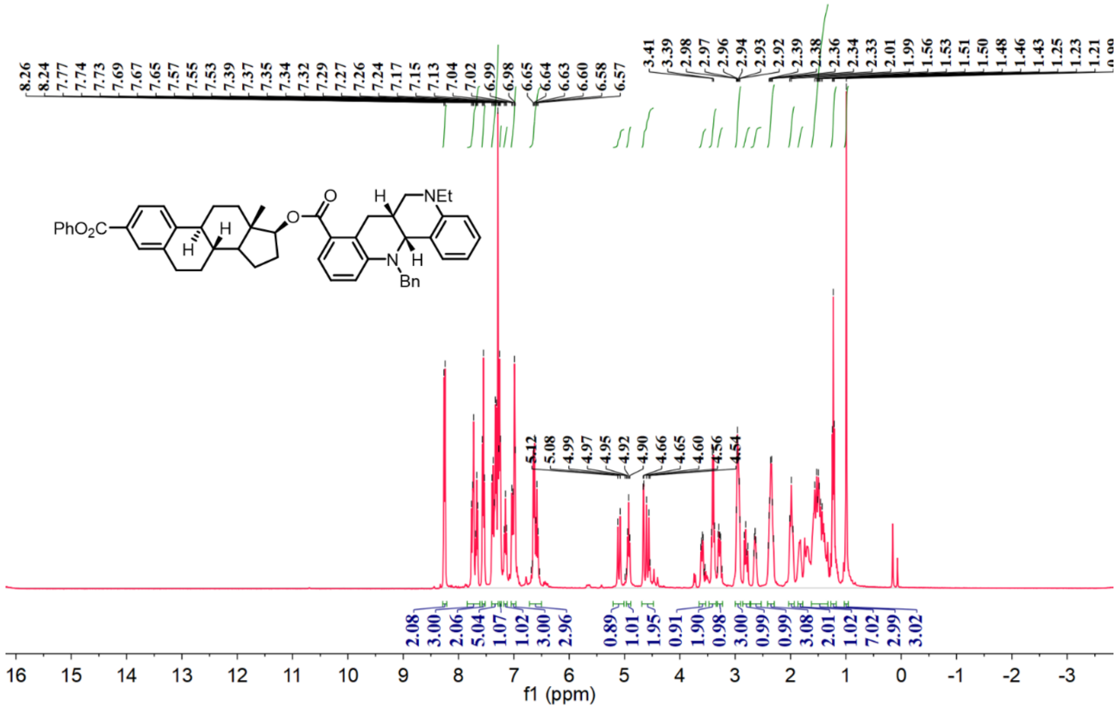

Supplementary Fig. 186 ^{1}H-NMR spectrum of P82

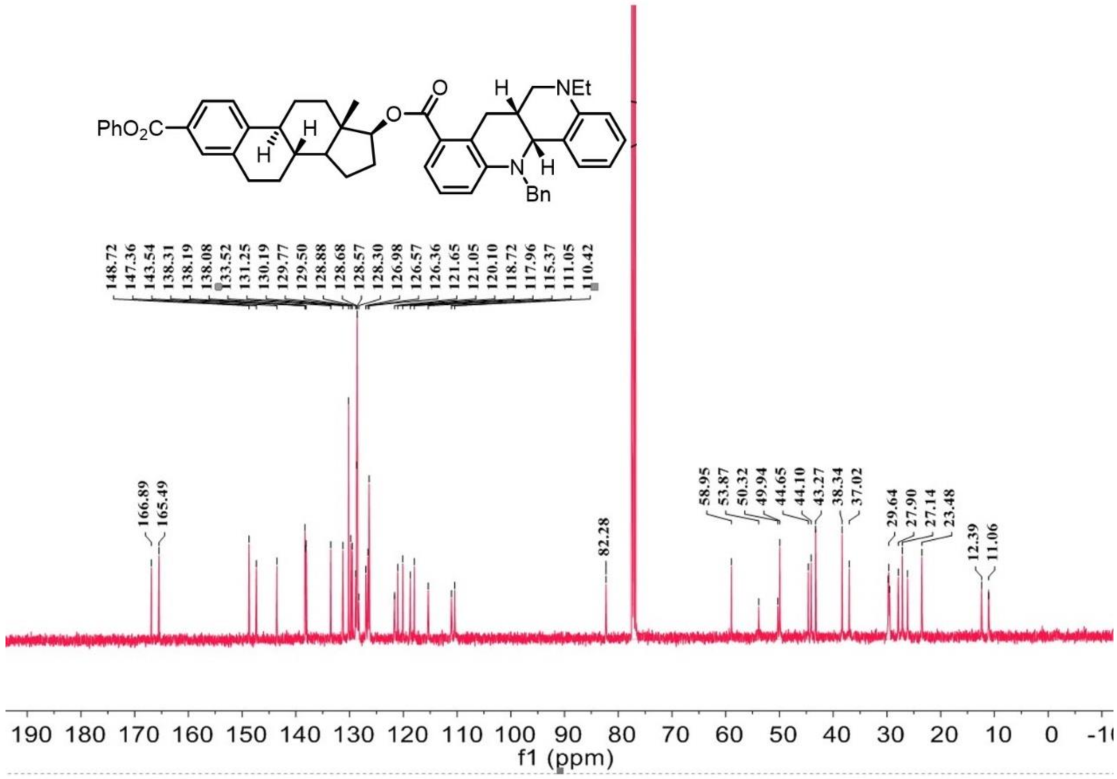

Supplementary Fig. 187 ^{13}C-NMR spectrum of P82

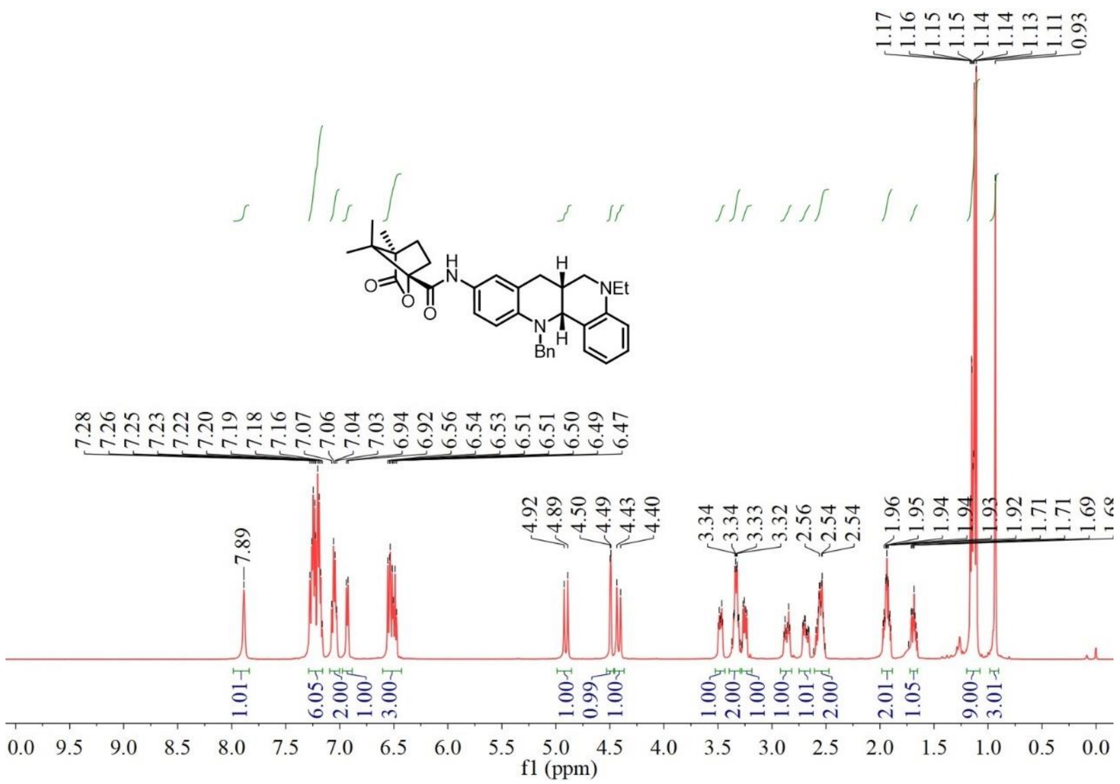

Supplementary Fig. 188 ^{1}H-NMR spectrum of P83

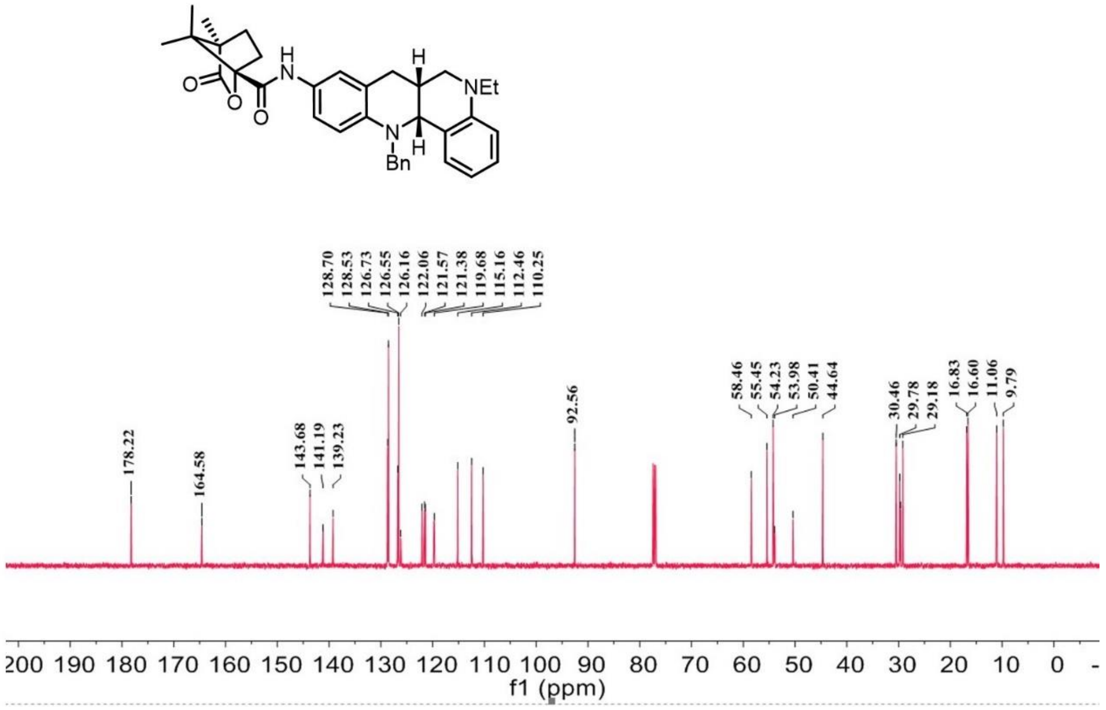

Supplementary Fig. 189 ^{13}C-NMR spectrum of P83

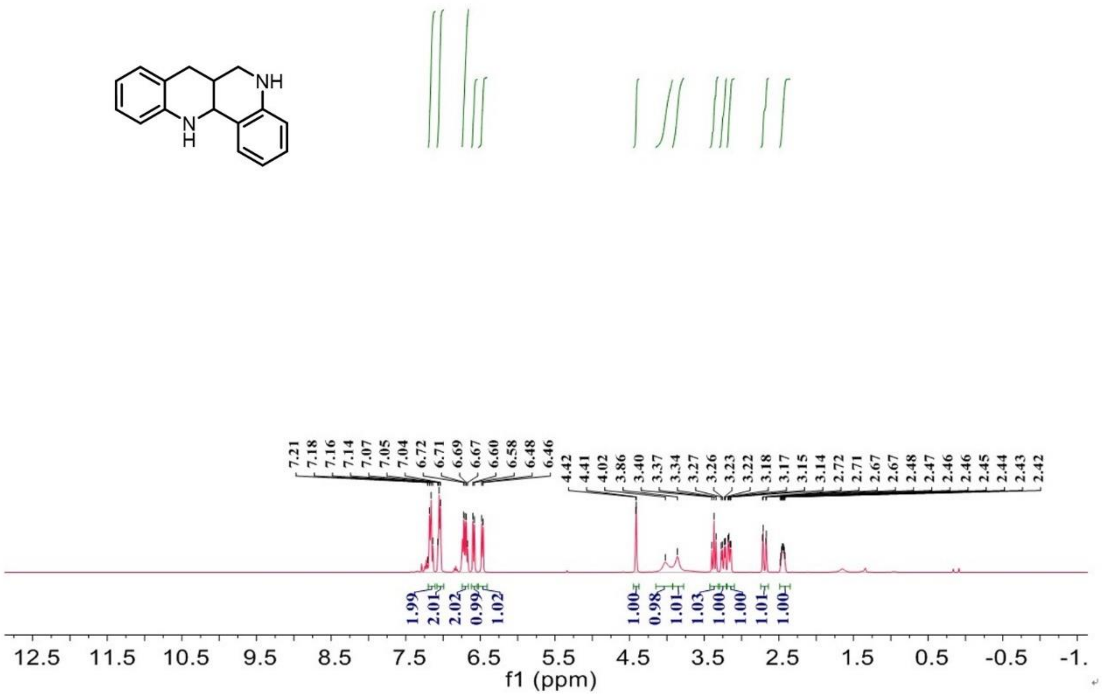

Supplementary Fig. 190 ^{1}H-NMR spectrum of P84

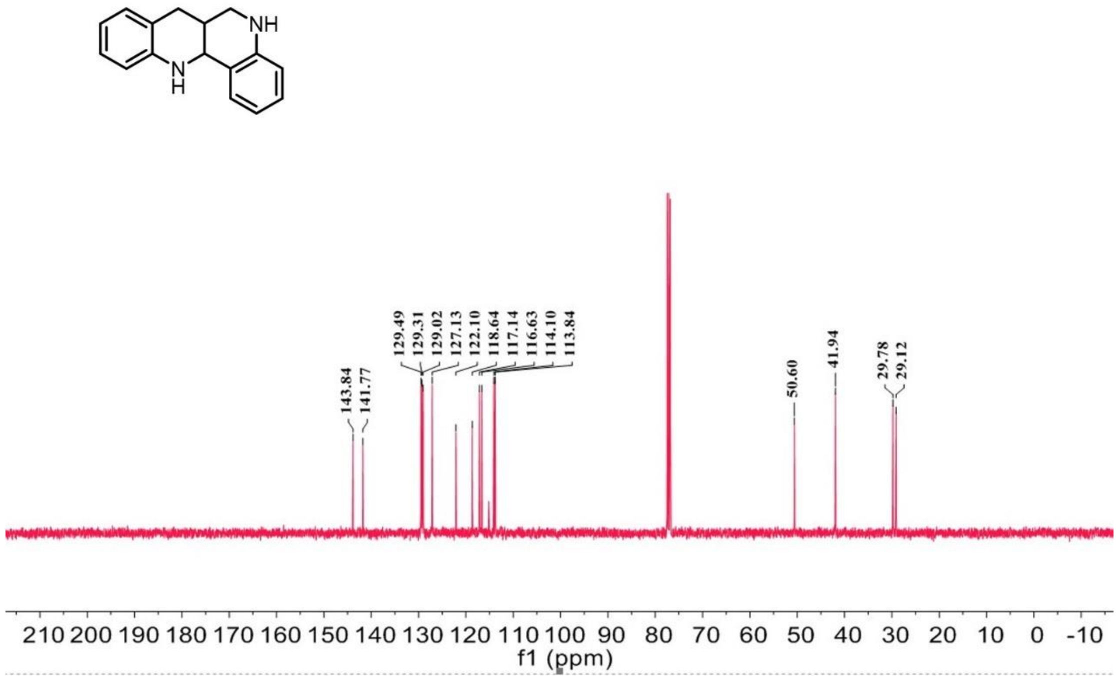

Supplementary Fig. 191 ^{13}C-NMR spectrum of P84

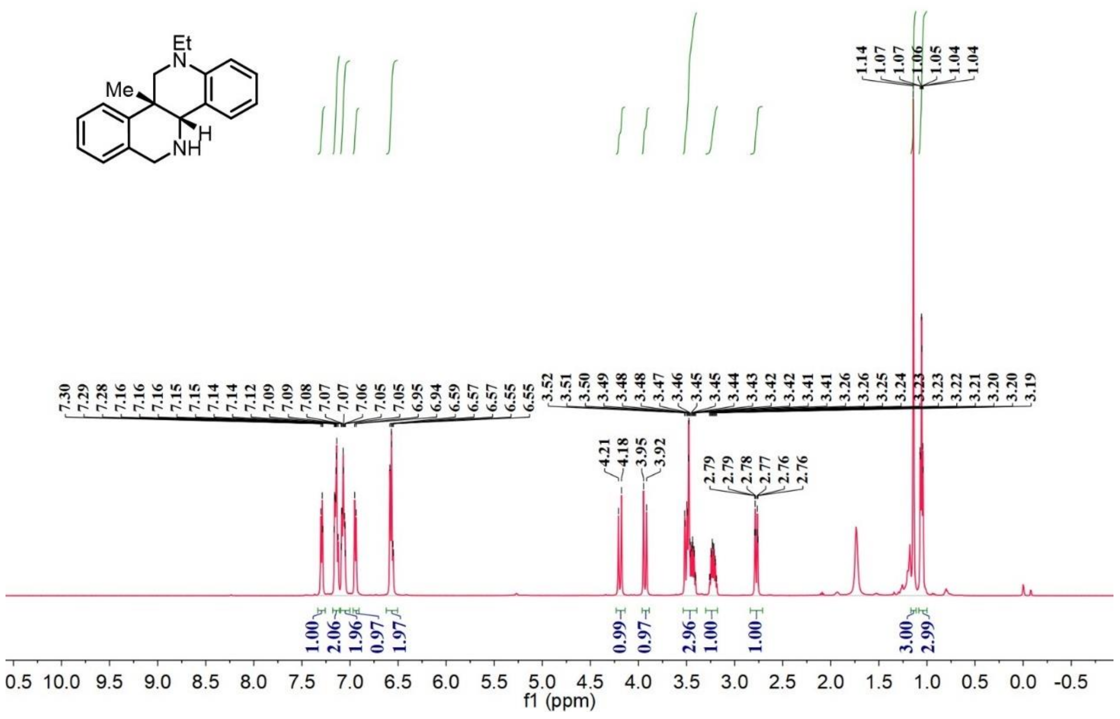

Supplementary Fig. 192 ¹H-NMR spectrum of P85

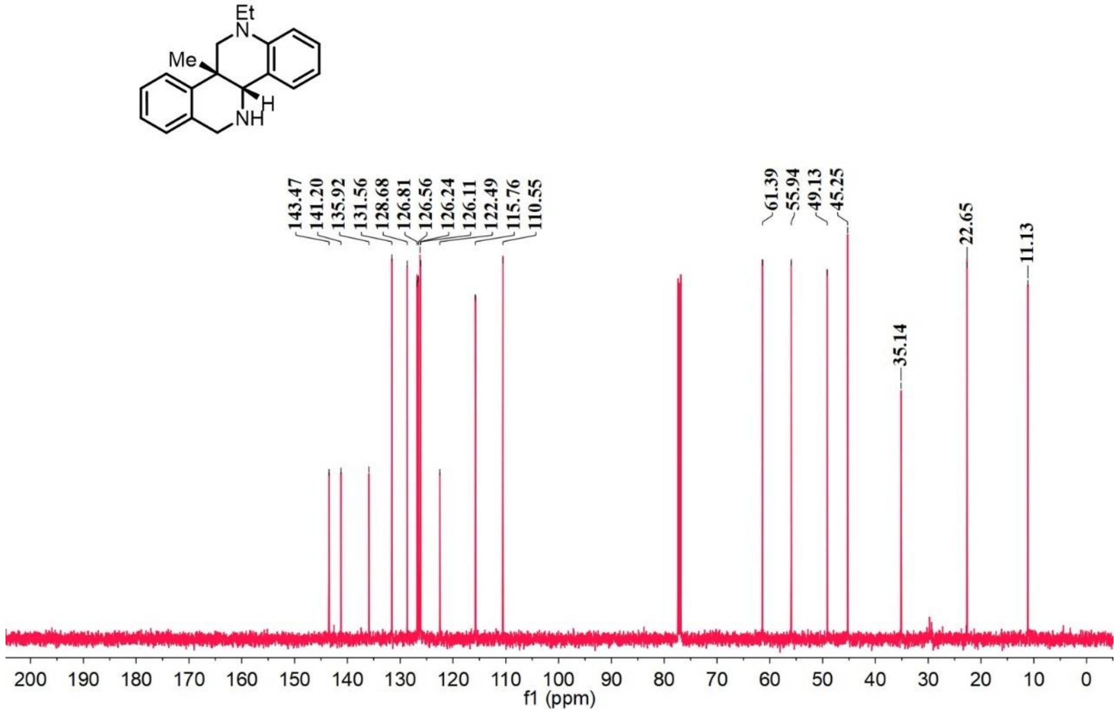

Supplementary Fig. 193 ¹³C-NMR spectrum of P85

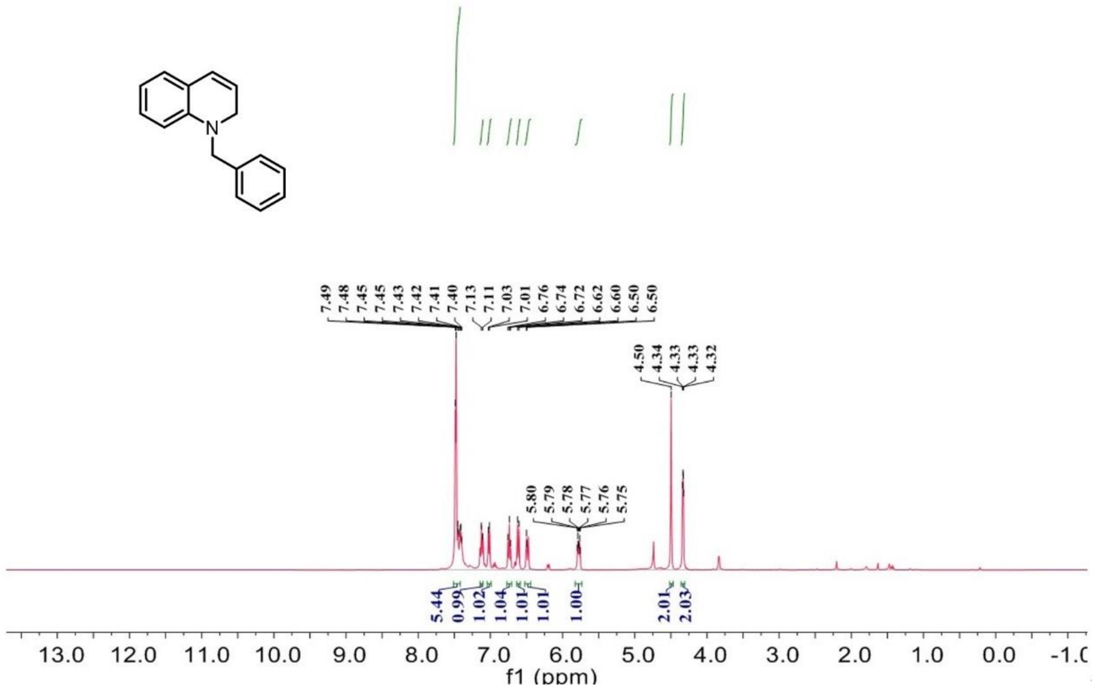

Supplementary Fig. 194 ^{1}H-NMR spectrum of int-1

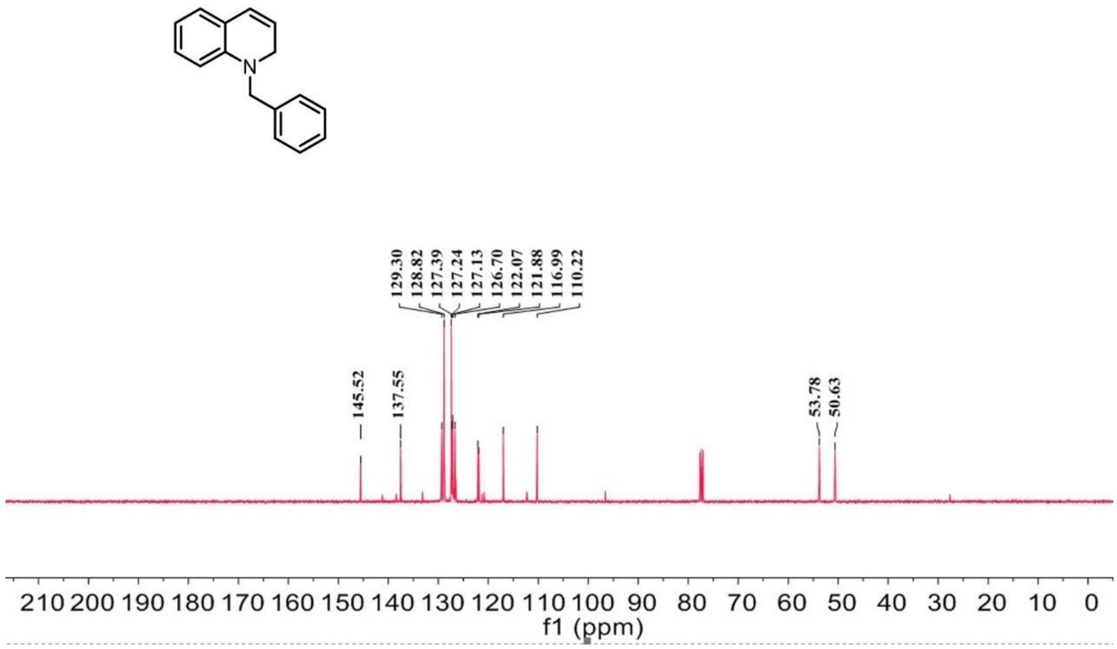

Supplementary Fig. 195 ^{13}C-NMR spectrum of int-1

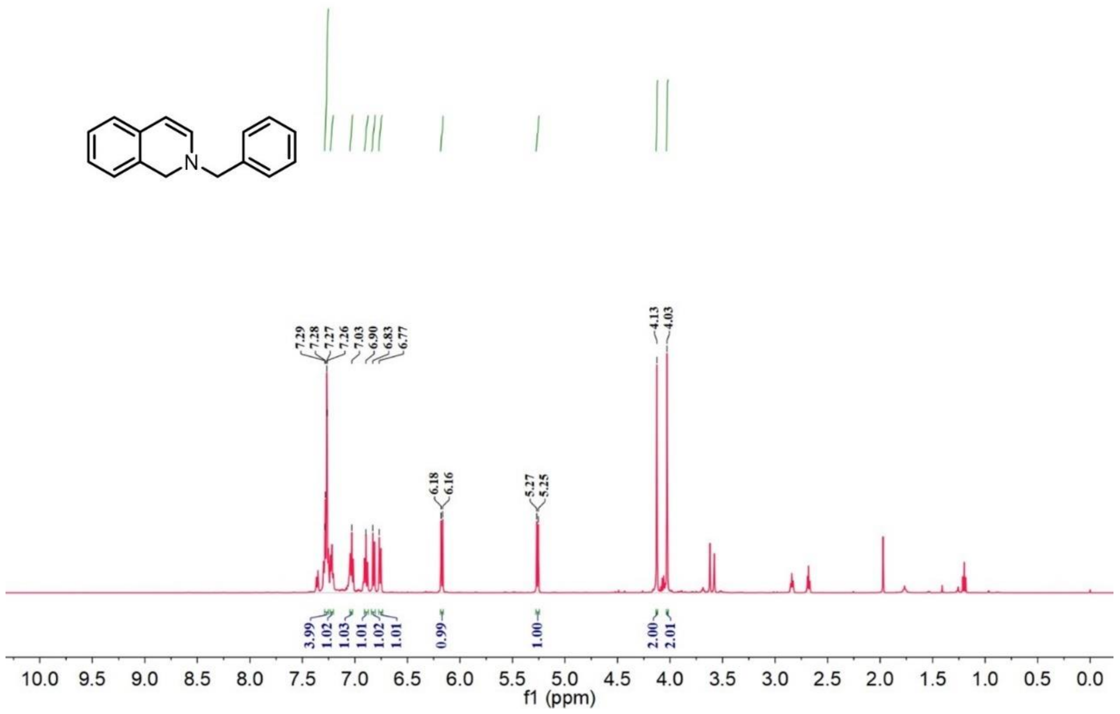

Supplementary Fig. 196 ^{1}H-NMR spectrum of int-6

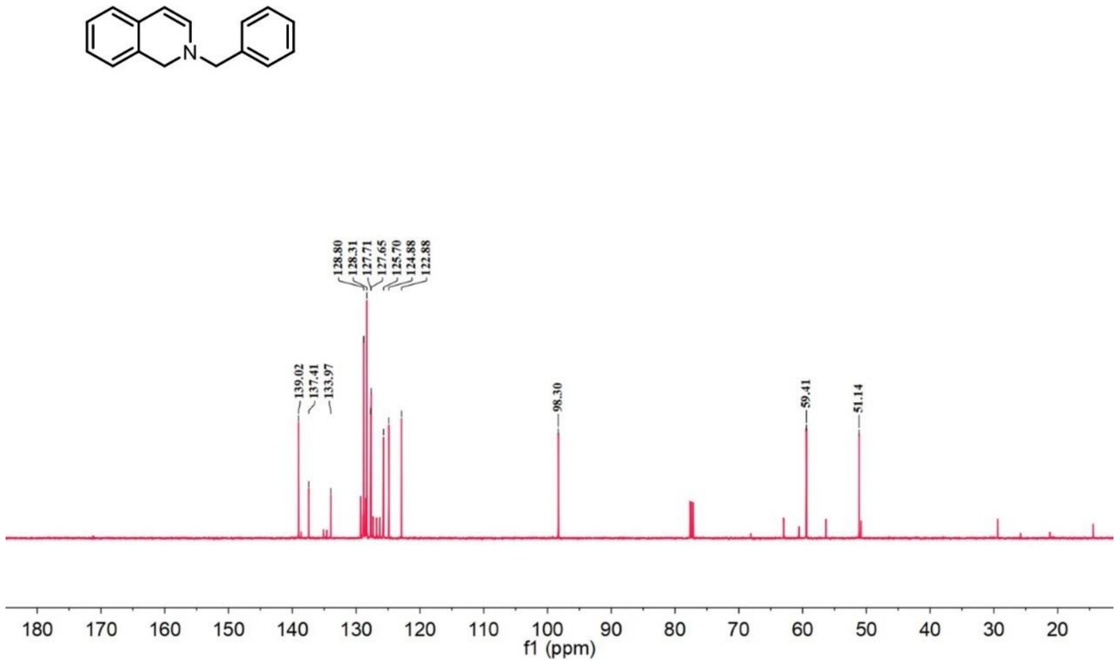

Supplementary Fig. 197 ^{13}C-NMR spectrum of int-6

Supplementary References

(1) Tan, Z. D.; Ci, C. G.; Yang, J.; Wu, Y.; Liang Cao, Jiang, H. F.; Zhang, M. Catalytic Conversion of N-Heteroaromatics to Functionalized Arylamines by Merging Hydrogen Transfer and Selective Coupling. *ACS Catal.* 10, 5243–5249 (2020).

(2) Yang, J.; Zhao, H.; Tan, Z. D.; Cao, L.; Jiang, H. F.; Ci, C. G.; Dixneuf, P. H.; Zhang, M. syn-Selective Construction of Fused Heterocycles by Catalytic Reductive Tandem Functionalization of N-Heteroarenes. *ACS Catal.* **11**, 9271–9278 (2021).

(3) Frisch, M. J., et al. Gaussian 16; Gaussian, Inc., Wallingford, CT, 2016.

(4) Lee, C.; Yang, C.; Parr, R. G. Development of the Colle-Salvetti correlation-energy formula into a functional of the electron density. *Phys. Rev. B: Condens. Matter Mater. Phys*. **37**, 785–789 (1988).

(5) Becke, A. D. Density-functional thermochemistry. III. The role of exact exchange. *J. Chem. Phys.* **98**, 5648-5652 (1993).

(6) Stephens, P. J.; Devlin, F. J.; Chabalowski, C. F.; Frisch, M. Ab Initio Calculation of Vibrational Absorption and Circular Dichroism Spectra Using Density Functional Force Fields. J. *J. Phys. Chem*. **98**, 11623–11627 (1994).

(7) Hay, P. J.; Wadt, W. R. Ab initio effective core potentials for molecular calculations. Potentials for the transition metal atoms Sc to Hg. *J. Chem. Phys.* **82**, 270–283 (1985).

(8) Francl, M. M.; Pietro, W. J.; Hehre, W. J.; Binkley, J. S.; Gordon, M. S.; Defrees, D. J.; Pople, J. A. Self-consistent molecular orbital methods. XXIII. A polarization-type basis set for second-row elements. *J. Chem. Phys*. **77**, 3654–3665 (1982).

(9) Hehre, W. J.; Ditchfield, R.; Pople, J. A. Self-Consistent Molecular Orbital Methods. XII. Further Extensions of Gaussian-Type Basis Sets for Use in Molecular Orbital Studies of Organic Molecules. *J. Chem. Phys*. **56**, 2257–2261 (1972).

(10) Hariharan, P. C.; Pople, J. A. The influence of polarization functions on molecular orbital hydrogenation energies. *Theor. Chim. Acta*. **28**, 213–222 (1973).

(11) Peng, C. Y.; Schlegel, H. B. Combining Synchronous Transit and Quasi-Newton Methods to Find Transition States. *Isr. J. Chem*. **33**, 449–454 (1993).

(12) Hratchian, H. P.; Schlegel, H. B. Accurate reaction paths using a Hessian based predictor-corrector integrator. *J. Chem. Phys.* **120**, 9918–9924 (2004).

(13) Cancès, E.; Mennucci, B.; Tomasi, J. A new integral equation formalism for the polarizable continuum model: Theoretical background and applications to isotropic and anisotropic dielectrics. *J. Chem. Phys.* **107**, 3032–3041 (1997).
