# Supplementary material for: Intermolecular diastereoselective annulation of azaarenes into fused N-heterocycles by Ru(II) reductive catalysis
Source: Nat Commun. 2022 May 2;13:2393. doi: 10.1038/s41467-022-29985-z (PMC9061824; doi:10.1038/s41467-022-29985-z)
Supplement: Supplementary file 2 — Description of Additional Supplementary Files [file 41467_2022_29985_MOESM2_ESM.pdf]

Description of Additional Supplementary files

File name: Supplementary Data 1

Description: Cartesian coordinates and absolute energies for all species
