# Supplementary material for: Intermolecular diastereoselective annulation of azaarenes into fused N-heterocycles by Ru(II) reductive catalysis
Source: Nat Commun. 2022 May 2;13:2393. doi: 10.1038/s41467-022-29985-z (PMC9061824; doi:10.1038/s41467-022-29985-z)
Supplement: Supplementary file 3 — Supplementary Dataset [file 41467_2022_29985_MOESM3_ESM.pdf]

Supplementary Information

Supplementary Data

Cartesian coordinates and absolute energies for all species

int-2

G +ZPE = -673.153856 a.u

N	-1.27767300	-0.38010100	-1.36049500
C	-0.04047000	0.14856100	-1.73654600
C	-2.49417800	0.21459100	-1.89810300
C	1.13805100	-0.33430000	-1.33354000
H	-0.10564500	1.00088900	-2.40115100
C	-1.31468800	-1.57314100	-0.62241400
C	-3.35308200	0.96810200	-0.89024500
H	-2.18702400	0.90770600	-2.68498200
H	-3.10026900	-0.55088200	-2.39409700
C	1.23947300	-1.49716300	-0.38672800
H	2.04531200	0.13997600	-1.68687700
C	-0.11122400	-2.16020100	-0.16452100
C	-2.53504100	-2.21138100	-0.33779200
C	-4.72474800	1.10987300	-1.12370800
C	-2.79681200	1.56872900	0.24176400
H	1.95499200	-2.23944300	-0.76319100
H	1.65283700	-1.16966400	0.57964300
C	-0.17612100	-3.36938100	0.52650000
C	-2.56753600	-3.41275700	0.36385600
H	-3.46813300	-1.76748800	-0.65510000
C	-5.52408200	1.84339500	-0.24883700
H	-5.17151700	0.64104800	-1.99516900
C	-3.59511200	2.29975000	1.12071800
H	-1.73665800	1.45908000	0.43898500
C	-1.38597900	-4.00606900	0.79779400
H	0.75264000	-3.81808300	0.86625100
H	-3.52438300	-3.88002800	0.56849200
C	-4.96066000	2.44143200	0.87790800
H	-6.58626100	1.94196500	-0.44348900
H	-3.14932400	2.75813100	1.99659500
H	-1.40312600	-4.94291100	1.34220500
H	-5.58111400	3.00843800	1.56261300

B1'

G +ZPE = -404.505178 a.u

C	3.02902400	-1.37459600	0.90282300
C	0.72155100	2.19306900	0.47748400
C	2.78403400	0.96602700	0.54804400
N	3.49308600	-0.29012900	0.40370000
H	3.54719300	-2.30792200	0.72253900
H	2.12892300	-1.35484000	1.50242500
C	1.40629800	3.34989500	0.84350200
H	-0.34551600	2.22263400	0.29607800
C	3.48595900	2.11937400	0.89810700
C	4.78265000	-0.31268600	-0.37463000
C	2.78480800	3.31079300	1.05094800
H	0.86874100	4.28311700	0.95878900
H	4.55265600	2.09330900	1.07343800
C	6.00740600	-0.42437600	0.52733100
H	4.79580700	0.59547300	-0.97133200
H	4.71223900	-1.16385800	-1.05071500
H	3.31959900	4.20804900	1.33617900
H	6.89484800	-0.45200600	-0.10753700
H	6.09935700	0.42562300	1.20408400
H	5.98353500	-1.34169600	1.11824800
C	1.40766400	0.99208300	0.32459300
H	0.88519500	0.09948800	0.00406900

int-3a

G +ZPE = -1077.649035 a.u

N	-0.22118100	1.52966300	11.87934900
C	0.52991300	1.95017900	13.04118600
C	-1.15941100	0.36944300	11.98385900
C	1.13146000	3.21795600	13.01561600
C	0.66124200	1.10536300	14.14398600
H	-0.56692500	-0.50778000	12.24298400
H	-1.54745800	0.21001000	10.97824900
C	-2.28664900	0.57809400	12.97281900
C	1.85466700	3.62402800	14.13499100
C	1.39461300	1.53203000	15.24563900
H	0.21119800	0.12433700	14.15761400
C	-2.65189700	-0.47282900	13.81843900
C	-3.01307700	1.77158200	13.01267200
H	2.32464300	4.60065500	14.12937700
C	1.98611400	2.79285500	15.24483800
H	1.50061300	0.87573300	16.10015000
H	-2.09560500	-1.40378700	13.79498300
C	-3.72964400	-0.33491500	14.69108800

C	-4.08310300	1.91253100	13.89299700
H	-2.75181300	2.59529200	12.35878100
H	2.55560900	3.12640300	16.10388000
H	-4.00358100	-1.15799400	15.34063600
C	-4.44520700	0.85984100	14.73278600
H	-4.63624200	2.84415100	13.91914800
H	-5.27937500	0.97108500	15.41567800
C	0.90759200	3.22830400	10.53411300
C	0.97095400	4.09115900	11.79954700
H	0.05624600	4.68809300	11.87359400
H	1.80245700	4.79286400	11.72318000
C	0.70022500	4.02741000	9.22907500
H	0.65663100	3.31956400	8.38747400
H	1.60579100	4.61858800	9.08414500
N	-0.42723800	4.95699300	9.27299700
C	-0.24521300	6.11225200	8.36379400
H	0.77644600	6.46993100	8.51243400
H	-0.32702600	5.80351200	7.30893800
C	-1.21558800	7.25625400	8.63911200
H	-2.25235200	6.96586300	8.45741300
H	-1.13067600	7.59704300	9.67408400
H	-0.98376300	8.09653100	7.97955100
C	-1.72434700	4.33344800	9.15208900
C	-2.69976900	4.60778900	10.11572700
C	-2.05049300	3.49167200	8.07896200
C	-3.97520700	4.05213700	10.01329300
H	-2.44945900	5.26659500	10.93880600
C	-3.32135600	2.92860300	7.98340400
H	-1.31521200	3.28072900	7.31062800
C	-4.28773600	3.20751000	8.95004800
H	-4.72189600	4.27808400	10.76589300
H	-3.55944700	2.27891000	7.14876000
H	-5.27696300	2.77154200	8.87239500
H	1.87848800	2.71732000	10.40935000
C	-0.05563300	2.11171800	10.73206000
H	-0.62332400	1.71375400	9.89924900

int-3b

G +ZPE = -1077.650149 a.u

N	-3.09934800	0.99066400	12.45182500
C	-2.57999300	-0.27107900	11.97734500
C	-4.04539800	1.01675900	13.61128600
C	-1.48336200	-0.24622200	11.10283600

C	-3.17527700	-1.47336200	12.36202500
H	-4.91836400	0.42680800	13.33480100
H	-4.37280200	2.05178700	13.70456500
C	-3.43910800	0.52160200	14.90739100
C	-0.99252200	-1.46094100	10.62940100
C	-2.66887900	-2.67189000	11.87189000
H	-4.02508400	-1.49342000	13.02688800
C	-4.17210700	-0.35891700	15.70754300
C	-2.19615200	0.97860600	15.35598400
H	-0.14580900	-1.45637100	9.95286500
C	-1.57597500	-2.66727100	11.00873000
H	-3.13279300	-3.60474300	12.16628600
H	-5.13982100	-0.71586600	15.37137700
C	-3.67139800	-0.77836000	16.93854200
C	-1.69158900	0.55089400	16.58143100
H	-1.61353800	1.66794400	14.75593800
H	-1.18077000	-3.60081400	10.62694700
H	-4.25053900	-1.46033700	17.54994800
C	-2.42799100	-0.32733600	17.37591700
H	-0.72494900	0.90769600	16.91713900
H	-2.03403000	-0.65751600	18.32999700
C	-1.90981100	2.18104200	10.66743700
C	-0.85613700	1.06994700	10.72751300
H	-0.09466200	1.34305200	11.46689400
H	-0.34800400	0.98264600	9.76780700
C	-1.33174200	3.61309100	10.42126800
H	-2.10687200	4.34282600	10.67897400
H	-1.17045500	3.72317000	9.35201500
N	-0.11127200	3.92672000	11.13676800
C	-0.28154100	4.44670800	12.49482100
H	-1.11851000	3.91144600	12.95182000
H	0.59201500	4.17842500	13.09119500
C	-0.54770000	5.95506300	12.57317900
H	0.28544400	6.52765500	12.16093100
H	-1.45110800	6.22640100	12.02115700
H	-0.68722900	6.25610300	13.61483600
C	1.12488300	4.00464100	10.48775800
C	1.32505000	3.47705300	9.19263700
C	2.24272700	4.59801100	11.11580300
C	2.56723200	3.54676700	8.56977400
H	0.51873600	2.99338200	8.65949100
C	3.48083600	4.65132400	10.48298200
H	2.15346200	5.03243900	12.10091200
C	3.66177400	4.13131000	9.20339400

H	2.67453800	3.12706500	7.57533500
H	4.31098900	5.11736300	11.00292000
H	4.62711700	4.17953400	8.71430400
H	-2.58911200	1.99599500	9.81759500
C	-2.80402500	2.10223900	11.85253200
H	-3.29172100	2.99420800	12.22941700

TS4

G +ZPE = -1077.636644 a.u

N	0.25605600	1.33298800	12.39511800
C	-0.21776400	2.36581400	13.25101700
C	0.11263900	-0.08545100	12.76686600
C	0.08665300	3.70957800	12.95279200
C	-0.98913800	2.05992600	14.38003200
H	0.44140400	-0.20338200	13.80089500
H	0.82589300	-0.63708600	12.15200500
C	-1.27649300	-0.68098700	12.59331400
C	-0.39993000	4.70664800	13.79782600
C	-1.45320600	3.07420400	15.21089100
H	-1.24438900	1.03624400	14.61030000
C	-1.77849100	-1.54502600	13.57089000
C	-2.04160800	-0.44207500	11.44841900
H	-0.16564400	5.74041500	13.56724800
C	-1.16313600	4.40471400	14.92225600
H	-2.04784200	2.81738500	16.07948800
H	-1.19494100	-1.74156500	14.46456200
C	-3.02128600	-2.15614400	13.41079700
C	-3.28648100	-1.04677900	11.28835300
H	-1.66793300	0.21369400	10.67069700
H	-1.52639500	5.19882000	15.56344600
H	-3.39643100	-2.82173500	14.17975400
C	-3.78037000	-1.90602200	12.26945300
H	-3.86860300	-0.84961400	10.39539900
H	-4.74913600	-2.37580200	12.14471300
C	1.07657700	2.93764300	10.79692800
C	0.97079200	4.07374000	11.77688300
H	0.59262800	4.97986800	11.29421600
H	1.97457400	4.33723900	12.14045000
C	-0.85410000	3.16174500	9.47823000
H	-1.44505300	2.76444900	10.28596600
H	-0.45503100	2.48501600	8.73828500
N	-1.04134600	4.42179000	9.12228300
C	-0.43474100	4.91987100	7.85297200

H	-0.54534800	4.11571100	7.12415900
H	-1.05442600	5.74971400	7.51813400
C	1.03032400	5.33902400	7.95432600
H	1.18667200	6.13832600	8.67987100
H	1.67294300	4.49862900	8.21900400
H	1.35029900	5.70506400	6.97615000
C	-1.88951100	5.30298100	9.88287700
C	-1.57654200	6.66149800	9.98999500
C	-3.05038100	4.80657500	10.48419300
C	-2.41037100	7.50825200	10.71419000
H	-0.68363900	7.06119600	9.52890000
C	-3.87267500	5.66112300	11.21244200
H	-3.33394300	3.76916000	10.36278200
C	-3.55638800	7.01255400	11.33337300
H	-2.15591800	8.55798500	10.79717500
H	-4.77175900	5.26748200	11.67102500
H	-4.20195400	7.67541600	11.89662500
H	1.71859500	3.05554700	9.93482500
C	0.86252900	1.65321900	11.22710000
H	1.14080400	0.80931700	10.60775000

TS4'

G +ZPE = -1077.635934 a.u

N	-2.96709400	0.91174600	11.47861200
C	-4.08973600	1.58968400	10.93814400
C	-2.97764100	-0.54820000	11.66527600
C	-4.11665400	3.00027400	10.92771000
C	-5.17311600	0.87221800	10.41098900
H	-3.91901400	-0.82841800	12.14183900
H	-2.19003900	-0.77183400	12.38709500
C	-2.76577600	-1.38056600	10.40958500
C	-5.23102800	3.64299700	10.38696300
C	-6.27401600	1.53956700	9.88529600
H	-5.15984700	-0.20742800	10.39500700
C	-3.54841800	-2.51875300	10.19518700
C	-1.75897900	-1.07330200	9.48995500
H	-5.25034100	4.72794600	10.38231700
C	-6.30879600	2.93113400	9.86846500
H	-7.10004600	0.96449500	9.48385700
H	-4.33114700	-2.77347700	10.90260400
C	-3.33473300	-3.33085600	9.08204600
C	-1.54640000	-1.87946100	8.37395100
H	-1.12773500	-0.20645100	9.64620600

H	-7.16223600	3.45629400	9.45669200
H	-3.95254700	-4.20851100	8.92987800
C	-2.33484000	-3.01096900	8.16601000
H	-0.76233900	-1.62632700	7.66942400
H	-2.16990800	-3.63778500	7.29725900
C	-1.78120600	2.98366500	11.81412200
C	-2.99428600	3.82161400	11.53303100
H	-2.75007800	4.66313400	10.87219000
H	-3.34123700	4.29050600	12.46489300
C	-0.48881000	3.15639900	9.68910800
H	-0.93590900	2.24806500	9.32082300
H	0.42391500	3.10051900	10.25938300
N	-0.84008800	4.28454400	9.11619500
C	-1.92783500	4.23678000	8.09401000
H	-2.65960400	3.51247800	8.45184600
H	-2.41531500	5.20886900	8.08470900
C	-1.41738400	3.84351100	6.70936900
H	-0.67758900	4.54914300	6.32869200
H	-0.96864300	2.84828900	6.72498200
H	-2.26104200	3.82459400	6.01628800
C	-0.19725300	5.53560300	9.42312200
C	-0.25492000	6.59319100	8.50904600
C	0.49477200	5.69961400	10.62850200
C	0.38364800	7.79536700	8.79927600
H	-0.77542200	6.49172700	7.56791800
C	1.12949900	6.90565300	10.90565100
H	0.53094200	4.90937400	11.36469300
C	1.07903800	7.95855700	9.99451900
H	0.33644600	8.60367600	8.07972900
H	1.65571100	7.02075500	11.84544700
H	1.57252300	8.89688100	10.21628100
H	-0.94039600	3.43808800	12.31996000
C	-1.87647300	1.62414600	11.87235300
H	-1.04916000	1.02885700	12.23835900

TS5

G +ZPE = -1077.635188 a.u

N	0.89982000	0.71898600	-1.11909900
C	1.65225100	1.63556800	-0.29004400
C	1.61276400	-0.35810100	-1.87381900
C	0.95355000	2.38926300	0.66652800
C	3.03156600	1.76733800	-0.45147900
H	2.29265700	0.12876500	-2.57244200

H	0.84794300	-0.85957700	-2.46622600
C	2.34798600	-1.34217300	-0.98982600
C	1.67804700	3.26530800	1.47047900
C	3.73088700	2.65576600	0.35853500
H	3.56747900	1.19879700	-1.19615300
C	3.64156200	-1.73946000	-1.33835500
C	1.73440400	-1.91273900	0.12871300
H	1.15152000	3.85252200	2.21378900
C	3.05696900	3.39977300	1.32325900
H	4.80076200	2.76162500	0.23117100
H	4.12508400	-1.30550500	-2.20716400
C	4.31445000	-2.69497000	-0.57906900
C	2.41147300	-2.85991200	0.89277900
H	0.72684000	-1.62771600	0.40797900
H	3.60167400	4.08993500	1.95601800
H	5.31686000	-2.99607600	-0.86002400
C	3.70202900	-3.25398900	0.54066500
H	1.92744900	-3.29290700	1.76042000
H	4.22648300	-3.99248600	1.13578600
C	-1.13506100	1.94904800	-0.59342800
C	-0.53920700	2.21777100	0.79785400
H	-0.75288100	1.39367500	1.48095400
H	-0.99020300	3.11767000	1.21723500
C	-2.66796000	1.86994300	-0.74353400
H	-2.86170900	2.01728400	-1.81229700
H	-3.06991700	2.75684800	-0.23913300
N	-3.41949900	0.66924200	-0.32116300
C	-4.56257800	0.39697400	-1.20808300
H	-4.95554600	1.36984900	-1.51289300
H	-4.25509200	-0.11259800	-2.13604700
C	-5.68651400	-0.37922300	-0.52624300
H	-5.37216700	-1.38035700	-0.22502500
H	-6.03321400	0.15243700	0.36355600
H	-6.52883400	-0.48628500	-1.21462700
C	-2.79636700	-0.44905200	0.29985400
C	-2.47602500	-0.39442400	1.66587000
C	-2.58013500	-1.65834700	-0.38223300
C	-1.92666100	-1.49445600	2.31842100
H	-2.70041800	0.51036400	2.21846100
C	-2.05366700	-2.76721200	0.27940300
H	-2.84345800	-1.74100300	-1.43032900
C	-1.71676400	-2.68975800	1.62927000
H	-1.69074700	-1.42718600	3.37456000
H	-1.90372200	-3.69238900	-0.26575800

H	-1.30858600	-3.55277000	2.14205700
H	-0.87244100	2.83447500	-1.20041600
C	-0.38257400	0.84951200	-1.25574600
H	-0.87460600	0.15033400	-1.92024200

TS6

G +ZPE = -1077.624254 a.u

N	-0.81454500	-0.69134100	-0.83133600
C	0.43736100	-0.19628900	-1.13564700
C	-2.00681400	-0.00801200	-1.36173900
C	1.61564600	-1.16274400	-1.09205200
H	0.42318500	0.46497000	-1.99283700
C	-0.98132500	-1.98007400	-0.23198600
C	-2.82107600	0.87872700	-0.42507300
H	-1.68253600	0.59265800	-2.21440900
H	-2.66419600	-0.78398500	-1.76472900
C	1.13237200	-2.58890000	-1.38900000
C	2.42257400	-1.11346300	0.23823400
H	2.30878600	-0.85370800	-1.87638400
C	0.29770300	2.41831400	-0.05748200
C	2.48457600	1.27101600	0.12505100
C	-0.00593700	-2.96042200	-0.48109100
C	-2.11982200	-2.30661200	0.51064000
C	-3.83997600	1.64770200	-1.00141800
C	-2.62620400	0.95475400	0.95523700
H	0.79572300	-2.63681500	-2.43151300
H	1.96080600	-3.29320200	-1.29212300
N	3.17525800	0.13162700	0.30334000
H	3.12851800	-1.93999900	0.26468900
H	1.76133900	-1.22331000	1.10173500
C	0.92101200	3.57718700	-0.41075400
H	-0.77800200	2.38672900	0.04308000
C	3.10771600	2.51512900	-0.16964900
C	-0.15977400	-4.23106200	0.06496900
C	-2.26813600	-3.59118600	1.03154300
H	-2.89252500	-1.57203700	0.68593800
C	-4.64437400	2.46882900	-0.21739300
H	-4.00333600	1.60317700	-2.07362400
C	-3.43105700	1.77960400	1.74385800
H	-1.84186500	0.37590400	1.42749600
C	4.64965600	0.07608700	0.29234800
C	2.34077700	3.61793200	-0.44769300
H	0.35085000	4.47308200	-0.62082200

H	4.18417100	2.60000900	-0.18382400
C	-1.28493400	-4.55320900	0.82420300
H	0.60061800	-4.98095000	-0.12537500
H	-3.15491400	-3.83002600	1.60668100
C	-4.44317700	2.53696700	1.16195800
H	-5.42628200	3.05795900	-0.68305800
H	-3.26135800	1.82601600	2.81351900
C	5.26176800	0.37083400	1.66212500
H	5.02907800	0.76673300	-0.46201700
H	4.92752100	-0.92478100	-0.03705100
H	2.83658100	4.54959900	-0.69599600
H	-1.39454500	-5.54819400	1.23894900
H	-5.06824900	3.17663500	1.77415000
H	6.35025400	0.30188700	1.59774300
H	5.00410600	1.37480300	2.00662900
H	4.91550000	-0.34870900	2.40709000
C	1.03159800	1.20083200	0.17865800
H	0.64486600	0.57192300	0.97495300

int-4

G +ZPE = -1077.631733 a.u

N	2.03144700	4.78253400	11.58300400
C	2.50422900	3.89047900	12.65469300
C	0.58105500	4.99602600	11.55055400
C	4.02335600	3.73635300	12.63573500
H	2.21544500	4.30335100	13.62927600
C	2.85913500	5.80047400	11.07100900
C	-0.18059800	4.28602300	10.43713700
H	0.16029900	4.69126200	12.51345900
H	0.38418700	6.07035600	11.48592900
C	4.69984800	5.10713500	12.64940500
C	4.44735400	2.89742500	11.43382100
H	4.30681500	3.18794500	13.53932000
C	0.88047400	2.14613300	13.64124300
C	2.73559600	1.32086600	12.13923000
C	4.17901500	5.98220100	11.53723100
C	2.39179900	6.64570600	10.04525500
C	-1.57746500	4.21259100	10.51407500
C	0.46151200	3.72796400	9.32942700
H	4.50500600	5.57465600	13.62137400
H	5.78457700	4.99678000	12.57222300
N	3.87396500	1.53869500	11.51642300
H	5.53019300	2.78141700	11.41976200

H	4.14720200	3.36019500	10.48985600
C	0.56363800	0.87813800	13.95311800
H	0.38808700	2.97848000	14.12979200
C	2.24041200	-0.00413400	12.38752000
C	4.97832400	6.97713100	10.97471800
C	3.20152400	7.64407800	9.51378300
H	1.39725300	6.51223300	9.64125100
C	-2.31383400	3.59894600	9.50526300
H	-2.09040200	4.63740000	11.37146400
C	-0.27600700	3.11000600	8.31723700
H	1.54137900	3.77784700	9.25817500
C	4.65293500	0.45682400	10.85983800
C	1.21651600	-0.20118100	13.26230500
H	-0.17702700	0.65456800	14.70979000
H	2.73035300	-0.85946200	11.94911900
C	4.50467700	7.81978700	9.97272700
H	5.99249800	7.09393600	11.34405100
H	2.80844400	8.27719700	8.72608400
C	-1.66404800	3.04433300	8.40089000
H	-3.39422100	3.54883700	9.58156100
H	0.23828300	2.68256700	7.46376600
C	4.14007800	0.13802900	9.45630800
H	4.64095800	-0.42351600	11.50002900
H	5.68386900	0.80328400	10.82049200
H	0.90542700	-1.21772300	13.47414700
H	5.14133500	8.59055400	9.55514300
H	-2.23683100	2.56437000	7.61568900
H	4.76495200	-0.64386400	9.01987400
H	3.10925500	-0.22101000	9.47698500
H	4.19002000	1.01519400	8.80864300
C	1.84069300	2.47439300	12.53914000
H	1.19011500	2.51511100	11.64209800

TS7

G +ZPE = -1077.596849 a.u

N	-0.74785900	-0.00087800	-0.68389400
C	0.60986900	0.17397100	-0.98414000
C	-1.72731700	0.72835400	-1.53696600
C	1.40084500	-1.03328100	-1.35859900
H	0.76849000	1.01856600	-1.64382400
C	-1.11634400	-1.23222600	-0.05749100
C	-3.01922500	1.14665100	-0.86855000
H	-1.21003500	1.61739400	-1.90143800

H	-1.96398000	0.11061800	-2.41104600
C	0.56411800	-2.28469700	-1.55890800
C	2.73727400	-1.40370000	0.08087600
H	2.10348800	-0.82853200	-2.16078800
C	0.75887200	2.15145000	0.90080400
C	2.86066100	0.94106800	0.39882800
C	-0.46421200	-2.40802100	-0.46380400
C	-2.06077200	-1.27538400	0.97225100
C	-4.19360400	0.42170800	-1.09012600
C	-3.07506800	2.28365000	-0.05410600
H	0.05903500	-2.20696700	-2.53197500
H	1.19599000	-3.17301900	-1.60817200
N	3.50170200	-0.22255000	0.16590200
H	3.31486400	-2.25670200	-0.25092600
H	2.11334100	-1.61781400	0.94309500
C	1.41731100	3.33903800	0.85754400
H	-0.29553900	2.10811200	1.14055700
C	3.52265900	2.20026100	0.36694800
C	-0.77767800	-3.61375000	0.16052900
C	-2.38136700	-2.49273200	1.57022700
H	-2.53370200	-0.36250900	1.30688500
C	-5.39382200	0.80814300	-0.49486400
H	-4.16789900	-0.45331200	-1.73080700
C	-4.27093900	2.67166300	0.54505900
H	-2.18196500	2.87867100	0.10113700
C	4.92995600	-0.28507000	-0.22281500
C	2.81449600	3.35340200	0.57950700
H	0.90054100	4.27006200	1.05326200
H	4.58727600	2.25665900	0.19548000
C	-1.74161500	-3.66286900	1.16697000
H	-0.26777900	-4.51992800	-0.14879400
H	-3.11912300	-2.52028400	2.36369100
C	-5.43367200	1.93107900	0.32916700
H	-6.29519000	0.23376900	-0.67610000
H	-4.29826600	3.55608800	1.17143700
C	5.85848400	-0.40161900	0.98474400
H	5.16998600	0.58932600	-0.82638900
H	5.03738700	-1.15457600	-0.87065300
H	3.33676600	4.30245700	0.54650800
H	-1.98241500	-4.60744300	1.64066400
H	-6.36554100	2.23418100	0.79271700
H	6.89255900	-0.47135900	0.63987700
H	5.77406000	0.46818700	1.63923700
H	5.63031800	-1.29648300	1.56749500

C	1.42065000	0.90023600	0.60044100
H	1.06143300	0.06644100	1.19784600s

C_1

G +ZPE = -1077.240477 a.u

N	2.04142500	4.56030600	11.72204200
C	2.65656000	3.83745800	12.86338500
C	0.58004500	4.59348500	11.67754700
C	4.16324400	3.66773900	12.62759300
C	2.08791500	2.45324500	13.08291300
H	2.50901300	4.41963200	13.78499500
C	2.73357200	5.64410000	11.16126900
C	-0.05473200	4.07182600	10.39476300
H	0.21267600	3.98003100	12.50060000
H	0.21622400	5.61087900	11.86998600
C	4.83213800	5.03176800	12.46822000
C	4.42119500	2.77750500	11.40713500
H	4.56300900	3.17402300	13.51729800
C	1.17138900	2.20510200	14.10125500
C	2.58252000	1.37751900	12.29802800
C	4.09318500	5.89875200	11.47303800
C	2.09767000	6.50606500	10.24100800
C	-1.32098900	4.53189300	10.01646200
C	0.57035200	3.10047100	9.60873200
H	4.85796700	5.52662700	13.44601000
H	5.87554300	4.91430600	12.15834000
N	3.50506400	1.63169400	11.29225500
H	5.45204300	2.40410400	11.47069300
H	4.35731500	3.37888000	10.49583400
C	0.67553700	0.92541300	14.35083000
H	0.83660300	3.03940800	14.71053200
C	2.08839900	0.08396400	12.57079600
C	4.74898500	6.96921600	10.86544700
C	2.77437400	7.57296900	9.65868500
H	1.06938700	6.33270800	9.95875100
C	-1.95274100	4.03006300	8.87984600
H	-1.81528600	5.29230100	10.61379900
C	-0.05869700	2.59994400	8.46885700
H	1.55239100	2.74182600	9.89463900
C	3.96796200	0.54743300	10.42504300
C	1.14206700	-0.12803500	13.56990000
H	-0.04674200	0.75729400	15.14053800
H	2.45681900	-0.76985500	12.02004900

C	4.10999900	7.81811500	9.96484300
H	5.79229000	7.13874100	11.11555400
H	2.24714900	8.20938200	8.95599000
C	-1.32211100	3.06060800	8.10037900
H	-2.93315300	4.40001700	8.60030000
H	0.43980500	1.84731200	7.86737200
C	4.47239500	1.02790700	9.06296500
H	3.12895000	-0.12610600	10.25116800
H	4.75653800	-0.04013400	10.92306900
H	0.78593600	-1.13724700	13.74795600
H	4.64401500	8.64464800	9.51097900
H	-1.80898400	2.67172600	7.21305900
H	4.71174600	0.15763100	8.44700900
H	3.70703900	1.61115500	8.54452300
H	5.37595300	1.63528800	9.13883100

int-6

G +ZPE = -673.155089 a.u

C	-2.34165300	-0.04883300	-1.47462200
C	-0.78792200	-1.34919100	-0.07347700
C	-2.70233700	-3.34560700	0.37041000
C	-3.11761200	1.04581500	-0.75180100
H	-1.40130700	0.35471700	-1.85998400
H	-2.92189500	-0.37787600	-2.34273000
C	-0.45875600	-2.39572600	0.72452100
H	-0.06340700	-0.58340200	-0.32670300
C	-1.39035100	-3.50106500	0.87151400
C	-3.59062600	-4.41612100	0.39715800
C	-4.29911000	1.55036300	-1.30090500
C	-2.65729800	1.58044100	0.45763000
H	0.54270200	-2.46940700	1.12740700
C	-1.02381300	-4.74194100	1.41858500
C	-3.21347100	-5.64517700	0.94412500
H	-4.59346000	-4.28822500	0.00079400
C	-5.00380400	2.57331700	-0.66546600
H	-4.67164200	1.14032000	-2.23430900
C	-3.36035700	2.59833300	1.09733300
H	-1.74612500	1.19799300	0.90481000
C	-1.92751000	-5.80015600	1.46158800
H	-0.01688200	-4.87311200	1.80205200
H	-3.91802000	-6.46856600	0.96835500
C	-4.53588400	3.09982800	0.53627800
H	-5.91847400	2.95312600	-1.10692200

H	-2.99071400	3.00217200	2.03333300
H	-1.62547800	-6.74917200	1.89128300
H	-5.08247200	3.89237600	1.03467700
C	-3.13909500	-1.97508400	-0.10560800
H	-3.91312400	-2.06473600	-0.87252300
H	-3.58703500	-1.41513500	0.73451400
N	-2.01676600	-1.21628000	-0.66503500

int-6'

G +ZPE = -1103.225968 a.u

C	-3.56138300	-0.56324300	1.08327900
N	-2.80794200	-0.65587800	-0.19041400
C	-3.46223600	-1.81759300	1.91347900
C	-3.22706000	0.27978000	-1.27501400
C	-1.84300000	-1.49263300	-0.35408700
H	-3.17466500	0.30878900	1.61808200
H	-4.59929800	-0.34987700	0.82520600
C	-4.43810100	-2.08034300	2.87777500
C	-2.40437100	-2.70947800	1.72293000
C	-3.16407300	1.72904900	-0.84659100
H	-4.24474800	0.00171500	-1.55242000
H	-2.57470900	0.08518700	-2.12532900
C	-1.34809000	-2.39869300	0.69642100
H	-1.34445400	-1.47539500	-1.31791200
O	-0.67773700	-2.41892800	5.46679900
C	-4.35644800	-3.22652100	3.66241500
H	-5.26323400	-1.38955400	3.01165400
C	-2.34996700	-3.87941600	2.48778000
C	-1.92918400	2.35536600	-0.64220900
C	-4.34054200	2.46161800	-0.66903200
C	-0.02445900	-1.74821800	1.34240500
H	-0.98890900	-3.30971700	0.21127600
C	-0.57402600	-2.40220100	6.91602700
Mg	0.76275900	-2.66085200	4.06666200
H	-1.58091500	-2.63523700	5.19840000
C	-3.31230100	-4.13159900	3.46274000
H	-5.11460600	-3.42397000	4.41072400
H	-1.54803500	-4.58682000	2.31680800
C	-1.87547300	3.69149600	-0.25582900
H	-1.00790500	1.80144400	-0.78817100
C	-4.28635700	3.80138300	-0.28708800
H	-5.30174300	1.98737200	-0.83460700
O	0.58243200	-2.62941500	2.19287000

H	0.62794400	-1.48074700	0.49674300
H	-0.34215200	-0.80738300	1.82185200
H	-1.26784300	-1.66512600	7.31806800
H	-0.78891200	-3.39269800	7.31660500
H	0.44727500	-2.11620500	7.15376900
H	-3.25860900	-5.03975800	4.05176500
C	-3.05447300	4.41648700	-0.07689100
H	-0.91516800	4.16916900	-0.10054500
H	-5.20497800	4.36096000	-0.15530200
H	-3.01108800	5.45765100	0.22102300

TS8

G +ZPE = -1103.221757 a.u

C	-3.59398900	-0.63263600	1.08516600
N	-2.89327300	-0.70169600	-0.21517300
C	-3.43067500	-1.88513700	1.91621800
C	-3.31441600	0.27491900	-1.24344000
C	-1.94628600	-1.57886100	-0.44475200
H	-3.21495500	0.24512100	1.61936200
H	-4.65036600	-0.44549700	0.87856100
C	-4.29991500	-2.12456900	2.97871600
C	-2.39994500	-2.79463800	1.63269100
C	-3.18703100	1.71537000	-0.79045800
H	-4.35256600	0.05380400	-1.50120800
H	-2.70426600	0.08859500	-2.12764400
C	-1.48482000	-2.51215400	0.51354100
H	-1.47939400	-1.52848400	-1.42250900
O	-0.82644200	-2.38181300	5.42737700
C	-4.14742700	-3.25896200	3.77654300
H	-5.10135900	-1.42360800	3.18646100
C	-2.27990900	-3.95246600	2.41087100
C	-1.93575500	2.26195500	-0.48268600
C	-4.32309100	2.52285400	-0.70058800
C	0.16312200	-1.60538000	1.27731100
H	-0.95657700	-3.35934300	0.09025000
C	-0.80076700	-2.26747400	6.87588200
Mg	0.68633500	-2.53702800	4.09522000
H	-1.71438800	-2.61627300	5.12059600
C	-3.13791600	-4.17814600	3.48657500
H	-4.82647800	-3.43459000	4.60233100
H	-1.50929300	-4.67599200	2.16992000
C	-1.82717400	3.59235600	-0.08742800
H	-1.04322400	1.64872200	-0.55246300

C	-4.21473100	3.85752700	-0.31085500
H	-5.29663300	2.10820100	-0.93945600
O	0.67056100	-2.37510400	2.18766700
H	0.72014900	-1.53156200	0.34002000
H	-0.31412500	-0.67051000	1.58877200
H	-1.49112300	-1.48505700	7.18846400
H	-1.06898300	-3.22222400	7.32721700
H	0.21546300	-1.99690300	7.15141400
H	-3.03173000	-5.07740300	4.08233100
C	-2.96701500	4.39347300	-0.00171800
H	-0.85367300	4.00663300	0.14816800
H	-5.10400800	4.47393100	-0.24824000
H	-2.88069800	5.43001400	0.30308600

TS9

G +ZPE = -1103.220292 a.u

C	-2.12269500	0.45767200	-0.55896600
C	-0.60969900	-1.26900600	0.21531700
C	-2.66624800	-2.91410500	1.22010300
C	-2.69540600	1.50866600	0.36576400
H	-1.19440600	0.79355500	-1.01955200
H	-2.82813500	0.20717400	-1.35190600
H	0.16274100	-0.67779700	-0.26284700
C	-1.35930600	-3.39686200	1.28871300
C	-3.73666100	-3.74791200	1.55731600
C	-4.02946800	1.90531000	0.24242900
C	-1.88651900	2.11351400	1.33488200
C	-1.13532600	-4.72017400	1.68400600
C	-3.50550500	-5.05566700	1.96723700
H	-4.75150900	-3.37039100	1.49624800
C	-4.55100900	2.89145700	1.07868300
H	-4.66156700	1.44776600	-0.51082400
C	-2.40910700	3.09403900	2.17284200
H	-0.84596400	1.82308800	1.43400900
C	-2.20012200	-5.54512100	2.02725600
H	-0.12256700	-5.10640400	1.71675200
H	-4.34047500	-5.69436100	2.22931300
C	-3.74279100	3.48454100	2.04574400
H	-5.58601300	3.19441700	0.97280100
H	-1.77537000	3.55813000	2.91937200
H	-2.01450300	-6.56756900	2.33383600
H	-4.14751600	4.25103300	2.69632400
C	-2.96034800	-1.50152100	0.78736800

H	-3.77699300	-1.47975400	0.06347600
H	-3.26138200	-0.87790200	1.63358000
N	-1.80985000	-0.82011400	0.15208300
C	-0.19872900	-2.50222300	0.92730400
H	0.49326700	-3.02351900	0.25531700
C	0.65807900	-2.09407500	2.17309000
H	0.02862300	-1.54923400	2.88393800
H	0.98822500	-3.01946200	2.65123300
O	1.76104100	-1.30896400	1.77628900
H	1.88812600	-0.27390500	2.24988600
Mg	3.72880900	-1.13049900	2.35629100
O	2.75141200	0.50861100	2.81853700
C	2.86241300	1.88852900	2.52682900
H	2.05848300	2.45015000	3.01738800
H	3.81655700	2.28296500	2.89278200
H	2.80605600	2.08687100	1.44819500

in-7

G +ZPE = -1103.228384 a.u

C	-0.57164500	1.99690000	-1.73956600
C	0.84608100	0.94511300	-0.07253800
C	-0.77111700	-1.33271400	0.11075500
C	-1.98659500	2.44975900	-1.45776700
H	0.14565000	2.79941800	-1.56752700
H	-0.47121100	1.66851000	-2.77548100
H	1.34113100	1.90960500	-0.02198200
C	0.31580800	-1.23505400	0.98526800
C	-1.76599900	-2.28641200	0.32948300
C	-2.95844700	2.37247300	-2.45841500
C	-2.32742600	2.98483500	-0.21045400
C	0.39707700	-2.11108700	2.07132500
C	-1.67441600	-3.16085600	1.40827600
H	-2.61115900	-2.34574400	-0.34725900
C	-4.25501700	2.82610800	-2.21837100
H	-2.70086500	1.96274600	-3.42906900
C	-3.62315200	3.43113800	0.03099400
H	-1.58121300	3.05808500	0.57335800
C	-0.59025400	-3.07145800	2.27864900
H	1.23685000	-2.05623800	2.75155600
H	-2.44631900	-3.90374300	1.56953300
C	-4.58899300	3.35365400	-0.97346800
H	-5.00041300	2.76517800	-3.00260100
H	-3.87836100	3.84485100	0.99959300

H	-0.51120600	-3.74715200	3.12209500
H	-5.59665500	3.70541800	-0.78501500
C	-0.85240300	-0.43228200	-1.09470000
H	-0.38890800	-0.89676200	-1.97324600
H	-1.88653400	-0.20272000	-1.34752200
N	-0.15052600	0.85353800	-0.88390900
C	1.37219100	-0.18266700	0.70589400
H	2.14493500	-0.65782800	-0.00355800
C	2.18968100	0.30824700	1.90356000
H	2.65477600	1.27052600	1.68471800
H	1.57421700	0.40430400	2.79817700
O	3.25167800	-0.65038400	2.19554800
H	3.52190800	-0.55043800	3.11685000
Mg	4.55364700	-1.57466000	0.87567300
O	3.47111600	-1.47308100	-0.65184300
C	3.34291800	-2.06939300	-1.91466900
H	2.46078700	-1.67335500	-2.43984100
H	4.21261600	-1.87142600	-2.55826200
H	3.21958500	-3.16100700	-1.85523100

TS10

G +ZPE = -1103.223018 a.u

C	-2.14170400	0.51110900	-1.28738500
C	-0.72655100	-0.55513400	0.38200500
C	-2.36207100	-2.81516800	0.55749200
C	-3.55464500	0.97884000	-1.00870700
H	-1.42071600	1.31033000	-1.11280500
H	-2.04416700	0.19111900	-2.32699200
H	-0.21441700	0.40115400	0.41416000
C	-1.26800300	-2.72141200	1.42732800
C	-3.35307400	-3.77234400	0.77443100
C	-4.52446300	0.92135400	-2.01248700
C	-3.89837700	1.49830800	0.24458900
C	-1.17866200	-3.61476500	2.50063600
C	-3.25446700	-4.65953600	1.84295000
H	-4.20263500	-3.82559200	0.10225900
C	-5.82016300	1.37745900	-1.77044400
H	-4.26620300	0.52249300	-2.98760900
C	-5.19256200	1.94832600	0.48837600
H	-3.15455000	1.55390100	1.03219800
C	-2.16272200	-4.57936200	2.70456500
H	-0.33496700	-3.56462300	3.17705700
H	-4.02442400	-5.40527600	2.00084500

C	-6.15619600	1.88928700	-0.51960100
H	-6.56344400	1.32996000	-2.55773700
H	-5.44886000	2.34930300	1.46215800
H	-2.07566600	-5.26529200	3.53912300
H	-7.16308700	2.24233400	-0.32913700
C	-2.45128300	-1.90626900	-0.64247700
H	-2.00729700	-2.37624900	-1.52954200
H	-3.48844800	-1.67149400	-0.87968200
N	-1.73795400	-0.63063500	-0.43149100
C	-0.23625500	-1.65085000	1.17734800
H	0.63991400	-2.17915700	0.43066300
C	0.56804900	-1.16693900	2.37797400
H	1.00089200	-0.18450300	2.18546700
H	-0.04506000	-1.11729300	3.27876100
O	1.67138100	-2.09121300	2.66473600
H	1.94357000	-1.97980000	3.58402100
Mg	2.93737800	-3.03082200	1.34330100
O	1.73342500	-2.83617900	-0.11787200
C	1.55593600	-3.41766100	-1.39517800
H	0.75373800	-2.90242100	-1.93843000
H	2.46776600	-3.32992000	-1.99652600
H	1.28934100	-4.47948600	-1.32754900

in-7'

G +ZPE = -1103.235332 a.u

C	-0.53279200	1.94673000	-1.74805800
C	0.86260700	0.92450100	-0.00032100
C	-0.76429000	-1.34438900	0.12278200
C	-1.94439700	2.44354500	-1.48573800
H	0.18821600	2.75113900	-1.58976800
H	-0.44003900	1.62417500	-2.79045200
H	1.39636200	1.86887700	-0.00623200
C	0.28825000	-1.20232500	1.05069600
C	-1.72082300	-2.34068300	0.29694500
C	-2.91739100	2.39488900	-2.48620200
C	-2.28913000	2.95770900	-0.23000800
C	0.37006400	-2.11626600	2.11308400
C	-1.63387900	-3.23598200	1.36301500
H	-2.53664600	-2.42604500	-0.41359000
C	-4.21338200	2.85172300	-2.24010600
H	-2.66271100	1.99675500	-3.46301200
C	-3.58031400	3.41340700	0.01819900
H	-1.54422800	2.99892400	0.55757100

C	-0.57779400	-3.12530900	2.26441300
H	1.18218700	-2.04161400	2.82537300
H	-2.37910900	-4.01367700	1.48191200
C	-4.54731300	3.36026100	-0.98757800
H	-4.95907300	2.80552000	-3.02560900
H	-3.83357300	3.81118200	0.99441700
H	-0.49286400	-3.82010900	3.09229600
H	-5.55361200	3.71353600	-0.79360700
C	-0.81529400	-0.45322100	-1.09834100
H	-0.32493900	-0.94662800	-1.95330700
H	-1.85048200	-0.26609200	-1.38897900
N	-0.15723800	0.83734800	-0.86667600
C	1.20967400	-0.06133900	0.90118700
H	2.50343200	-0.92131700	-0.43540300
C	2.15556000	0.31813600	1.98820900
H	2.58773700	1.30174000	1.80841900
H	1.66879800	0.31495800	2.96610000
O	3.29681000	-0.63705500	2.14266300
H	3.56778900	-0.63275800	3.06959000
Mg	4.57239400	-1.54616000	0.85213700
O	3.32811700	-1.39329200	-0.68815100
C	3.19962200	-2.04365900	-1.97565100
H	2.73298600	-1.35678900	-2.67870600
H	4.20172100	-2.29033100	-2.31632100
H	2.60029700	-2.95071100	-1.87851500

in-8

G +ZPE = -711.669251 a.u

C	-1.94922300	0.37812500	-1.12270100
C	-0.73306900	-1.13985900	0.33122200
C	-2.70719600	-3.14101900	0.26843900
C	-3.12026900	1.19057400	-0.61443700
H	-1.01115600	0.92249000	-1.02203000
H	-2.08222300	0.12177600	-2.17473300
H	0.01512400	-0.35619600	0.37324400
C	-1.56749100	-3.37973000	1.04810100
C	-3.74095600	-4.08072800	0.23619500
C	-4.25764300	1.35923900	-1.40849700
C	-3.06488400	1.80225800	0.64319200
C	-1.48725100	-4.56676300	1.79179100
C	-3.64735100	-5.25482000	0.97161800
H	-4.62061200	-3.88876600	-0.36812500
C	-5.32829800	2.12690700	-0.95197400

H	-4.30502400	0.89440500	-2.38730100
C	-4.13569700	2.56466500	1.10034600
H	-2.18382600	1.68921300	1.26584800
C	-2.51509900	-5.49705000	1.75274900
H	-0.61972300	-4.76481100	2.40802600
H	-4.45377400	-5.97752100	0.94041100
C	-5.26930400	2.72816000	0.30311000
H	-6.20433500	2.25420600	-1.57700000
H	-4.08355200	3.03624500	2.07465500
H	-2.43802900	-6.40908100	2.33213500
H	-6.10085800	3.32523700	0.65899800
C	-2.86092300	-1.89499700	-0.56339700
H	-2.88452700	-2.13978900	-1.63061500
H	-3.79960900	-1.38699300	-0.33068000
N	-1.78213300	-0.90734500	-0.38592500
C	-0.49949600	-2.36140900	1.06661100
C	0.68614300	-2.44631100	1.70585200
H	1.39842800	-1.63223700	1.66293600
H	0.97718700	-3.31732200	2.27695400

TS11

G +ZPE = -1655.607731 a.u

C	-0.52589700	-2.12956200	-1.01355000
C	-0.39061100	-1.92254400	1.40656700
C	-2.83175100	-3.23124900	1.85643400
C	-1.47509600	-1.30573800	-1.85962700
H	0.44190000	-1.63692200	-0.91694400
H	-0.36034800	-3.10745600	-1.47137100
H	0.51969100	-1.36638700	1.21581100
C	-2.12385100	-2.74456600	2.96725600
C	-4.10394600	-3.77923200	2.02449400
C	-2.00776800	-1.83761100	-3.03632400
C	-1.80240500	0.00440100	-1.49073100
C	-2.72159100	-2.81348700	4.23517200
C	-4.68068900	-3.85353900	3.28809000
H	-4.64506600	-4.15064500	1.16087900
C	-2.85388600	-1.07203300	-3.83833200
H	-1.75882300	-2.85190200	-3.32926900
C	-2.65170800	0.76606300	-2.28847300
H	-1.39823300	0.43107100	-0.57880500
C	-3.98523600	-3.36736000	4.39584300
H	-2.20001100	-2.42604800	5.10137700
H	-5.66865300	-4.28189800	3.40741600

C	-3.17774900	0.23008400	-3.46485200
H	-3.25916400	-1.49458900	-4.75041500
H	-2.89977200	1.77945000	-1.99472600
H	-4.43064200	-3.41436800	5.38249300
H	-3.83640800	0.82610400	-4.08598100
C	-2.23271900	-3.20843500	0.47188400
H	-1.94286000	-4.21856300	0.15677900
H	-2.96038800	-2.84326900	-0.25567500
N	-1.03130500	-2.36411800	0.35825400
C	-0.80101300	-2.14056700	2.74851000
C	0.04657600	-1.69901000	3.73810400
H	1.03269700	-1.33913500	3.47640100
H	-0.09101000	-1.99526900	4.76834000
H	-0.36833500	-0.08362100	4.21753800
C	-1.37691200	1.70476600	6.82467900
C	-1.89270200	2.93862100	6.28221300
C	-1.09610000	3.61881000	5.36786100
C	0.17268000	3.07320400	4.97868800
C	0.77412200	1.97561300	5.67146700
C	-0.03973300	1.29997300	6.62812700
H	-2.01454600	1.11096200	7.46807400
H	-1.47229100	4.49971200	4.86374700
H	0.72526400	3.56615200	4.19051800
H	0.33102700	0.41230800	7.12374700
C	2.21142500	1.54584200	5.44775500
H	2.25716300	0.47416400	5.66609700
C	2.72192200	1.76049300	4.01766100
H	3.71725100	1.32193200	3.91621000
H	2.06544700	1.29044200	3.28237200
H	2.80855200	2.82234900	3.77151100
C	3.10818500	2.27270200	6.47194600
H	2.78201800	2.08242700	7.49721400
H	4.14034800	1.92732400	6.37414600
H	3.09423200	3.35355800	6.30440200
C	-3.27037700	3.40117200	6.65523900
H	-3.98228100	2.57295000	6.63341800
H	-3.26210900	3.79534600	7.67677200
H	-3.62337900	4.18680600	5.98724300
Ru	-1.05668900	1.33137200	4.66510000
Cl	-1.61764200	1.48442500	2.30039500

int-9

G +ZPE = -712.427423 a.u

C	-2.17742300	0.11607700	-1.33896800
C	-0.93868600	-1.13604200	0.38143800
C	-2.77972500	-3.23028900	0.33495600
C	-3.15024300	1.14311200	-0.77164500
H	-1.19741200	0.57512300	-1.49317800
H	-2.53612100	-0.20247800	-2.32280700
H	-0.22616900	-0.32032800	0.31970700
C	-1.62431300	-3.32382700	1.14597100
C	-3.59042000	-4.34269900	0.13729000
C	-4.30131200	1.49353100	-1.48195800
C	-2.91067700	1.75977100	0.46289900
C	-1.34786200	-4.55548500	1.76503100
C	-3.29997500	-5.56104900	0.75666100
H	-4.46870100	-4.25585100	-0.49522800
C	-5.19274700	2.44206600	-0.97895400
H	-4.50221400	1.02137600	-2.43848200
C	-3.79946700	2.70410300	0.97028300
H	-2.02479000	1.50087200	1.03264200
C	-2.17900900	-5.65832800	1.57776300
H	-0.47017800	-4.65473600	2.39259400
H	-3.94542500	-6.41808100	0.60202700
C	-4.94392800	3.04945500	0.24946700
H	-6.08020600	2.70200800	-1.54518600
H	-3.59854400	3.17383100	1.92674900
H	-1.94411300	-6.59649500	2.06889300
H	-5.63489400	3.78553300	0.64453600
C	-3.16370700	-1.88029100	-0.22909900
H	-3.73894400	-1.99864400	-1.15119400
H	-3.81681400	-1.35167700	0.48932700
N	-1.97891400	-1.07348600	-0.51674400
C	-0.73691800	-2.16437400	1.24562800
C	0.45967600	-2.17977700	2.16108200
H	0.17110100	-2.31259200	3.21021200
H	1.15451500	-2.99367600	1.92014000
H	1.01400100	-1.24155200	2.08846300

TS12

G +ZPE = -1116.909685 a.u

C	-2.25981400	-1.37785500	8.61889300
C	0.27711400	-2.48789500	5.98172300
C	-1.39639200	-0.85490200	9.60071200
C	-3.61039500	-1.56727600	8.90571900
H	-0.16448900	-3.48580700	5.90991600

H	1.34669100	-2.61207100	6.15301300
C	0.03066200	-1.72232500	4.69400300
C	-1.93108700	-0.52954900	10.85812800
C	-4.12983900	-1.22929900	10.15384700
H	-4.26375700	-1.98224100	8.14520700
C	-0.97523500	-2.12250900	3.81005900
C	0.81330000	-0.60841500	4.36798600
H	-1.28275500	-0.13976100	11.63297500
C	-3.28398700	-0.70817400	11.13078200
H	-5.18321200	-1.37653600	10.36080400
H	-1.57966700	-2.99312800	4.04219600
C	-1.20492500	-1.41788100	2.62842800
C	0.58608200	0.09700600	3.18906600
H	1.61368500	-0.29660100	5.03097700
H	-3.67419600	-0.44735200	12.10780900
H	-1.98829600	-1.74067200	1.95233100
C	-0.42637600	-0.30538200	2.31725600
H	1.20315600	0.95462100	2.94660100
H	-0.60169700	0.24207400	1.39828600
C	0.49170700	-1.34320100	8.13994100
C	0.04003300	-0.71281500	9.29561300
C	0.09875500	1.46367200	8.23010900
H	-0.97580000	1.37594400	8.23913200
H	0.62953600	1.16611600	7.34141800
N	0.64428700	2.35693900	9.03859000
C	-0.24810700	3.02735300	10.02719200
H	-1.01546300	2.30291300	10.29477600
H	0.33066900	3.22619700	10.92701100
C	-0.89174900	4.29723200	9.47196500
H	-0.14989800	5.04220900	9.18138000
H	-1.51136900	4.07049000	8.60182100
H	-1.53209600	4.73484400	10.24095400
C	2.03291800	2.71256700	8.96471500
C	2.93348100	1.92542200	8.23390700
C	2.50494000	3.85412400	9.62431900
C	4.27489600	2.28437100	8.15966900
H	2.61203800	1.02375800	7.73273600
C	3.85063500	4.20164100	9.54305700
H	1.84041000	4.48714100	10.19325800
C	4.74307000	3.42348800	8.81135200
H	4.95596800	1.65984800	7.59410700
H	4.19505000	5.09117600	10.05665700
H	5.78929300	3.69745200	8.75230600
H	1.55729900	-1.46592100	7.97946500

N	-0.28612100	-1.83617900	7.17704900
C	-1.75031700	-1.71813100	7.23522900
H	-2.07028400	-0.96057200	6.50887000
H	-2.17702900	-2.66852600	6.90055400
C	1.02793800	-0.47247700	10.41031600
H	2.05389400	-0.52874700	10.04311900
H	0.90293300	0.50914600	10.87657500
H	0.91389500	-1.21881700	11.20359100

TS12'

G +ZPE = -1116.906388 a.u

C	-1.09765700	2.69140300	-0.16339600
C	-3.17542400	-0.04349700	1.53481300
C	0.17928700	2.39304700	0.34365200
C	-1.31011400	3.86967300	-0.87846900
H	-3.92257400	0.67796100	1.87502600
H	-2.85544500	-0.62416700	2.39992900
C	-3.76989600	-0.95008100	0.47431100
C	1.22254400	3.30212600	0.10492300
C	-0.26437500	4.75838000	-1.11468600
H	-2.30335600	4.09432700	-1.25331100
C	-4.89267400	-0.55358600	-0.25777900
C	-3.20496600	-2.20530400	0.22101400
H	2.21233400	3.09925200	0.49400000
C	1.00581500	4.46993100	-0.61992500
H	-0.44221100	5.66861800	-1.67501900
H	-5.35300100	0.40850100	-0.05937100
C	-5.43285800	-1.38828100	-1.23584700
C	-3.74325600	-3.04105700	-0.75424000
H	-2.35047200	-2.53874700	0.80021900
H	1.82735300	5.15577900	-0.79161700
H	-6.30416700	-1.06827600	-1.79552700
C	-4.85733900	-2.63178800	-1.48771000
H	-3.29977900	-4.01333300	-0.93564200
H	-5.27826100	-3.28286000	-2.24518900
C	-0.78920100	0.49719600	1.53656300
C	0.38334100	1.14753300	1.12074800
C	0.92504800	-0.23750400	-0.50029400
H	1.42629600	0.60756000	-0.95115600
H	-0.09523100	-0.42696700	-0.80552900
N	1.68536700	-1.30406100	-0.23192100
C	1.07541200	-2.64319300	-0.04284100
H	0.00850100	-2.52714500	-0.23427600

H	1.47930200	-3.29113000	-0.82586500
C	1.29660600	-3.28210600	1.32607900
H	2.34749400	-3.51407000	1.50246400
H	0.93703000	-2.64433200	2.13571600
H	0.74042500	-4.22201800	1.36262100
C	3.12163800	-1.22505000	-0.37506400
C	3.97051000	-1.37052600	0.72318200
C	3.65365700	-1.02396600	-1.65131100
C	5.35090500	-1.31135500	0.53984100
H	3.56042700	-1.50840500	1.71429700
C	5.03384900	-0.95775000	-1.82509000
H	2.98744600	-0.92735900	-2.50056000
C	5.88471900	-1.10387500	-0.73076400
H	6.00715600	-1.41884400	1.39530500
H	5.44160300	-0.79872500	-2.81633700
H	6.95857300	-1.05538000	-0.86719400
H	-0.72609300	-0.28065900	2.28959000
N	-2.00412200	0.72546900	1.06540100
C	-2.26244700	1.74944900	0.04098300
H	-2.52149300	1.24271300	-0.89564900
H	-3.15081600	2.30903200	0.34921900
C	1.58177700	1.04509400	2.04368600
H	1.58433000	0.09054900	2.57215100
H	2.52593500	1.12411400	1.50341100
H	1.56585500	1.84150100	2.79516300

int-10a

G +ZPE = -1116.920512 a.u

C	-2.28080000	-1.24749400	8.16982200
C	1.22138100	-1.82989100	6.71185800
C	-2.03134200	-0.25627800	9.12352100
C	-3.59277800	-1.56485400	7.80850800
H	1.37259400	-2.87653400	6.98184700
H	2.12637100	-1.27848700	6.96254100
C	0.87524400	-1.69643900	5.24697400
C	-3.11868700	0.39535000	9.71867100
C	-4.66430800	-0.91204900	8.40594000
H	-3.77178900	-2.32738400	7.05836300
C	0.56167900	-2.82893800	4.48982300
C	0.89166900	-0.44145600	4.62800500
H	-2.95721600	1.16019700	10.46727700
C	-4.42429000	0.06885600	9.36649100
H	-5.67889900	-1.16487600	8.12233900

H	0.55760100	-3.80687500	4.95889300
C	0.26110800	-2.70924100	3.13374100
C	0.58772700	-0.32192200	3.27500800
H	1.15122600	0.44146000	5.20120000
H	-5.25184300	0.58316800	9.84038700
H	0.02227200	-3.59384700	2.55519000
C	0.27087800	-1.45575200	2.52598200
H	0.60516600	0.65371700	2.80355300
H	0.03800500	-1.36168600	1.47166000
C	0.37073400	-0.39020000	8.47317300
C	-0.59442200	0.07736400	9.51017300
C	-0.41232300	1.62933100	9.74559900
H	-0.90267900	1.86273500	10.69036200
H	-0.96493900	2.16628500	8.97493600
N	0.96215000	2.09285400	9.82130100
C	1.47381200	2.38557400	11.17159500
H	1.03883400	1.64939500	11.84698500
H	2.54967800	2.20446600	11.19216700
C	1.15105600	3.79572100	11.67700700
H	1.58693000	4.56354100	11.03493900
H	0.07091200	3.95941600	11.71828900
H	1.55056400	3.92909900	12.68588000
C	1.57337200	2.67282300	8.70172100
C	0.97591300	2.63416100	7.42253800
C	2.84076100	3.29095900	8.79865800
C	1.61310800	3.18607100	6.31211400
H	0.00238700	2.18745000	7.27374400
C	3.46444200	3.83162500	7.68074200
H	3.34801400	3.35651900	9.75012000
C	2.86165700	3.78905500	6.42375500
H	1.11300800	3.14392000	5.35068400
H	4.43640400	4.29752100	7.80121900
H	3.35124100	4.21774200	5.55790600
H	1.36961100	0.03186600	8.49144000
N	0.14411700	-1.31929900	7.61650500
C	-1.15424200	-2.01861000	7.53767900
H	-1.35356200	-2.20697300	6.48327400
H	-1.01145300	-2.99108000	8.02191700
C	-0.19023300	-0.70043500	10.80449700
H	0.85170900	-0.51620900	11.07168300
H	-0.82924900	-0.37023800	11.62550900
H	-0.32829800	-1.77446000	10.67176400

int-10b

G +ZPE = -1116.919487 a.u

C	-3.78689700	2.20812400	7.00870700
C	-1.83538200	5.45871700	6.32033800
C	-2.84027500	1.45787000	7.71107100
C	-5.09654300	1.73751600	6.86998000
H	-1.89832900	5.44936600	5.23124400
H	-0.80315900	5.66252700	6.60068400
C	-2.78316500	6.48099900	6.90529500
C	-3.22624000	0.22375700	8.25149400
C	-5.46881700	0.51591700	7.41597500
H	-5.82343900	2.33270200	6.32797300
C	-3.75892600	7.07800700	6.10160900
C	-2.67578200	6.86166200	8.24784100
H	-2.50699600	-0.38493700	8.78533000
C	-4.52648600	-0.24566100	8.10626600
H	-6.48562300	0.15932100	7.30303800
H	-3.84183500	6.79832100	5.05689600
C	-4.62128800	8.03585300	6.63290300
C	-3.53931300	7.81532400	8.77902800
H	-1.91114400	6.42221000	8.87896500
H	-4.80316800	-1.20306800	8.53148600
H	-5.37210100	8.49420300	6.00008800
C	-4.51432400	8.40308600	7.97239100
H	-3.44698500	8.10513700	9.81913500
H	-5.18358500	9.14829200	8.38636000
C	-1.27040100	3.37755500	7.42912000
C	-1.41506900	1.96597700	7.87327000
C	-0.96745600	1.90387000	9.38730200
H	-0.95045600	0.84916600	9.66188700
H	-1.74683200	2.37648600	9.99064500
N	0.33297900	2.52795500	9.64173400
C	0.24484800	3.71111400	10.53109600
H	-0.61521800	4.29747500	10.19644400
H	0.03103800	3.40366500	11.56692800
C	1.48849100	4.59233300	10.50163400
H	2.36891700	4.06392200	10.87214000
H	1.70054900	4.93988300	9.48707900
H	1.32989300	5.46631600	11.13860100
C	1.39483700	1.61628500	9.99972400
C	2.59048600	1.64267500	9.27788800
C	1.26832800	0.72239100	11.07077900
C	3.64001700	0.78757200	9.61299100
H	2.68854600	2.33717600	8.45196300

C	2.31395800	-0.13763800	11.40025300
H	0.35122600	0.69818500	11.64942500
C	3.50351700	-0.10721500	10.67262200
H	4.56095800	0.81801000	9.04171800
H	2.20100300	-0.82773700	12.22887400
H	4.31660300	-0.77626600	10.92988600
H	-0.32771900	3.85964100	7.66945600
N	-2.13165100	4.06188100	6.76999000
C	-3.43529700	3.51435200	6.34869000
H	-4.18431700	4.27667000	6.56687400
H	-3.38799500	3.40706700	5.25984100
C	-0.42366600	1.14072600	7.00103500
H	0.59492300	1.51449500	7.10944200
H	-0.44714200	0.09987300	7.32831500
H	-0.70630000	1.17864500	5.94772300

TS13

G +ZPE = -1116.891526 a.u

C	1.76829500	5.28551000	11.70674900
C	-0.46239100	3.94403500	14.07406800
C	2.04903500	4.12493400	10.97816100
C	2.76592900	6.23415200	11.93825500
H	0.49098900	3.84762400	14.60846400
H	-0.95478500	2.97017900	14.14378200
C	-1.31132200	4.98738600	14.77970400
C	3.34705200	3.93886700	10.48660700
C	4.05060300	6.04243900	11.43973100
H	2.53363900	7.12295000	12.51526300
C	-1.11392200	5.18883700	16.15041800
C	-2.31110800	5.72040400	14.13298600
H	3.59695300	3.05148100	9.91971700
C	4.33934800	4.88928600	10.71309100
H	4.82129400	6.78189000	11.62354300
H	-0.33215800	4.63912900	16.66448200
C	-1.90749400	6.08534000	16.86223300
C	-3.10123500	6.62484800	14.84170400
H	-2.47054600	5.60344900	13.06797300
H	5.33720800	4.72473300	10.32344600
H	-1.73997400	6.22593700	17.92401300
C	-2.90569100	6.80719400	16.20911400
H	-3.86837100	7.18748800	14.32196200
H	-3.51994600	7.51053800	16.75938100
C	-0.17539700	3.15783300	11.76110600

C	0.92920600	3.11983600	10.67054900
C	0.38112400	3.51150800	9.25012200
H	1.21016500	3.52433800	8.54697500
H	-0.03268700	4.52304500	9.27581400
N	-0.60739400	2.55320200	8.77411300
C	-0.34713300	1.80326800	7.53033700
H	0.72119100	1.87735500	7.33199900
H	-0.55918000	0.74638700	7.69838400
C	-1.14297500	2.34144000	6.34057600
H	-2.21871400	2.26944500	6.51435200
H	-0.89407600	3.38692200	6.14596800
H	-0.90226000	1.75952700	5.44779400
C	-1.64900700	2.29226400	9.58330300
C	-1.82661800	3.15442200	10.74049900
C	-2.52705600	1.18939500	9.39768300
C	-2.91796100	2.85514100	11.63646600
H	-1.70267400	4.21139600	10.51531000
C	-3.50971000	0.93490200	10.32145300
H	-2.42797000	0.54498900	8.53662200
C	-3.70872400	1.76013800	11.46180700
H	-3.08905800	3.52203500	12.47223900
H	-4.15495600	0.07647000	10.17344300
H	-4.50143800	1.52835900	12.16149200
H	-0.29981300	2.19956900	12.25061400
N	-0.19762200	4.19292200	12.65247800
C	0.37328500	5.47691900	12.24824000
H	-0.27250300	5.95316700	11.49889900
H	0.38151000	6.14062500	13.11066000
C	1.45412300	1.66742600	10.62615900
H	2.13050300	1.50853900	9.78548700
H	1.98934600	1.43037300	11.54756200
H	0.63294700	0.95619900	10.52328300

TS14
G +ZPE = -1116.900475 a.u

C	-3.94088000	2.16963200	7.50306800
C	-1.50447100	4.43792100	5.73516400
C	-3.08714900	1.19949600	8.03242100
C	-5.29244700	1.88595400	7.29437600
H	-1.96464800	4.11931500	4.79847600
H	-0.42699100	4.30324800	5.65203000
C	-1.86362500	5.87256900	6.04618500
C	-3.60879000	-0.05771500	8.35416900

C	-5.80401100	0.63705800	7.62754200
H	-5.94058600	2.64362600	6.86842500
C	-2.88697500	6.50773100	5.33627100
C	-1.16248400	6.59061300	7.02195300
H	-2.96813800	-0.82762200	8.76330000
C	-4.95820700	-0.33557700	8.15764800
H	-6.85331500	0.42121200	7.46602500
H	-3.42482600	5.96710200	4.56498800
C	-3.21389500	7.83601200	5.60411800
C	-1.49114600	7.91582900	7.29204300
H	-0.35005900	6.12190300	7.56543500
H	-5.34617600	-1.31393000	8.41523700
H	-4.00688700	8.31829500	5.04486600
C	-2.51905800	8.54013800	6.58480600
H	-0.94072300	8.46306000	8.04823300
H	-2.77135600	9.57324600	6.79362400
C	-1.19215300	2.59718400	7.29354100
C	-1.61018000	1.54952100	8.28791200
C	-1.59135300	2.08903300	9.77143800
H	-2.00774400	1.24692800	10.33780100
H	-2.33069700	2.89452700	9.83504600
N	-0.35170000	2.53060600	10.44246800
C	-0.42307500	2.26079200	11.88993400
H	-1.45886300	2.44072200	12.18834300
H	-0.21706800	1.20324200	12.12105800
C	0.47828900	3.17251500	12.71828000
H	1.53567200	3.02015200	12.49389100
H	0.23648100	4.22192700	12.53180300
H	0.32870400	2.96777600	13.78147200
C	0.93502100	2.45777600	9.83762000
C	1.32391900	3.45612800	8.93431400
C	1.86764600	1.46054400	10.16591000
C	2.57728200	3.42707900	8.32389300
H	0.64490100	4.27988600	8.75106900
C	3.12916400	1.44720200	9.57649300
H	1.60248400	0.68681100	10.87638100
C	3.48610300	2.41989800	8.64167600
H	2.85160600	4.20914000	7.62488000
H	3.83343900	0.66633100	9.84125700
H	4.46723700	2.40242800	8.18186600
H	-0.16563100	2.60636400	6.94718800
N	-1.97835300	3.47251300	6.77828500
C	-3.40890900	3.53324300	7.15175700
H	-3.49894000	4.23859700	7.98310600

H	-3.95012400	3.95795100	6.30829400
C	-0.68857100	0.32442000	8.09641600
H	0.36054300	0.61394000	8.09579800
H	-0.82853500	-0.39034700	8.90702900
H	-0.91193100	-0.17551500	7.15209700

int-11
G +ZPE = -1116.893169 a.u

C	2.06673100	4.73741700	12.30326300
C	-1.38665900	4.07597900	13.64661000
C	2.07264900	4.02023400	11.10740200
C	3.16187200	5.54586400	12.62905300
H	-1.08513200	3.45617900	14.50731000
H	-2.27723900	3.61362600	13.22111300
C	-1.78663800	5.45401800	14.14217500
C	3.17830600	4.11418100	10.25188700
C	4.25458100	5.63905900	11.77503500
H	3.15299500	6.10472300	13.55929800
C	-1.79021100	5.74615500	15.50815700
C	-2.20210600	6.44138800	13.24067400
H	3.19015900	3.54632000	9.32690700
C	4.26424700	4.91730500	10.57964800
H	5.09535400	6.27095200	12.03736100
H	-1.46704400	4.99110200	16.21744100
C	-2.20199500	6.99743300	15.96909000
C	-2.60994700	7.69181400	13.69724700
H	-2.20072400	6.22668400	12.17797500
H	5.11437900	4.97943500	9.91022500
H	-2.19749900	7.20786200	17.03272000
C	-2.61203300	7.97385400	15.06450000
H	-2.92968800	8.44666200	12.98740700
H	-2.93018900	8.94753000	15.41954400
C	-0.07014800	2.86421100	11.89815300
C	0.90088400	3.13685000	10.73598300
H	1.29551200	2.17157200	10.40091600
C	0.10611400	3.72497400	9.56030900
H	0.76080200	4.05814300	8.75966700
H	-0.48907200	4.57855000	9.89842000
N	-0.77325600	2.67861500	9.01248100
C	-0.86888200	2.57263200	7.53712800
H	0.09532400	2.89308000	7.14464700
H	-0.98753900	1.52657300	7.26353200
C	-1.99782900	3.43182700	6.96925300

H	-2.97046900	3.10957800	7.34647300
H	-1.85798500	4.48499000	7.22028400
H	-2.00348900	3.33834000	5.88140000
C	-1.42007700	1.89667400	9.85060600
C	-1.39999500	2.24384400	11.32639000
C	-2.22088300	0.79246900	9.41285800
C	-1.96696100	1.17902000	12.21365600
H	-2.13225000	3.07196100	11.34535600
C	-2.78583500	-0.05183100	10.31691300
H	-2.34929700	0.60085500	8.35893600
C	-2.63092100	0.11352600	11.73759300
H	-1.84383900	1.31724500	13.28020400
H	-3.36048300	-0.89507700	9.95159600
H	-3.05258600	-0.62571200	12.40607300
H	0.38206800	2.11314600	12.56390400
N	-0.32468600	4.12498500	12.62398200
C	0.91160500	4.60424000	13.25848800
H	0.70223900	5.57364700	13.71292300
H	1.20479400	3.92897300	14.08512900

D_1

G +ZPE = -1116.507594 a.u

C	2.21594500	4.66784200	12.32363000
C	-1.31919900	3.90791500	13.40918700
C	2.33585500	3.89575600	11.16322000
C	3.27033400	5.50545500	12.70569100
H	-1.07721500	3.38199000	14.35010700
H	-2.09974600	3.32740700	12.91634500
C	-1.87477800	5.28054600	13.74209200
C	3.51246200	3.97306900	10.40712000
C	4.43411500	5.57955300	11.94758400
H	3.17458200	6.10405100	13.60647300
C	-2.07172800	5.67126700	15.06912200
C	-2.24022700	6.16821900	12.72197900
H	3.60829900	3.36636200	9.51182500
C	4.55750000	4.80660300	10.79156500
H	5.24112100	6.23512400	12.25509500
H	-1.78874300	4.99662700	15.87090500
C	-2.62433600	6.91599700	15.37560600
C	-2.78890600	7.41186200	13.02339400
H	-2.08701600	5.87808900	11.68871100
H	5.46282500	4.85300100	10.19681000
H	-2.76814800	7.20146100	16.41191300

C	-2.98421000	7.79058100	14.35311100
H	-3.06762900	8.08672700	12.22134100
H	-3.41146200	8.75914700	14.58750200
C	0.24011900	2.67327200	11.89196400
C	1.19680600	2.99912600	10.73145100
H	1.60910400	2.05555900	10.35715800
C	0.39243300	3.65116200	9.59778100
H	1.05289000	3.85219600	8.75099000
H	0.00533900	4.61384600	9.95513300
N	-0.68100700	2.78019500	9.12893600
C	-1.21477400	3.06784900	7.79960100
H	-0.38000700	3.43054900	7.19506800
H	-1.54213900	2.13640300	7.33114900
C	-2.34936900	4.10051800	7.77741600
H	-3.20945900	3.75864000	8.35793500
H	-2.01671500	5.05573100	8.19154500
H	-2.68109700	4.27435300	6.74985800
C	-1.34341500	1.95400400	10.01874300
C	-0.90678200	1.83391800	11.36721200
C	-2.44087100	1.15807700	9.61072100
C	-1.50454000	0.88451000	12.19805300
C	-3.03343500	0.24655100	10.47482100
H	-2.82935500	1.24359500	8.60572700
C	-2.56460300	0.08629500	11.77784100
H	-1.12704100	0.78164200	13.21090400
H	-3.86829500	-0.34753300	10.11812900
H	-3.01478200	-0.63483800	12.44930500
H	0.78055600	2.06640000	12.63911100
N	-0.16503700	3.96559600	12.50707100
C	0.98182900	4.56836700	13.19008800
H	0.69737400	5.56664300	13.53065400
H	1.23630700	3.99059800	14.10194000

TS15

G +ZPE = -1103.213819 a.u

N	-1.33849200	-0.14475300	-1.46157800
C	-0.37389100	0.59367800	-1.99856100
C	-2.73937000	0.07290400	-1.88786900
C	0.98800800	0.46099100	-1.68059500
H	-0.70509200	1.36751000	-2.68118400
C	-1.01926200	-1.16041700	-0.49980200
C	-3.62032800	0.81919100	-0.90075500
H	-2.68816100	0.62403400	-2.82799900

H	-3.16975300	-0.90201200	-2.11919100
C	1.42159600	-0.87370800	-1.12625100
H	1.65088600	0.91129700	-2.41161800
C	0.32853300	-1.50711700	-0.29628000
C	-2.03189500	-1.79135900	0.22890900
C	-4.96843700	0.46179900	-0.79877200
C	-3.14565300	1.89641000	-0.14875100
H	1.69760300	-1.55020200	-1.94565600
H	2.32608100	-0.74214500	-0.52600200
C	0.62455900	-2.48264400	0.65469700
C	-1.70923000	-2.76821200	1.16556900
H	-3.06747400	-1.51915400	0.08860000
C	-5.82810800	1.16950100	0.03885800
H	-5.34887700	-0.37363500	-1.37741200
C	-4.00320800	2.60031300	0.69456400
H	-2.10569800	2.19414700	-0.21427100
C	-0.37908300	-3.11446100	1.38481900
H	1.66249000	-2.75203000	0.81691700
H	-2.50189500	-3.24908200	1.72554900
C	-5.34663600	2.24029700	0.78975500
H	-6.87043500	0.88025400	0.10727100
H	-3.62108900	3.43240100	1.27461800
H	-0.12351800	-3.87057700	2.11739400
H	-6.01251900	2.78917100	1.44543500
O	4.37761100	-0.70467500	2.45370100
C	1.32366200	1.81335400	-0.23181900
C	4.69336600	-0.95780700	3.85109500
Mg	3.61950300	0.96293500	1.59000900
H	4.71584000	-1.41831400	1.90015100
O	2.55116700	1.64135600	0.15972400
H	1.12343000	2.71003400	-0.82202300
H	0.52022000	1.54690200	0.46223700
H	4.23863500	-1.89805900	4.15954700
H	5.77407600	-0.98718300	3.98438900
H	4.26932300	-0.13578700	4.42193000

int-12
G +ZPE = -1103.225791 a.u

N	-1.37396900	0.03791900	-1.29304000
C	-0.39341800	0.76740800	-1.73652300
C	-2.75536600	0.29175800	-1.79820400
C	1.00290900	0.59933400	-1.30646900
H	-0.65855100	1.55344500	-2.43460200

C	-1.13750000	-1.03754300	-0.35553400
C	-3.66494700	1.02910800	-0.83309300
H	-2.63550700	0.86216600	-2.71945400
H	-3.17812400	-0.67508600	-2.06834300
C	1.29727800	-0.86593000	-0.98042100
H	1.64447700	0.94997800	-2.11715300
C	0.18194000	-1.48310700	-0.17648400
C	-2.19969200	-1.61870200	0.33788700
C	-5.01367000	0.66360200	-0.77615600
C	-3.21747100	2.10546500	-0.06288300
H	1.42999900	-1.43516500	-1.90768400
H	2.24110600	-0.92005700	-0.43569200
C	0.40496700	-2.52623100	0.71937500
C	-1.94873100	-2.66024400	1.22549100
H	-3.21089000	-1.26156600	0.21313500
C	-5.90158900	1.36352500	0.03790900
H	-5.37228900	-0.16926400	-1.37158500
C	-4.10432600	2.79908400	0.75790800
H	-2.17857000	2.41234900	-0.09278000
C	-0.64725000	-3.11512300	1.41751500
H	1.41870700	-2.88172600	0.86482400
H	-2.77316800	-3.10787300	1.76607700
C	-5.44795200	2.43150100	0.80955900
H	-6.94425000	1.07004100	0.07161100
H	-3.74422800	3.62927100	1.35419800
H	-0.44996300	-3.92516500	2.10926400
H	-6.13609200	2.97319400	1.44787000
O	4.66405500	-0.67971700	2.35806600
C	1.32565900	1.57149900	-0.08505500
C	5.23713200	-0.88534800	3.67782100
Mg	3.89102500	0.98751700	1.49990700
H	4.76868400	-1.47640400	1.82501600
O	2.65989000	1.51052400	0.20911600
H	1.01963900	2.58262100	-0.39346100
H	0.67463200	1.27595500	0.75376300
H	4.72217100	-1.70753600	4.17332400
H	6.30247900	-1.09461300	3.58740500
H	5.08537300	0.03559700	4.23428700

TS16

G +ZPE = -1103.21612 a.u

N	-1.38109600	0.38851300	-1.18637800
C	-0.51770800	1.30597400	-1.49269400

C	-2.76516100	0.47915700	-1.74696900
C	0.87639100	1.31069900	-0.98485800
H	-0.86863800	2.10505000	-2.13538100
C	-1.02272900	-0.72918400	-0.34000200
C	-3.81350500	0.96120200	-0.76171000
H	-2.69744400	1.15635700	-2.59823900
H	-3.01560100	-0.50727900	-2.13401900
C	1.37852100	-0.12321600	-0.79282900
H	1.48980700	1.82652800	-1.72739000
C	0.34055200	-0.98243500	-0.12057800
C	-2.01118300	-1.53684700	0.22444700
C	-5.07209500	0.35173400	-0.77133400
C	-3.58379800	2.03805000	0.09874800
H	1.62639700	-0.55617200	-1.76854600
H	2.29791700	-0.09860600	-0.20796400
C	0.68556900	-2.06608800	0.68328400
C	-1.63873300	-2.61381500	1.02168600
H	-3.05934100	-1.33031100	0.07063600
C	-6.08547200	0.81077500	0.06744700
H	-5.26219800	-0.48354700	-1.43689400
C	-4.59491800	2.49021600	0.94372500
H	-2.62013700	2.53322900	0.12098000
C	-0.29163800	-2.87968500	1.25207400
H	1.73475200	-2.27340100	0.85923900
H	-2.40547000	-3.23805600	1.46273400
C	-5.84769400	1.87892400	0.92994800
H	-7.05622000	0.32950100	0.04944500
H	-4.40311300	3.32274100	1.61043300
H	-0.00092200	-3.71763700	1.87398900
H	-6.63268800	2.23265500	1.58798300
C	0.94655000	2.16604800	0.32638700
H	0.62690100	3.18860500	0.09785900
H	0.26159300	1.75242900	1.07164400
O	2.25267200	2.13626700	0.84637600
H	3.06084700	2.84431000	0.41778800
Mg	3.15011100	3.09458000	2.44478600
O	3.97472800	3.67957100	0.75739600
C	5.25978800	3.69635600	0.16693400
H	5.66712900	2.68433900	0.04237400
H	5.22224000	4.17090300	-0.82087300
H	5.95896400	4.26837100	0.78619200

int-13

G +ZPE = -1103.21302 a.u

N	-0.55290900	0.39814700	-0.57991900
C	0.58100000	1.02765200	-0.54800100
C	-1.71441100	1.02400900	-1.28356400
C	1.78568900	0.47701100	0.09700400
H	0.61475300	1.98284200	-1.06043800
C	-0.69539600	-0.90652900	0.02813500
C	-2.85332300	1.38477600	-0.35332300
H	-1.31910900	1.91005300	-1.77945100
H	-2.03944500	0.33849700	-2.06446600
C	1.40838700	-0.38557200	1.30692400
H	2.27503600	-0.16492300	-0.68453000
C	0.27927500	-1.31814100	0.95013900
C	-1.76623100	-1.73358700	-0.31086800
C	-4.15990600	1.02183900	-0.68925200
C	-2.62825400	2.13577500	0.80492900
H	2.27728000	-0.95143400	1.64088700
H	1.10142800	0.25892900	2.14105800
C	0.14753700	-2.57712800	1.52967700
C	-1.87337600	-2.98873600	0.27948600
H	-2.51250800	-1.42448400	-1.02652900
C	-5.22834800	1.40169300	0.12137500
H	-4.34741300	0.44612500	-1.58931400
C	-3.69482800	2.50723100	1.61905400
H	-1.62221800	2.43599900	1.07737000
C	-0.91978900	-3.41048000	1.20149700
H	0.89360300	-2.90634500	2.24369600
H	-2.70305000	-3.63145700	0.01367200
C	-4.99717400	2.14134600	1.27891300
H	-6.23747800	1.11523700	-0.15055700
H	-3.50942800	3.08642600	2.51602700
H	-1.00266700	-4.38750800	1.66183200
H	-5.82635300	2.43265200	1.91302500
C	2.80577400	1.57307200	0.41219100
H	2.93937300	2.24388600	-0.43851400
H	2.51172800	2.15695700	1.28491300
O	4.08117700	0.93693500	0.70268400
H	4.50920700	1.37313700	1.44784400
Mg	5.17217400	-0.18908300	-0.65161300
O	3.75273900	-0.85992300	-1.66683400
C	3.56636500	-1.78462700	-2.70105900
H	4.10742900	-1.50793800	-3.61997400
H	3.89338800	-2.80103200	-2.42902600
H	2.50146600	-1.85599500	-2.97128400

TS17

G +ZPE = -1103.221511 a.u

N	-1.34943800	1.39629600	-1.60855200
C	-0.20328000	2.04671100	-1.55880100
C	-2.49005200	1.99274700	-2.34452100
C	0.96872400	1.56838400	-0.91382600
H	-0.17758600	2.98454600	-2.10311600
C	-1.47137000	0.09099700	-1.01456300
C	-3.64421400	2.39724700	-1.44649000
H	-2.09268500	2.86375300	-2.86648300
H	-2.81827000	1.29065000	-3.11095000
C	0.70872800	0.54235300	0.18973900
H	1.60437000	0.95927900	-1.85487800
C	-0.44842100	-0.36189400	-0.16606400
C	-2.56969700	-0.72141800	-1.30365800
C	-4.95700800	2.15348200	-1.85787700
C	-3.42270100	3.06860300	-0.24015400
H	1.60337100	-0.05579600	0.36831200
H	0.48608900	1.06193300	1.13266200
C	-0.55424900	-1.64193900	0.37373100
C	-2.65453400	-1.99473400	-0.74756000
H	-3.36217100	-0.38061200	-1.95282500
C	-6.03290300	2.57523300	-1.07801500
H	-5.14200300	1.63336000	-2.79187400
C	-4.49703000	3.48379800	0.54280800
H	-2.41129800	3.26774900	0.09601500
C	-1.64638700	-2.45931000	0.09095000
H	0.23376500	-1.99887800	1.02759600
H	-3.51023600	-2.61778600	-0.97664400
C	-5.80512100	3.23932500	0.12542400
H	-7.04620100	2.37896200	-1.40877700
H	-4.31213600	4.00011100	1.47774000
H	-1.70729600	-3.45108500	0.52252900
H	-6.64027400	3.56278800	0.73581300
C	1.94821800	2.66994800	-0.54745300
H	1.95106300	3.46810400	-1.29214300
H	1.72556400	3.09317600	0.43428900
O	3.29905200	2.11011200	-0.46664800
H	3.81867000	2.61912100	0.16609800
Mg	4.23162100	0.85716600	-1.79037200
O	2.56963300	0.38585400	-2.62013400
C	2.11605800	-0.65527600	-3.46893000

H	2.62323300	-0.61326100	-4.43877400
H	2.28837600	-1.64493000	-3.03011100
H	1.04031200	-0.54789700	-3.65133700

int-14

G +ZPE = -1103.234934 a.u

N	-1.40265500	1.18159300	-1.23499900
C	-0.20779600	1.85444700	-1.11041700
C	-2.40754600	1.68063800	-2.17476700
C	0.79582500	1.49269500	-0.28034800
H	-0.12113300	2.74027800	-1.72917000
C	-1.55910300	-0.07053800	-0.59767500
C	-3.68844900	2.19192600	-1.53181800
H	-1.93989100	2.49405400	-2.73342100
H	-2.64574600	0.90610400	-2.90978400
C	0.62356200	0.33345400	0.66830500
H	1.46280100	0.67779500	-2.33563000
C	-0.56694500	-0.52937500	0.29408400
C	-2.67649400	-0.87779000	-0.85876800
C	-4.89328600	2.10261000	-2.23540800
C	-3.68392900	2.79992400	-0.27383100
H	1.52971900	-0.28100400	0.69515800
H	0.49952700	0.71697400	1.69348100
C	-0.71377800	-1.79071000	0.86998400
C	-2.80337700	-2.12770800	-0.26013400
H	-3.45706900	-0.53484900	-1.52245700
C	-6.07117200	2.61737600	-1.69655100
H	-4.91177000	1.62492900	-3.21001700
C	-4.86176900	3.31106400	0.26882800
H	-2.75918600	2.86853900	0.28758100
C	-1.81878400	-2.59611400	0.60444100
H	0.05622000	-2.14168200	1.54967000
H	-3.67690000	-2.73177500	-0.47648900
C	-6.05857700	3.22346900	-0.44116000
H	-6.99789100	2.53822300	-2.25366200
H	-4.84413700	3.77746400	1.24749000
H	-1.90988900	-3.57025300	1.06987000
H	-6.97438300	3.61955500	-0.01752600
C	1.99573900	2.35962600	-0.13892000
H	1.96523900	3.21938100	-0.80691300
H	2.12516800	2.71295300	0.88647800
O	3.24752700	1.61822600	-0.45783100
H	3.77232100	1.49431200	0.34097100

Mg	4.01158600	1.09757200	-2.26232200
O	2.20331400	0.56061700	-2.95966800
C	1.73885000	0.14076500	-4.26764100
H	1.08858000	0.90539700	-4.69199300
H	2.61928200	0.01449400	-4.89285600
H	1.20955800	-0.80746700	-4.17945600

int-15

G +ZPE = -711.662522 a.u

N	-1.62998900	0.59352100	-1.53496300
C	-0.50704900	1.23706600	-1.70994800
C	-2.78874100	0.94814200	-2.40081800
C	0.68404700	1.00066700	-0.94853300
H	-0.50190400	2.00469600	-2.47371100
C	-1.77353100	-0.40963600	-0.51015600
C	-3.85001800	1.78332800	-1.70762500
H	-2.37352600	1.48620700	-3.25304700
H	-3.20770100	0.01904900	-2.78489100
C	0.71806700	-0.19506000	-0.03002400
C	-0.65053500	-0.77206700	0.24868200
C	-3.01862900	-1.00037500	-0.27707000
C	-5.19023300	1.58832200	-2.05503200
C	-3.52134200	2.78448800	-0.79006100
H	1.33332500	-0.97380200	-0.49622300
H	1.21306200	0.06302100	0.90832100
C	-0.81277600	-1.72683700	1.25012100
C	-3.15201400	-1.95598000	0.72306700
H	-3.88787700	-0.71801300	-0.85055900
C	-6.18705100	2.38506300	-1.49608900
H	-5.45713200	0.81336100	-2.76576100
C	-4.52013400	3.57482200	-0.22506400
H	-2.48970100	2.95500600	-0.50428000
C	-2.04945100	-2.31852700	1.49152900
H	0.04834800	-2.01296300	1.84327500
H	-4.11954200	-2.40837800	0.90003300
C	-5.85433600	3.37880800	-0.57740900
H	-7.22196500	2.22395200	-1.77445600
H	-4.25339400	4.34464300	0.48954500
H	-2.14975600	-3.05937500	2.27531600
H	-6.62947500	3.99490200	-0.13693500
C	1.70582700	1.86041900	-1.12259800
H	1.64119700	2.67667700	-1.83180800
H	2.62512700	1.76621100	-0.55708300

TS18

G +ZPE = -1655.601004 a.u

N	-1.64301700	-0.60719300	-1.26125800
C	-0.61420000	-0.01489600	-1.84598500
C	-3.01021100	-0.25048600	-1.70184800
C	0.73244100	-0.26040000	-1.52583000
H	-0.85576900	0.72419100	-2.59853400
C	-1.44498700	-1.56146200	-0.20905400
C	-3.76943900	0.65321900	-0.74529500
H	-2.90071800	0.23729300	-2.67110400
H	-3.55997400	-1.17532900	-1.87870100
C	1.06659500	-1.41113000	-0.61332800
C	-0.13693400	-1.93804500	0.13662300
C	-2.54230400	-2.10210300	0.46901800
C	-5.15790200	0.51943600	-0.64915600
C	-3.13017300	1.65492000	-0.01045400
H	1.48788500	-2.22394500	-1.22129700
H	1.84984800	-1.12639000	0.09388300
C	0.03562700	-2.85214600	1.17465400
C	-2.34300900	-3.01933400	1.49482700
H	-3.55019400	-1.80745900	0.21857100
C	-5.89651500	1.37616400	0.16435300
H	-5.66490900	-0.25726100	-1.21219400
C	-3.86824600	2.50675200	0.80923800
H	-2.05443800	1.77475300	-0.06725700
C	-1.05178900	-3.39531000	1.85318700
H	1.04340000	-3.14561400	1.44752500
H	-3.20023400	-3.43087400	2.01318000
C	-5.25273500	2.37148100	0.89759700
H	-6.97227000	1.26075100	0.22936800
H	-3.35968000	3.27665500	1.37796300
H	-0.89051300	-4.10600500	2.65469700
H	-5.82512300	3.03471900	1.53566000
C	1.68932300	0.49743700	-2.15140300
H	1.40692300	1.33477100	-2.77629600
H	2.72052200	0.46412000	-1.82292500
H	2.24037000	-0.38189200	-3.58860900
C	2.40579300	0.01870600	-6.74666900
C	2.56767500	-1.26735900	-7.37929700
C	3.59799400	-2.07414700	-6.90541800
C	4.46025000	-1.61424100	-5.85487600
C	4.45690200	-0.27353500	-5.39283200

C	3.38429400	0.54236100	-5.85320300
H	1.56287500	0.64047000	-7.02125500
H	3.69886800	-3.09180900	-7.26074500
H	5.17869200	-2.31228100	-5.44574500
H	3.26288400	1.54529200	-5.46446000
C	5.50718000	0.28490700	-4.45147600
H	5.03797300	1.11657300	-3.91608500
C	6.00967200	-0.72023000	-3.40730700
H	6.67716700	-0.21584100	-2.70481100
H	5.18220500	-1.15268100	-2.83983000
H	6.57662900	-1.53559400	-3.86504800
C	6.67363700	0.85769300	-5.28166500
H	6.32651200	1.60983500	-5.99430600
H	7.40800200	1.32766000	-4.62266900
H	7.17782700	0.06515200	-5.84227800
C	1.60170300	-1.71965900	-8.43261400
H	0.57283200	-1.50194000	-8.13833800
H	1.79717800	-1.18210900	-9.36609200
H	1.69413000	-2.78842900	-8.62617800
Ru	2.45949800	-1.20954300	-4.98271800
Cl	0.15114200	-1.88286400	-4.65071700

int-16

G +ZPE = -712.42951 a.u

N	-1.69003700	0.74896400	-1.50956900
C	-0.44133600	1.38082400	-1.56972700
C	-2.77370200	1.25227000	-2.34132300
H	-0.41533200	2.24149400	-2.22721000
C	-1.84390500	-0.40152600	-0.72990600
C	-3.86733500	2.01791200	-1.60587800
H	-2.32303300	1.91269300	-3.08634100
H	-3.22273000	0.42918000	-2.90659900
C	0.59755800	-0.19802900	0.02447900
C	-0.74975900	-0.89885900	0.01616100
C	-3.07299700	-1.08529100	-0.67416800
C	-5.14367500	2.10649000	-2.17130900
C	-3.61760400	2.67792000	-0.40037100
H	1.38864900	-0.91125000	-0.25157500
H	0.84953000	0.11371200	1.05038400
C	-0.92220500	-2.06158100	0.76655400
C	-3.21375300	-2.24188500	0.08575400
H	-3.92946700	-0.70911700	-1.21605500
C	-6.14826800	2.84595100	-1.55009800

H	-5.35375900	1.59088200	-3.10349300
C	-4.62229600	3.41565600	0.22512300
H	-2.63569800	2.60989500	0.05351900
C	-2.13696100	-2.74296500	0.81222600
H	-0.07545700	-2.43910000	1.33232400
H	-4.17348100	-2.74622700	0.10835400
C	-5.89004500	3.50404100	-0.34785600
H	-7.13289000	2.90272400	-2.00084400
H	-4.41387700	3.92061000	1.16193600
H	-2.23962500	-3.64330200	1.40652000
H	-6.67115600	4.07619100	0.13978700
C	1.95547100	1.72175500	-1.00866700
H	2.28653200	2.09604800	-0.03131900
H	2.74909900	1.05518500	-1.36980900
H	1.89091500	2.57184000	-1.69168300
C	0.64712600	0.99445500	-0.89505600

TS19

G +ZPE = -1116.900602 a.u

N	-1.78125100	0.16510000	1.38393800
C	-1.46105400	1.43936400	0.82621000
C	-3.17733100	-0.30760100	1.42389300
C	-0.15545900	1.94275500	0.97202500
C	-2.42694900	2.17934600	0.13475400
H	-3.79366100	0.49313700	1.83706300
H	-3.20439600	-1.12360000	2.14797600
C	-3.76409300	-0.78268400	0.10339200
C	0.14884900	3.17775500	0.40098500
C	-2.09975300	3.41492800	-0.41509100
H	-3.42851400	1.79580000	0.00701700
C	-5.13338000	-0.60816800	-0.12269900
C	-3.00090300	-1.44515300	-0.85994200
H	1.15623600	3.56538500	0.50861700
C	-0.80816300	3.91758600	-0.28915000
H	-2.85815800	3.97551700	-0.94825300
H	-5.73827700	-0.09116400	0.61523400
C	-5.72859700	-1.09058800	-1.28647100
C	-3.59287100	-1.92249100	-2.02801800
H	-1.93801700	-1.58827900	-0.70916000
H	-0.54655600	4.87523000	-0.72292300
H	-6.79091600	-0.94615000	-1.44656700
C	-4.95874900	-1.74873300	-2.24422600
H	-2.98533000	-2.43094700	-2.76791000

H	-5.41852400	-2.11982300	-3.15284900
C	0.52821300	-0.26242500	1.98520800
C	0.88531300	1.19513000	1.77700800
H	1.86582800	1.29327800	1.30367400
H	0.98993400	1.66940700	2.76475400
C	0.98465400	-1.19837200	0.02714400
H	0.58455000	-0.34368900	-0.49936900
H	0.34713400	-2.06231100	0.14305300
N	2.29172200	-1.44174900	-0.10408600
C	2.80703200	-2.82908700	0.06943000
H	2.02223100	-3.38755100	0.57921400
H	2.91924300	-3.25323600	-0.93382200
C	4.12062000	-2.95726700	0.83004900
H	4.94093300	-2.45249900	0.31845100
H	4.04490500	-2.56691800	1.84643100
H	4.37305000	-4.01828600	0.89434100
C	3.14053700	-0.47144100	-0.76101300
C	4.15049300	0.19091200	-0.06217800
C	2.94233400	-0.21982100	-2.12036500
C	4.95691100	1.11229600	-0.72747300
H	4.29861200	-0.00078600	0.99259100
C	3.74984100	0.70577200	-2.77714300
H	2.16398600	-0.74935300	-2.65722200
C	4.75804900	1.37177600	-2.08261800
H	5.73665200	1.63051300	-0.18226900
H	3.59160900	0.90141100	-3.83100600
H	5.38637300	2.09062400	-2.59498200
C	-0.81647900	-0.59241600	1.93836400
H	-1.15113500	-1.55139900	2.31751700
C	1.42678900	-1.04022000	2.91148400
H	1.08421300	-2.06786600	3.05115800
H	2.44877800	-1.06955600	2.52506500
H	1.47080700	-0.55726000	3.89386100

int-17

G +ZPE = -1116.918547 a.u

N	-0.19856200	1.56276200	11.89307600
C	0.53645800	2.00548600	13.05638300
C	-1.15146900	0.41551900	12.01242400
C	1.21366800	3.23166700	12.98129900
C	0.58818900	1.21579300	14.20628800
H	-0.56840800	-0.46075200	12.29650300
H	-1.52876800	0.23783700	11.00581900

C	-2.29234600	0.64620600	12.98075000
C	1.93408500	3.65271900	14.09729400
C	1.31647800	1.65795800	15.30496500
H	0.07838500	0.26610400	14.25999100
C	-2.72363700	-0.41660600	13.77968400
C	-2.96726700	1.86765600	13.04613800
H	2.46509500	4.59650900	14.05198600
C	1.98495300	2.87871700	15.25383400
H	1.35895100	1.04460900	16.19611200
H	-2.20679300	-1.36944900	13.73717100
C	-3.81639800	-0.26187800	14.63031100
C	-4.05239100	2.02509400	13.90530700
H	-2.65296800	2.69996000	12.42829300
H	2.55166900	3.22452700	16.10988200
H	-4.14167700	-1.09414400	15.24347300
C	-4.48113000	0.96095200	14.69757400
H	-4.56469000	2.97900000	13.95204800
H	-5.32676000	1.08494100	15.36395800
C	1.02247500	3.15943200	10.47501300
C	1.13667900	4.04988700	11.72164500
H	0.26475600	4.71242000	11.75295600
H	2.01740800	4.68941700	11.63256000
C	0.68521000	3.99545900	9.20720800
H	0.61286500	3.30755200	8.35279200
H	1.56035700	4.62036400	9.02237300
N	-0.46941600	4.88050800	9.33908300
C	-0.31863400	6.13816200	8.56779600
H	0.69304500	6.50317100	8.75887400
H	-0.39311100	5.94731400	7.48500600
C	-1.31621600	7.22106400	8.96522600
H	-2.34558100	6.93067500	8.74635100
H	-1.24197400	7.44655000	10.03210900
H	-1.10092800	8.13442900	8.40451700
C	-1.75047800	4.24366100	9.14680200
C	-2.75448600	4.44184200	10.10011400
C	-2.03776400	3.46824100	8.01410600
C	-4.01848400	3.87741300	9.92917500
H	-2.53404600	5.04823800	10.97060800
C	-3.29692600	2.89482100	7.85050700
H	-1.28215400	3.31745000	7.25157900
C	-4.29176400	3.09816900	8.80696100
H	-4.78629900	4.04316100	10.67608900
H	-3.50372200	2.29675900	6.97014400
H	-5.27184700	2.65428000	8.67628800

C	0.00754400	2.09343700	10.72693000
H	-0.54991800	1.66994500	9.89912000
C	2.35302900	2.40230500	10.18152200
H	2.25846300	1.76199200	9.30239700
H	3.13380300	3.14142400	9.98845600
H	2.66339100	1.78755100	11.02719500

TS20

G +ZPE = -1116.892376 a.u

N	-0.81458000	-0.69271700	-0.85890400
C	0.42877100	-0.19082600	-1.18591200
C	-2.02194500	-0.02188000	-1.37442700
C	1.62773700	-1.14851400	-1.22179200
H	0.38183000	0.49443600	-2.02320000
C	-0.96025400	-1.96399900	-0.21994400
C	-2.82699600	0.87585000	-0.43999100
H	-1.71709200	0.56568500	-2.24286600
H	-2.68109300	-0.80912600	-1.75227900
C	1.10562500	-2.58410600	-1.44194100
C	2.43433300	-1.13954500	0.12194400
C	0.31424600	2.40696600	-0.05306600
C	2.49212700	1.24769500	0.13837600
C	0.01853900	-2.93909300	-0.46983200
C	-2.07220500	-2.28226300	0.56440000
C	-3.85364400	1.63466700	-1.01718600
C	-2.62125400	0.97088600	0.93765100
H	0.70482400	-2.65068000	-2.46064200
H	1.93543300	-3.29142800	-1.38337000
N	3.17915700	0.10116300	0.28246700
H	3.14626600	-1.96180700	0.11263500
H	1.76016600	-1.30469600	0.96656400
C	0.94958600	3.57682800	-0.34403600
H	-0.76371500	2.38262600	0.02245200
C	3.12625700	2.50032200	-0.09005300
C	-0.09590500	-4.19234300	0.12324800
C	-2.18339400	-3.55102900	1.13256400
H	-2.85327500	-1.55641900	0.73802000
C	-4.65478800	2.46244400	-0.23696400
H	-4.02558900	1.57719400	-2.08740200
C	-3.42270900	1.80265400	1.72231700
H	-1.83167500	0.40228400	1.41314800
C	4.65140100	0.05802700	0.37727600
C	2.36939900	3.61592300	-0.34412800

H	0.38621100	4.48180900	-0.53229500
H	4.20310600	2.57957400	-0.08076900
C	-1.19085500	-4.50411300	0.92977000
H	0.66809500	-4.93787400	-0.06877600
H	-3.04945100	-3.78371200	1.74077900
C	-4.44232100	2.54907800	1.13955900
H	-5.44298900	3.04220500	-0.70363300
H	-3.24415600	1.86315900	2.78976000
C	5.16244900	0.39582300	1.77850100
H	5.08167700	0.72842700	-0.36951500
H	4.95774800	-0.95015500	0.10319500
H	2.87301400	4.55465300	-0.54519400
H	-1.27151900	-5.48521600	1.38228000
H	-5.06474300	3.19396900	1.74888300
H	6.25303600	0.33226600	1.79287500
H	4.87697500	1.40627300	2.07798500
H	4.76710200	-0.30661800	2.51522800
C	1.03828800	1.17738100	0.15514600
H	0.64139300	0.52807700	0.93002600
C	2.54243100	-0.75656900	-2.39567000
H	3.42750200	-1.39708600	-2.41716700
H	2.01167700	-0.88441800	-3.34155800
H	2.87468800	0.28118000	-2.33922600

int-18

G +ZPE = -1116.901008 a.u

N	2.03856600	4.77884100	11.60617400
C	2.48892500	3.89280100	12.69511900
C	0.59056700	5.00859200	11.56145400
C	4.01887400	3.74353900	12.75915700
H	2.15279600	4.30548000	13.65477600
C	2.87832600	5.76034300	11.05228900
C	-0.18080900	4.26461300	10.47672800
H	0.16520100	4.74993200	12.53624600
H	0.41040500	6.08209500	11.45089500
C	4.66763900	5.13702100	12.70150300
C	4.47785000	2.92637200	11.54315100
C	0.87039000	2.10264700	13.63321500
C	2.70901800	1.34313400	12.08069300
C	4.19245000	5.94736400	11.52630100
C	2.43322400	6.56033200	9.98148300
C	-1.57575700	4.18169600	10.57467200
C	0.45069800	3.68588500	9.37303800

H	4.42464900	5.65676000	13.63601500
H	5.75656100	5.03685300	12.68120500
N	3.86732500	1.58141900	11.50635600
H	5.55713900	2.77905400	11.58307700
H	4.24737200	3.44467700	10.60911800
C	0.52671400	0.82254000	13.85780600
H	0.39651000	2.91221800	14.17526200
C	2.18047100	0.01528000	12.22715400
C	5.01721100	6.89585300	10.92204100
C	3.26599800	7.51659500	9.40946200
H	1.43985100	6.42396900	9.57541800
C	-2.32080800	3.53902200	9.59047800
H	-2.08031800	4.62152300	11.42938900
C	-0.29545700	3.03840900	8.38607000
H	1.52921900	3.74126200	9.28528500
C	4.64085900	0.53125800	10.79436100
C	1.15145300	-0.21905900	13.08738300
H	-0.21636300	0.56236100	14.60024800
H	2.65173500	-0.81744300	11.72868900
C	4.56841700	7.69184900	9.87137800
H	6.02828200	7.01651000	11.29839900
H	2.89190400	8.11621200	8.58705400
C	-1.68170100	2.96391800	8.49049800
H	-3.39953200	3.48165900	9.68316500
H	0.21065200	2.59478400	7.53600700
C	4.14651300	0.31149700	9.36544600
H	4.60567100	-0.38772000	11.37782200
H	5.67707200	0.86366800	10.79243400
H	0.81534000	-1.24029900	13.22529600
H	5.22261900	8.42846300	9.42061900
H	-2.26114200	2.46081400	7.72493200
H	4.76767200	-0.44891600	8.88779400
H	3.11069400	-0.03287600	9.34631800
H	4.21845700	1.22902900	8.77850000
C	1.82862500	2.47890700	12.54548300
H	1.16640700	2.55280700	11.65826600
C	4.43080000	3.06183600	14.07733600
H	5.51745700	2.95401600	14.11617300
H	4.12270800	3.67457400	14.92727600
H	3.99705600	2.06921400	14.21192400

C_1'

G +ZPE = -1116.510123 a.u

N	2.05037300	4.56350500	11.73931900
C	2.65474700	3.82904400	12.88345600
C	0.58892800	4.61498300	11.69962400
C	4.18001500	3.66263200	12.69350800
C	2.06166400	2.44749500	13.05599900
H	2.48350800	4.39810600	13.80914700
C	2.74568400	5.62883100	11.15321600
C	-0.06358600	4.06626900	10.43694300
H	0.21345900	4.03620200	12.54335700
H	0.24443300	5.64419400	11.86098500
C	4.81570700	5.04582400	12.49768800
C	4.42993400	2.80275900	11.44129400
C	1.11047700	2.18821200	14.03892700
C	2.55862300	1.38593400	12.25401600
C	4.10168200	5.88464200	11.46730600
C	2.11991200	6.46716400	10.20437100
C	-1.34910900	4.49649700	10.08851500
C	0.56254900	3.10564800	9.63937000
H	4.81087800	5.56322700	13.46518500
H	5.86946600	4.93320800	12.22112400
N	3.53201300	1.64524500	11.30190800
H	5.46583700	2.44080500	11.48221100
H	4.34798900	3.42701600	10.54758000
C	0.57824200	0.91370000	14.23307800
H	0.77720600	3.01022600	14.66550800
C	2.02672000	0.09596700	12.47043000
C	4.77111300	6.93129500	10.83376800
C	2.80808700	7.51248700	9.59670900
H	1.09251500	6.29187500	9.91984100
C	-1.99828500	3.97500900	8.97110300
H	-1.84474500	5.24902600	10.69469400
C	-0.08418400	2.58513100	8.51816700
H	1.55936100	2.77063400	9.90072100
C	4.01433500	0.58233100	10.41841400
C	1.04489600	-0.12489200	13.43228700
H	-0.17092200	0.73776000	14.99553000
H	2.39461300	-0.75000200	11.90753900
C	4.14378700	7.75711500	9.90383300
H	5.81348700	7.10147300	11.08719300
H	2.29001600	8.13192900	8.87235200
C	-1.36639100	3.01537700	8.18017800
H	-2.99356100	4.32163200	8.71517000
H	0.41586600	1.84101000	7.90747600
C	4.52736800	1.09202300	9.06976900

H	3.18504800	-0.09628100	10.22224700
H	4.80322800	-0.00584300	10.91423800
H	0.66118400	-1.13080800	13.56646400
H	4.68626600	8.56627700	9.42918100
H	-1.86708900	2.61109000	7.30756600
H	4.78444500	0.23506800	8.44230400
H	3.75989300	1.67381500	8.55278300
H	5.42203200	1.70967000	9.16501300
C	4.80329100	3.00840800	13.94027600
H	5.89382600	3.02545000	13.85756700
H	4.52620000	3.55665100	14.84551700
H	4.49173100	1.97082700	14.06624600

TS21

G +ZPE = -1077.641935 a.u

C	-2.39935100	-1.36114600	8.46212100
C	0.34932600	-2.44805900	6.05490100
C	-1.62735500	-0.78280500	9.48661000
C	-3.74379300	-1.65187100	8.68366800
H	-0.04498700	-3.46722100	6.00943500
H	1.41022400	-2.51701200	6.29896200
C	0.15433900	-1.76601200	4.71213200
C	-2.23808600	-0.49843400	10.71773200
C	-4.34324000	-1.35505000	9.90729900
H	-4.33009700	-2.10904000	7.89319700
C	-0.67537100	-2.33572300	3.74314900
C	0.80960700	-0.56406200	4.41948300
H	-1.64437200	-0.07100500	11.51889700
C	-3.58554600	-0.77517500	10.92475900
H	-5.39183600	-1.57822000	10.06486600
H	-1.18099500	-3.27233600	3.95367300
C	-0.85500300	-1.71564700	2.50649300
C	0.63100000	0.05788200	3.18669400
H	1.47117400	-0.11503800	5.15259100
H	-4.04184300	-0.54738600	11.88128400
H	-1.50106700	-2.17051800	1.76438800
C	-0.20389200	-0.51652400	2.22704500
H	1.14799600	0.98606900	2.97138700
H	-0.34090200	-0.03328900	1.26647900
C	0.38168400	-1.19412100	8.16515100
C	-0.20238900	-0.55705600	9.24732900
C	-0.09881300	1.76569300	8.46030400
H	-1.15040000	1.70593600	8.69093300

H	0.23161000	1.52084000	7.46387100
N	0.65789900	2.52220500	9.22613200
C	0.06276200	3.05758400	10.48461400
H	-0.58591500	2.27638600	10.87962600
H	0.87123700	3.19581000	11.19923200
C	-0.72697700	4.34631300	10.26557000
H	-0.10071700	5.14943100	9.87432500
H	-1.55463000	4.18586500	9.57164700
H	-1.14367400	4.67216000	11.22099100
C	2.03445300	2.79060800	8.92012500
C	2.75776100	1.92315800	8.09459300
C	2.66017300	3.92634600	9.44628000
C	4.08696000	2.19909100	7.79016700
H	2.30399400	1.02336300	7.70540100
C	3.99036600	4.19130700	9.13501500
H	2.12225800	4.61546700	10.08144700
C	4.70963800	3.33351600	8.30593900
H	4.63667300	1.51453900	7.15553900
H	4.46067800	5.07772700	9.54314500
H	5.74533900	3.54385000	8.06864800
H	1.45971100	-1.28034600	8.09092800
N	-0.31120300	-1.75682600	7.17240400
C	-1.77742300	-1.62979700	7.10805900
H	-2.02770600	-0.82635400	6.40255700
H	-2.17752300	-2.55459600	6.68504900
H	0.44011100	-0.29882800	10.07839800

int-19

G +ZPE = -1077.655926 a.u

C	-2.36345200	-1.18922500	8.29983900
C	0.98816300	-1.97378200	6.61234600
C	-1.94819600	-0.38777300	9.36232800
C	-3.66247600	-1.70576700	8.27956100
H	0.88425300	-3.03462900	6.84560700
H	1.97346700	-1.64648100	6.94066100
C	0.78670300	-1.72808700	5.13499600
C	-2.83778100	-0.12108900	10.40923600
C	-4.54708000	-1.42000600	9.31270500
H	-3.97774000	-2.33452600	7.45405200
C	0.20375700	-2.70970900	4.32811000
C	1.20941900	-0.52724800	4.55396200
H	-2.51281100	0.48077900	11.25094900
C	-4.13161200	-0.62823300	10.38410500

H	-5.55288100	-1.82198400	9.28879600
H	-0.10967000	-3.65234600	4.76379400
C	0.03181700	-2.48931300	2.96254400
C	1.03539200	-0.30625000	3.19062200
H	1.68540100	0.23074500	5.16526000
H	-4.81163500	-0.41352600	11.19977400
H	-0.41871600	-3.25810600	2.34589200
C	0.44360000	-1.28618600	2.39323700
H	1.36876400	0.62585400	2.74956300
H	0.31180700	-1.11472600	1.33121900
C	0.36687100	-0.50038100	8.44041000
C	-0.54356700	0.16766900	9.39811200
C	-0.52443100	1.74763600	9.22057400
H	-1.29031400	2.11948000	9.90286300
H	-0.85863600	2.00187100	8.21703200
N	0.73470100	2.36848300	9.54273500
C	0.95716700	2.65509100	10.96487700
H	0.38821800	1.91982500	11.53878000
H	2.00488700	2.47441500	11.21248000
C	0.53459500	4.06602600	11.38687900
H	1.10162900	4.82989500	10.85097200
H	-0.52786000	4.22974000	11.18911300
H	0.70497600	4.20342300	12.45805100
C	1.64255200	2.77869600	8.56792900
C	1.41867700	2.54994800	7.19196000
C	2.83961200	3.44366800	8.92052000
C	2.34075400	2.96456100	6.23388300
H	0.51696000	2.06240500	6.84895700
C	3.75239700	3.84361200	7.95142500
H	3.06214400	3.66119800	9.95496200
C	3.51867000	3.61165400	6.59677700
H	2.12325400	2.77986100	5.18745200
H	4.65740800	4.35116500	8.26731500
H	4.23101000	3.93181100	5.84633300
H	1.43565000	-0.35865800	8.56006200
N	-0.00880800	-1.26460500	7.47580300
C	-1.43276300	-1.51683800	7.16225600
H	-1.66481000	-0.92876000	6.26965800
H	-1.51676200	-2.56866900	6.88604700
H	-0.10438100	-0.00956000	10.38676700

TS22

G +ZPE = -1077.626713 a.u

C	1.78120000	5.32754100	11.71446100
C	-0.47601900	3.95099300	14.03999700
C	2.01168200	4.19906500	10.92584300
C	2.82515000	6.21115800	11.99184500
H	0.47527000	3.83284500	14.57397700
H	-0.97744500	2.98019000	14.08410900
C	-1.31852800	4.98322700	14.76950200
C	3.29474000	3.95462400	10.42882700
C	4.09872000	5.97256600	11.48082400
H	2.64265400	7.08209600	12.61232600
C	-1.12658500	5.14718300	16.14594400
C	-2.30769100	5.74245300	14.13675100
H	3.48105800	3.06975200	9.82918200
C	4.33448000	4.84000300	10.70158200
H	4.90710300	6.66038700	11.70011200
H	-0.35312900	4.57658100	16.64973100
C	-1.91497100	6.03313100	16.87653600
C	-3.09257100	6.63629700	14.86452600
H	-2.46217600	5.65422800	13.06816200
H	5.32680400	4.64287900	10.31298600
H	-1.75174300	6.14473700	17.94243000
C	-2.90241200	6.78159300	16.23714400
H	-3.85126000	7.21990600	14.35560400
H	-3.51242500	7.47686600	16.80219500
C	-0.19738800	3.21794900	11.71023200
C	0.86183600	3.26512900	10.59445600
C	0.28538500	3.61180800	9.18520600
H	1.09907500	3.64797000	8.46567800
H	-0.18284300	4.59981700	9.19428300
N	-0.65599600	2.58278900	8.76546600
C	-0.32853700	1.76885200	7.57989300
H	0.75308000	1.81472700	7.45087200
H	-0.57103400	0.72627400	7.78461100
C	-1.03730900	2.25946800	6.31720900
H	-2.12260500	2.20449000	6.42655600
H	-0.76501900	3.29302400	6.09244000
H	-0.74646500	1.63607200	5.46846800
C	-1.67983500	2.30744200	9.59226700
C	-1.87391100	3.19047900	10.73137900
C	-2.52152800	1.17043900	9.44652900
C	-2.94566000	2.87971600	11.64632200
H	-1.77460200	4.24652700	10.49265000
C	-3.48382500	0.90634800	10.38843700
H	-2.41535200	0.51127800	8.59765800

C	-3.69876000	1.75318100	11.51025200
H	-3.13267600	3.56465800	12.46398700
H	-4.10293600	0.02465900	10.26804500
H	-4.47654200	1.51337000	12.22385800
H	-0.28020900	2.24142100	12.17178300
N	-0.20698300	4.23395100	12.62629000
C	0.38733200	5.52473000	12.26155500
H	-0.24979900	6.03194500	11.52609400
H	0.40206400	6.15969200	13.14523600
H	1.25483400	2.25089700	10.50164200

int-20

G +ZPE = -1077.627937 a.u

C	2.05805800	4.74601600	12.28392000
C	-1.38863100	4.07866900	13.64112800
C	2.05844100	4.03323900	11.08540600
C	3.15419900	5.55410900	12.60715100
H	-1.08373600	3.45484500	14.49775400
H	-2.28151600	3.61890000	13.21758700
C	-1.78537800	5.45478000	14.14468600
C	3.15967800	4.13124300	10.22466600
C	4.24252200	5.65130000	11.74798300
H	3.14959000	6.10954300	13.53947300
C	-1.78653600	5.73928400	15.51229100
C	-2.20036000	6.44791900	13.24934000
H	3.16731100	3.56684300	9.29752600
C	4.24666100	4.93399900	10.54989800
H	5.08413100	6.28286400	12.00841300
H	-1.46357000	4.97978200	16.21689200
C	-2.19558400	6.98863200	15.98081500
C	-2.60541700	7.69646000	13.71349900
H	-2.20057500	6.23927800	12.18546100
H	5.09333700	4.99924900	9.87640300
H	-2.18922500	7.19307500	17.04560200
C	-2.60520800	7.97082600	15.08230400
H	-2.92473700	8.45584400	13.00832200
H	-2.92118500	8.94304500	15.44323900
C	-0.07986700	2.87317200	11.88266300
C	0.88569900	3.14993700	10.71686800
C	0.08504300	3.74033200	9.54626600
H	0.73576200	4.07643800	8.74362800
H	-0.50969900	4.59227400	9.88923300
N	-0.79544800	2.69431000	8.99964300

C	-0.89655300	2.59100900	7.52444700
H	0.06594000	2.91289100	7.12892600
H	-1.01548300	1.54536100	7.24940700
C	-2.02835100	3.45036400	6.96251900
H	-2.99923100	3.12680200	7.34315200
H	-1.88819000	4.50319600	7.21474600
H	-2.03832400	3.35869500	5.87454400
C	-1.43819300	1.91003900	9.83869100
C	-1.41218700	2.25406100	11.31512300
C	-2.23985200	0.80613400	9.40176700
C	-1.97510700	1.18705200	12.20236400
H	-2.14437600	3.08210200	11.33910800
C	-2.80064800	-0.04042100	10.30628200
H	-2.37235000	0.61674400	8.34794400
C	-2.64023200	0.12212000	11.72669000
H	-1.84786000	1.32306700	13.26869900
H	-3.37618700	-0.88330000	9.94151900
H	-3.05878700	-0.61878600	12.39528000
H	0.37570500	2.12009900	12.54382800
N	-0.33137400	4.13140000	12.61391900
C	0.90780800	4.60849100	13.24437800
H	0.70036400	5.57595700	13.70377700
H	1.20529700	3.92993500	14.06677600
H	1.27968000	2.18593100	10.37745900

D_1'

G +ZPE = -1077.240823 a.u

C	2.21594500	4.66784200	12.32363000
C	-1.31919900	3.90791500	13.40918700
C	2.33585500	3.89575600	11.16322000
C	3.27033400	5.50545500	12.70569100
H	-1.07721500	3.38199000	14.35010700
H	-2.09974600	3.32740700	12.91634500
C	-1.87477800	5.28054600	13.74209200
C	3.51246200	3.97306900	10.40712000
C	4.43411500	5.57955300	11.94758400
H	3.17458200	6.10405100	13.60647300
C	-2.07172800	5.67126700	15.06912200
C	-2.24022700	6.16821900	12.72197900
H	3.60829900	3.36636200	9.51182500
C	4.55750000	4.80660300	10.79156500
H	5.24112100	6.23512400	12.25509500
H	-1.78874300	4.99662700	15.87090500

C	-2.62433600	6.91599700	15.37560600
C	-2.78890600	7.41186200	13.02339400
H	-2.08701600	5.87808900	11.68871100
H	5.46282500	4.85300100	10.19681000
H	-2.76814800	7.20146100	16.41191300
C	-2.98421000	7.79058100	14.35311100
H	-3.06762900	8.08672700	12.22134100
H	-3.41146200	8.75914700	14.58750200
C	0.24011900	2.67327200	11.89196400
C	1.19680600	2.99912600	10.73145100
H	1.60910400	2.05555900	10.35715800
C	0.39243300	3.65116200	9.59778100
H	1.05289000	3.85219600	8.75099000
H	0.00533900	4.61384600	9.95513300
N	-0.68100700	2.78019500	9.12893600
C	-1.21477400	3.06784900	7.79960100
H	-0.38000700	3.43054900	7.19506800
H	-1.54213900	2.13640300	7.33114900
C	-2.34936900	4.10051800	7.77741600
H	-3.20945900	3.75864000	8.35793500
H	-2.01671500	5.05573100	8.19154500
H	-2.68109700	4.27435300	6.74985800
C	-1.34341500	1.95400400	10.01874300
C	-0.90678200	1.83391800	11.36721200
C	-2.44087100	1.15807700	9.61072100
C	-1.50454000	0.88451000	12.19805300
C	-3.03343500	0.24655100	10.47482100
H	-2.82935500	1.24359500	8.60572700
C	-2.56460300	0.08629500	11.77784100
H	-1.12704100	0.78164200	13.21090400
H	-3.86829500	-0.34753300	10.11812900
H	-3.01478200	-0.63483800	12.44930500
H	0.78055600	2.06640000	12.63911100
N	-0.16503700	3.96559600	12.50707100
C	0.98182900	4.56836700	13.19008800
H	0.69737400	5.56664300	13.53065400
H	1.23630700	3.99059800	14.10194000

TS1

G +ZPE = -544.903059 a.u

C	-1.95110600	0.25090600	-1.42135500
O	-1.04353000	-0.02285800	-0.64485200
H	-2.08039800	1.28149900	-1.78199600

H	-2.62165600	-0.51112700	-1.82858400
C	-4.76961000	-0.87194400	0.67095800
H	-5.18321500	-1.38395100	-0.21628500
H	-5.42343600	-0.00450800	0.86372800
H	-4.91058900	-1.56226500	1.52095000
O	-3.44007800	-0.48844800	0.48834500
C	0.13573700	-3.55410100	1.68444400
H	-0.25541400	-4.33959300	1.01134300
H	0.40379600	-4.06592100	2.62577400
H	1.08984100	-3.21450500	1.24025000
O	-0.76635300	-2.51840100	1.90383500
Mg	-1.76178100	-1.30074400	0.90428400

int-21

G +ZPE = -544.916387 a.u

C	-2.50116000	0.46030800	-0.67916000
O	-1.16451700	0.35024700	-0.59105400
H	-2.91477900	1.42076400	-0.32359000
H	-2.92711500	0.22304700	-1.67205000
C	-4.41668900	-0.92610600	0.12494300
H	-4.63412500	-1.30713800	-0.87865900
H	-5.04661300	-0.05353400	0.32438600
H	-4.63460800	-1.69918000	0.86079200
O	-3.03875500	-0.58567100	0.25104800
C	-0.04731300	-3.81503600	1.80717800
H	0.44127300	-4.05006600	2.76966700
H	0.52881600	-4.35552100	1.03336200
H	-1.04162800	-4.29729700	1.84446000
O	-0.10349500	-2.44438200	1.58147600
Mg	-1.07708500	-1.22639300	0.56804200

int-22

G +ZPE = -1948.584984 a.u

C	-1.23486600	0.30672000	0.31426400
C	-0.11309100	0.46146400	-0.53398700
C	0.17241300	1.77246300	-1.03997900
C	-0.67107300	2.87359600	-0.77030100
C	-1.76970400	2.73607400	0.11019200
C	-2.00918000	1.43931900	0.67384100
H	-1.38454800	-0.63495400	0.83913400
H	1.07215300	1.92675700	-1.62213000
H	-0.38867000	3.84926000	-1.13824000

H	-2.78903600	1.32983900	1.41723600
C	-2.68512200	3.88406900	0.48635100
H	-2.96359100	3.73055200	1.53432600
C	-2.05044900	5.27323800	0.35984300
H	-2.74231900	6.02355100	0.74885400
H	-1.11900700	5.34030000	0.92496300
H	-1.84501300	5.53119900	-0.68303800
C	-3.97155500	3.77944200	-0.36232000
H	-4.46487800	2.81312200	-0.23483100
H	-4.67341100	4.56103100	-0.06240600
H	-3.74728800	3.91202500	-1.42440600
C	0.78046700	-0.69296200	-0.87348200
H	0.68471200	-1.49886500	-0.14687100
H	0.49420900	-1.08397300	-1.85569700
H	1.82524900	-0.38404800	-0.93057500
Ru	0.05123300	1.93260700	1.14409400
O	0.69355900	0.81233400	2.73165800
C	1.21922800	1.23829800	3.94457800
H	0.64438800	2.05181300	4.39727600
H	2.28027100	1.50857500	3.87729800
O	1.08987300	0.04833700	4.75895000
C	1.85719400	0.03084200	5.96903100
H	1.54679400	0.84903600	6.62375500
H	1.65501900	-0.92091000	6.45577900
H	2.92506400	0.11526700	5.74876300
C	-2.29914300	-2.07495000	3.15100000
H	-3.06705000	-1.57561700	2.53162400
H	-2.64330300	-3.11587800	3.28177000
H	-2.35493500	-1.60334000	4.14851800
O	-1.03297600	-2.00601500	2.56849300
Mg	0.60176500	-1.22016300	3.11570100
Cl	2.65247600	-2.25112600	2.53417700
Cl	1.15059200	3.88740000	2.07883100

int-23

G +ZPE = -1172.969884 a.u

C	-1.25148300	1.18104500	0.07881700
C	-0.06520700	1.36335400	-0.67997100
C	0.23067300	2.68112600	-1.15868800
C	-0.62776600	3.76925800	-0.89368300
C	-1.75917900	3.61392800	-0.06038900
C	-2.04599100	2.29566800	0.43089600
H	-1.47112800	0.21126600	0.50586800

H	1.15264000	2.85117700	-1.69931700
H	-0.32702900	4.75587600	-1.21551300
H	-2.87818000	2.15815200	1.11054700
C	-2.68183000	4.75781800	0.31436300
H	-3.04061100	4.54787800	1.32765500
C	-2.00746000	6.13441000	0.32814700
H	-2.70469400	6.87727000	0.72232300
H	-1.11387900	6.13465400	0.95577000
H	-1.72412900	6.45704400	-0.67784000
C	-3.90440100	4.74364200	-0.62796900
H	-4.42489400	3.78337300	-0.60057100
H	-4.61156400	5.52116600	-0.32894000
H	-3.60085200	4.93806500	-1.66059300
C	0.85216900	0.21535900	-0.98238900
H	0.77686200	-0.56426800	-0.22415100
H	0.57552000	-0.22115800	-1.94791900
H	1.88931700	0.54643800	-1.04959400
Ru	0.00180800	2.74495200	1.04577900
O	0.38430500	1.67714900	2.65779100
C	1.18798300	2.00153000	3.73438200
H	0.81271800	2.88533400	4.27800300
H	2.22921500	2.19977900	3.42844000
O	1.16659000	0.87509800	4.60679200
C	1.97627100	1.07858100	5.75240000
H	1.63511200	1.93936400	6.34475800
H	1.90368200	0.17941300	6.36449900
H	3.02960900	1.24018200	5.48278800
Cl	1.37212100	4.59746400	1.87113400

TS2

G +ZPE = -1172.96371 a.u

C	-1.92691400	0.18264000	-0.11702700
C	-0.69010300	-0.00368100	-0.75675100
C	0.17771700	1.13461400	-0.88862500
C	-0.20508300	2.40616100	-0.43185100
C	-1.45577500	2.59589900	0.23952900
C	-2.28700600	1.45926600	0.40760000
H	-2.57827100	-0.66534300	0.05107500
H	1.16043100	1.00018000	-1.32044600
H	0.48771400	3.22973600	-0.53434900
H	-3.21459600	1.56061400	0.95706700
C	-1.93157300	3.95575400	0.71796300
H	-2.59284500	3.77369400	1.57144400

C	-0.81119000	4.89690600	1.17844600
H	-1.24889100	5.80208900	1.60524300
H	-0.18105200	4.43530900	1.94157800
H	-0.17187500	5.20655800	0.34725600
C	-2.77408300	4.60627600	-0.39964300
H	-3.61155600	3.96888500	-0.69231000
H	-3.17667700	5.56162500	-0.05430300
H	-2.16330400	4.79610400	-1.28706700
C	-0.25936100	-1.34378600	-1.27514400
H	-0.33252300	-1.35357800	-2.36738200
H	0.77742800	-1.54906800	-1.00405100
H	-0.88915700	-2.14223800	-0.88224000
Ru	-0.31419300	0.96331500	1.29273000
O	-0.99763400	0.41836200	3.21792000
C	-0.34876300	1.47259500	3.61853500
H	-0.92873700	2.38316600	3.84052800
H	0.34170300	1.95791700	2.55628200
O	0.63248300	1.24638200	4.55244400
C	1.27588800	2.42787300	5.04282400
H	0.54262300	3.15646500	5.40301900
H	1.91109700	2.11347100	5.86787600
H	1.89384600	2.88455200	4.26258000
Cl	1.70248200	-0.45368000	1.73493500

int-24

G +ZPE = -1172.990867 a.u

C	-1.74648600	0.83942900	0.68625400
C	-0.62536200	0.53890300	-0.17498300
C	0.02652100	1.61123200	-0.76163800
C	-0.41702800	2.95740300	-0.50290800
C	-1.64661700	3.24208300	0.14332600
C	-2.31392400	2.13114600	0.75440200
H	-2.22577200	0.02947700	1.22308200
H	0.92205300	1.45349800	-1.34959600
H	0.17086500	3.77279700	-0.90181500
H	-3.22213000	2.28922200	1.32159200
C	-2.27624400	4.62358300	0.17944100
H	-2.87129000	4.67281000	1.09726800
C	-1.27016200	5.78015100	0.21242200
H	-1.80409300	6.72671200	0.32732500
H	-0.57241700	5.67891200	1.04605200
H	-0.69264100	5.84412500	-0.71415900
C	-3.24291400	4.76599600	-1.01415400

H	-3.99306600	3.97115700	-1.01970700
H	-3.76564200	5.72473700	-0.96358500
H	-2.69819600	4.72561800	-1.96219400
C	-0.17781500	-0.88364500	-0.35146700
H	-0.09924500	-1.39133900	0.61309800
H	-0.90962400	-1.43610300	-0.94982300
H	0.78799000	-0.93857300	-0.85461900
Ru	-0.28404900	2.24122800	1.58060600
O	0.25886400	0.82420000	3.17427400
C	0.72566400	1.11017600	4.26653600
H	-0.81201500	3.16829700	2.75164100
H	0.87930000	2.14148700	4.59756600
O	1.07268400	0.15972200	5.10267300
C	1.60434700	0.53705500	6.39899800
H	0.90503000	0.18955700	7.15671300
H	2.56411000	0.03767600	6.50919000
H	1.73069600	1.61783200	6.46510000
Cl	1.92378700	3.33850900	2.05254200

int-25

G +ZPE = -947.431605 a.u

C	-2.56762600	0.62033500	-0.72586800
O	-1.25572900	0.62754100	-0.72354200
H	-3.05304200	1.53182400	-0.30236000
H	-3.05016800	0.41613600	-1.71172200
C	-4.44124900	-0.93692600	0.09611200
O	-3.07535400	-0.51024800	0.18625200
C	-4.65438000	-1.85319000	1.30834000
H	-5.67115600	-2.25639800	1.32497300
H	-3.95264400	-2.69150900	1.27757500
H	-4.48590200	-1.29898200	2.23556900
C	-5.41294600	0.25427500	0.17626500
H	-5.32360700	0.90623900	-0.69544200
H	-6.44697200	-0.09955500	0.22517400
H	-5.21099600	0.84913100	1.07113200
C	-4.67832400	-1.73676000	-1.19942400
H	-3.98160600	-2.57802500	-1.25206900
H	-5.69787900	-2.13280300	-1.23275900
H	-4.53109500	-1.11465100	-2.08484500
K	-0.59241300	-1.27580700	0.78085600

int-26

G +ZPE = -2351.115885 a.u

C	-0.71921700	0.17210700	-0.11710500
C	0.34887700	0.71801800	-0.89814500
C	0.31628100	2.10699500	-1.21030700
C	-0.68499600	2.94430200	-0.68582400
C	-1.69741600	2.42527900	0.17172700
C	-1.71845100	1.01204500	0.40514500
H	-0.70808400	-0.87799600	0.15813600
H	1.13777500	2.54347300	-1.76281800
H	-0.61302800	4.01032900	-0.84846200
H	-2.46677600	0.59813000	1.06959500
C	-2.77292400	3.29960600	0.78622200
H	-3.11636000	2.78282700	1.68796800
C	-2.28959700	4.69573100	1.19880000
H	-3.09069300	5.21585900	1.72880000
H	-1.42340900	4.63706600	1.86156800
H	-2.02054900	5.30808900	0.33402300
C	-3.96480700	3.38093900	-0.19117100
H	-4.34255100	2.38826300	-0.44742900
H	-4.77965500	3.94699700	0.26651100
H	-3.67712300	3.88671100	-1.11717400
C	1.45478300	-0.16050000	-1.40484200
H	1.64270900	-0.99267400	-0.72591500
H	1.16435900	-0.57762100	-2.37516200
H	2.37778900	0.40323300	-1.54317000
Ru	0.25562000	1.81636200	1.02072100
O	0.41635400	0.91748100	2.79666300
C	1.25239800	1.29835300	3.84118800
H	1.00187000	2.30300900	4.20424300
H	2.30693800	1.27984300	3.54146100
O	1.01934200	0.33633900	4.87782300
C	1.86643200	0.37805400	6.05992300
Cl	-1.13699800	-3.29377000	1.37650100
Cl	1.96622000	3.46748400	1.60960200
K	-0.80437300	-1.31453600	3.73665700
C	1.26671300	-0.69078300	6.97649900
H	1.29576600	-1.67033300	6.49244600
H	1.82952400	-0.75360100	7.91057000
H	0.22754400	-0.45018900	7.21421800
C	1.79360500	1.75519100	6.73452500
H	2.31371600	1.72692200	7.69524800
H	2.26201800	2.53350300	6.12816800
H	0.75274300	2.03529400	6.91603000
C	3.31388500	0.01301400	5.69893200

H	3.91169000	-0.08719100	6.60843000
H	3.34126300	-0.93901900	5.16292100
H	3.78693200	0.77549500	5.07669000

int-27

G +ZPE = -1290.79235 a.u

C	-0.37297500	1.18696600	0.11975400
C	0.63313100	1.75655100	-0.72723400
C	0.41955700	3.06931700	-1.23211900
C	-0.69064900	3.83092700	-0.82441400
C	-1.64268900	3.30470500	0.09702700
C	-1.49033600	1.94312400	0.51607900
H	-0.22396400	0.19636000	0.53027800
H	1.18582600	3.53180600	-1.84005300
H	-0.75594900	4.86489700	-1.13230400
H	-2.19053400	1.52639700	1.22891800
C	-2.83706000	4.10557000	0.57906500
H	-3.14401700	3.66419500	1.53248000
C	-2.54017300	5.59078700	0.82236000
H	-3.41499400	6.06869300	1.26916100
H	-1.69547800	5.72086900	1.50238400
H	-2.31886000	6.12146900	-0.10753900
C	-3.99734200	3.92001800	-0.42126200
H	-4.24319400	2.86443200	-0.55968900
H	-4.88990500	4.43127100	-0.05256300
H	-3.74227000	4.34216400	-1.39745000
C	1.85098400	0.96694200	-1.11000700
H	2.13648500	0.27044400	-0.32085500
H	1.63359100	0.38346100	-2.01114900
H	2.69550900	1.62132200	-1.32764900
Ru	0.34503100	3.06725000	1.03332200
O	0.51086700	2.46348400	2.90037400
C	1.24340300	3.05180300	3.93945800
H	1.02836300	4.12193700	4.01650600
H	2.32056600	2.92385800	3.77239200
O	0.85395800	2.49750500	5.17977200
C	1.58327000	1.35241500	5.70155700
Cl	1.90644900	4.92432400	1.40137600
C	0.73479100	0.89870800	6.89169600
H	1.20453700	0.05475700	7.40284900
H	0.61852200	1.71687300	7.60672300
H	-0.25843400	0.59272800	6.55420300
C	1.70037500	0.22295000	4.66983800

H	0.71218800	-0.08706300	4.32529200
H	2.28349800	0.53120700	3.80004300
H	2.19889400	-0.63894700	5.12243200
C	2.97148600	1.80202400	6.18182700
H	2.87441700	2.61828100	6.90201600
H	3.49558800	0.97352800	6.66593400
H	3.59339100	2.14647400	5.35199400

TS3

G +ZPE = -1290.781851 a.u

C	-2.04462100	0.21624100	-0.11429300
C	-0.85637000	-0.06242800	-0.81058100
C	0.06626800	1.01853800	-1.02463100
C	-0.21773900	2.32382900	-0.59260500
C	-1.41660500	2.60634300	0.13720900
C	-2.30279300	1.52670700	0.38585600
H	-2.73323800	-0.58671200	0.11551500
H	1.01574500	0.81365800	-1.50090900
H	0.51405300	3.10154500	-0.75965700
H	-3.19358700	1.69931100	0.97681200
C	-1.78902900	4.00571900	0.59356900
H	-2.36301100	3.89208300	1.51915900
C	-0.59160200	4.91858700	0.88416100
H	-0.94402200	5.85474900	1.32272800
H	0.10809200	4.45968000	1.58545700
H	-0.04418700	5.17274600	-0.02766900
C	-2.71950700	4.64035600	-0.46181500
H	-3.61071500	4.03123100	-0.62890600
H	-3.04047300	5.63027100	-0.12836700
H	-2.20088600	4.75586400	-1.41800000
C	-0.53220600	-1.44050400	-1.30741200
H	-1.17992600	-2.18930100	-0.85087200
H	-0.67241200	-1.48062200	-2.39242300
H	0.50637400	-1.69619200	-1.09155200
Ru	-0.31961300	0.93833800	1.18486700
O	-0.92967200	0.44032400	3.13749700
C	-0.22122300	1.47019300	3.52184300
H	-0.76376300	2.39803800	3.74482300
H	0.42884700	1.90905400	2.42115800
O	0.77285200	1.18554000	4.41821000
C	1.34261500	2.25796400	5.25270800
C	0.27610300	2.78140500	6.22089800
H	0.72867300	3.48809300	6.92067500

H	-0.53194500	3.30376100	5.70391700
H	-0.15325100	1.95660300	6.79409400
C	1.91857700	3.37489000	4.37728900
H	2.45490700	4.09133900	5.00400400
H	2.62071300	2.96755100	3.64635100
H	1.13861200	3.92565000	3.84674800
C	2.45657100	1.54252500	6.01566500
H	3.20016500	1.14500700	5.32179900
H	2.95208000	2.23589200	6.69881900
H	2.04695800	0.71408300	6.59765600
Cl	1.66097200	-0.55274800	1.55095200

int-28

G +ZPE = -1290.813539 a.u

C	-1.71762700	0.87879200	0.54969100
C	-0.53874200	0.64376500	-0.25287500
C	0.12490500	1.75750600	-0.74045300
C	-0.36236100	3.08026300	-0.44211100
C	-1.63579100	3.30777400	0.13767600
C	-2.31638900	2.15451100	0.64725600
H	-2.21190200	0.03327300	1.01313000
H	1.05907500	1.64743800	-1.27702900
H	0.23136400	3.92597400	-0.76139700
H	-3.26228600	2.26599200	1.16141400
C	-2.29687300	4.67333300	0.20346100
H	-2.95468900	4.66101900	1.07873300
C	-1.32163800	5.84473900	0.36837900
H	-1.88296400	6.77372200	0.49570100
H	-0.68136300	5.71046600	1.24248400
H	-0.68293200	5.96953000	-0.51060900
C	-3.18205100	4.86272300	-1.04559000
H	-3.91175900	4.05529400	-1.14614000
H	-3.72869200	5.80718400	-0.98088600
H	-2.57232000	4.88457700	-1.95384300
C	-0.05150200	-0.75924100	-0.47531800
H	-0.03376600	-1.32052600	0.46217800
H	-0.72603100	-1.28785300	-1.15683300
H	0.94961600	-0.76996300	-0.90743200
Ru	-0.34602200	2.25642300	1.60757200
O	0.09184000	0.77105800	3.16528000
C	0.47516500	1.01383500	4.30475600
H	-0.96050300	3.12045400	2.78456900
H	0.61719000	2.03630300	4.65871800

O	0.72863600	0.03169400	5.12999900
C	1.18103000	0.31305200	6.53919800
C	1.33842800	-1.08802600	7.11807300
H	2.07935100	-1.65633900	6.55261000
H	1.66995500	-1.02082300	8.15613300
H	0.38805000	-1.62445200	7.09066600
C	2.51552300	1.05332200	6.49135200
H	2.41595600	2.05797900	6.07573600
H	2.90555800	1.15251600	7.50664100
H	3.24289800	0.49627500	5.89735900
C	0.08868000	1.09855000	7.26139500
H	-0.03497700	2.10419200	6.85447900
H	-0.86659600	0.57298000	7.20159700
H	0.35867500	1.20030100	8.31466800
Cl	1.81503800	3.34465600	2.27325200

int-29

G +ZPE = -829.614872 a.u

C	-2.54027100	0.38039900	-0.78292200
O	-1.24473600	0.52684600	-0.68693900
H	-3.16403500	1.27247700	-0.50407500
H	-2.92764300	0.01509600	-1.77241700
C	-4.42674200	-0.88431800	0.12063000
H	-4.75166500	-1.20384000	-0.88259500
H	-4.98320000	0.03251800	0.37343000
H	-4.70738300	-1.66254200	0.83487400
O	-3.03464900	-0.69024000	0.18591400
K	-0.57512200	-1.13005200	1.09879600

int-30

G +ZPE = -2233.298213 a.u

C	-0.83207500	0.15951600	0.04143000
C	0.23484900	0.60218400	-0.80248200
C	0.24827300	1.96540000	-1.21767500
C	-0.71050100	2.87823300	-0.74148900
C	-1.71743400	2.46654000	0.17733600
C	-1.78189500	1.07728200	0.52360800
H	-0.85178000	-0.86618900	0.39693800
H	1.07129800	2.32579100	-1.82060600
H	-0.60319900	3.92496300	-0.98792100
H	-2.52410500	0.74710400	1.23980500
C	-2.74286800	3.42589400	0.74882300

H	-3.07392700	2.99694600	1.69999400
C	-2.20055400	4.83303600	1.02919900
H	-2.96447000	5.42142100	1.54245700
H	-1.31191300	4.79836700	1.66326300
H	-1.94585200	5.36365700	0.10790200
C	-3.96220200	3.47060100	-0.19653900
H	-4.38186500	2.47505400	-0.35924400
H	-4.74192800	4.10148300	0.23685600
H	-3.68699900	3.88911400	-1.16877900
C	1.29328000	-0.35473400	-1.26620800
H	1.45871400	-1.14778800	-0.53661400
H	0.96689700	-0.82163600	-2.20183300
H	2.23631400	0.15820400	-1.45698500
Ru	0.23362100	1.84923300	1.02478700
O	0.44572600	1.04985700	2.84276200
C	1.33536400	1.44729300	3.82526000
H	1.14410600	2.47533700	4.17035400
H	2.38309700	1.37542800	3.49498000
O	1.13589300	0.54772400	4.92241800
C	2.00632500	0.81657800	6.01338100
H	1.84927900	1.82766900	6.41006400
H	1.78150600	0.08964300	6.79325100
H	3.05856000	0.71310100	5.71954300
Cl	-1.22663000	-3.19280300	1.76061900
Cl	1.94847700	3.53138800	1.49464600
K	-0.79752600	-1.08936000	3.98733300

[$RuCl_2$]

G +ZPE = -1403.658702 a.u

C	-1.99058200	0.68511500	-0.66000900
C	-0.65853400	0.55003000	-1.15049400
C	0.13195000	1.71898400	-1.18439300
C	-0.40336200	2.98248300	-0.79753800
C	-1.76949500	3.14048000	-0.43094600
C	-2.54334600	1.95863700	-0.34194100
H	-2.58956900	-0.20234900	-0.50026700
H	1.18533400	1.64683500	-1.42198600
H	0.26531900	3.83122800	-0.75474400
H	-3.54767200	2.00668400	0.05914100
C	-2.38856100	4.48209800	-0.09606500
H	-3.16203100	4.28956300	0.65357900
C	-1.40745300	5.50462300	0.48935200
H	-1.95801000	6.39560800	0.79919400

H	-0.89071000	5.10505200	1.36382600
H	-0.66254700	5.82279700	-0.24532600
C	-3.07984700	5.03084000	-1.36222700
H	-3.82115700	4.32985000	-1.75289400
H	-3.58872600	5.96870200	-1.12736900
H	-2.34835900	5.22889300	-2.15083700
C	-0.10221800	-0.78853300	-1.52899900
H	-0.56330800	-1.58801300	-0.94847800
H	-0.31169900	-0.97465400	-2.58730500
H	0.97741100	-0.82489300	-1.38479900
Ru	-0.72827500	1.59265700	0.86013900
Cl	-1.37712200	2.52268700	2.99688200
Cl	0.73017300	0.02636600	1.98201700

[RuHCl]

G +ZPE = -943.956897 a.u

C	-2.03791200	0.66582800	-0.46992000
C	-0.75510800	0.52471600	-1.06399900
C	0.02680300	1.70927400	-1.13626400
C	-0.50654200	2.94457400	-0.65957200
C	-1.92070700	3.11111700	-0.39067800
C	-2.66389400	1.94950300	-0.28191900
H	-2.61980100	-0.22771900	-0.27698900
H	1.05421900	1.66503000	-1.47261600
H	0.14317100	3.80978100	-0.65378700
H	-3.70146800	1.98985400	0.02669800
C	-2.54561300	4.47984700	-0.21180900
H	-3.52658800	4.32022900	0.24696200
C	-1.74120000	5.41349200	0.70621300
H	-2.30064000	6.33837800	0.86731900
H	-1.54836700	4.95040900	1.67544200
H	-0.77971300	5.68693400	0.26260400
C	-2.77321100	5.12411100	-1.59523000
H	-3.39454600	4.49043600	-2.23256200
H	-3.27436200	6.08870100	-1.47936000
H	-1.82311300	5.29776300	-2.10920300
C	-0.24125700	-0.80638200	-1.53394200
H	-0.66037600	-1.62140400	-0.94158000
H	-0.53432300	-0.96602400	-2.57675800
H	0.84663000	-0.85716900	-1.47729000
Ru	-0.55564500	1.49203700	0.93184000
Cl	-0.11837300	2.68957300	3.01677900
H	0.82058100	0.74695300	1.20024500
